# Therapeutic Vaccines for Hematological Cancers: A Scoping Review of This Immunotherapeutic Approach as Alternative to the Treatment of These Malignancies

**DOI:** 10.3390/vaccines13020114

**Published:** 2025-01-23

**Authors:** Fernando Augusto Siqueira Mathias, Maria Gabriela Reis Carvalho, Jeronimo Conceição Ruiz

**Affiliations:** 1 Grupo de Informática de Biossistemas, Instituto René Rachou, Fundação Oswaldo Cruz, Belo Horizonte 30190-002, Brazil; fasmathias@gmail.com; 2 Biologia Computacional e Sistemas (BCS), Instituto Oswaldo Cruz (IOC), Fundação Oswaldo Cruz, Rio de Janeiro 21040-900, Brazil

**Keywords:** immunotherapy, vaccine, hematological cancers

## Abstract

**Background/Objectives**: The need for innovative cancer treatments has brought immunotherapies to the forefront as a promising approach, with therapeutic vaccines demonstrating the potential to mobilize immune cells to eliminate tumor cells. However, challenges such as genetic variability among patients, immune evasion mechanisms, and disease relapse contribute to the complexity of achieving an ideal therapy, especially for hematological cancers. This review systematically identifies and analyzes recent studies focused on the development of therapeutic immunotherapy vaccines, examining critical aspects such as development stages, key assays for therapeutic validation, treatment outcomes, and study limitations. **Methods**: A scoping review was conducted following the PRISMA extension guidelines (PRISMA-ScR). Literature searches were conducted across Scopus, PubMed, Web of Science, and Science Direct databases using keywords including “immunotherapy”, “vaccines”, “immunization”, “hematological malignancies”, “blood cancer”, “hematopoietic neoplasms”, and “leukemia”. **Results**: A total of 56 articles published from 2013 to 2024 were included in the analysis. The majority of studies are in the preclinical stage, with some advancing to phase 1 and phase 2 clinical trials. Acute myeloid leukemia emerged as the most frequently studied malignancy. While first- and second-generation vaccines dominate the field, innovative approaches, such as dendritic-cell-based vaccines and mRNA vaccines, are gaining prominence. Notably, preclinical models often demonstrate superior outcomes compared to clinical trials, as results observed in animal models are not fully replicated in human studies. **Conclusions**: Despite challenges related to disease progression and patient loss, the studies reviewed highlight significant advancements in patient prognosis, emphasizing the potential of novel therapeutic vaccines as an effective alternative for the treatment of hematological cancers.

## 1. Introduction

Hematological cancers, encompassing various conditions such as leukemias, lymphomas, and myelomas, continue to pose complex challenges in the field of oncology. A study published in 2023, analyzing data on hematological malignancies from 1990 to 2019, highlighted that these cancers are among the most prevalent, with global incidence and mortality rates increasing over this period. Additionally, the age-standardized death rate for all hematologic malignancies is declining [1]. The International Agency for Research on Cancer (WHO), available in the Global Cancer Observatory (GLOBOCAN), a public domain, shows that in 2022, the absolute number of leukemias, multiple-myelomas, Hodgkin and non-Hodgkin lymphomas is more than 1.3 million. In addition to that, the cost of treatments per patient is still a challenge for the health system. In the United States, for example, the treatment cost average in 12 months, including chemotherapy, biologic agents, and immunotherapy, is about $180,000 [2]. These data underscore the need for advancing cancer treatment strategies.

The study of these hematological malignancies has been an important tool in malignant diseases at all because the affected organ is already systemic, which increases the challenge of treating this type of cancer [3]. The immune cell origin of hematologic malignancies offers a unique opportunity to understand both the mechanisms of immune responsiveness and immune escape, thereby accelerating the progress of immunotherapy [4]. Traditionally, treatments such as chemotherapy and hematopoietic stem cell transplantation have been pivotal in addressing these malignancies. Nevertheless, conventional treatments face challenges when dealing with toxicity, non-responsive patients, immunologically vulnerable patients, or those experiencing disease relapses with poor prognosis. As an alternative, the use of immunotherapy like immune-checkpoint blockade has revolutionized cancer treatment. Nonetheless, there are still subsets of patients across multiple cancers who do not respond to these agents [5]. The tireless search for more effective and less invasive approaches has led to therapeutic vaccines and their potential to overcome the immune system anergy, emerging as promising candidates.

The ability of immune system cells to eradicate cancers was first demonstrated through the graft-versus-leukemia (GVL) effect, where allogeneic hematopoietic cell transplantation enables donor T cells to recognize and target leukemia-associated antigens [6]. The GVL reaction is an important example of immune-mediated tumor destruction where the coordinated humoral and cellular response results in tumor cell killing [7]. However, there is a risk, depending on the genetic disparity between patients and donor, of disease relapse or development of graft-versus-host-disease [8,9].

Over the past few decades, cancer immunotherapy has progressed from a promising experimental approach to an established clinical strategy, with cancer vaccines being extensively investigated as a subset of immunotherapeutics for a wide range of malignancies [10], including hematological cancers. In this sense, as we better understand the biology of these diseases, the need for more efficient therapeutic strategies becomes evident. The therapeutic vaccines are designed to target specific tumor-associated antigens, representing an innovative approach to elicit selective immune responses and have as their role to effectively stimulate an antitumor cytotoxic lymphocyte (CTL) response [11].

While therapeutic vaccination holds promise as a cancer immunotherapy approach, one major challenge is tumor heterogeneity, stemming from the genetic and molecular diversity between patients, which often leads to variations in the antigens expressed on cancer cell surfaces. Personalized cancer vaccines, which have gained traction in recent years, offer a potential solution. However, this strategy is hindered by time-consuming production processes, high costs, limited accessibility, and the targeting of only a small number of tumor antigens [12]. On the other hand, the precise identification of antigens such as overexpressed proteins or neoantigens derived from genetic mutations could be a key factor in designing these vaccines. By customizing vaccines based on these markers, the goal is not only to maximize therapeutic efficacy but also to minimize undesirable side effects. There are many immunization strategies and kinds of antigen sources including tumor cell lysates, synthetic peptides, and cell exosomes, among others [13].

In the development of these strategies, the preclinical phase assumes a crucial role. According to Olson and colleagues (2018), there are four preclinical models to study cancer immunotherapy with their advantages and disadvantages that yield different responses: (1) syngeneic tumor cells; (2) genetically engineered; (3) patient-derived xenograft; and (4) humanized patient-derived xenograft [5]. Additionally, non-human primate models (NHP), such as the rhesus macaque, play a significant role due to their genetic and immunological similarities to humans. These models are particularly useful for evaluating the safety, immunogenicity, and efficacy of vaccine candidates in a system that closely replicates human physiology [14]. Overall, experimental models provide crucial insights, allowing for a more accurate simulation of interactions between the immune system and cancer cells. This preclinical stage not only validates the feasibility of vaccines but also informs the design of more robust clinical trials.

The progression of these innovative approaches to clinical trials reveals a promising outlook. Phase I, II, and III trials demonstrate the ongoing need for refinement in the studies. The Cancer Vaccine Clinical Trial Working Group (CVCTWG) established important parameters that served as a base to Food and Drug Administration (FDA) guidance to conduct clinical experiments [15,16]. Comprehensive assessment, including parameters like immune response, overall survival, and quality of life, are essential to translate the therapeutic potential of therapeutic vaccines into clinically meaningful benefits.

Considering the epidemiological and clinical significance of hematological tumors, along with the urgent need for advancements in therapeutic approaches, the development of therapeutic vaccines represents a transformative step in treatment modalities. By thoroughly investigating the biological intricacies of these malignancies and tailoring therapeutic strategies accordingly, these vaccines present a promising avenue for enhancing patient outcomes. Therefore, it is imperative to evaluate the current landscape of vaccine development to identify existing gaps and opportunities in this field. This scoping review aims to provide a comprehensive overview of the progress made over the past decade regarding therapeutic vaccines for hematological malignancies. To guide our exploration, we pose several critical questions: Which hematological malignancies are primarily targeted? What specific therapeutic targets are being pursued? What strategies and preclinical models have been implemented for validation? Furthermore, what significant results have emerged from these efforts? Addressing these questions will not only illuminate the current status of vaccine research but also inform future directions for immunotherapeutic innovations in this domain.

## 2. Materials and Methods

**Figure 1:**
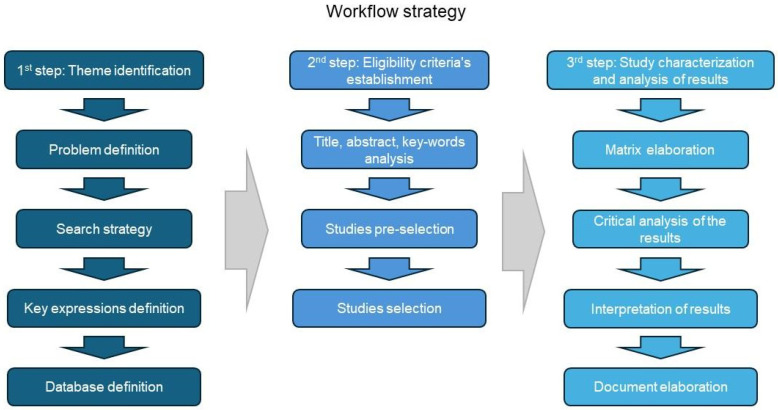
Workflow strategy for review construction.

To achieve the objective of this study, answering the main guideline questions, we have performed a scoping review in accordance with the PRISMA extension for scoping reviews (PRISMA-ScR) guidelines [17]. Figure 1 illustrates the workflow strategy employed to assess the current landscape of therapeutic vaccine development for hematological cancers. In the initial phase of preparing this review, we defined the search strategy, which involved selecting relevant databases and establishing eligibility criteria that included both inclusion and exclusion parameters. This research aimed to elucidate the preclinical and clinical development stages of immunotherapeutic vaccines for treating hematological cancers. Specifically, we concentrated on original publications that presented experimental studies. The final phase entailed retrieving and summarizing the data, results, and conclusions from the selected studies.

This review included articles published in English between 1 January 2013, and 30 March 2024. We selected the Scopus, PubMed, Web of Science, and ScienceDirect databases, which encompass the fields of biology, immunology, and oncology. Our search strategy involved querying combinations of the following key expressions in the title, abstract, or keywords of the articles: (“Immunotherapy”) AND ((“Vaccines” OR “Immunization”) AND (“Hematological Malignancies” OR “Blood Cancer” OR “Hematopoietic Neoplasms” OR “Leukemia”)). The filters applied were as follows:

➢ Pubmed: Free Full text, Full Text, 10 years;
➢ Science Direct: Title, abstract or author-specified keywords, 10 years;
➢ Scopus: Title, abstract or author-specified keywords, 10 years;
➢ Web of Science: Open Access, Article, English, 10 years.

The included studies comprised qualitative and/or quantitative analyses in either preclinical or clinical phases, specifically full articles that were open access and published in English. Publications relying solely on computational or in vitro approaches, as well as review articles, were deliberately excluded. The lists of excluded articles are provided in Supplementary Materials. After compiling the list of all articles from databases, we utilized the online tool available on new.rayyan.ai (1 Broadway, 14th Floor Cambridge, MA 02142 USA) to eliminate duplicates. Subsequently, we conducted a thorough screening of the titles and abstracts to identify publications genuinely related to vaccines for hematological cancers. Any publications lacking these essential aspects were excluded during this screening process. In the final selection step, we excluded publications that focused solely on (1) computational or in vitro approaches; (2) CAR-T or other cell adoptive transfer studies; and (3) immunotherapy without a clear protocol for therapeutic vaccination. To facilitate analysis, we created a matrix to synthesize the most relevant information such as the disease, study stage, antigen selection, vaccine composition and therapeutic scheme, assays performed, and vaccine efficacy aiming to answer the guiding questions described before.

## 3. Results and Discussion

The number of articles from the four databases were 1396, 866 from Web of Science, 221 from Pubmed, 294 from Scopus, and 15 from Science Direct. After removing 217 duplicates, 1056 publications that did not focus on hematological cancer studies were excluded. Subsequently, we screened material and methods, results, discussion, and conclusion topics and excluded articles classified as (1) in vitro/in silico studies (n = 34), (2) CAR-T/adoptive cell transference studies (n = 23), and (3) immunotherapy without vaccine development (n = 11).

**Figure 2:**
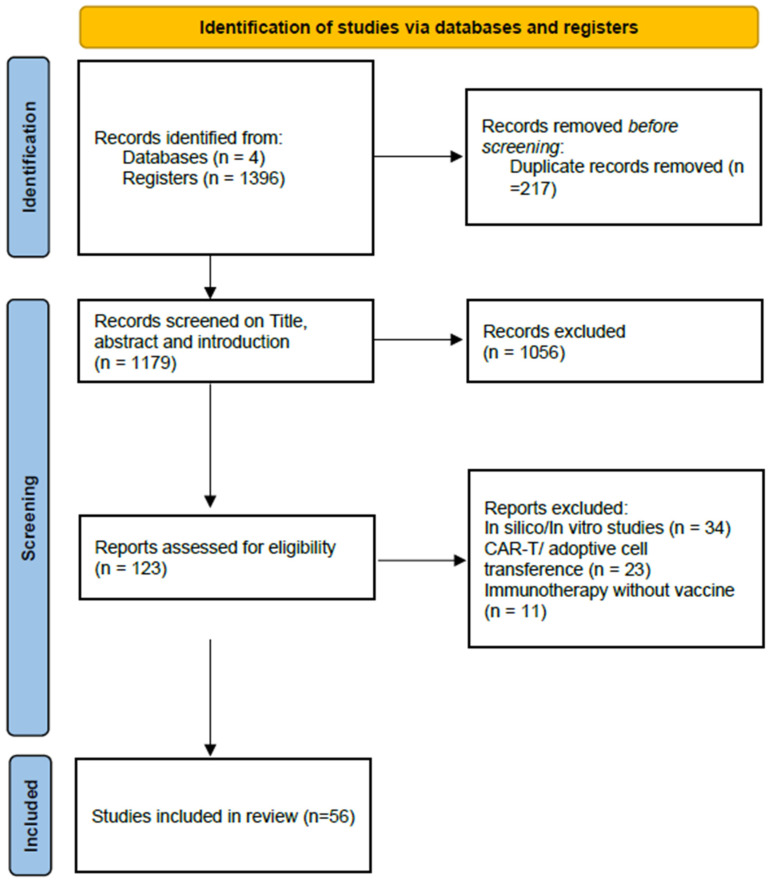
PRISMA flow diagram.

Ultimately, 56 remaining articles were included in this systematic review (Figure 2). The data were organized to feature the most relevant information in these publications, aiming to show the state of the art of the development of immunotherapeutic vaccines for hematological cancers. In these publications, we looked for (1) pathology and study development stage; (2) vaccine formulation, therapeutics approaches, and study follow up; (3) assays employed for study validation and treatment outcomes; and (4) study limitations. All these topics are covered in this review. The publications are chronologically organized in Table 1.

### 3.1. Pathology

**Figure 3:**
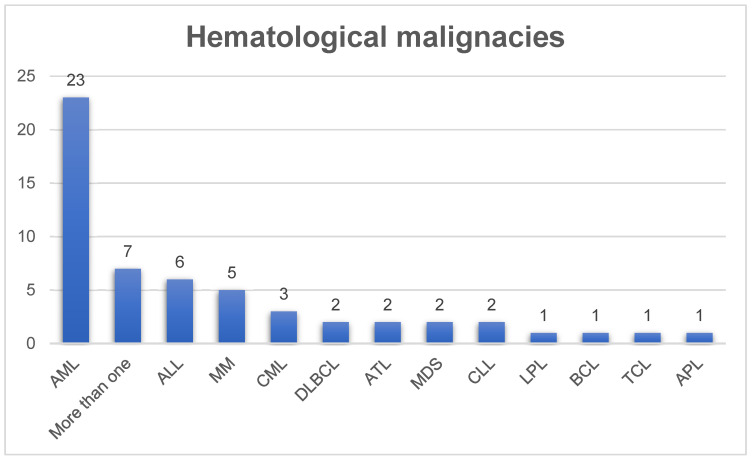
Distribution of distinct hematological malignancies in the study. Acute myeloid leukemia (AML), acute promyelocytic leukemia (APL), acute lymphocytic leukemia (ALL), chronic myeloid leukemia (CML), chronic lymphocytic leukemia (CLL), multiple myeloma (MM), myelodysplastic syndrome (MDS), adult T-cell lymphoma (ATL), T-cell lymphoma (TCL), B-cell lymphoma (BCL), diffuse large B-cell lymphomas (DLBCL), and lymphoblastic lymphoma (LPL).

This paper aims to assess the developmental stage of immunotherapeutic vaccines as a potential treatment tool for hematological cancers. Within the broad spectrum of hematological cancers, our study specifically addresses the following pathologies: acute myeloid leukemia (AML) [18,20,29,30,31,33,34,37,39,42,43,44,45,46,47,51,53,54,56,59,61,65,67,69,70,71,72], acute promyelocytic leukemia (APL) [25,56], acute lymphocytic leukemia (ALL) [22,28,30,32,51,54,72], chronic myeloid leukemia (CML) [59,62,67], chronic lymphocytic leukemia (CLL) [61,65], multiple myeloma (MM) [21,24,35,41,58], myelodysplastic syndrome (MDS) [25,39,40,48], Hodgkin’s lymphoma (HL) and non-Hodgkin’s lymphoma (NHL) subtypes such as adult T-cell lymphoma (ATL) [23,27,30], T-cell lymphoma (TCL) [19], B-cell lymphoma (BCL) [26,66], diffuse large B-cell lymphomas (DLBCL) [36,38,47], lymphoblastic lymphoma (LPL) [49], mantle cell lymphoma (MCL) [36], and cutaneous T-cell lymphoma (CTCL) [63]. It is worth noting that some publications covered more than one pathology, as shown in Figure 3.

As we observed in our review, the larger number of studies focused on new immunotherapeutic vaccines are on leukemias, mainly AML. Probably this correlation is in accordance with the risk of mortality data. According to data collected from the International Agency for Research on Cancer (WHO) in 2022, NHL in general represents 42% of total cases of hematological cancers, while leukemias represent 37%, MM 14%, and HL 6%. On the other hand, when we look at the mortality numbers, leukemias represent 44%, followed by NHL with 36%, MM with 17%, and HL with 3% of records.

Leukemias affect mainly the bone marrow and the blood and can be classified in acute and chronic forms, involving both myeloid and lymphoid cells [74]. AML is a bone marrow disorder, characterized by genetic alterations in hematopoietic stem cells, leading to an overproduction of neoplastic clonal myeloid stem cells. This proliferation impairs the normal production of red blood cells, platelets, and non-B/non-T white blood cells [75]. A distinct type of AML, APL is defined by the PML-RARA rearrangement due to the t(15;17)(q24;q21) translocation. This translocation, associated with secondary cooperating events, contributes to APL pathogenesis marked tendency towards coagulopathy, hemorrhage, and early death [76,77]. ALL is marked by the rapid growth and accumulation of malignant immature lymphoid cells in the bone marrow, which can also extend to extramedullary locations. The disease is categorized into B-lineage and T-lineage types [78,79]. CML is a clonal myeloproliferative neoplasm of hematopoietic stem cells, driven by the BCR-ABL1 oncoprotein, which leads to uncontrolled proliferation of myeloid cells at various stages of differentiation [80,81]. CLL is a lymphoproliferative disease characterized by the clonal accumulation and accumulation of mature, typically CD5-positive B-cells within the blood, bone marrow, lymph nodes, and spleen, which affect both the innate and adaptive arms of the immune response and accumulate during disease progression [82,83].

MM is characterized by the presence of terminally differentiated malignant plasma cells infiltrated in the bone marrow and the elevated secretion of monoclonal immunoglobulin protein [84,85], heterogeneous group of clonal hematopoietic stem cell disorders, featuring ineffective hematopoiesis, cytopenia, and dysplasia in myeloid cells, with a high risk of progression to AML [86,87].

Lymphomas represent a diverse group of malignancies arising from the clonal proliferation of B-cells, T-cells, and natural killer (NK) cells at various stages of maturation, primarily affecting the lymphatic system and lymph nodes. They are broadly categorized into non-Hodgkin (NHL) and Hodgkin (HL) types [88,89]. HL is characterized by the presence of a few giant multinucleated Hodgkin and Reed–Sternberg cells surrounded by numerous dysfunctional immune cells [90,91]. NHL is the most common hematologic malignancy in the world, comprising a heterogeneous group of diseases that derive from malignant lymphocytes and their precursors that accumulate in the lymph nodes, extending to other organs [92,93]. ATL is a distinct mature peripheral T-cell malignancy of Treg/Th2 phenotype caused by human T-cell leukemia/lymphotropic virus type I (HTLV-1), endemic in some areas in the world mature [94,95]. TCL is a rare subtype of non-Hodgkin lymphoma (less than 10%) originating from mature T-cells that tend to be more aggressive than B-cell lymphomas [96]. BCL encompasses a group of B-cell neoplasms characterized by an abnormal proliferation of lymphoid cells at various stages of differentiation in lymphoid tissues and extra nodal territories [97,98]. DLBCL comprises a genetically varied collection of aggressive B-cell neoplasms, all sharing the hallmark of a diffuse growth of large, transformed B cells, exhibiting significant clinical, biological, and pathological diversity [99,100]. LPL is a slow-going low-grade B cell lymphoproliferative neoplasm characterized by small lymphocytes and monoclonal IgM monoclonal gammopathy with bone marrow infiltration [101]. MCL is an uncommon B-cell lymphoma that typically exhibits expression of the T-cell-associated antigen CD5 and often is a progressive and incurable disease by conventional chemotherapy [99]. CTCL is a subtype of TCL characterized by malignant T-cell proliferation within inflamed skin lesions [102,103].

### 3.2. Vaccine Formulation, Therapeutic Approaches, and Study Follow-Up

Historically, vaccine development has been divided into three distinct generations. The first generation is characterized by whole antigens delivered through weakened or inactivated vaccines. The second generation employs purified subunits, proteins, or antigens with immunogenic potential, significantly reducing the risk of infection and disease development. The third vaccine generation comprises DNA plasmids capable of utilizing the host cell machinery to produce the desired antigen [104,105]. Following the COVID-19 pandemic, a common and directed effort of the scientific community advanced with the development of mRNA vaccines, called fourth-generation vaccines, which also use the host cell machinery to produce the antigen and induce innate and adaptive immunity [106]. Another category of vaccines, although not fitting neatly into the generational classification mentioned, is dendritic cell (DC) vaccines, which have become a cornerstone in immunotherapeutic approaches, especially for cancer treatments [107]. The use of these tumor antigens associated with autologous DCs in the vaccination enhances the activation of a broad repertoire of tumor-cell-specific T cells [29,61] and increases the chance of a good immune system activation due to the natural capability of these cells for good antigen presentation, and by being the own patient’s cells, they reduce the chance of rejection.

In cancer research, all types of vaccines are represented, with a wide heterogeneity of formulations and strategies to achieve the main goal: activate the immune system to eliminate specific cells. Our review identified, for hematological cancers, studies exploring first-generation, second-generation, third-generation, fourth-generation, and for DCs vaccines (Table 2), where each one has its advantages and disadvantages [108,109,110,111].

Regarding antigen sources, vaccines derived from cell lysates [26,34,36,38,39,44,45,46,49,52,56,59,61,63], peptides [20,21,23,30,33,35,40,41,43,47,53,57,58,62,66,69], and DNA [18,24,25,50,60] were reported. Additionally, we observed vaccines based on bacteria or viruses expressing specific peptides [27,28,65,67,70,71], as well as exosome and shRNA/mRNA vaccines [22,29,31,32,37,48,51,54,55,64,68,72]. This diversity in antigen sources underscores the flexibility and adaptability of therapeutic vaccine development for hematologic cancers.

Although most studies in our review employed whole-cell antigens or synthetic peptides, each antigen source offers distinct immunogenic properties and challenges. For instance, cell lysates provide a broad spectrum of tumor-associated antigens, while peptide-based vaccines enable precise targeting of specific epitopes. DNA vaccines allow for sustained antigen expression, whereas mRNA vaccines utilize the host cell machinery to produce specific antigens. Dendritic cell (DC) vaccines are designed to enhance antigen presentation, promoting robust, adaptive immune responses that may improve specificity and immune memory.

However, each antigen source also presents limitations. First-generation vaccines may be unsuitable for immunocompromised patients, while second-generation vaccines often exhibit low immunogenicity. Third-generation vaccines, such as DNA-based approaches, can be costly to produce, and fourth-generation RNA-based vaccines face stability challenges due to the inherent degradability of RNA. DC vaccines, by their intrinsic nature, may encounter compatibility issues with MHC matching, and they require isolation and culture, which can impact their migratory capacity.

A nuanced understanding of these characteristics is essential for balancing immunogenicity, safety, and practical applicability. Achieving this balance is crucial for advancing vaccine efficacy and fostering personalized cancer treatment approaches.

By definition, an antigen is the immunogenic fraction that will be responsible by immune cell activation and differentiation in specific cells that will recognize in the malignant cells the same structure that was activated. In the development of therapeutic cancer vaccines, two primary classifications of antigens are commonly employed: tumor-associated antigens (TAAs) and tumor-specific antigens (TSAs). TAA [18,19,20,21,22,25,26,29,30,31,32,33,34,35,36,37,38,39,40,41,42,43,44,45,46,47,48,49,51,52,53,54,55,56,57,58,59,60,61,63,64,65,66,67,68,69,70,71,72,73] are antigens that, while present in non-tumor cells, exhibit significantly higher expression in cancerous cells, thereby allowing for selective immune targeting. Conversely, TSAs [23,24,27,28,50,62] are exclusive to tumor cells, enhancing the specificity of immune recognition and reducing the risk of off-target effects.

Among the TAAs, Wilms Tumor 1 (WT1) protein, a common renal tumor overexpressed in many hematological malignancies such as AML, ALL, MDS, CML, stands out as a promising candidate. It is present in 16 selected publications in our review [20,29,30,33,37,40,42,43,47,54,60,65,69,70,71,72], in accordance with the National Cancer Institute consensus study prioritizing it as a top immunotherapy target [43,112,113].

The choice of therapeutic vaccine formulations and antigen sources aims to elicit a targeted immune response by engaging crucial immune cells in controlling disease progression. In the field of cancer immunotherapy, it is well established that T CD8^+^ cells (cytotoxic T lymphocytes, CTLs) have a central role in antigen recognition and destruction of mutated cells. These cells are critical for the success of various immunotherapeutic strategies [114,115]. Conversely, the role of T CD4^+^ T cells in cancer immunotherapy is becoming increasingly elucidated. T CD4^+^ cells have the ability to induce a potent microenvironment to help against tumoral cells [116] with cytokine production and cell activation. Recently, Kruse and colleagues in 2023 demonstrated the potential of CD4^+^ effector T cells to eradicate MHC-deficient tumor cells by inducing inflammatory cell death [117]. This finding highlights their ability to function independently of CD8^+^ T cells, which cannot target MHC-deficient cells [118]. The articles included in this review frequently assess the antigen recognition/activation, cytokine production by these cells and their ability to control tumor cells and disease progression.

The tumor microenvironment possesses the capacity to suppress the immune system cells. In this case, the antigen alone may not be enough, necessitating the inclusion of adjuvants alongside antigens to enhance cell activation, recruitment, and cytokine production. In general, an adjuvant is defined as a compound that boosts the immune response by acting on innate immunity, thereby facilitating a more effective activation of both innate and adaptive immune systems. Various classes of adjuvants have been identified, including plant vegetal extracts, bacteria fragments, emulsions able to produce a depot system, cytokines, and more recently developed compounds such as nanoparticle vehicles, oligonucleotides, double-stranded RNA molecules (dsRNA), etc. [119,120,121]. These compounds, when with antigens, should induce a stronger, faster, and longer-lasting immune response compared to the antigen alone [104]. However, only a limited number of adjuvants or adjuvant systems have been licensed for human use (Alum, MF59, AS01, AS03, AS04, CpG 1018, Matrix M*), with others currently undergoing phase 1 and 2 clinical trials [105,121,122,123].

Our review indicates that numerous therapeutic vaccine formulations targeting hematological tumors incorporate adjuvants to enhance the immunogenicity of antigens that naturally are low immunogenic. These include proteins or cytokines/growth factors [30,34,36,49,50,63], Toll-like receptors (TLRs) [38,51,53,54,56,59,69], emulsions [20,21,33,40,42,43,47,58,66], lipids [26,45,65,72] or bacteria [70,71] The choice of use or not of an adjuvant and what class of adjuvant use is totally dependent on the antigen source (e.g., peptide or chimeric protein) and the kind of immune response desired.

In the context of developing therapeutic vaccines against hematological tumors, the selection of the appropriate immunization route and scheme is crucial for the vaccine’s success [106,124]. In the studies included in this review, various immunization routes were employed, including the intradermal route (ID) [27,29,30,31,37,39,40,44,49,50,54,60,62,63,68], intramuscular route (IM) [18,24,25,41,51,69], intraperitoneal route (IP) [28,36], intravenous route (IV) [19,26,28,48,53,55,64,65,72,73], subcutaneous route (SC) [20,21,22,23,30,32,33,34,35,38,39,42,43,46,47,51,52,55,56,57,58,59,61,67], and oral route (O) [69,70]. Each immunization route is associated with distinct mechanisms of immune activation and can influence the magnitude and quality of the immune response. For instance, the ID or SC routes are often associated with more efficient activation of DCs compared to other routes [125].

As mentioned above, it is well known that the activation of the immune system by professional antigen presenting cells (APCs) like DCs increases the success rate of the treatment. Due to their highly specialized nature, DCs have a unique capacity to establish and regulate primary immune responses [107,126]. Immature DCs are present in the peripheral tissues, and upon antigen recognition, they start the migration to peripheral lymphoid organs and undergo maturation [113,127,128]. It is no surprise that many of the articles (Table 2) in our review use dendritic cells as a tool for efficient vaccination.

The choice of the number of immunizations in therapeutic vaccine protocols is a critical factor that can influence the strength, duration, and quality of the immune response, enhancing the immune memory and sustaining the high levels of effector cells needed to combat residual cancer cells effectively. In our review, we observed that therapeutic vaccines currently under development for hematological cancers exhibit a wide variety of dosing regimens. While some studies administer only one dose, others utilize two, three, four, even up to six doses or more. Dosing schedules can vary significantly, with intervals ranging from one day, one or two weeks between doses to more extended schedules, such as one dose monthly or every six months.

The choice of number of interventions depends on various factors, including the stage of study development (preclinical or clinical), the specific tumor type, and the stage of disease.

In preclinical studies, the number of doses is typically limited to a range of one to four. However, some studies employed six doses [27], ten doses [69,70], and fourteen doses [68]. For instance, phase 1 clinical studies can employ dose escalation designs to identify the maximum tolerated dose without causing adverse events. In phase 2 studies, the immunization schemes are more variable regarding the interval between doses and boosters, becoming more spaced out when the patient goes into remission or does not have a diagnosis of minimal residual disease (MRD).

In our review, we examined 14 publications that explored the inclusion of associated treatments with therapeutic vaccines to enhance disease prognosis in hematological cancers. Most of these studies utilized monoclonal antibodies (mAB) as checkpoint inhibitors [26,28,45,53,61] and employed cytokines and/or growth factors as cell microenvironment stimulators [34,36,43,63,72]. Additionally, we noted studies that investigated the inoculation of PBMC/leukocytes as associated treatment [30,39], as well as the use of vitamin derivatives or lipids/glycolipids [25,36,38].

In the last ten years, mAB therapies against specific cell targets like CTLA-4 and PD-L1/PD-1 have been safe and effective against hematologic malignancies [129], reinforcing the role of checkpoint inhibitors in disease progression. In addition, the use of cytokines such as IFN, IL-2, IL-12, and IL-21 as associated treatments is an important strategy due to their capability to regulate both the innate and adaptive immune response, allowing the communication between these cells and improving the cancer cell recognition and destruction [130]. Another associated strategy is the cell transplant that aims to improve the vaccine antigen recognition increasing availability of immune cells by direct inoculation of donor lymphocytes or by HSC transplantation [131].

The combination of therapies is an important strategy, especially due to its ability to subvert the modulated tumor immune microenvironment. This approach also has the potential to overcome challenges associated with conventional therapies, such as resistance mechanisms, thereby offering new opportunities to enhance treatment efficacy and improve patient outcomes [132]. For immunotherapeutic vaccines, which rely on the patient’s cellular response to achieve success, the use of these associated treatments is particularly beneficial.

In preclinical studies, the associated treatments primarily aimed to improve the immune response mechanisms of therapeutic vaccines by enhancing cell recruitment/activation or avoiding immune escape mechanisms triggered by cancer cells. Conversely, in clinical studies, the focus of these treatments is on assisting patients in achieving cancer remission and clearance of MRD, and the associated treatments employed were cell inoculation and cytokine for cell stimulation.

Another important aspect to the study of immunotherapeutic vaccines is the establishment of disease in preclinical models. In the context of disease challenge, this is a strategy to induce tumor development in the animal. In our review, we noticed that the main routes utilized for tumor cell inoculation are IV [19,25,26,28,34,45,46,53,57,59,61,68,72,73], IP [27,38,62], SC [21,22,32,35,36,41,42,48,52,53,55,56,60,64,70,71], and one study had IV and SC routes [66]. It is important to highlight that route choice will directly impact the tumor development and disease mimicking. On the natural evolution of hematological cancers, often there is no solid tumor development. However, in preclinical models, depending on study objectives and route for challenge such as SC injection, a growth of solid mass of malignant cells is observed. In these studies, the primary aim is often to test the efficacy of vaccines in reducing tumor growth by the diameter measure or tumor mass weight and evaluating the cell-infiltrating microenvironment within these tissues, such as the presence of DCs and T-cells or cytokines production.

### 3.3. Study Stage Development

**Figure 4:**
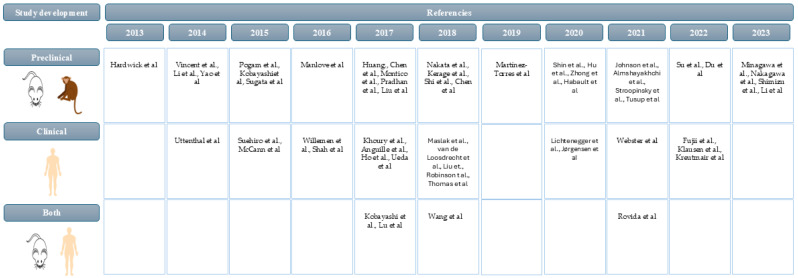
Categorical distribution of trials within a 10-year time window [18,19,20,21,23,24,25,26,27,28,29,30,31,32,33,34,35,36,37,38,39,40,41,42,43,44,45,46,47,48,49,50,51,52,53,54,55,56,57,58,59,60,61,62,63,64,65,66,67,68,69,70,71,72,73].

The development stages of therapeutic vaccines typically span preclinical and clinical phases (1, 2, 3, and 4). However, we observed that in our review, studies were concentrated in preclinical, phase 1, and phase 2 trials, with some addressing more than one stage of development. Specifically, the studies were categorized as follows: preclinical [18,19,21,22,25,26,27,28,32,34,36,38,41,42,45,46,48,52,53,55,56,57,59,60,61,64,67,68,70,71,72,73], preclinical and phase 1 [33,35,62], phase 1 [23,24,29,39,44,47,49,50,54,58,65,69], phase 2 [31,37,43,63], phase 1 and 2 [20,30,40,66], and all stages [51]. Figure 4 illustrates the categorical distribution of stage trials over the past decade.

It is important to point out that the focus of the studies has an intrinsic relationship with the model and the stage of development. Preclinical studies were the majority in our review and predominantly aimed to elucidate the immune response triggered by the vaccines, investigating parameters such as cytokine and chemokine production, cell recruitment and activation, tumor cell lysis, and tumor regression or clearance. The most commonly used models in these preclinical studies were transgenic mice [18,19,21,25,26,27,28,35,36,48,57,60,62,73] and non-transgenic mice [22,26,27,28,32,34,38,41,42,45,46,48,52,53,55,56,59,61,62,65,67,68,70,71,72], with some studies utilizing monkeys [27,51] to mimic human disease or mouse-specific disease. These preclinical studies focused primarily on evaluating vaccine performance in controlling cancer progression or providing protection against future relapses. Most of the research examined the role of the immune system in controlling tumor cells, with further details on specific strategies explored in a subsequent section.

The selection of the appropriate animal model is essential for evaluating cancer vaccines, as only experimental models with a fully functional immune system can accurately replicate human responses to vaccination and predict the clinical outcomes of these potential anticancer strategies [112].

Although mouse models are widely used and have the best cost-effective preclinical tools [5], concerns remain regarding their representativeness of human biology, dividing opinions among researchers [114,115]. The fact is that a considerable number of preclinical candidates fail to perform similarly in clinical trials, highlighting the limitations of these models. To address this issue, for this reason, an alternative is humanized mice models such as xenograft models bearing characteristics of the human immune system and human tumors [10,116]. However, the use of transgenic models also has their own limitations. The development of the disease in mouse preclinical models does not always correspond to the natural course of the disease in patients, which leads to some discrepancies between studies.

Non-human primate (NHP) models share many genetic, physiological, immune cell, and immunological mechanisms similarities with humans, having the potential to overcome the limitations of other preclinical models [118]. They are highly important animal models to study complex human diseases because they provide a better understanding of biological functions or even to study the safety of new therapies [112].

It is important to point out that the successful transition to the clinical is totally dependent on a meticulous preclinical evaluation of several parameters such as pharmacokinetic, pharmacodynamic, metabolism, efficacy, and safety [10].

In clinical studies, the eligibility criteria, patient’s profiles, and disease stage are plural. However, 100% of studies of our review are conducted in patients already diagnosed with cancer and who are in some disease stage. The approaches vary from patients who have already undergone chemotherapy [20,24,29,31,33,39,43,44,47,54,58,63,65,69], patients who received allogeneic hematopoietic stem cell transfer (allo-HSCT) [24,30,40], and patients non-responsive to any therapy [39,40,62,65]. The primary focus of these studies is to evaluate the potential of cancer vaccines to improve prognosis and overall survival.

Notwithstanding, many guidelines provided by the World Health Organization (WHO) and regulatory agencies such as FDA and Centers for Disease Control and Prevention (CDC) offer a comprehensive framework for vaccine development [113,114]. These guidelines cover essential components such as FDA-approved vaccine ingredients, proof of concept, clinical trial phases (1, 2, 3, and 4), the vaccine manufacturing process, and the criteria for vaccine approval. Our review shows that the immunotherapeutic vaccine studies that are most advanced in phase 2 are for AML, followed by ALL, CML, CLL and HL.

In summary, these articles emphasize the necessity of advances in the area of cancer immunotherapy using vaccines as important tools as alternative treatment. Given the diversity of hematologic cancers and the range of disease stages, there is a clear necessity for novel approaches and strategies to improve patient outcomes and quality of life. While there remains a substantial gap between preclinical and clinical stages, both are indispensable. The molecular, cellular, and immunological responses observed in preclinical studies provide the necessary foundation for translating these findings into clinical trials, where successful performance is crucial.

### 3.4. Assays and Techniques Employed to Vaccine Evaluation

Assessing the efficacy of therapeutic vaccines in hematological cancers requires a comprehensive set of assays to evaluate both the immunologic response and clinical outcomes. These assessments range from cellular and molecular analyses that track immune activation and tumor-specific responses to clinical evaluations that determine how effectively these responses translate into patient benefits. Selection of the appropriate assays and outcome measures is essential to accurately gauge vaccine success and refine strategies that could improve patient prognosis.

In our review, we identified a variety of tools available to assess the efficacy of therapeutic vaccine treatments for hematological cancers. However, not all studies are equipped to utilize these techniques, and the choice of study model influences the types of questions that can be effectively addressed. While the goal is to advance cancer treatment with a focus on patient outcomes, preclinical approaches offer valuable insights into the understanding of the cellular, molecular, and immunologic mechanisms at play.

#### 3.4.1. Preclinical Studies Overview

Among the most employed assays at preclinical are serological assays, particularly the enzyme-linked immunosorbent assay (ELISA), which is widely used for detecting antibodies and/or cytokines [18,25,26,27,33,34,35,36,38,41,46,51,55,56,57,62,64,67,68,71]. These assays facilitate the analysis of specific immunoglobulins such as IgG and pro or anti-inflammatory cytokines, chemokines, growth factors that are essential for establishing a protective immune response.

Histological assays [25,41,42,51,71] are also prevalent, with techniques such as hematoxylin-eosin (HE), immunohistochemistry (IHC), or blood or bone marrow smear tests being utilized to assess the presence of immature cells, identifying specific cell populations or even analyzing tissue/organ structure. These methodologies enable a comprehensive diagnostic approach, ranging from tumor cell identification to the assessment of immune cell populations within tissue microenvironments.

In terms of cellular immunity, flow cytometry (FC) [18,19,21,22,25,26,27,28,32,33,34,35,36,38,41,42,45,46,48,51,52,53,55,56,57,59,60,61,62,64,67,68,70,71,72,73], enzyme-linked immunosorbent spot (ELISPOT) [18,19,28,36,46,51,53,59,60,62,68,72], and multiplex assays [72] are important tools for elucidating the immunophenotype of cells populations in the peripheral blood mononuclear cells (PBMC), bone marrow mononuclear cells (BMMC), or specific organ cells, such as splenocytes These techniques enable various assessments, including intracellular staining, proliferation assays, cytokine production, tetramer assay for antigen cell recognition, and apoptosis assay. Collectively, these methodologies enhance our understanding of the immunological stats of individuals or animals undergoing treatment with the immunotherapeutic vaccine.

In addition to the previously mentioned assays, several other ex vivo assays are commonly employed to enhance our understanding of therapeutic vaccines for hematological cancers [18,19,21,22,26,27,28,32,33,34,35,36,38,41,46,48,50,51,52,53,55,56,57,59,60,61,62,64,67,68,71,73]. These include cytolytic assay, such as cell-killing assays or CR51 assay, as well as cell migration assays, cell differentiation assays, and antigen presentation assays.

Biochemical analyses were reported [73] by measuring albumin, blood creatinine, and urea levels, as well as alanine transaminase (ALT) and aspartate aminotransferase (AST), which show important parameters of clinical status of hepatic or renal functions.

Imaging techniques [22,32,34,35,38,45,48,55,59,60,68], including confocal microscopy and IVIS spectrum in vivo imaging system have been utilized to evaluate cell behavior and tumor progression, as well as to monitor the survival rate of these animals after the disease challenge. The advantage of follow-up to the disease progression or regression in vivo is that it is a powerful tool to validate the vaccine efficacy. For structural analysis of cells/vehicles or tissues, techniques such as electronic microscopy, micro-computed tomography analysis, and nanoparticle tracking analysis have been employed. Knowing if the vaccine compounds are intact, viable, and functional is important to guarantee the quality of production of the vaccine.

Moreover, analytical chemistry assays [33] of vaccine compounds were performed by high-performance liquid chromatography (HPLC). These studies reinforce the importance of evaluating the integrity and the quality of the micro/nanostructures where the antigens are loaded to ensure better vaccine stability and, consequently, the better immune system activation.

#### 3.4.2. Clinical Studies Overview

In clinical-stage studies, the number of approaches that can be employed with patients and the facility to obtain a broad range of samples is limited when compared with preclinical models. However, it is important to highlight that it is in this stage that we really start to know how effective our vaccine is. The main focuses in phase 1 are safety and tolerance of vaccine administration, if there are adverse events after immunotherapeutic treatment, dose concentration analysis. On phase 2, the focus changes to gather some immune responses, usually with the observation of specific antibodies, delayed-type hypersensitivity (DTH) response to detect the presence of a specific cell-mediated immune response, PBMC evaluations, and the evaluation of molecular markers such as expression of WT1 and others related to MRD, disease remission or progression, and overall survival rate to establish a prognosis.

The clinical studies predominantly employed the following assays: ELISA [24,29,37,39,54,69] for the detection of cytokines and antibodies; histological assays [31,37,50,54] for smear analysis; flow cytometry [20,23,37,39,40,43,44,54,58,65,66,69] for immunophenotypic analysis; ELISPOT [20,24,30,31,43,47,50,54,58,65,66] for analyzing cytokines and cells populations; multiplex assays [49,58] for cytokines and chemokines analysis; and ex vivo assays [20,23,29,40,44,47,49,54,58,63,65,69] for cytotoxicity, cell activation, and antigen recognition analysis.

In the validation process of an immunotherapeutic vaccine, each assay serves a specific purpose, addresses a distinct question, and contributes to advancing our understanding of the immunological mechanisms triggered by the vaccine in response to the disease. The detection of specific proteins, such as specific antibodies like immunoglobulin g (IgG) or cytokines (e.g., IFN-γ, TNF, IL-12, IL-4), serves as to find important biomarkers. These biomarkers indicate that the vaccine successfully induced long lasting memory B cells or that treatment is inducing a significant proinflammatory microenvironment conducive to disease control [133]. Given the relevance of cytokine production induced by immunotherapeutic interventions, various assays are employed to detect these molecules. Among the most commonly utilized methods are those described above in this topic. They can recruit and activate cells that were being avoided by tumor cells as an escape mechanism or even to reverse cell exhaustion, a phenotype very common in cancer disease. Another essential indicator of successful vaccination is the cell differentiation triggered after immunization. It is a consensus that cellular responses have a main role in controlling disease advancement. T CD8^+^ cells are responsible for recognizing and killing the malignant cells through cytolytic action, T CD4^+^ cells are responsible for both the production of cytokines and the elimination of tumor cells. Natural killers, DCs, and other innate immune cells are responsible also for the creation of favorable microenvironments [134]. In addition to that, it is important that a vaccine be able to induce memory cell generation that ideally will prevent disease relapse [117]. Many of these evaluations are performed in the blood, bone marrow, or other tissue (e.g., spleen) samples. It is also possible to perform evaluations looking for the presence of immature blasts as an important biomarker for disease remission or progression. It has been reported that MRDs are a small group of persistent cells responsible for the disease return; in this sense, it is desired that an immunotherapeutic vaccine promotes the clearance even of these cells. To confirm that, some studies quantify by molecular techniques the expression levels of markers such as WT1 gene in circulating blood. A complementary analysis to confirm the immunotherapeutic success of vaccine administration is the follow-up of overall survival rate accompanied by (in murine models) detection of green fluorescence protein (GFP^+^) or luciferase markers in tumor cells, indicating the disease advance or remission [135,136].

### 3.5. Vaccine Treatment Outcomes

Regarding the treatment outcomes, there is a complexity to appraising the immunotherapeutic vaccine performance. In general, the main biomarkers evaluated are those that were discussed before, like tumor regression, profile of cytokines produced after immunization protocol (e.g., TNF, IFN, IL-12), the induction of memory cells (CD44^+^CD62L^+^), cytotoxic markers, molecular traces of MRD markers such as WT1 protein, presence of immature blasts in blood or bone marrow smears, and of course, the adverse events and toxicity triggered by the vaccines and the overall survival. The compilation of the factors like efficient antigen presentation, proinflammatory cytokine production, T cell recruitment and activation, memory cell induction, tumor cell regression or remission, a good tolerance of adverse effects, and an increment of overall survival are good indicators of vaccination success [42,123,137].

As we demonstrated in this review, there is a huge divergence of approaches between preclinical and clinical studies and, consequently, the results that can be accomplished. In the first one, the vaccine’s success or failure can be measured in different steps. As described above, ex vivo assays are performed and tissues and organs are exercised for a diversity of cellular response evaluations, molecular detection of disease markers, and tumor cell persistence analysis. In addition to that, it is easier to perform in vivo assays to test vaccine antigens, dose response, immunization vias, and a follow-up to establish survival rates in preclinical models. The number of samples and the possibilities of approaches available are infinitely more accessible. Vaccine candidates typically induce immune cell activation, promote tumor regression or clearance, and, in some cases, protect vaccinated animals against re-challenges, simulating disease relapse. These effects are often associated with an improvement in overall survival.

We observed that the vaccine candidates for the preclinical stage covered in this review were able to reduce the tumor size/volume, protect the animal from disease development, and improve the survival rate. The main features that led to these outcomes were that the vaccine induced a pro-inflammatory microenvironment labeled by TNF and IFN cytokine families [18,19,21,25,26,28,36,38,41,42,46,52,53,59,60,61,68,71,72,73]. The main cells were NK [32,38,45,68,72] and DC [22,32,36,42,55,56,71] cells from innate immunity, T CD4^+^ TCD8^+^ cells from adaptive immunity, and memory cells, specifically effector memory T cells [21,25,27,28,41,42,45,52,59,60,61,70,72]. The central mechanism triggered by the vaccines to fight against tumor cells was the increment of cytotoxicity by NK and CTLs [18,19,21,22,25,26,27,28,32,34,35,36,38,41,42,45,46,48,51,52,53,55,56,57,59,60,61,62,64,67,68,70,71,72,73]. In addition, the immunotherapeutic vaccines also focused on reducing Tregs numbers [34,35,61].

In clinical studies, the vaccine immunotherapy performance analysis is more complex. The treatment outcome usually comes attached to disease prognosis. The most common ex vivo assays are cellular investigations (PBMC or BMMC) profiles, serological analysis (specific IgG) or molecular analyses (MRD markers), and body rejection of the treatment and clinical evaluations. Regarding disease status, the classification of cancer staging may vary slightly from pathology to pathology; however, it always is observed if the treatment induced cancer remission or at least promoted a delayed progression, improving overall survival in comparison to the previous average.

It is important to highlight that in our review, we did not find any study in the clinical stage that observed complete cancer clearance in all patients. However, some studies showed a high percentage of patients that maintained the disease in cancer remission stage (CR) or, if not in CR, did not observe cancer progression during the study follow-up. Other studies showed that vaccine treatment delayed the disease progression. Few studies did not have success in improving overall survival.

For clinical stages, the features that we observed for vaccines were that formulations were safe with low grade side effects [20,23,24,29,30,31,33,35,37,39,43,44,47,49,50,51,54,58,62,63,65,66,69]. One study had high grade side effects [40]. Usually, the severity of adverse effects ranges from grade 1 to 5, with grades 1 and 2 representing the mildest and most tolerable effects, while grades 3 and above are considered more serious. Common signs evaluated include allergic reactions, hypersensitivity (including fever), autoimmune responses, vasculitis, WBC count variations, lymphopenia, myelodysplasia, platelet levels, splenic function, general cardiac performance, coagulation capacity, and constitutional symptoms such as fatigue, hypothermia, and insomnia, among other indicators. As observed in preclinical studies, here, the importance of cellular immunity triggered by vaccines and the cytokines produced were evaluated too [20,23,29,30,33,35,37,39,40,43,44,47,49,51,54,58,62,63,66,69] with a positive correlation and the presence of proinflammatory cytokines (e.g., IFN, TNF) and better treatment outcome. Moreover, DTH assays [30,31,37,39,40,54,58] were performed as a complement to cellular immune response evaluation, displaying a good response in the vaccine candidates who had an efficient cellular immune activation. Finally, the main feature evaluated was if the vaccinations were able to improve patient prognosis and survival rate [20,23,24,29,30,31,33,35,37,39,40,43,44,47,49,50,51,54,58,62,63,65,66,69].

In general, the studies reviewed suggest that leveraging the immune system to combat tumor cells is a promising approach in the treatment of hematological cancers. Preclinical findings have demonstrated that vaccine candidates can successfully induce a pro-inflammatory microenvironment, activate cytotoxic immune responses through NK cells and CTLs, and promote the recruitment and persistence of memory T cells, which are essential components for a sustained immune response. These early-stage studies also indicated that vaccine formulations could reduce tumor size, delay disease progression, and improve survival rates in animal models, providing a solid proof of concept for further clinical testing.

Although none of the clinical studies reviewed have progressed beyond phase 2, the promising results underscore the potential of this immunotherapeutic strategy. The methodologies applied, including flow cytometry, ELISA, ELISPOT, cytotoxicity assays, molecular analyses, and in vivo imaging, reinforce the feasibility and potential impact of these vaccines. Moving forward, late-stage clinical trials will be critical for assessing the durability of immune responses, long-term survival, and quality of life outcomes in patients. Collectively, these promising preclinical and early clinical findings establish a strong foundation for immunotherapeutic vaccines to become a viable standard of care in hematological oncology.

### 3.6. Study Limitations

In accordance with previous topics, each phase of study development presents limitations. Preclinical studies are essential for understanding numerous disease mechanisms and have significantly contributed to advancements in cancer treatment. However, the results obtained from these studies exhibit limitations when considering their applicability to human studies. This is attributed to the various intraspecific genetic characteristics of humans, as well as the differing genetic mutations among patients, which complicate the faithful replication of the disease in preclinical models.

In the preclinical stage, despite the many studies using transgenic mice and xenograft models [18,21,26,27,28,36,42,48,57], it is not possible to guarantee that the same treatment response will be observed in patients. In addition, these models need a high birth-control to keep their genetic characteristics and usually need a specific non-conventional infrastructure for the colony’s maintenance, a problem shared with the studies with NHP models [27,51]. Not everyone can conduct experiments with these models.

Another limitation that we found is that not every study has an extensive immunological evaluation of vaccine treatment. Some studies have limited assessments [18,22,25,57,67,73], maybe due to the lack of samples to perform all experiments, maybe due to the lack of subsidies to perform these techniques, of which some of them are expensive. The fact is that many studies choose to evaluate one or two immunological aspects to help better understand the acting mechanisms of the vaccine candidate, but still other techniques could be performed to give more substantive information.

Moreover, the induction of disease in animal models often lacks sufficient similarity to human conditions. The review indicates that many studies did not attempt to simulate the natural course of the disease by inoculating tumor cells via intraperitoneal or subcutaneous routes [21,22,27,32,35,36,38,41,42,48,52,53,55,56,60,62,64,67,70,71]. Although these approaches facilitate a better understanding of the interactions between tumor cells and immune cells at a controlled tumor site, the representation of naturally disseminated disease is consequently lost. Additionally, some malignancies like CML do not have a specific preclinical model [60,68] that is able to reproduce the disease in vivo. Furthermore, we noticed that two studies did not perform an in vivo challenge [18,19], which compromises the conclusion about the vaccine efficacy.

In the context of clinical studies, the diversity of diseases and the intrinsic variability among patients are prevalent. These studies provide insights into the performance of immunotherapeutic vaccines for the treatment of hematological diseases, specifically targeting the patient population. However, in addition to the restricted variety of approaches available for study, one of the main limitations is the often-limited number of individuals participating in clinical trials, ranging from three to ten patients, especially in phase 1 [23,54,58,69]. Another point is that some studies are beginning and do not have many conclusive data on the effect of treatment on disease progression in patients [20,30,44,47,49,50].

Unfortunately, in long-term follow-up studies, participant loss due to disease progression, death, or voluntary withdrawal is a common reality [20,23,24,29,30,31,37,39,40,43,44,47,49,50,54,58,63,65,66,69]. Regarding immunological assessments, in many studies of our review, the approach was limited [24,29,30,37,40,44,47,49,50,58]. Probably, as we observed, because of the restricted types of samples obtained, such as peripheral blood, PBMC, skin biopsies, and DTH assay.

Apart from the limitations observed in our review, it is widely recognized that the number of compounds available for testing in humans is constrained by the potential for severe adverse events. Even compounds already licensed for human use often provoke complications, which can vary based on the patient’s immune response and dosage levels. This constraint poses a significant barrier to the progression of clinical trials in this field.

These limitations underscore the inherent complexities and challenges in preclinical and clinical research on immunotherapeutic vaccines for hematological diseases. Addressing these challenges calls for innovative study designs and methodological advancements that can account for genetic variability, expand immunological assessments, and refine animal models to more closely simulate human disease. Continued efforts to optimize clinical trial structures and to develop safe, potent compounds will be essential for enhancing patient outcomes and advancing the field of immunotherapy for hematological malignancies.

### 3.7. Restrictions of the Scoping Review Process

Regarding the limitations of this scoping review, we restricted our analysis to publicly accessible articles written in English. Additionally, many studies focusing on immunotherapy approaches concentrate on treatments with CAR-T cells or checkpoint inhibitors, rather than on vaccine-based therapies. Immunotherapy as a field encompasses a wide range of possibilities, and not all vaccine studies included in vivo testing of vaccines, containing only in vitro analysis.

Another limitation was the lack of consistency in the therapeutic vaccine schemes presented. Not all studies clearly detailed essential information, such as dose, vaccine composition, number of immunizations, and intervals between doses, data that are crucial for this review and the articles that lacked this information were excluded.

## 4. Conclusions and Perspectives

It is evident that conventional treatments have limitations, and cancer cells exhibit a broad range of escape mechanisms that increase the likelihood of disease relapse. Hematopoietic stem cell transplantation (HSCT) has demonstrated the central role of the immune system in combating malignancies by targeting tumor cells. Within this context, vaccines have emerged as a viable immunotherapeutic strategy for the treatment of cancers, including hematological malignancies. Vaccines offer the distinct advantage of activating the immune system to recognize specific targets and elicit a tailored immune response.

In addressing our guiding questions, we observed that most studies focus on leukemias, particularly acute myeloid leukemia (AML), with significant attention also given to Hodgkin and non-Hodgkin lymphomas. Regarding therapeutic targets, tumor-associated antigens (TAA) are prioritized over tumor-specific antigens (TSA), likely due to guidelines from organizations such as the WHO, given that TAAs are shared across multiple malignancies, potentially broadening their applicability. For strategies and preclinical models, mice remain the predominant study model, though some advanced studies utilize non-human primates. Vaccine validation strategies are diverse and predominantly focus on therapeutic applications, with relatively few studies exploring the prophylactic potential of candidate vaccines.

Promising results have emerged from these studies, including a deeper understanding of the roles of immune cells in suppressing cancer—NK cells, DCs, CTLs, and Tregs have a major role in the balance of this response—as well as insights into the optimal cytokine environment with the production of IFN-γ, TNF, IL-6, and IL-12 to counteract tumor inhibition and identification of biomarkers for tracking disease progression or remission like expression of WT1. Additionally, there is growing evidence of the positive impact of vaccine formulations on patient quality of life.

Our review underscores the progress in immunotherapeutic vaccine development across various stages, incorporating multiple strategies with encouraging outcomes. Overall, vaccines have shown potential to induce a cell-mediated response with low adverse event risks for patients, contributing to a relative improvement in disease prognosis by directly targeting tumor cells. Both preclinical and clinical studies highlight the importance of a favorable immunocompetent microenvironment as essential for tumor regression. As research continues to advance, these vaccines hold promise for transforming the future landscape of hematological cancer treatments.

Perspectives include strategies to identify suitable targets using bioinformatics tools to accelerate this process and enhance accuracy. These approaches are increasingly prominent in cancer treatment studies, establishing themselves as fundamental steps in the field. One of the key advantages of bioinformatics is the possibility of identifying new epitopes or targets, either specific to individual patients or common across populations. Additionally, these targets can be ranked based on their immunogenic potential using algorithms specifically developed for this purpose, thereby reducing the time required and eliminating non-promising candidates.

Moreover, therapies that stimulate the tumor microenvironment by utilizing autologous patient cells to activate the immune system and reduce the risk of treatment rejection, such as dendritic cell (DC) vaccines, represent a promising direction for future advancements. Among novel vaccine approaches, mRNA vaccines stand out as an intriguing alternative, leveraging cellular machinery to produce desired targets. They offer advantages such as mass production capability, no risk of infection, relatively simple design, and the potential for personalized therapies, mainly in cases where the patient is refractory to treatment.

Finally, we believe that combining these therapeutic vaccine strategies with other immunotherapies—such as checkpoint inhibitors and cytokines—or even with traditional chemotherapies, is key to improving treatment success and disease prognosis. Despite the necessity of specific tumor targets in many situations, the development of vaccines targeting common-shared antigens (TAAs) remains highly desirable, aiming for an effective broad-spectrum approach.

## Figures and Tables

**Table 1:** Synthesis matrix of immunotherapeutic vaccine development for hematological cancers.

Ag	Vaccine Type	Pathology	Study Stage	Patients Profile	Disease Stage	Model	Challenge Via	Vaccine Scheme	Immunization Via	Associated Treatment	Follow Up	Assay	Efficacy	Limitations	Ref.
PASD1 antigen	DNA vaccine	AML	preclinical	N/A	N/A	HHD mice	N/A	1 dose	IM	N/A	N/A	(1) In silico-Bioinformatics; (2) Ex vivo; (3) ELISA; (4) ELISPOT; (5) Flow cytometry	PASD1-derived peptides binding to MHC-I; Stimulation of IFN-γ production by CD8^+^ T cells; Effector CD8^+^ T cells for the lysis of PASD1-expressing cells.	Limitations associated with the preclinical nature of the study; Requirement for transgenic animal models to enhance translational relevance; Absence of in vivo challenge experiments, restricting the assessment of protective efficacy; Limited scope of immunological evaluations to characterize the vaccine-induced response.	[18]
Peptides derived from leukemia associated Ags (LAAs) and minor histocompatibility Ags (miHAs)	DC vaccine	TCL	preclinical	N/A	N/A	B10, C57BL/10 and C57BL/6 mice	IV	2 doses, 1 week interval	IV	N/A	100 days	(1) Ex vivo; (2) In vitro; (3) ELISPOT; (4) Flow cytometry	Enhanced survival rate observed in experimental models; Increased frequency of antigen-specific T cells producing IFN-γ; Antigens with higher abundance did not consistently elicit the most robust immune response.	Limitations inherent to the preclinical nature of the study; Absence of in vivo challenge experiments, limiting the evaluation of real-world applicability.	[19]
Peptides derived from WT1 and pan-DR binding peptide epitope (PADRE)	Peptide vaccine	AML	clinical-phase 1 and 2	HLA-A*0201+ low risk patients	CR1, CR2, PR and slow progression	N/A	N/A	5 doses, 3 weeks interval	SC	N/A	41 months	(1) Safety and cytotoxicity; (2) Ex vivo; (3) Flow cytometry; (4) ELISPOT; (5) Molecular analyses	Safe formulations for administration in patients; Increase in specific CD8^+^ T cells up to 12 weeks; Increase in IFN-γ-producing CD8^+^ T cells in 6/7 patients; 2/5 eligible patients showed reduction in WT1 mRNA in PBMC.	Preliminary study with limited data available; Insufficient conclusive evidence regarding the treatment’s impact on disease progression in patients.	[20]
Synthetic HSP (heat-shock protein) peptides	Peptide vaccine	MM	preclinical	MM patients	N/A	HLA-A2.1-tg and NOD/SCID mice	SC	1 dose	SC	N/A	2 weeks	(1) In silico-Bioinformatics; (2) Ex vivo; (3) Molecular assays; (4) In vitro; (5) Flow cytometry; (6) Overall survival	MM cells show increased expression of HSPs; Increased proliferation of IFN-γ-producing CD8^+^ T cells; Increased lysis of MM cells by CD8^+^ T effector memory cells; Reduced tumor growth and increased survival rate.	Limitations inherent to the preclinical nature of the study; Necessity of employing transgenic animal models for further validation.	[21]
L1210 or K-562 Leukemic cell exosomes (LEXs)	DC vaccine	ALL	preclinical	N/A	N/A	DBA/2 mice	SC	1 dose	SC	N/A	70 days	(1) Imaging analysis; (2) Flow cytometry; (3) Ex vivo	LEXs are important antigenic sources; Their phagocytosis by DCs is efficient and stable even after 72 h; Increased cytotoxic capacity in animals; Prophylactic potential with increased resistance to disease establishment after vaccination; Increased survival rate.	Limitations due to the preclinical nature of the study; Limited evaluation of the immunological response to the vaccine.	[22]
Synthetic peptides Tax11-19 (LLFGYPVYV) and Tax301-309 (SFHSLHLLY)	DC vaccine	ATL	clinical-phase 1	HLA-A*0201, A*24:02 or A*11:01 patients	SD or PR	N/A	N/A	3 doses, 2 weeks interval	SC	N/A	14 months	(1) Ex vivo; (2) Safety and cytotoxicity; (3) Flow cytometry; (4) Molecular analysis	Safe vaccine formulation for administration in patients; Despite the fluctuation between patients, there was an increase in cytotoxic cells producing IFN-γ; Improvement in patient prognosis.	Small sample size; Limited conclusive data on the impact of treatment on disease progression in patients.	[23]
DNA encoding the patient-specific idiotype with the VH and VL region genes	DNA vaccine	MM	clinical-phase 1	MM patients who have already undergone chemotherapy and immunotherapy protocols	CR, PR, or SD	N/A	N/A	6 doses, 1 to 4 weeks interval	IM	N/A	52 weeks	(1) Molecular analyses; (2) ELISA; (3) ELISPOT; (4) Overall survival	Safe vaccine formulation for administration in patients; Increased production of specific IgG; 11/14 patients remained in remission after 52 weeks; 7/14 patients after 52 weeks remained without detection of disease biomarkers.	Limited immunological assessments of vaccine response.	[24]
Nonspecific immunogenic DNA sequences inserted into a vector	DNA vaccine	APL and MDS	preclinical	N/A	N/A	FVB/N mice	IV	3 doses, 20 days interval	IM	Vitamin-All-trans retinoic acic (ATRA)	200 days	(1) Molecular analyses; (2) Overall survival; (3) Histological analysis; (4) ELISA; (5) Flow cytometry	Increased survival rate; Reduction in the number of immature blasts in the bone marrow; The production of specific IgG for both vaccines was variable in the animals; Both the isolated vaccines and those associated with treatment showed an increase in memory T cells, cytotoxic T cells, and IFN-γ production.	Limitations arising from the preclinical nature of the study; Limited comprehensive immunological assessments of the vaccine response.	[25]
Eu-myc cell whole antigen	Cell lysate vaccine	BCL	preclinical	N/A	N/A	C57BL/6 and B6. SJLxOT-I e IFN-gKO mice	IV	1 dose	IV	mAB-anti-4-1BB	180 days	(1) Elisa; (2) Ex vivo; (3) Flow cytometry	The vaccine + antibody combination promoted the greatest increase in survival rate; The vaccine promoted long-lasting protection after a new challenge; An increase in cytotoxic T cells producing IFN-γ was observed; The importance of these cells in disease control was demonstrated by depletion and transfer assays.	Limitations due to the preclinical nature of the study; Requirement for the use of transgenic animals.	[26]
HBZ protein	Recombinant virus vaccine	ATL	preclinical	N/A	N/A	C57BL/6, NOD/SCID mice and Rhesus monkeys	IP	6 doses, 3–4 weeks interval	ID	N/A	45 days	(1) Flow cytometry; (2) Ex vivo; (3) ELISA	Vaccination induced increased production of pro-inflammatory cytokines by T cells; Immunization was protective with increased cytotoxic activity; The vaccine induced the formation of memory T cells.	Limitations due to this being a preclinical study; Need to utilize transgenic animals; Difficulty in employing non-human primates.	[27]
Bap (BCR-ABL) peptide	Peptide vaccine and Recombinant virus vaccine	ALL	preclinical	N/A	N/A	C57BL/6, Cdkn2a-/-, Foxp3-GFP, Ifng-/- and OT-I x Rag2-/-. mice	IV	3 doses, 7 days interval	IV or IP	mAB-anti-PDL1, anti-CTLA	80 days	(1) Flow cytometry; (2) Overall survival; (3) Ex vivo; (4) ELISPOT	Activation of CD4^+^ T cells by MHC-II molecules influences disease progression; The use of blocking antibodies promoted a modest increase in survival; Vaccination induced the formation of multifunctional cytokine-producing T cells and memory T cells; The cytokine IFN-γ is important in the anti-leukemic response.	Limitations due to this being a preclinical study; Need for the use transgenic animals.	[28]
mRNA RHAMM (hyaluronic acid)or mRNA WT1	DC vaccine	AML	clinical-phase 1	AML Patients in remission who have already undergone chemotherapy protocol	CR1	N/A	N/A	4 doses, 2 weeks interval	ID	N/A	8 weeks	(1) Ex vivo; (2) Molecular analysis; (3) Flow cytometry; (4) ELISA	Activation of CD4^+^ T cells by MHC-II molecules influences disease progression; The use of blocking antibodies promoted a modest increase in survival; Vaccination induced the formation of multifunctional cytokine-producing T cells and memory T cells; The cytokine IFN-γ is important in the anti-leukemic response.	Limited immunological assessments of vaccine response.	[29]
Peptides derived from WT1	DC vaccine	ALL, AML and HL	clinical-phase 1 and 2	HLA-A2 patients in relapse after allo-HSCT expressing WT1	Relapse	N/A	N/A	6 doses, 2 weeks interval	SC or ID	Cell inoculation—Donnor lymphocytes	36 weeks	(1) ELISPOT; (2) DTH	Vaccine formulation safe for administration in patients; Despite segment loss, 3 patients presented DTH; Vaccination induced IFN-γ production.	Limited immunological assessments of vaccine response; Early study, limited conclusive data on the effect of treatment on disease progression in patients.	[30]
mRNA hTERT (human telomerase reverse transcriptase)	DC vaccine	AML	clinical-phase 2	Patients with AML in first or second remission	CR1 or CR2	N/A	N/A	Cycle 1: 6 doses, 1 week interval; cycle 2: 6 doses, 2 weeks interval	ID	N/A	2 years	(1) Histological analysis; (2) ELISPOT; (3) DTH; (4) Molecular analysis	74% of vaccinated patients remained in remission during the study follow-up period; In 69% of patients in remission, specific T cells developed; Patients who were in second remission remained in this state for 24 months; In 5/7 patients over 60 years of age, T cell responses were observed, and there was no recurrence of the disease during the follow-up period.	Loss of patient follow-up throughout the study.	[31]
Leukemic tumor cell exosomes (LEX) transfected with a lentiviral vector containing a shRNA (small harpin RNA) sequence	Exosome vaccine	ALL	preclinical	N/A	N/A	DBA/2 mice	SC	3 doses, 5–7 days interval	SC	N/A	50 days	(1) Imaging analysis; (2) Molecular analysis; (3) Flow cytomety; (4) Ex vivo; (5) Overall survival	In vitro, LEX TGF-B1si captured by DCs induces pro-inflammatory cytokines and CD4^+^ T cell proliferation; Immunization induces increased cytotoxic activity of cytotoxic T lymphocytes and NK cells; In vivo, the presence of LEX TGF-β1si induced increased protection against tumor establishment; Increased survival rate.	Limitations due to this being a preclinical study.	[32]
Synthetic peptide OCV-501 derived from WT1	Peptide vaccine	AML	preclinical and clinical-phase 1	AML patients HLA-DRB1*01:01, *04:05, *15:01, *15:02, *08:03, or *09:01 in remission who have already undergone chemotherapy protocol	CR	N/A	N/A	4 doses, 4 weeks interval	SC	N/A	50 days	(1) In vitro; (2) Ex vivo; (3) Flow cytometry; (4) ELISA; (5) Analytical chemistry assay; (6) Safety and cytotoxicity; (7) Molecular assays	In vitro, the vaccine peptide induced the differentiation and proliferation of Th1 cells from PBMC; An increase in specific cytotoxic T cells was also observed; In clinical studies, the vaccine formulation was safe for administration to patients; During the observation period, patients did not present relapse; Vaccination induced a DTH response in patients; 4/9 patients presented reduced WT1 mRNA expression (MRD).	This is an initial study with limited conclusive data on the impact of treatment on disease progression in patients.	[33]
Inactivated WEHI-3 total leukemic cell antigen	Cell lysate vaccine	AML	preclinical	N/A	N/A	Balb/c mice	IV	4 doses, 3 days interval	SC	Cytokine-G-CSF	50 days	(1) Ex vivo; (2) In vitro; (3) Imaging analysis; (4) ELISA; (5) Flow cytometry; (6) Overall survival	G-CSF associated with the vaccine induced the reversal of the immunosuppressive tumor microenvironment by reducing Treg cells in the bone marrow and increasing these cells in the peripheral blood; The treatment increased the survival rate of vaccinated animals.	Limitations due to it being a preclinical study.	[34]
MMSA-1 (multiple myeloma special antigen) and DKKP-1 (Dickkopft-(1))	Peptide vaccine	MM	preclinical and clinical-phase 1	MM patients	N/A	SCID mice	SC	3 doses, 1 week interval	SC	N/A	60 days	(1) Molecular analysis; (2) ELISA; (3) Ex vivo; (4) Flow cytometry; (5) Overall survival; (6) Hematological assays; (7) Imaging analysis	MMSA-1 expression is essential in the establishment of the disease; In vitro MMSA-1 peptides induced the formation of specific cytotoxic T cells from PBMC of patients; In vivo it was found that the vaccine induced an increase in cytotoxic T cells producing IFN-γ and IL-2 and a reduction in Treg cells; Vaccination led to a reduction in tumor volume and bone degradation with an increase in the survival rate.	Limitations due to this being a preclinical study; Need to use transgenic animals.	[35]
Tumor cell lysate (TCL)		DLBCL and MCL	preclinical	N/A	N/A	NOD/SCID mice	SC	3 doses, 1 week interval	IP	Cytokine-IFN-α; Vitamin-retinoic acid	43 days	(1) Flow cytometry; (2) ELISA; (3) Ex vivo; (4) Imaging analysis	In vitro, DCs incubated with TCL-RA-IFN-α induced tumor cell death by inducing CRT expression in them and increasing phagocytosis of these cells by DCs; Increased immunogenicity of cytotoxic T cells; In vivo, the vaccine led to inhibition of tumor growth.	Limitations due to this being a preclinical study; Need to use transgenic animals.	[36]
WT1 mRNA	DC vaccine	AML	clinical-phase 2	AML patients at high risk of relapse	CR	N/A	N/A	16 doses, 2 weeks interval	ID	N/A	10 years	(1) Molecular analysis; (2) ELISA; (3) Flow cytometry; (4) DTH assay; (5) Histological analysis	Vaccination induced molecular remission in 9 patients by reducing WT1 transcripts in blood and bone marrow; 2/9 patients went from partial to complete remission; Vaccination led to an increase in the frequency of IFN-γ and TNF-α-producing cytotoxic T lymphocytes and increased survival rate.	Limited immunological assessments of vaccine response.	[37]
A20 tumor cell lysate	Cell lysate vaccine	DLBCL	preclinical	N/A	N/A	Balb/c mice	IP	Schedule 1: 3 doses, days 1, 7, and 15; Schedule 2: 3 doses, days 7, 10, and 14	SC	Glycolipid-α-GalCer	160 days	(1) Flow cytometry; (2) ELISA; (3) Ex vivo; (4) Imaging analysis; (5) Multiplex assay	Vaccination induced protection with increased animal survival; There was an increase in NK cells and cytotoxic T cells producing IFN-γ; The association with α-GalCer intensified the cellular response and reduced the number of Tregs.	Limitations arise from the study being preclinical in nature, restricting direct applicability to clinical settings.	[38]
Whole antigen of K-562 cells expressing GM-CSF and autologous tumor cells	Cell lysate vaccine	MDS and AML	clinical-phase 1	Patients with advanced MDS or high-risk AML	Advanced disease, high risk	N/A	N/A	Cycle 1: 3 doses, 1 week interval; Cycle 2: 3 doses, 2 week interval	SC/ID	Cell inoculation-HSCT	6 years	(1) Hematology assay; (2) DTH assay; (3) Flow cytometry; (4) ELISA	Safe vaccine formulation for administration in patients, 39% of patients presented relapse-free survival during 5 years of follow-up; Vaccination induced an increase in CD4^+^ and CD8^+^ cells in the initial 2 months of treatment; Biopsies of the vaccination site confirmed the influx of DCs, macrophages, neutrophils and lymphocytes.	Loss of patient follow-up throughout the study; Limited immunological assessments of vaccine response.	[39]
Synthetic peptide WT4869 derived from WT1	Peptide vaccine	MDS	clinical-phase 1 and 2	MDS patients unresponsive to conventional treatments	N/A	N/A	N/A	Escalonade dose 3 + 3, 2 weeks interval	ID	N/A	3 years	(1) Safety and cytotoxicity; (2) DTH assay; (3) Flow cytometry; (4) Molecular assay; (5) Ex vivo	The vaccine formulation presented grade 3 or higher adverse effects in 19 of the 26 patients; Vaccination promoted an improvement in the hematological condition of the patients; The patients presented a DTH response and 11/25 patients presented an increase in cytotoxic T cells; There was an increase in the mean survival rate of 64.71%; At the end of the study, 13/26 patients died.	Limited immunological assessments of vaccine response; Adverse effects of vaccination.	[40]
DKKP-1 (Dickkopft-(1)) antigen and HSP70	DNA vaccine	MM	preclinical	N/A	N/A	Balb/c	SC	3 doses, interval not reported	IM	N/A	100 days	(1) Safety and cytotoxicity; (4) Histological analysis; (3) Flow cytometry; (4) Molecular analysis; (5) Ex vivo	The vaccine formulation induced tumor regression in both prophylactic and therapeutic models; vaccination promoted an increase in IFN-γ-producing CD4^+^ and CD8^+^ cells and a decrease in T-reg cells in the spleen; it enhanced CTL activity and antigen-specific antibody responses; the number of apoptotic tumor cells was increased in the vaccinated group.	Limitations due to the preclinical nature of the study.	[41]
Synthetic peptides derived from WT1	Peptide vaccine	AML	preclinical	N/A	N/A	C57BL/6J CD45.1 and CD45.2 mice	SC	1 dose	SC	N/A	N/A	(1) Histological analysis; (2) Flow cytometry; (3) Molecular analysis; (4) In silico-Bioinformatics	Vaccination with 2 peptides promoted an increase in CD4^+^ T cells and mainly CD8^+^ T cells and DCs with induction of necrotic lesions in the tumor; Vaccination also induced an increase in the production of IFN-γ and TNF-α by specific CD4^+^ and CD8^+^ T cells in addition to effector memory and resident memory T cells.	Limitations due to this being a preclinical study; Need to use transgenic animals.	[42]
Synthetic peptides derived from WT1	Peptide vaccine	AML	clinical-phase 2	AML patients in remission who have already undergone chemotherapy protocol	CR1	N/A	N/A	6 doses, 2 weeks interval	SC	Cytokine-G-CSF	80 months	(1) Hematological analysis; (2) Molecular analysis; (3) Flow cytometry; (4) ELISPOT	Safe vaccine formulation for administration in patients; Vaccination promoted an increase in the overall survival rate (67.6 months); The mean disease-free survival was 16.9 months; 11/22 patients remained alive at the end of the study, and 9 of these remained in remission; Vaccination induced an increase in specific IFN-γ-producing CD8^+^ T cells and the proliferation of CD4^+^ T cells.	Loss of patient follow-up throughout the study.	[43]
Whole antigens associated with AML	DC vaccine	AML	clinical-phase 1	AML patients in remission who have already undergone chemotherapy protocol	CR2, de novo CR1, smolerding AML	N/A	N/A	4 doses, 2 weeks interval	ID	N/A	2 years	(1) Ex vivo; (2) Flow cytometry; (3) In vitro; (4) Safety and cytotoxicity	Vaccine formulation safe for administration in patients; After 126 days of follow-up, 9/12 patients remained alive and 6/12 patients remained in remission; Vaccination induced an increase in CD4^+^, CD8^+^, NK T cells, and DCs at the immunization site; Cytokine evaluation showed the induction of a Th1 or mixed response with production of IFN-γ, IL-2, IL-4, IL-6, IL-10, and IL-17.	Limited immunological assessments of vaccine response; Early study with limited data, resulting in insufficient conclusive evidence on the impact of treatment on disease progression in patients.	[44]
Whole antigens associated with AML	Cell lysate vaccine	AML	preclinical	N/A	N/A	C57BL/6 mice	IV	1 dose	IV	mAB-anti-PDL1, anti-4-1BB	120 days	(1) Imaging analysis; (2) Flow cytometry; (3) Overall survival	Vaccination associated with anti-4-1BB mAB promoted the cure of 100% of the animals; There was an increase in central and effector memory NK and CD8^+^ T cells; The adoptive transfer of effector memory T cells to sick animals confirmed the importance of this population in controlling the disease.	Limitations due to it being a preclinical study.	[45]
Whole antigens of parental leukemic cells	Cell lysate vaccine	AML	preclinical	N/A	N/A	C3H mice	IV	3 doses, 2 weeks interval	SC	N/A	450 days	(1) Flow cytometry; (2) ELISA; (3) Ex vivo; (4) In vitro; (5) ELISPOT; (6) Overall survival; Molecular analysis	Vaccination induced proliferation of CD4^+^ and CD8^+^ T cells, increased cytolytic activity and IFN-γ production; There was an increase in the survival rate of the animals.	Limitations due to it being a preclinical study.	[46]
Synthetic peptides derived from WT1	Peptide vaccine	AML	clinical-phase 1	HLA-A*02 patients with MDS or AML	CR ou CRi	N/A	N/A	6 doses, 2 weeks interval	SC	N/A	14 months	(1) Molecular analyis; (2) ELISPOT; (3) Ex vivo	Safe vaccine formulation for administration in patients; Vaccination induces an increase in CD8^+^ T cells with IFN-γ production for both AML and MDS patients.	Limited immunological assessments of vaccine response; As this is an early-stage study, there is limited conclusive data on the impact of treatment on disease progression in patients.	[47]
Tumor cell exosomes	DC vaccine	DLBCL	preclinical	N/A	N/A	C57BL/6, Balb/c and NOD/SCID mice	SC	3 doses, 2 weeks interval	IV	N/A	56 days	(1) Imaging analysis; (2) Flow cytometry; (3) Ex vivo; (4) Molecular analysis	Vaccination stimulated the proliferation of specific cytotoxic T cells with production of TNF-α and IL-6 and anti-tumor response.	Limitations due to being a preclinical study; Necessity for the use of transgenic animals.	[48]
Whole antigen of K-562 cell	Cell lysate vaccine	MDS	clinical phase 1	Patients with any subtype of MDS who have not been treated for at least 2 months and have not undergone HSCT	N/A	N/A	N/A	5 doses, weeks 0, 3, 6, 9 and 17	ID	N/A	22 weeks	(1) Ex vivo; (2) Multiplex assay; Molecular assay	Vaccine formulation safe for administration in patients; Vaccination was not able to induce cell proliferation in in vitro culture samples from all patients; Patients who responded were observed to have an increase in IL-6 in cell cultures; TCR sequencing analysis of a single patient showed that the vaccine induced a polyclonal expansion of CD4^+^ and CD8^+^ T cells.	Limited immunological assessments of vaccine response; In the early stages of study, there are limited conclusive data on the impact of treatment on disease progression in patients.	[49]
scFv-CCL20 plasmid DNA	DNA vaccine	LPL	clinical phase 1	Asymptomatic patients not treated with LPL	N/A	N/A	N/A	3 doses, 1 month interval	ID	N/A	1 year	(1) Ex vivo; (2) ELISPOT (3) Hematological analysis; (4) Histological analysis; (5) Safety and cytotoxicity	Safe vaccine formulation for administration to patients.	Limited immunological assessments of vaccine response; In the early stages of study, there are limited conclusive data on the impact of treatment on disease progression in patients.	[50]
Survivin-MUC1 protein	DC vaccine	AML and ALL	preclinical, clinical-phase 1 and 2	Patients with relapse after allo-HSCT	Relapse	Rhesus monkeys	N/A	Patients: 4 doses, 2 days interval; animals 1 dose	SC-humans/IM-monkeys	N/A	10 years (humans) 14 days (animal)	(1) Flow cytometry; (2) ELISA; (3) Ex vivo; (4) ELISPOT; (5) Hematological analysis; (6) Molecular analysis; (7) Histological analysis	In vitro, it was observed that Ad-siSSF was able to activate DCs and that Ad-siSSF-DCs activated cytotoxic T cells; In vivo, vaccination promoted an increase in IFN-γ, increasing the survival rate of animals; Vaccination also induced a reduction in WT1 expression and a reduction in MRD.	Difficulty in using non-human primates.	[51]
L5178Y-R tumor cell lysate (TCL) treated with PKHB1 (PKHB1-TCL)	Cell lysate vaccine	ALL	preclinical	N/A	N/A	Balb/c mice	SC	4 doses, 3 days interval	SC	N/A	150 days	(1) Ex vivo; (2) In vitro; (3) Flow cytometry; (4) Overall survival	In vitro, the vaccine peptide induced tumor cell lysis, dendritic cell activation, and production of IFN-γ, TNF-α, and IL-2 and T cell activation; In vivo, vaccination induced tumor reduction (80%), memory cell production and increased survival rate. When challenged again, the immunized animals protected 100% of the animals.	Limitations due to it being a preclinical study.	[52]
PLK1 derived peptides	DC vaccine	AML	preclinical	N/A	N/A	C57BL/6 mice	SC	2 doses, 1 week interval	IV	mAB-anti-PDL1	100 days	(1) Ex vivo; (2) Flow cytometry; (3) ELISPOT; (4) In vitro; (5) Overall survival; (6) Molecular analysis	2/8 vaccine peptides showed stimulation and expansion of cytotoxic T cells producing IFN-γ. Vaccination with these isolated peptides induced tumor reduction; The immunized animals proved resistant to the establishment of the disease after a new challenge; Vaccination was also able to protect the animals against more than one type of tumor. There was an increase in the survival rate. The association of the vaccine with anti-PD-L1 treatment further increased cytotoxic T cells with greater activity. The association of the peptides promoted better vaccine performance than the isolated vaccines.	Limitations due to it being a preclinical study.	[53]
mRNA encoding WT1, PRABE and (CMV)pp65 antigens	DC vaccine	AML	clinical-phase 1	AML patients in remission who have already undergone chemotherapy protocol	CR	N/A	N/A	10 doses total: 4 doses, 1 week interval + 1 dose, 2 weeks interval + 5 doses, 4 weeks interval	ID	N/A	3 years	(1) Ex vivo; (2) Flow cytometry; (3) DTH assay; (4) Histological analysis; (5) ELISPOT; (6) ELISA; 7 Safety and cytotoxicity	Safe vaccine formulation for administration in patients; Vaccination promoted a 50% recurrence rate; Patients under 65 years of age had a better immune response with the presence of specific T cells.	Small sample size, there is not much conclusive data on the effect of treatment on disease progression in patients.	[54]
Exosomes from tumor cells expressing CD80/86	Exossome vaccine	ALL	preclinical	N/A	N/A	DBA/2 mice	SC	3 doses, 1 week interval	SC	N/A	81 days	(1) Flow cytometry; (2) Imaging analysis; (3) Molecular analysis; (4) ELISA; (5) Ex vivo; (6) Overall survival	Vaccination reduced tumor size and increased survival rate; immunized animals showed greater proliferation of CD4^+^ T cells, greater activity of cytotoxic T cells, production of pro-inflammatory cytokines and maturation of DCs.	Limitations due to it being a preclinical study.	[55]
Whole antigens expressed on U937 cells	Cell lysate vaccine	AML	preclinical	N/A	N/A	Balb/c mice	SC	4 doses, 1 week interval	SC	N/A	30 days	(1) Ex vivo; (2) In vitro; (3) Flow cytometry; (4) ELISA; (5) Molecular analysis; (6) Overall survival	Vaccination promoted in vitro the activation of DCs and CTLs with production of pro-inflammatory cytokines; It was observed that the vaccine was able to reduce tumor size and increase the survival rate of treated animals.	Limitations due to it being a preclinical study.	[56]
Synthetic peptide RT53	Peptide-pulsed splenocyte vaccine	APL	preclinical	N/A	N/A	FVB/N mice	IV	1 dose	SC	N/A	260 days	(1) Flow cytometry; (2) Ex vivo; (3) ELISA; (4) Overall survival	The RT53 peptide promoted the death of leukemic cells in vitro; Vaccination induced an increase in the survival rate; Both the therapeutic and prophylactic approaches were able to protect the animals from the development of the disease with an important role of CD4^+^ T cells.	Limitations due to this being a preclinical study; Need to use transgenic animals; Limited immunological evaluations of the vaccine response.	[57]
Long peptide (19aa) IO103	Peptide vaccine	MM	clinical-phase 1	MM patients who have already undergone chemotherapy protocol in the last 6 months and allo-HSCT	N/A	N/A	N/A	15 total doses: 6 doses, 2 weeks interval + 9 doses, 4 weeks interval	SC	N/A	3 years	(1) Ex vivo; (2) DTH assay; (3) ELISPOT; (4) Flow cytometry; (5) Multiplex assay	Safe vaccine formulation for administration in patients; Patients showed DTH response; Lymphocytes from the biopsy site were able to produce IFN-γ and TNF-α; 8/10 patients remained alive at the end of the study.	Limited immunological assessments of vaccine response; Small sample size, there is not much conclusive data on the effect of treatment on disease progression in patients.	[58]
Antigens from the cell membrane of leukemic cells	Cell-membrane-coated nanoparticle vaccine	AML	preclinical	N/A	N/A	C57BL/6 mice	IV	3 doses, 2 ou 7 dias de intervalo	SC	N/A	12 weeks	(1) In vitro; (2) Overall survival; (3) Imaging analysis; (4) Flow cytometry; (5) Ex vivo; (6) ELISPOT; (7) Overall survival	Vaccination induced the activation of specific IFN-γ-producing T cells; the formation of central and effector memory cells and an increase in the survival rate were also observed.	Limitations due to it being a preclinical study.	[59]
WT1 and HAGE derived peptides	DNA vaccine	CML	preclinical	N/A	N/A	HHDII-DR1 transgenic mice	SC	3 doses, 1 week interval	ID	N/A	60 days	(1) In vitro; (2) Ex vivo; (3) ELISPOT; (4) Imaging analysis; (5) Flow cytometry	Vaccination induced the activation of specific IFN-γ-producing T cells; the formation of central and effector memory cells and an increase in the survival rate were also observed.	Limitations due to this being a preclinical study; Absence of a specific model for studying the disease in vivo.	[60]
Whole antigens expressed in leukemic cells	DC vaccine	AML	preclinical	N/A	N/A	C57BL/6 mice	IV	1 dose	SC	mAB-anti-PDL1, anti-TIM3, anti-RGMb	180 days	(1) Ex vivo; (2) Flow cytometry; (3) Molecular analysis	Vaccination associated with anti-PD-1, anti-TIM3, and anti-RGMb mABs showed 100% survival for 90 days; In a second challenge, these animals maintained 100% survival; Vaccination induced an increase in IFN-γ-producing cytotoxic T lymphocytes, an increase in memory T cells and a reduction in Treg cells.	Limitations due to it being a preclinical study.	[61]
Synthetic peptides derived from BCRs	DC vaccine	CLL	preclinical and clinical-phase 1	Patients with severe and progressive CLL	Progressive	C57BL/6N mice	IP	1 dose	ID	N/A	200 days	(1) Ex vivo; (2) ELISPOT; (3) Flow cytometry; (4) ELISA	Vaccination promoted an increase in IFN-γ-producing T cells and increased cytotoxicity when cultured with Eu-TCL1 cells; T cells from vaccinated mice were able to produce IFN-γ when cultured with other tumor clones; There was an increase in the survival rate.	Limitations due to it being a preclinical study; Only a prophylactic study, absence of an in vivo therapeutic study.	[62]
Whole antigens expressed in K562/GM-CSF cells	Cell lysate vaccine	CML	clinical-phase 2	CML patients on chemotherapy protocol	Chronic phase	N/A	N/A	Up to 17 doses, 3 weeks interval	ID	Cytokines-IFN-α, GM-CSF	60 months	(1) Molecular assay; (2) Safety and cytotoxicity; (3) Flow cytometry	Vaccination promoted an increase in IFN-γ-producing T cells and increased cytotoxicity when cultured with Eu-TCL1 cells; T cells from vaccinated mice were able to produce IFN-γ when cultured with other tumor clones; There was an increase in the survival rate.	Loss of patient follow-up throughout the study; The vaccine was not very effective.	[63]
mRNA encoding the CDR3 region of the TCR hypervariable chain	mRNA vaccine	TCL and CTCL	preclinical	N/A	N/A	C57BL/6 mice	SC	2 doses, 1 week interval	IV	N/A	N/A	(1) Flow cytometry; (2) ELISA; (3) Overall survival	Vaccination was able to induce a specific immune response against malignant T cells and promoted tumor reduction.	Limitations due to it being a preclinical study; Conducted as a prophylactic study with no in vivo therapeutic assessment.	[64]
WT1 antigen	Cell vector vaccine	AML	clinical-phase 1	AML patients in remission who have already undergone chemotherapy or are refractory	Relapse or refractory	N/A	N/A	2 doses, 1 week interval	IV	N/A	12 months	(1) Molecular assay; (2) Safety and cytotoxicity; (3) Flow cytometry; (4) Hematological analysis; (5) ELISPOT; (6) In silico-Bioinformatics	Vaccination was able to induce a specific immune response against malignant T cells and promoted tumor reduction.	Loss of patient follow-up throughout the study; Adverse effects of vaccination.	[65]
Synthetic peptides derived from PD-L1 and PD-L2	Peptide vaccine	CLL	clinical-phase 1 and 2	Untreated CLL patients with IgHV gene	Progressive	N/A	N/A	9 total doses: 6 doses, 2 weeks interval + 3 doses, 1 month interval	N/A	N/A	52 weeks	(1) Safety and cytotoxicity; (2) ELISPOT; (3) Flow cytometry	Safe vaccine formulation for administration in patients; 17/19 patients remained with stable disease during follow-up; Vaccination induced an increase in IFN-γ-producing T cells and effector memory T cells.	Loss of patient follow-up throughout the study.	[66]
VEE virus RNA replicating particles encoding FLT3	Recombinant virus vaccine	AML and BCL	preclinical	N/A	N/A	Balb/c and B6. SJL mice	IV and SC	Scheme 1: 2 doses, 2 weeks interval; Scheme 2: 3 doses, 2 weeks interval	SC	N/A	42 days	(1) Flow cytometry; (2) ELISA; (3) Molecular analysis	Vaccination induced an increase in specific IgG-producing B cells, cytotoxic T cells; the increase in specific responsive antibodies bound to tumor cells attenuated tumor growth.	Limitations due to this being a preclinical study; Limited immunological assessments of the vaccine response.	[67]
Exosomes from dendritic cells stimulated with leukemic cell lysate	Exosome vaccine	CML	preclinical	N/A	N/A	Balb/c mice	IV	14 doses, 2 days interval	ID	N/A	180 days	(1) In vitro; (2) Imaging analysis; (3) Flow cytometry; (4) Ex vivo; (5) ELISPOT; (6) ELISA; (7) Hematological analysis; (8) Overall survival	In vitro, it was observed that DEX presented high affinity with T and NK cells, with increased activation, proliferation, and cytotoxic potential with production of IFN-γ and TNF; In vivo, vaccination protected 100% against the development of the disease.	Limitations due to it being a preclinical study; Absence of a specific model for studying the disease in vivo.	[68]
WT1-A10 protein derived from WT1	Protein vaccine	AML	clinical-phase 1	AML patients expressing WT1 transcripts in blasts	CR, PR and CRi	N/A	N/A	Cycle 1: 6 doses, 2 weeks interval; Cycle 2: 6 doses, 3 weeks interval; Cycle 3: 4 doses, 6 weeks interval; Cycle 4: 4 doses, 3 months interval; Cycle 5: 4 doses, 6 months interval	IM	N/A	75 months	(1) Molecular analysis; (2) In silico Bioinformatics; (3) ELISA; (4) Flow cytometry	Safe formulation for administration in patients; Vaccination induced an increase in the humoral response with production of specific antibodies and cellular response with an increase in CD4^+^ and CD8^+^ T cells; WT1 expression was reduced in vaccinated patients and consequent absence of MRD.	Small sample size, there are not many conclusive data on the effect of treatment on disease progression in patients.	[69]
WT1 protein expressed by *B. longum*	Recombinant bacterial vaccine	AML	preclinical	N/A	N/A	C57BL/6 mice	SC	10 total doses: 1 dose/day for 5 days/week + 1 dose/day for 5 days/week	Oral	N/A	24 days	(1) Ex vivo; (2) Flow cytometry; (3) Overall survival	Vaccination associated with *B. longum* 420/2656 showed tumor reduction; There was an increase in cytotoxic T lymphocytes in the peripheral blood and an increase in effector memory T cells; Vaccination induced an increase in CD4^+^ T cells in the tumor infiltrate.	Limitations due to it being a preclinical study.	[70]
WT1 protein expressed by *B. longum*	Recombinant bacterial vaccine	AML	preclinical	N/A	NA	C57BL/6 mice	SC	10 total doses: 1 dose/day for 5 days/week + 1 dose/day for 5 days/week	Oral	N/A	30 days	(1) Flow cytometry; (2) Histological analaysis; (3) Ex vivo; (4) ELISA	Vaccination promoted tumor reduction due to the presence of cytotoxic T cells; An increase in cytotoxic T cells was observed in the peripheral blood in addition to the increase in specific IgG antibodies; Vaccination showed an increase in DCs and CD4^+^ T cells producing IFN-γ.	Limitations due to it being a preclinical study.	[71]
WT1 protein and OVA	mRNA vaccine	AML	preclinical	N/A	N/A	C57BL/6 mice	IV	1 dose	IV	Cytokines-IL-2	120 days	(1) Flow cytometry; (2) Molecular analysis; (3) ELISPOT; (4) Overall survival	Vaccination induced activation of NK, iNK, CD4^+^ T, and cytotoxic T cells with increased production of IFN-γ, in addition to effector memory cells; There was an increase in the survival rate of the animals.	Limitations due to it being a preclinical study.	[72]
Cell lysate of W10-iPSCs	DC vaccine and T cell vaccine	ALL	preclinical	N/A	N/A	NOD/SCID mice	IV	3 doses, 1 week interval	IV	N/A	45 days	(1) Ex vivo; (2) Overall survival; (3) Flow cytometry; (4) Multiplex analysis; (5) Biochemical analyses	Vaccination did not present acute toxicity in animals; There was a reduction in circulating tumor cells and an increase in T cells in peripheral blood; Vaccination induced an increase in the cytokines IL-6, IFN-γ, and TNF and a reduction in cellular exhaustion factors.	Limitations due to this being a preclinical study; Limited immunological assessments of the vaccine response.	[73]

N/A: Not Applicable.

**Table 2:** Vaccine types with their advantages and disadvantages.

Vaccine Classification	Nº of Articles	References	Advantages	Disadvantages
1st generation	14	[26,34,36,38,39,44,45,46,49,52,56,59,61,63]	Simplified formulation	Risk of infection
			Strong immune response	Not suitable for immunocompromised
			Multivalent	
2nd generation	16	[20,21,23,30,33,35,40,42,43,47,53,57,58,62,66,69]	Easy antigen modification	Less immunogenic
			Non-infectious	Necessity of adjuvants
			Stable	Time-consuming production
3rd generation	5	[18,24,25,41,50,60]	Easy antigen modification	High cost of production
			Non-infectious	Potential risk of genetic integration
			Well-tolerated	Necessity of improved logistics of storage and transportation
			Strong immune response	
			Constitutional antigen expression	
4th generation	4	[65,73]	Non-infectious	Fast degradation by RNAses
			Readily designed	Necessity of delivery vehicles
			Mass production capability	Drugs can impact mRNA metabolism
			Strong T and B cell responses	
DC vaccines	16	[19,22,23,29,30,31,36,37,44,48,51,53,54,61,62,73]	Relatively simple to obtain DCs from monocytes (MoDCs)	Require MHC match for antigen presentation
			Ready-to-use when acquired from allogeneic transplant	Migratory capability is affected by long-term cultures
			Increase the chances of cross-presentation of targeted antigens	Depending on source (e.g., from CD34^+^ hematopoietic precursors), they provide a heterogeneous population
			Can be obtained from different sources and provide a gamut of possibilities for antigen presentation/immune system activation	In vitro cultured MoDCs shown some differences from natural DCs

## Supplementary Materials

The following supporting information can be downloaded at: https://www.mdpi.com/article/10.3390/vaccines13020114/s1, PRISMA-ScR checklist and the list of excluded articles.
