# Supplementary material for: Therapeutic Vaccines for Hematological Cancers: A Scoping Review of This Immunotherapeutic Approach as Alternative to the Treatment of These Malignancies
Source: Vaccines (Basel). 2025 Jan 23;13(2):114. doi: 10.3390/vaccines13020114 (PMC11860334; doi:10.3390/vaccines13020114)
Supplement: Supplementary file 1 [file vaccines-13-00114-s001.zip › vaccines-3329059-supplementary.pdf]

Preferred Reporting Items for Systematic reviews and Meta-Analyses extension for Scoping Reviews (PRISMA-ScR) Checklist

SECTION	ITEM	PRISMA-ScR CHECKLIST ITEM	REPORTED ON PAGE #
**TITLE**			
Title	1	Identify the report as a scoping review.	1
**ABSTRACT**			
Structured summary	2	Provide a structured summary that includes (as applicable): background, objectives, eligibility criteria, sources of evidence, charting methods, results, and conclusions that relate to the review questions and objectives.	1
**INTRODUCTION**			
Rationale	3	Describe the rationale for the review in the context of what is already known. Explain why the review questions/objectives lend themselves to a scoping review approach.	1-2
Objectives	4	Provide an explicit statement of the questions and objectives being addressed with reference to their key elements (e.g., population or participants, concepts, and context) or other relevant key elements used to conceptualize the review questions and/or objectives.	2-3
**METHODS**			
Protocol and registration	5	Indicate whether a review protocol exists; state if and where it can be accessed (e.g., a Web address); and if available, provide registration information, including the registration number.	3-4
Eligibility criteria	6	Specify characteristics of the sources of evidence used as eligibility criteria (e.g., years considered, language, and publication status), and provide a rationale.	3-4
Information sources*	7	Describe all information sources in the search (e.g., databases with dates of coverage and contact with authors to identify additional sources), as well as the date the most recent search was executed.	3-4
Search	8	Present the full electronic search strategy for at least 1 database, including any limits used, such that it could be repeated.	3-4
Selection of sources of evidence†	9	State the process for selecting sources of evidence (i.e., screening and eligibility) included in the scoping review.	3-4
Data charting process‡	10	Describe the methods of charting data from the included sources of evidence (e.g., calibrated forms or forms that have been tested by the team before their use, and whether data charting was done independently or in duplicate) and any processes for obtaining and confirming data from investigators.	3-4
Data items	11	List and define all variables for which data were sought and any assumptions and simplifications made.	3-4
Critical appraisal of individual sources of evidence§	12	If done, provide a rationale for conducting a critical appraisal of included sources of evidence; describe the methods used and how this information was used in any data synthesis (if appropriate).	3-4
Synthesis of results	13	Describe the methods of handling and summarizing the data that were charted.	3-4

SECTION	ITEM	PRISMA-ScR CHECKLIST ITEM	REPORTED ON PAGE #
**RESULTS**			
Selection of sources of evidence	14	Give numbers of sources of evidence screened, assessed for eligibility, and included in the review, with reasons for exclusions at each stage, ideally using a flow diagram.	4-42
Characteristics of sources of evidence	15	For each source of evidence, present characteristics for which data were charted and provide the citations.	4-42
Critical appraisal within sources of evidence	16	If done, present data on critical appraisal of included sources of evidence (see item 12).	4-42
Results of individual sources of evidence	17	For each included source of evidence, present the relevant data that were charted that relate to the review questions and objectives.	4-42
Synthesis of results	18	Summarize and/or present the charting results as they relate to the review questions and objectives.	4-42
**DISCUSSION**			
Summary of evidence	19	Summarize the main results (including an overview of concepts, themes, and types of evidence available), link to the review questions and objectives, and consider the relevance to key groups.	4-42
Limitations	20	Discuss the limitations of the scoping review process.	42
Conclusions	21	Provide a general interpretation of the results with respect to the review questions and objectives, as well as potential implications and/or next steps.	42-43
**FUNDING**			
Funding	22	Describe sources of funding for the included sources of evidence, as well as sources of funding for the scoping review. Describe the role of the funders of the scoping review.	43

JBI = Joanna Briggs Institute; PRISMA-ScR = Preferred Reporting Items for Systematic reviews and Meta-Analyses extension for Scoping Reviews.

* Where *sources of evidence* (see second footnote) are compiled from, such as bibliographic databases, social media platforms, and Web sites.

† A more inclusive/heterogeneous term used to account for the different types of evidence or data sources (e.g., quantitative and/or qualitative research, expert opinion, and policy documents) that may be eligible in a scoping review as opposed to only studies. This is not to be confused with *information sources* (see first footnote).

‡ The frameworks by Arksey and O'Malley (6) and Levac and colleagues (7) and the JBI guidance (4, 5) refer to the process of data extraction in a scoping review as data charting.

§ The process of systematically examining research evidence to assess its validity, results, and relevance before using it to inform a decision. This term is used for items 12 and 19 instead of "risk of bias" (which is more applicable to systematic reviews of interventions) to include and acknowledge the various sources of evidence that may be used in a scoping review (e.g., quantitative and/or qualitative research, expert opinion, and policy document).

From: Tricco AC, Lillie E, Zarin W, O'Brien KK, Colquhoun H, Levac D, et al. PRISMA Extension for Scoping Reviews (PRISMAScR): Checklist and Explanation. Ann Intern Med. 2018;169:467–473. doi: 10.7326/M18-0850.

First step selection list		
Title	Authors	doi
Emerging role of natural products in cancer immunotherapy	Dong S, Guo X, Han F, He Z, Wang Y.	10.1016/j.apsb.2021.08.020
Targeting cancer stem cell pathways for cancer therapy	Yang L, Shi P, Zhao G, Xu J, Peng W, Zhang J, Zhang G, Wang X, Dong Z, Chen F, Cui H.	10.1038/s41392-020-0110-5
CAR-NK cell in cancer immunotherapy; A promising frontier	Marofi F, Abdul-Rasheed OF, Rahman HS, Budi HS, Jalil AT, Yumashev AV, Hassanzadeh A, Yazdanifar ...	10.1111/cas.14993
The Past, Present, and Future of Non-Viral CAR T Cells	Moretti A, Ponzo M, Nicolette CA, Tcherepanova IY, Biondi A, Magnani CF.	10.3389/fimmu.2022.867013
Immunotherapy in Acute Myeloid Leukemia: Where We Stand	Isidori A, Cerchione C, Daver N, DiNardo C, Garcia-Manero G, Konopleva M, Jabbour E, Ravandi F, K...	10.3389/fonc.2021.656218
Advances in Acute Myeloid Leukemia: Recently Approved Therapies and Drugs in Development	Stanchina M, Soong D, Zheng-Lin B, Watts JM, Taylor J.	10.3390/cancers12113225
Cytomegalovirus Infections after Hematopoietic Stem Cell Transplantation: Current Status and Future Immunotherapy	Cho SY, Lee DG, Kim HJ.	10.3390/ijms20112666
Recent Advances in Solid Tumor CAR-T Cell Therapy: Driving Tumor Cells From Hero to Zero?	Safarzadeh Kozani P, Safarzadeh Kozani P, Ahmadi Najafabadi M, Yousefi F, Mirarefin SMJ, Rahbariz...	10.3389/fimmu.2022.795164
The Rapid Development and Early Success of Covid 19 Vaccines Have Raised Hopes for Accelerating the Cancer Treatment Mechanism	Amanpour S.	10.22092/ari.2021.353761.1612
Neoantigens in Hematologic Malignancies	Biernacki MA, Bleakley M.	10.3389/fimmu.2020.00121
CAR-T "the living drugs", immune checkpoint inhibitors, and precision medicine: a new era of cancer therapy	Liu D.	10.1186/s13045-019-0819-1
Cancer Immunotherapy and Delivery System: An Update	Yang M, Olaoba OT, Zhang C, Kimchi ET, Staveley-O'Carroll KF, Li G.	10.3390/pharmaceutics14081630
Immunotherapy approaches for hematological cancers	Lanier OL, Pérez-Herrero E, Andrea APD', Bahrami K, Lee E, Ward DM, Ayala-Suárez N, Rodríguez-Mén...	10.1016/j.isci.2022.105326
Immune targets and neoantigens for cancer immunotherapy and precision medicine	Wang RF, Wang HY.	10.1038/cr.2016.155
Research progress on dendritic cell vaccines in cancer immunotherapy	Yu J, Sun H, Cao W, Song Y, Jiang Z.	10.1186/s40164-022-00257-2
Hypomethylating Agents and Immunotherapy: Therapeutic Synergism in Acute Myeloid Leukemia and Myelodysplastic Syndromes	Wong KK, Hassan R, Yaacob NS.	10.3389/fonc.2021.624742
Whole leukemia cell vaccines: Past progress and future directions	Ji YS, Park SK, Ryu S.	10.1016/j.vaccine.2020.03.042
Immunoglobulin therapy in hematologic neoplasms and after hematopoietic cell transplantation	Ueda M, Berger M, Gale RP, Lazarus HM.	10.1016/j.blre.2017.09.003
Vaccination Therapy for Acute Myeloid Leukemia: Where Do We Stand?	Barbullushi K, Rampi N, Serpenti F, Sciumè M, Fabris S, De Roberto P, Fracchiolla NS.	10.3390/cancers14122994
Self-replicating vehicles based on negative strand RNA viruses	Lundstrom K.	10.1038/s41417-022-00436-7
Immunotherapy for acute leukemia	Boyiadzis M, Whiteside TL, Pavletic SZ.	10.18632/aging.100768
Tim-3 and Tim-4 as the potential targets for antitumor therapy	Cheng L, Ruan Z.	10.1080/21645515.2015.1056953
Dendritic Cell-Based Cancer Immunotherapy Targeting Wilms' Tumor 1 for Pediatric Cancer	Shimodaira S, Hirabayashi K, Yanagisawa R, Higuchi Y, Sano K, Koizumi T.	10.15586/codon.wt.2016.ch8
Personal tumor antigens in blood malignancies: genomics-directed identification and targeting	Penter L, Wu CJ.	10.1172/JCI129209
Acute myeloid leukaemia and the immune system: implications for immunotherapy	Barrett AJ.	10.1111/bjh.16310
Advances in Cancer Immunotherapy in Solid Tumors	Menon S, Shin S, Dy G.	10.3390/cancers8120106
BITES and CARS and checkpoints, oh my! Updates regarding immunotherapy for myeloid malignancies from the 2018 annual ASH meeting	Przespolewski AC, Griffiths EA.	10.1016/j.blre.2020.100654
Antibody-Based Immunotherapeutic Strategies for the Treatment of Hematological Malignancies	Han Y, Liu Z, Liu J, Yan W, Xia Y, Yue S, Yu J.	10.1155/2020/4956946
Vaccine-associated feline sarcoma: current perspectives	Saba CF.	10.2147/VMRR.S116556
A perspective of immunotherapy for acute myeloid leukemia: Current advances and challenges	Chen Y, Wang J, Zhang F, Liu P.	10.3389/fphar.2023.1151032
BDCA1+CD14+ Immunosuppressive Cells in Cancer, a Potential Target?	van Ee TJ, Van Acker HH, van Oorschot TG, Van Tendeloo VF, Smits EL, Bakdash G, Schreibelt G, de ...	10.3390/vaccines6030065
T Cell Based Immunotherapy for Cancer: Approaches and Strategies	Want MY, Bashir Z, Najar RA.	10.3390/vaccines11040835
Wilms' Tumor 1 (WT1): The Vaccine for Cancer	Hein KZ, Yao S, Fu S.	10.36401/JIPO-20-12
New Era of Immunotherapy in Pediatric Brain Tumors: Chimeric Antigen Receptor T-Cell Therapy	Wu WT, Lin WY, Chen YW, Lin CF, Wang HH, Wu SH, Lee YY.	10.3390/ijms22052404
Immune Dysfunctions and Immune-Based Therapeutic Interventions in Chronic Lymphocytic Leukemia	Griggio V, Perutelli F, Salvetti C, Boccellato E, Boccadoro M, Vitale C, Coscia M.	10.3389/fimmu.2020.594556
Immunotherapy in acute myeloid leukemia	Grosso DA, Hess RC, Weiss MA.	10.1002/cncr.29378
Engineering Metabolism of Chimeric Antigen Receptor (CAR) Cells for Developing Efficient Immunotherapies	Mangal JL, Handlos JL, Esrafili A, Inamdar S, Mcmillian S, Wankhede M, Gottardi R, Acharya AP.	10.3390/cancers13051123
Immunooncology in Breast Cancer: Active and Passive Vaccination Strategies	Schütz F, Marmé F, Domschke C, Sohn C, von Au A.	10.1159/000486330
The cancer-immunity cycle as rational design for synthetic cancer drugs: Novel DC vaccines and CAR T-cells	Ramachandran M, Dimberg A, Essand M.	10.1016/j.semcancer.2017.02.010
Migratory Engineering of T Cells for Cancer Therapy	Michaelides S, Obeck H, Kechur D, Endres S, Kobold S.	10.3390/vaccines10111845
Unleashing Natural Killer Cells in the Tumor Microenvironment-The Next Generation of Immunotherapy?	Ben-Shmuel A, Biber G, Barda-Saad M.	10.3389/fimmu.2020.00275
Novel therapy in Acute myeloid leukemia (AML): moving toward targeted approaches	Winer ES, Stone RM.	10.1177/2040620719860645

Immunotherapy for acute myeloid leukemia: from allogeneic stem cell transplant to novel therapeutics	Knorr DA, Goldberg AD, Stein EM, Tallman MS.	10.1080/10428194.2019.1639167
Role of tumor microenvironment in cancer progression and therapeutic strategy	Wang Q, Shao X, Zhang Y, Zhu M, Wang FXC, Mu J, Li J, Yao H, Chen K.	10.1002/cam4.5698
Plant-made immunotoxin building blocks: A roadmap for producing therapeutic antibody-toxin fusions	Knödler M, Buyel JF.	10.1016/j.biotechadv.2020.107683
Innovative strategies to advance CAR T cell therapy for solid tumors	Huang M, Deng J, Gao L, Zhou J.	
Combining low-dose or metronomic chemotherapy with anticancer vaccines: A therapeutic opportunity for lymphomas	Sheng Sow H, Mattarollo SR.	10.4161/onci.27058
Cancer Resistance to Immunotherapy: Comprehensive Insights with Future Perspectives	Said SS, Ibrahim WN.	10.3390/pharmaceutics15041143
All systems go: converging synthetic biology and combinatorial treatment for CAR-T cell therapy	Lanitis E, Coukos G, Irving M.	10.1016/j.copbio.2020.01.009
CAR T-Cell Immunotherapy Treating T-ALL: Challenges and Opportunities	Ren A, Tong X, Xu N, Zhang T, Zhou F, Zhu H.	10.3390/vaccines11010165
Precision Tools in Immuno-Oncology: Synthetic Gene Circuits for Cancer Immunotherapy	Bonfá G, Blazquez-Roman J, Tarnai R, Siciliano V.	10.3390/vaccines8040732
Humoral and cellular immunogenicity of SARS-CoV-2 vaccines in chronic lymphocytic leukemia: a prospective cohort study	Haydu JE, Maron JS, Redd RA, Gallagher KME, Fischinger S, Barnes JA, Hochberg EP, Johnson PC, Tak...	10.1182/bloodadvances.2021006627
Immune Dysfunction in Patients with Chronic Lymphocytic Leukemia and Challenges during COVID-19 Pandemic	Langerbeins P, Eichhorst B.	10.1159/000514071
Wilms tumor 1 peptide vaccination after hematopoietic stem cell transplant in leukemia patients	Hosen N, Maeda T, Hashii Y, Tsuboi A, Nishida S, Nakata J, Oji Y, Oka Y, Sugiyama H.	10.21037/sci.2016.11.08
Combination Strategies for Immune-Checkpoint Blockade and Response Prediction by Artificial Intelligence	Huemer F, Leisch M, Geisberger R, Melchardt T, Rinnerthaler G, Zaborsky N, Greil R.	10.3390/ijms21082856
Methods of Controlling Invasive Fungal Infections Using CD8(+) T Cells	Kumaresan PR, da Silva TA, Kontoyiannis DP.	10.3389/fimmu.2017.01939
[Pathogenesis, prophylaxis and treatment of infections in patients with chronic lymphocytic leukemia]	Stelmach P, Robak T.	10.5604/17322693.1053901
Novel therapies in AML: reason for hope or just hype?	Larkin K, Blum W.	10.14694/EdBook_AM.2014.34.e341
The Role of Tumor-Associated Antigen HER2/neu in Tumor Development and the Different Approaches for Using It in Treatment: Many Choices and Future Directions	Alrhmoun S, Sennikov S.	10.3390/cancers14246173
CAR-T cells and allogeneic hematopoietic stem cell transplantation for relapsed/refractory B-cell acute lymphoblastic leukemia	Liu J, Zhang X, Zhong JF, Zhang C.	10.2217/imt-2017-0072
LGL leukemia patients exhibit substantial protective humoral responses following SARS-CoV-2 vaccination	Cheon H, Elghawy O, Shemo BC, Feith DJ, Loughran TP Jr.	10.1002/jha2.472
Keeping Myeloma in Check: The Past, Present and Future of Immunotherapy in Multiple Myeloma	Ackley J, Ochoa MA, Ghoshal D, Roy K, Lonial S, Boise LH.	10.3390/cancers13194787
Dendritic cell cancer vaccines: from the bench to the bedside	Katz T, Avivi I, Benyamini N, Rosenblatt J, Avigan D.	10.5041/RMMJ.10158
How Can We Prevent Mother-to-Child Transmission of HTLV-1?	Itabashi K, Miyazawa T, Uchimaru K.	10.3390/ijms24086961
Review of the Results of WT1 Peptide Vaccination Strategies for Myelodysplastic Syndromes and Acute Myeloid Leukemia from Nine Different Studies	Di Stasi A, Jimenez AM, Minagawa K, Al-Obaidi M, Rezvani K.	10.3389/fimmu.2015.00036
Cellular therapy against public neoantigens	Armistead PM.	10.1172/JCI126116
Prospects for immunotherapy of acute myeloid leukemia using γδ T cells	Halim L, Parente-Pereira AC, Maher J.	10.2217/imt-2016-0139
COVID-19 vaccines elicit robust cellular immunity and clinical protection in chronic lymphocytic leukemia	Parry H, Bruton R, Roberts T, McIlroy G, Damery S, Sylla P, Dowell AC, Tut G, Lancaster T, Bone D...	10.1016/j.ccell.2022.05.001
Joint consensus statement on the vaccination of adult and paediatric haematopoietic stem cell transplant recipients: Prepared on behalf of the British society of blood and marrow transplantation and cellular therapy (BSBMTCT), the Children's cancer and Leukaemia Group (CCLG), and British Infectio...	Miller P, Patel SR, Skinner R, Dignan F, Richter A, Jeffery K, Khan A, Heath PT, Clark A, Orchard...	10.1016/j.jinf.2022.11.005
The evolving clinical landscape for dendritic cell vaccines and cancer immunotherapy	Cannon MJ, Block MS, Morehead LC, Knutson KL.	10.2217/imt-2018-0129
Mesothelin-targeted CAR-T cells for adoptive cell therapy of solid tumors	Zhang GZ, Li TF, Han SY.	10.5114/aoms.2019.84888
Serological response to COVID-19 vaccination in patients with cancer older than 80 years	Iacono D, Cerbone L, Palombi L, Cavalieri E, Sperduti I, Cocchiara RA, Mariani B, Parisi G, Garuf...	10.1016/j.jgo.2021.06.002
The Role of TRL7/8 Agonists in Cancer Therapy, with Special Emphasis on Hematologic Malignancies	Leśniak M, Lipniarska J, Majka P, Kopyt W, Lejman M, Zawitkowska J.	10.3390/vaccines11020277
Chronic Lymphocytic Leukemia-Induced Humoral Immunosuppression: A Systematic Review	Grywalska E, Zaborek M, Łyczba J, Hrynkiewicz R, Bębnowska D, Becht R, Sosnowska-Pasiarska B, Smo...	10.3390/cells9112398
Chimeric antigen receptor T-cell therapy hits the market	Boyer MW.	10.2217/imt-2018-0075
Maintenance of long remission in adult T-cell leukemia by Tax-targeted vaccine: A hope for disease-preventive therapy	Kannagi M, Hasegawa A, Nagano Y, Iino T, Okamura J, Suehiro Y.	10.1111/cas.13948
Predictors of Humoral Response to SARS-CoV-2 Vaccination after Hematopoietic Cell Transplantation and CAR T-cell Therapy	Tamari R, Politikos I, Knorr DA, Vardhana SA, Young JC, Marcello LT, Doddi S, Devlin SM, Ramanath...	10.1158/2643-3230.BCD-21-0142
A vaccine against HTLV-1 HBZ makes sense	Mahieux R.	10.1182/blood-2015-06-652040

Herpes simplex virus lymphadenitis is associated with tumor reduction in a patient with chronic lymphocytic leukemia	Chang A, Sholukh AM, Wieland A, Jaye DL, Carrington M, Huang ML, Xie H, Jerome KR, Roychoudhury P...	10.1172/JCI161109
Antibody and T-cell responses to SARS-CoV-2 vaccination in myeloproliferative neoplasm patients	How J, Gallagher KME, Liu Y, Katsis K, Elder EL, Larson RC, Leick MB, Neuberg D, Maus MV, Hobbs GS.	10.1038/s41375-022-01533-0
Beat pediatric ALL MRD: CD28 CAR T and transplant	Ruella M, Locke FL.	10.1182/blood.2019003821
An intranasal stringent response vaccine targeting dendritic cells as a novel adjunctive therapy against tuberculosis	Karanika S, Gordy JT, Neupane P, Karantanos T, Ruelas Castillo J, Quijada D, Comstock K, Sandhu A...	10.3389/fimmu.2022.972266
Immune responses and long-term disease recurrence status after telomerase-based dendritic cell immunotherapy in patients with acute myeloid leukemia	Khoury HJ, Collins RH Jr, Blum W, Stiff PS, Elias L, Lebkowski JS, Reddy A, Nishimoto KP, Sen D, ...	10.1002/cncr.30696
Impaired humoral responses to COVID-19 vaccination in patients with lymphoma receiving B-cell-directed therapies	Ghione P, Gu JJ, Attwood K, Torka P, Goel S, Sundaram S, Mavis C, Johnson M, Thomas R, McWhite K,...	10.1182/blood.2021012443
An analogue peptide from the Cancer/Testis antigen PASD1 induces CD8+ T cell responses against naturally processed peptide	Hardwick N, Buchan S, Ingram W, Khan G, Vittes G, Rice J, Pulford K, Mufti G, Stevenson F, Guinn BA.	
Cationic liposomes bearing Bet v 1 by coiled coil-formation are hypo-allergenic and induce strong immunogenicity in mice	Warmenhoven H, Leboux R, Bethanis A, van Strien J, Logiantara A, van Schijndel H, Aglas L, van Ri...	10.3389/falgy.2022.1092262
Wilms tumour 1 peptide vaccine as a cure-oriented post-chemotherapy strategy for patients with acute myeloid leukaemia at high risk of relapse	Nakata J, Nakae Y, Kawakami M, Morimoto S, Motooka D, Hosen N, Fujiki F, Nakajima H, Hasegawa K, ...	10.1111/bjh.14768
Rejection of Leukemic Cells Requires Antigen-Specific T Cells with High Functional Avidity		https://doi.org/10.1016/j.bbmt.2013.10.020
Reinvigoration of innate and adaptive immunity via therapeutic cellular vaccine for patients with AML		https://doi.org/10.1016/j.omto.2022.09.001
Reduced humoral immune response after BNT162b2 coronavirus disease 2019 messenger RNA vaccination in cancer patients under antineoplastic treatment		https://doi.org/10.1016/j.esmoop.2021.100274
Neopepsee: accurate genome-level prediction of neoantigens by harnessing sequence and amino acid immunogenicity information		https://doi.org/10.1093/annonc/mdy022
Immunotherapy in ovarian cancer		https://doi.org/10.1093/annonc/mdx444
One-year breakthrough SARS-CoV-2 infection and correlates of protection in fully vaccinated hematological patients	Piñana J.L., Vazquez L., Calabuig M., López-Corral L., Martin-Martin G., Villalon L., Sanz-Linare... and Cell Therapy Group (G[illegible]	10.1038/s41408-022-00778-3
Duration of Replication-Competent Severe Acute Respiratory Syndrome Coronavirus 2 (SARS-CoV-2) Shedding Among Patients With Severe or Critical Coronavirus Disease 2019 (COVID-19)	Kim D.Y., Lin M.Y., Jennings C., Li H., Jung J.H., Moore N.M., Ghinai I., Black S.R., Zaccaro D.J...	10.1093/cid/ciac405
Clinical efficacy and long-term immunogenicity of an early triple dose regimen of SARS-CoV-2 mRNA vaccination in cancer patients	Lee M.X., Peng S., Lee A.R.Y.B., Wong S.Y., Tay R.Y.K., Li J., Tariq A., Goh C.X.Y., Tan Y.K., Ta...	10.47102/annals-acadmedsg.2022302
Predictive model for BNT162b2 vaccine response in cancer patients based on blood cytokines and growth factors	Konnova A., De Winter F.H.R., Gupta A., Verbruggen L., Hotterbeekx A., Berkell M., Teuwen L.-A., ...	10.3389/fimmu.2022.1062136
Optimal approach to T-cell ALL	O'Dwyer K.M.	10.1182/hematology.2022000337
Perspectives in Immunotherapy: meeting report from the Immunotherapy Bridge, December 1st–2nd, 2021	Ascierto P.A., Avallone A., Bhardwaj N., Bifulco C., Bracarda S., Brody J.D., Buonaguro L., Demar...	10.1186/s12967-022-03471-y
SARS-CoV-2 vaccine response and rate of breakthrough infection in patients with hematological disorders	Piñana J.L., López-Corral L., Martino R., Vazquez L., Pérez A., Martin-Martin G., Gago B., Sanz-L... and Cell Therapy Group (G[illegible]	10.1186/s13045-022-01275-7
Systemic Delivery of mPEG-Masked Trispecific T-Cell Nanoengagers in Synergy with STING Agonists Overcomes Immunotherapy Resistance in TNBC and Generates a Vaccination Effect	Shen M., Chen C., Guo Q., Wang Q., Liao J., Wang L., Yu J., Xue M., Duan Y., Zhang J.	10.1002/advs.202203523
Antibody response to a third booster dose of SARS-CoV-2 vaccination in adults with haematological and solid cancer: a systematic review	Al Hajji Y., Taylor H., Starkey T., Lee L.Y.W., Tilby M.	10.1038/s41416-022-01951-y
Efficacy of COVID-19 Booster Vaccines in Patients with Hematologic Malignancies: Experiences in a Real-World Scenario	Krekeler C., Reitnauer L., Bacher U., Khandanpour C., Steger L., Boeckel G.R., Klosner J., Tepass...	10.3390/cancers14225512
Antibody Response in Immunocompromised Patients with Hematologic Cancers Who Received a 3-Dose mRNA-1273 Vaccination Schedule for COVID-19	Haggenburg S., Hofsink Q., Lissenberg-Witte B.I., Broers A.E.C., Van Doesum J.A., Van Binnendijk ...	10.1001/jamaoncol.2022.3227
Short Research Communication Anti-Spike Antibody Response to COVISHIELD™ (SII-ChAdOx1 nCoV-19) Vaccine in Patients with B-Cell and Plasma Cell Malignancies and Hematopoietic Cell Transplantation Recipients	Chopra M., Jain A., Chhabra S., Kaundal S., Singh C., Jandial A., Prakash G., Khadwal A., Das C.,...	10.1007/s12288-022-01528-y
Predictors of poor serologic response to COVID-19 vaccine in patients with cancer: a systematic review and meta-analysis	Yang W., Zhang D., Li Z., Zhang K.	10.1016/j.ejca.2022.05.031
Absolute Lymphocyte Count After COVID-19 Vaccination Is Associated with Vaccine-Induced Hypermetabolic Lymph Nodes on 18F-FDG PET/CT: A Focus in Breast Cancer Care	Seban R.-D., Richard C., Nascimento-Leite C., Ghidaglia J., Provost C., Gonin J., Le Tourneau C.,...	10.2967/jnumed.121.263082
Antibody Response to SARS-CoV-2 Vaccination in Patients With Lymphoproliferative Disorders and Plasma Cell Dyscrasias: Anti-Lymphoma Therapy as a Predictive Biomarker of Response to Vaccination	Gung C., McGuire R., George M., Abdulkareem A., Belden K.A., Porcu P., Martinez-Outschoorn U., Bi...	10.3389/fonc.2022.840451

Immune Responses to SARS-CoV-2 Vaccination in Young Patients with Anti-CD19 Chimeric Antigen Receptor T Cell-Induced B Cell Aplasia	Jarisch A., Wiercinska E., Huenecke S., Bremm M., Cappel C., Hauler J., Rettinger E., Soerensen J...	10.1016/j.jtct.2022.04.017
Remdesivir in Coronavirus Disease 2019 patients treated with anti-CD20 monoclonal antibodies: a case series	Rüfenacht S., Gantenbein P., Boggian K., Flury D., Kern L., Dollenmaier G., Kohler P., Albrich W.C.	10.1007/s15010-022-01821-y
Rituximab-treated patients with lymphoma develop strong CD8 T-cell responses following COVID-19 vaccination	Riise J., Meyer S., Blaas I., Chopra A., Tran T.T., Delic-Sarac M., Hestdalen M.L., Brodin E., Ru...	10.1111/bjh.18149
Effectiveness, immunogenicity, and safety of COVID-19 vaccines for individuals with hematological malignancies: a systematic review	Piechotta V., Mellinghoff S.C., Hirsch C., Brinkmann A., Iannizzi C., Kreuzberger N., Adams A., M...	10.1038/s41408-022-00684-8
Risk and Outcome of Breakthrough COVID-19 Infections in Vaccinated Patients With Cancer: Real-World Evidence From the National COVID Cohort Collaborative	Song Q., Bates B., Shao Y.R., Hsu F.-C., Liu F., Madhira V., Mitra A.K., Bergquist T., Kavuluru R...	10.1200/JCO.21.02419
Immune responses against SARS-CoV-2 variants after two and three doses of vaccine in B-cell malignancies: UK PROSECO study	Lim S.H., Stuart B., Joseph-Pietras D., Johnson M., Campbell N., Kelly A., Jeffrey D., Turaj A.H....	10.1038/s43018-022-00364-3
Immunogenicity of a Third Dose of the BNT162b2 mRNA Covid-19 Vaccine in Patients with Impaired B Cell Reconstitution After Cellular Therapy—A Single Center Prospective Cohort Study	Ram R., Freund T., Halperin T., Ben-Ami R., Amit O., Bar-On Y., Beyar-Katz O., Eilaty N., Gold R....	10.1016/j.jtct.2022.02.012
Immunogenicity of COVID-19 Vaccinations in Hematological Patients: 6-Month Follow-Up and Evaluation of a 3rd Vaccination	Schubert L., Koblischke M., Schneider L., Porpaczy E., Winkler F., Jaeger U., Blüml S., Haslacher...	10.3390/cancers14081962
Acute myeloid leukemia cell membrane-coated nanoparticles for cancer vaccination immunotherapy	Johnson D.T., Zhou J., Kroll A.V., Fang R.H., Yan M., Xiao C., Chen X., Kline J., Zhang L., Zhang...	10.1038/s41375-021-01432-w
Mortality among Adults with Cancer Undergoing Chemotherapy or Immunotherapy and Infected with COVID-19	Várnai C., Palles C., Arnold R., Curley H.M., Purshouse K., Cheng V.W.T., Booth S., Campton N.A.,...	10.1001/jamanetworkopen.2022.0130
T-cell immune response after mRNA SARS-CoV-2 vaccines is frequently detected also in the absence of seroconversion in patients with lymphoid malignancies	Marasco V., Carniti C., Guidetti A., Farina L., Magni M., Miceli R., Calabretta L., Verderio P., ...	10.1111/bjh.17877
Safety of the BNT162b2 mRNA COVID-19 vaccine in oncologic patients undergoing numerous cancer treatment options: A retrospective single-center study	Kian W., Zemel M., Kestenbaum E.H., Rouvinov K., Alguayn W., Levitas D., Ievko A., Michlin R., Ab...	10.1097/MD.0000000000028561
Systemic Abscopal Effect of Low-dose Radiotherapy (2 Gy ×2) against Palatine Tonsil Follicular Lymphoma	Togitani K., Asagiri T., Iguchi M., Igawa T., Yoshino T., Kojima K.	10.2169/internalmedicine.8968-21
Trispecific T-cell engagers for dual tumor-targeting of colorectal cancer	Tapia-Galisteo A., Sánchez Rodríguez Í., Aguilar-Sopeña O., Harwood S.L., Narbona J., Ferreras Gu...	10.1080/2162402X.2022.2034355
Immune-based therapeutic strategies for acute myeloid leukemia	Böhme M., Kayser S.	10.3390/cancers14010105
Quantitative analysis of SARS-CoV-2 antibody status between patients with cancer and healthy individuals with extended vaccination dosing intervals in Canada	Robinson A., Mazurek A., Xu M., Gong Y.	10.3390/curroncol29010006
Humoral Immune Response in Hematooncological Patients and Health Care Workers Who Received SARS-CoV-2 Vaccinations	Mair M.J., Berger J.M., Berghoff A.S., Starzer A.M., Ortmayr G., Puhr H.C., Steindl A., Perkmann ...	10.1001/jamaoncol.2021.5437
Enhancing the immunogenicity of cancer vaccines by harnessing CLEC9A	Lahoud M.H., Radford K.J.	10.1080/21645515.2021.1873056
Prolonged viral positivity induced recurrent coronavirus disease 2019 (COVID-19) pneumonia in patients receiving anti-CD20 monoclonal antibody treatment Case reports	Deveci B., Saba R.	10.1097/MD.0000000000028470
Safety and efficacy of the mRNA BNT162b2 vaccine against SARS-CoV-2 in five groups of immunocompromised patients and healthy controls in a prospective open-label clinical trial	Bergman P., Blennow O., Hansson L., Mielke S., Nowak P., Chen P., Söderdahl G., Österborg A., Smi...	10.1016/j.ebiom.2021.103705
Tissue pathogens and cancers: A review of commonly seen manifestations in histo-and cytopathology	Chua T.H., Punjabi L.S., Khor L.Y.	10.3390/pathogens10111410
Subcutaneous immunoglobulin replacement for treatment of humoral immune dysfunction in patients with chronic lymphocytic leukemia	Mustafa S.S., Jamshed S., Vadamalai K., Ramsey A.	10.1371/journal.pone.0258529
Emerging RNA-Dependent RNA Polymerase Mutation in a Remdesivir-Treated B-cell Immunodeficient Patient with Protracted Coronavirus Disease 2019	Martinot M., Jary A., Fafi-Kremer S., Leducq V., Delagreverie H., Garnier M., Pacanowski J., Méki...	10.1093/cid/ciaa1474
Safety and Immunogenicity of the BNT162b2 mRNA COVID-19 Vaccine in Patients after Allogeneic HCT or CD19-based CART therapy—A Single-Center Prospective Cohort Study	Ram R., Hagin D., Kikozashvilli N., Freund T., Amit O., Bar-On Y., Beyar-Katz O., Shefer G., Mosh...	10.1016/j.jtct.2021.06.024
Identification of a class of non-conventional ER-stress-response-derived immunogenic peptides	Melacarne A., Ferrari V., Tiraboschi L., Mishto M., Liepe J., Aralla M., Marconato L., Lizier M.,...	10.1016/j.celrep.2021.109312
Anti-sars-cov-2 immune responses in patients receiving an allogeneic stem cell or organ transplant	Atanackovic D., Luetkens T., Avila S.V., Hardy N.M., Lutfi F., Sanchez-Petitto G., Mause E.V., Gl...	10.3390/vaccines9070737
A Novel HAGE/WT1-ImmunoBody® Vaccine Combination Enhances Anti-Tumour Responses When Compared to Either Vaccine Alone	Almshayakhchi R., Nagarajan D., Vadakekolathu J., Guinn B.-A., Reeder S., Brentville V., Metherin...	10.3389/fonc.2021.636977
Two patients with rituximab associated low gammaglobulin levels and relapsed covid-19 infections treated with convalescent plasma	Ormazabal Vélez I., Induráin Bermejo J., Espinoza Pérez J., Imaz Aguayo L., Delgado Ruiz M., Garc...	10.1016/j.transci.2021.103104
Short term results of vaccination with adjuvanted recombinant varicella zoster glycoprotein E during initial BTK inhibitor therapy for CLL or lymphoplasmacytic lymphoma	Zent C.S., Brady M.T., Delage C., Strawderman M., Laniewski N., Contant P.N., Kanagaiah P., Sangs...	10.1038/s41375-020-01074-4
Bacillus calmette–guérin immunotherapy for cancer	Cardillo F., Bonfim M., Sousa P.S.V., Mengel J., Castello-Branco L.R.R., Pinho R.T.	10.3390/vaccines9050439

Leukemia vaccine overcomes limitations of checkpoint blockade by evoking clonal T-cell responses in a murine acute myeloid leukemia model	Stroopinsky D., Liegel J., Bhasin M., Cheloni G., Thomas B., Bhasin S., Panchal R., Ghiasuddin H....	10.3324/haematol.2020.259457
Splice it up: Atypical transcripts to boost leukemia immunotherapy	Cieri N., Wu C.J.	10.1016/j.immuni.2021.03.016
Molecular and genetic biomarkers implemented from next-generation sequencing provide treatment insights in clinical practice for Waldenström macroglobulinemia	Wang Y., Gali V.L., Xu-Monette Z.Y., Sano D., Thomas S.K., Weber D.M., Zhu F., Fang X., Deng M., ...	10.1016/j.neo.2021.02.002
Phenotypical Characterization and Clinical Outcome of Canine Burkitt-Like Lymphoma	Aresu L., Agnoli C., Nicoletti A., Fanelli A., Martini V., Bertoni F., Marconato L.	10.3389/fvets.2021.647009
Subcutaneous immunoglobulins replacement therapy in secondary antibody deficiencies: Real life evidence as compared to primary antibody deficiencies	Cinetto F., Neri R., Vianello F., Visentin A., Barilà G., Gianese S., Lanciarotta A., Milito C., ...	10.1371/journal.pone.0247717
A multidisciplinary consensus on the morphological and functional responses to immunotherapy treatment	Leon-Mateos L., Garcia-Velloso M.J., García-Figueiras R., Rodriguez-Moreno J.F., Vercher-Conejero...	10.1007/s12094-020-02442-3
Acute Immune Signatures and Their Legacies in Severe Acute Respiratory Syndrome Coronavirus-2 Infected Cancer Patients	Abdul-Jawad S., Baù L., Alaguthurai T., del Molino del Barrio I., Laing A.G., Hayday T.S., Monin ...	10.1016/j.ccell.2021.01.001
Emerging immunotherapy for acute myeloid leukemia	Tabata R., Chi S., Yuda J., Minami Y.	10.3390/ijms22041944
Convalescent plasma-mediated resolution of COVID-19 in a patient with humoral immunodeficiency	Honjo K., Russell R.M., Li R., Liu W., Stoltz R., Tabengwa E.M., Hua Y., Prichard L., Kornbrust A...	10.1016/j.xcrm.2020.100164
Paradigms on immunotherapy combinations with chemotherapy	Salas-Benito D., Pérez-Gracia J.L., Ponz-Sarvisé M., Rodriguez-Ruiz M.E., Martínez-Forero I., Cas...	10.1158/2159-8290.CD-20-1312
Categorisation of patients based on immune profiles: a new approach to identifying candidates for response to checkpoint inhibitors	Bornschlegl S., Gustafson M.P., Delivanis D.A., Ryder M., Liu M.C., Vasmatzis G., Hallemeier C.L....	10.1002/cti2.1267
A combination of anti-pd-l1 treatment and therapeutic vaccination facilitates improved retroviral clearance via reactivation of highly exhausted t cells	Knuschke T., Kollenda S., Wenzek C., Zelinskyy G., Steinbach P., Dittmer U., Buer J., Epple M., W...	10.1128/mBio.02121-20
Noninvasive imaging of cancer immunotherapy	Abousaway O., Rakhshandehroo T., Van den Abbeele A.D., Kircher M.F., Rashidian M.	10.7150/ntno.50860
Endogenous retroviruses expressed in human tumours cannot be used as targets for anti-tumour vaccines	Denner J.	10.1016/j.tranon.2020.100941
Immunomodulatory effects of il-2 and il-15; implications for cancer immunotherapy	Yang Y., Lundqvist A.	10.3390/cancers12123586
Immunization against leukemia inhibitory factor and its receptor suppresses tumor formation of breast cancer initiating cells in BALB/c mouse	Ghanei Z., Mehri N., Jamshidizad A., Joupari M.D., Shamsara M.	10.1038/s41598-020-68158-0
Persistent COVID-19 in an immunocompromised patient temporarily responsive to two courses of remdesivir therapy	Helleberg M., Niemann C.U., Moestrup K.S., Kirk O., Lebech A.-M., Lane C., Lundgren J.	10.1093/infdis/jiaa446
Intercellular Adhesion Molecule-1 as Target for CAR-T-Cell Therapy of Triple-Negative Breast Cancer	Wei H., Wang Z., Kuang Y., Wu Z., Zhao S., Zhang Z., Li H., Zheng M., Zhang N., Long C., Guo W., ...	10.3389/fimmu.2020.573823
An effective peptide vaccine strategy circumventing clonal MHC heterogeneity of murine myeloid leukaemia	Shin A.-R., Lee S.-E., Choi H., Sohn H.-J., Cho H.-I., Kim T.-G.	10.1038/s41416-020-0955-y
Lack of myeloid cell infiltration as an acquired resistance strategy to immunotherapy	Beyranvand Nejad E., Labrie C., Abdulrahman Z., Van Elsas M.J., Rademaker E., Kleinovink J.W., Va...	10.1136/jitc-2020-001326
Inhibition of acute leukemia with attenuated Salmonella typhimurium strain VNP20009	Li M., Lu M., Lai Y., Zhang X., Li Y., Mao P., Liang Z., Mu Y., Lin Y., Zhao A.Z., Zhao Z., Zhou ...	10.1016/j.biopha.2020.110425
Autoimmune haemolytic anaemia secondary to influenza A in an allogeneic haematopoietic stem cell transplant recipient	Heath J.A., Lambeth M., Harrison J., Fleming J., Crighton G.	10.1111/jpc.14784
Preventing Varicella-zoster: Advances with the recombinant zoster vaccine	Ilyas S., Chandrasekar P.H.	10.1093/ofid/ofaa274
Immune escape and immunotherapy of acute myeloid leukemia	Vago L., Gojo I.	10.1172/JCI129204
Immunotherapy of multiple myeloma	Minnie S.A., Hill G.R.	10.1172/JCI129205
Clinical Cancer Advances 2020: Annual report on progress against cancer from the American Society of Clinical oncology	Markham M.J., Wachter K., Agarwal N., Bertagnolli M.M., Chang S.M., Dale W., Diefenbach C.S.M., R...	10.1200/JCO.19.03141
Therapeutic cancer vaccination with ex vivo rna-transfected dendritic cells—an update	Dörrie J., Schaft N., Schuler G., Schuler-Thurner B.	10.3390/pharmaceutics12020092
Epidemiology, management, and economic impact of acute myeloid leukemia and myelodysplastic syndrome in Spain at the hospital level: a claims database analysis	Marsà A., Ascanio M., Diaz-García J., Darbà J.	10.1080/13696998.2020.1840180
Toll-like receptor 7/8-matured RNA-transduced dendritic cells as post-remission therapy in acute myeloid leukaemia: results of a phase I trial	Lichtenegger F.S., Schnorfeil F.M., Rothe M., Deiser K., Altmann T., Bücklein V.L., Köhnke T., Au...	10.1002/cti2.1117
2020 AAFP Feline Retrovirus Testing and Management Guidelines	Little S., Levy J., Hartmann K., Hofmann-Lehmann R., Hosie M., Olah G., Denis K.S.	10.1177/1098612X19895940
Mitochondria-Targeting Immunogenic Cell Death Inducer Improves the Adoptive T-Cell Therapy Against Solid Tumor	Jiang Q., Zhang C., Wang H., Peng T., Zhang L., Wang Y., Han W., Shi C.	10.3389/fonc.2019.01196
Efficacy of cancer immunotherapy: An umbrella review of meta-analyses of randomized controlled trials	Kim J.Y., Lee K.H., Eisenhut M., van der Vliet H.J., Kronbichler A., Jeong G.H., Shin J.I., Gamer...	10.3390/cancers11111801
Novel biomarkers for personalized cancer immunotherapy	Shindo Y., Hazama S., Tsunedomi R., Suzuki N., Nagano H.	10.3390/cancers11091223
HPV vaccination knowledge, intentions, and practices among caregivers of childhood cancer survivors	Kirchhoff A.C., Mann K., Warner E.L., Kaddas H.K., Fair D., Fluchel M., Knackstedt E.D., Kepka D.	10.1080/21645515.2019.1619407
Chimeric antigen receptor T-cell therapy for acute myeloid leukemia: How close to reality?	Cummins K.D., Gill S.	10.3324/haematol.2018.208751

Adverse effects of chemotherapy and their management in Pediatric patients with Non-Hodgkin's Lymphoma in Kenya: A descriptive, situation analysis study	Opanga L., Mulaku M.N., Opanga S.A., Godman B., Kurdi A.	10.1080/14737140.2019.1606717
Dendritic cell-based immunotherapy of acute myeloid leukemia	Acker H.H.V., Versteven M., Lichtenegger F.S., Roex G., Campillo-Davo D., Lion E., Subklewe M., T...	10.3390/jcm8050579
Immunotherapy in endometrial cancer: New scenarios on the horizon	Di Tucci C., Capone C., Galati G., Iacobelli V., Schiavi M.C., Di Donato V., Muzii L., Panici P.B.	10.3802/jgo.2019.30.e46
A characterization of dendritic cells and their role in immunotherapy in glioblastoma: From preclinical studies to clinical trials	Srivastava S., Jackson C., Kim T., Choi J., Lim M.	10.3390/cancers11040537
Recent advances with Treg depleting fusion protein toxins for cancer immunotherapy	Kumar P., Kumar A., Parveen S., Murphy J.R., Bishai W.	10.2217/imt-2019-0060
Mutation-derived neoantigens for cancer immunotherapy	Castle J.C., Uduman M., Pabla S., Stein R.B., Buell J.S.	10.3389/fimmu.2019.01856
PKHB1 Tumor Cell Lysate Induces Antitumor Immune System Stimulation and Tumor Regression in Syngeneic Mice with Tumoral T Lymphoblasts	Martínez-Torres A.C., Calvillo-Rodríguez K.M., Uscanga-Palomeque A.C., Gómez-Morales L., Mendoza-...	10.1155/2019/9852361
Cytokines in the Treatment of Cancer	Conlon K.C., Miljkovic M.D., Waldmann T.A.	10.1089/jir.2018.0019
Engineering nanoparticles for targeted remodeling of the tumor microenvironment to improve cancer immunotherapy	Gao S., Yang D., Fang Y., Lin X., Jin X., Wang Q., Wang X., Ke L., Shi K.	10.7150/thno.29431
Stochastic modeling of tumor progression and immune evasion	George J.T., Levine H.	10.1016/j.jtbi.2018.09.012
Industry update: The latest developments in the field of therapeutic delivery, July 2018	Timmins P.	10.4155/tde-2018-0055
Extremely strong infiltration of WT1-specific CTLs into mouse tumor by the combination vaccine with WT1-specific CTL and helper peptides	Nakata J., Nakajima H., Hayashibara H., Imafuku K., Morimoto S., Fujiki F., Motooka D., Okuzaki D...	10.18632/oncotarget.26338
Selection of tumor-specific cytotoxic T lymphocytes in acute myeloid leukemia patients through the identification of T-cells capable to establish stable interactions with the leukemic cells: "Doublet technology"	García-Guerrero E., Sánchez-Abarca L.I., Domingo E., Ramos T.L., Bejarano-García J.A., Gonzalez-C...	10.3389/fimmu.2018.01971
Phosphoinositide 3-kinase δ inhibition promotes antitumor responses but antagonizes checkpoint inhibitors	Lim E.L., Cugliandolo F.M., Rosner D.R., Gyori D., Roychoudhuri R., Okkenhaug K.	10.1172/jci.insight.120626
Potential of genetically- modified measles virus as a treatment modality for carcinoma: A review	Kabilan A., Lakshmi, Priyadarshoni S.P.	10.13005/bpj/1448
Clinicopathological features and outcomes of pythiosis	Chitasombat M.N., Larbcharoensub N., Chindamporn A., Krajaejun T.	10.1016/j.ijid.2018.03.021
Advances in immunotherapy for acute myeloid leukemia	Przespolewski A., Szeles A., Wang E.S.	10.2217/fon-2017-0459
Phase 2 trial of a multivalent WT1 peptide vaccine (galinpepimut-S) in acute myeloid leukemia	Maslak P.G., Dao T., Bernal Y., Chanel S.M., Zhang R., Frattini M., Rosenblat T., Jurcic J.G., Br...	10.1182/bloodadvances.2017014175
KIR downregulation by IL-12/15/18 unleashes human NK cells from KIR/HLA-I inhibition and enhances killing of tumor cells	Ewen E.-M., Pahl J.H.W., Miller M., Watzl C., Cerwenka A.	10.1002/eji.201747128
Combination therapy with EpCAM-CAR-NK-92 cells and regorafenib against human colorectal cancer models	Zhang Q., Zhang H., Ding J., Liu H., Li H., Li H., Lu M., Miao Y., Li L., Zheng J.	10.1155/2018/4263520
Enhancement of Anti-Leukemia Immunity by Leukemia-Derived Exosomes Via Downregulation of TGF-β1 Expression	Huang F., Wan J., Hu W., Hao S.	10.1159/000484677
Wilms' Tumor Gene 1 (WT1) Peptide Vaccine Therapy for Hematological Malignancies: From CTL Epitope Identification to Recent Progress in Clinical Studies Including a Cure-Oriented Strategy	Oka Y., Tsuboi A., Nakata J., Nishida S., Hosen N., Kumanogoh A., Oji Y., Sugiyama H.	10.1159/000481353
Autosomal recessive agammaglobulinemia due to defect in μ heavy chain caused by a novel mutation in the IGHM gene	Silva P., Justicia A., Regueiro A., Farinã S., Couselo J.M., Loidi L.	10.1038/gene.2017.14
Impaired B cell immunity in acute myeloid leukemia patients after chemotherapy	Goswami M., Prince G., Biancotto A., Moir S., Kardava L., Santich B.H., Cheung F., Kotliarov Y., ...	10.1186/s12967-017-1252-2
Cancer immunology with a focus on understudied cancers as targets for immunotherapy	Young M.R.I.	10.3390/ijms18010127
Antibodies against Pneumococcal Capsular Polysaccharides and Natural Anti-Galactosyl (Alpha-Gal) in Patients with Humoral Immunodeficiencies	Kralickova P., Kuhnova J., Soucek O., Vodarek P., Zak P., Simkovic M., Motyckova M., Smolej L., M...	10.1155/2017/7304658
Excellent response to chemotherapy post immunotherapy	Dwary A.D., Master S., Patel A., Cole C., Mansour R., Mills G., Koshy N., Peddi P., Burton G., Ha...	10.18632/oncotarget.20030
Injecting nanoparticles into immunotherapy	Webb S.	10.2144/000114564
Treatments for hematologic malignancies in contrast to those for solid cancers are associated with reduced red cell alloimmunization	Evers D., Zwaginga J.J., Tijmensen J., Middelburg R.A., de Haas M., de Vooght K.M.K., van de Kerk...	10.3324/haematol.2016.152074
The society for immunotherapy of cancer consensus statement on immunotherapy for the treatment of hematologic malignancies: Multiple myeloma, lymphoma, and acute leukemia	Boyiadzis M., Bishop M.R., Abonour R., Anderson K.C., Ansell S.M., Avigan D., Barbarotta L., Barr...	10.1186/s40425-016-0188-z
Secondary antibody deficiency-causes and approach to diagnosis	Srivastava S., Wood P.	10.7861/clinmedicine.16-6-571
Intratumoral immunization by p19arf and interferon-β gene transfer in a heterotopic mouse model of lung carcinoma	Catani J.P.P., Medrano R.F.V., Hunger A., Valle P.D., Adjemian S., Zanatta D.B., Kroemer G., Cost...	10.1016/j.tranon.2016.09.011
Maintenance therapy in acute myeloid leukemia: An evidence-based review of randomized trials	Rashidi A., Walter R.B., Tallman M.S., Appelbaum F.R., DiPersio J.F.	10.1182/blood-2016-03-674127
Introduction: Cancer Immunology Special Issue-Immunotherapy	Kawakami Y.	10.1093/intimm/dxw028
Chimeric antigen receptor-modified T cells strike back	Frigault M.J., Maus M.V.	10.1093/intimm/dxw018
Heterologous vaccination and checkpoint blockade synergize to induce antileukemia immunity	Manlove L.S., Schenkel J.M., Manlove K.R., Pauken K.E., Williams R.T., Vezys V., Farrar M.A.	10.4049/jimmunol.1600130
Increased regulatory T cells in acute lymphoblastic leukaemia patients	Idris S.-Z., Hassan N., Lee L.-J., Md Noor S., Osman R., Abdul-Jalil M., Nordin A.-J., Abdullah M.	10.1080/10245332.2015.1101965

Cocktails for cancer with a measure of immunotherapy	Ledford H.	10.1038/532162a
The tumor-associated antigen RHAMM (HMMR/CD168) is expressed by monocyte-derived dendritic cells and presented to T cells	Willemen Y., Van den Bergh J.M.J., Bonte S.M., Anguille S., Heirman C., Stein B.M.H., Goossens H....	10.18632/oncotarget.12170
Luciferase mRNA Transfection of Antigen Presenting Cells Permits Sensitive Nonradioactive Measurement of Cellular and Humoral Cytotoxicity	Omokoko T.A., Luxemburger U., Bardissi S., Simon P., Utsch M., Breitkreuz A., Türeci Ö., Sahin U.	10.1155/2016/9540975
Novel targets for natural killer/T-cell lymphoma immunotherapy	Kumai T., Kobayashi H., Harabuchi Y.	10.2217/imt.15.103
Randomized, placebo-controlled, phase III trial of yeast- derIVed granulocyte-macrophage colony-stimulating factor (GM-CSF) versus peptide vaccination versus GM-CSF plus peptide vaccination versus placebo in patients with no evidence of disease after complete surgical resection of locally advance...	Lawson D.H., Lee S., Zhao F., Tarhini A.A., Margolin K.A., Ernstoff M.S., Atkins M.B., Cohen G.I....	10.1200/JCO.2015.62.0500
Combined cytotoxic activity of an infectious, but non-replicative herpes simplex virus type 1 and plasmacytoid dendritic cells against tumour cells	Thomann S., Boscheinen J.B., Vogel K., Knipe D.M., DeLuca N., Gross S., Schuler-Thurner B., Schus...	10.1111/imm.12509
The expanding role of immunopharmacology: IUPHAR Review 16	Tiligada E., Ishii M., Riccardi C., Spedding M., Simon H.-U., Teixeira M.M., Landys Chovel Cuervo...	10.1111/bph.13219
Autologous cellular vaccine overcomes cancer immunoediting in a mouse model of myeloma	Mazzocco M., Martini M., Rosato A., Stefani E., Matucci A., Dalla Santa S., De Sanctis F., Ugel S...	10.1111/imm.12477
Molecular pathways: Activating T cells after cancer cell phagocytosis from blockade of CD47 "Don't eat Me" signals	McCracken M.N., Cha A.C., Weissman I.L.	10.1158/1078-0432.CCR-14-2520
Update in lung cancer 2014	Spira A., Halmos B., Powell C.A.	10.1164/rccm.201504-0756UP
Target discovery for T cell therapy: Next steps to advance Immunotherapies	Bot A., Brewer J.E., Eshhar Z., Frankel S.R., Hickman E., Jungbluth A.A., Morgan R., Peretz Y., R...	10.1186/s40425-015-0061-5
Generation of mouse pluripotent stem cell-derived proliferating myeloid cells as an unlimited source of functional antigen-presenting cells	Zhang R., Liu T.-Y., Senju S., Haruta M., Hirosawa N., Suzuki M., Tatsumi M., Ueda N., Maki H., N...	10.1158/2326-6066.CIR-14-0117
Cytokine-induced killer cells: A novel immunotherapy strategy for leukemia	Yang X.-Y., Zeng H., Chen F.-P.	10.3892/ol.2014.2780
Applying extracellular vesicles based therapeutics in clinical trials - An ISEV position paper	Lener T., Gimona M., Aigner L., Börger V., Buzas E., Camussi G., Chaput N., Chatterjee D., Court ...	10.3402/jev.v4.30087
An adjuvanted whole cell vaccine as post-remission immunotherapy for acute leukemia	Weinkove R., Ancelet L.R., Gibbins J.D., Hermans I.F.	10.1080/2162402X.2014.995568
A microfluidic approach towards hybridoma generation for cancer immunotherapy	Lu Y.-T., Pendharkar G.P., Lu C.-H., Chang C.-M., Liu C.-H.	10.18632/oncotarget.5550
A novel cancer immunotherapy based on the combination of a synthetic carbohydrate-pulsed dendritic cell vaccine and glycoengineered cancer cells	Qiu L., Li J., Yu S., Wang Q., Li Y., Hu Z., Wu Q., Guo Z., Zhang J.	10.18632/oncotarget.2908
Targeting the tumor microenvironment to enhance antitumor immune responses	van der Jeught K., Bialkowski L., Daszkiewicz L., Broos K., Goyvaerts C., Renmans D., van Lint S....	10.18632/oncotarget.3204
Therapeutic uses of anti-PD-1 and anti-PD-L1 antibodies	Philips G.K., Atkins M.	10.1093/intimm/dxu095
Immunoglobulin G treatment of secondary immunodeficiencies in the era of novel therapies	Seppänen M.	10.1111/cei.12493
Entering the mainstream of cancer treatment	Rosenberg S.A.	10.1038/nrclinonc.2014.174
Clinical trials of dendritic cell-based cancer vaccines in hematologic malignancies	Pyzer A.R., Avigan D.E., Rosenblatt J.	10.4161/21645515.2014.982993
Adjuvants and myeloid-derived suppressor cells: Enemies or allies in therapeutic cancer vaccination	Fernández A., Oliver L., Alvarez R., Fernández L.E., Lee K.P., Mesa C.	10.4161/hv.29847
Engineering better immunotherapies via RNA interference	Sioud M.	10.4161/hv.29754
A Safety and Feasibility Study of an Allogeneic Colon Cancer Cell Vaccine Administered with a Granulocyte–Macrophage Colony Stimulating Factor–Producing Bystander Cell Line in Patients with Metastatic Colorectal Cancer	Zheng L., Edil B.H., Soares K.C., El-Shami K., Uram J.N., Judkins C., Zhang Z., Onners B., Laheru...	10.1245/s10434-014-3844-x
Lowest numbers of primary CD8+ T cells can reconstitute protective immunity upon adoptive immunotherapy	Stemberger C., Graef P., Odendahl M., Albrecht J., Dössinger G., Anderl F., Buchholz V.R., Gastei...	10.1182/blood-2013-12-547349
Stem cells and cancer immunotherapy: Arrowhead's 2nd annual cancer immunotherapy conference	Bot A., Chiriva-Internati M., Cornforth A., Czerniecki B.J., Ferrone S., Geles K., Greenberg P.D....	10.1186/2051-1426-2-6
Wilms' Tumour 1 (WT1) peptide vaccination in patients with acute myeloid leukaemia induces short-lived WT1-specific immune responses	Uttenthal B., Martinez-Davila I., Ivey A., Craddock C., Chen F., Virchis A., Kottaridis P., Grimw...	10.1111/bjh.12637
A new class of bispecific antibodies to redirect T cells for cancer immunotherapy	Rossi D.L., Rossi E.A., Cardillo T.M., Goldenberg D.M., Chang C.-H.	10.4161/mabs.27385
AACR Cancer Progress Report 2014	Arteaga C.L., Adamson P.C., Engelman J.A., Foti M., Gaynor R.B., Hilsenbeck S.G., Limburg P.J., L...	10.1158/1078-0432.CCR-14-2123
Classification of current anticancer immunotherapies	Galluzzi L., Vacchelli E., Bravo-San Pedro J.-M., Buqué A., Senovilla L., Baracco E.E., Bloy N., ...	10.18632/oncotarget.2998
Inflammation programs self-reactive CD8+ T cells to acquire T-box-mediated effector function but does not prevent deletional tolerance	Jackson S.R., Yuan J., Berrien-Elliott M.M., Chen C.L., Meyer J.M., Donlin M.J., Teague R.M.	10.1189/jlb.1A0913-500RR
Trial watch: Immunostimulatory cytokines in cancer therapy	Vacchelli E., Aranda F., Obrist F., Eggermont A., Galon J., Cremer I., Zitvogel L., Kroemer G., G...	10.4161/onci.29030
Infections caused by Mycobacterium tuberculosis in recipients of hematopoietic stem cell transplantation	Al-Anazi K.A., Al-Jasser A.M., Alsaleh K.	10.3389/fonc.2014.00231
Anti-leukemic potency of piggyBac-mediated CD19-specific T cells against refractory Philadelphia chromosome-positive acute lymphoblastic leukemia	Saito S., Nakazawa Y., Sueki A., Matsuda K., Tanaka M., Yanagisawa R., Maeda Y., Sato Y., Okabe S...	10.1016/j.jcyt.2014.05.022
PD-1 pathway inhibitors: Changing the landscape of cancer immunotherapy	Dolan D.E., Gupta S.	10.1177/107327481402100308
Cancer treatment: The killer within	Ledford H.	10.1038/508024a
Allogeneic tumor cell vaccines: The promise and limitations in clinical trials	Srivatsan S., Patel J.M., Bozeman E.N., Imasuen I.E., He S., Daniels D., Selvaraj P.	10.4161/hv.26568

Trends in translational medicine and drug targeting and delivery: New insights on an old concept - Targeted drug delivery with antibody-drug conjugates for cancers	Ho R.J.Y., Chien J.	10.1002/jps.23761
Peptide vaccines for hematological malignancies: a missed promise?	Bocchia, M and Defina, M and Aprile, L and Sicuranza, A	10.1007/s12185-013-1497-3
DC-based immunotherapy for hematological malignancies	Kitawaki, T	10.1007/s12185-013-1496-4
Biomimetic Glyconanoparticle Vaccine for Cancer Immunotherapy	Reuven, EM and Ben-Arye, SL and Yu, H and Duchi, R and Perota, A and Conchon, S and Abramovitch, SB and Soulillou, JP a	10.1021/acsnano.8b07241
Trained Immunity-Based Vaccine in B Cell Hematological Malignancies With Recurrent Infections: A New Therapeutic Approach	Ochoa-Grullon, J and Cuesta, CB and Fernandez, AG and Torres, GC and Lopez, CP and Cortijo, AP and Hall, LC and Morales	10.3389/fimmu.2020.611566
Reader-free ELISPOT assay for immuno-monitoring in peptide-based cancer vaccine immunotherapy	Hayashi, S and Imanishi, R and Adachi, M and Ikejima, S and Nakata, J and Morimoto, S and Fujiki, F and Nishida, S and Tsul	10.3892/br.2020.1289
Convalescent hyperimmune plasma for chemo-immunotherapy induced immunodeficiency in COVID-19 patients with hematological malignancies	Ferrari, S and Caprioli, C and Weber, A and Rambaldi, A and Lussana, F	10.1080/10428194.2021.1872070
ABO blood type correlates with survival on prostate cancer vaccine therapy	Muthana, SM and Gulley, JL and Hodge, JW and Schlom, J and Gildersleeve, JC	10.18632/oncotarget.4993
Patients with Hematological Malignancies Treated with T-Cell or B-Cell Immunotherapy Remain at High Risk of Severe Forms of COVID-19 in the Omicron Era	Zerbit, J and Detroit, M and Meyer, A and Decroocq, J and Deau-Fischer, B and Deschamps, P and Birsen, R and Mondesir, J a	10.3390/v14112377
Blood microbiota diversity determines response of advanced colorectal cancer to chemotherapy combined with adoptive T cell immunotherapy	Yang, D and Wang, XL and Zhou, XN and Zhao, J and Yang, HB and Wang, S and Morse, MA and Wu, JP and Yuan, YH and Li, S	10.1080/2162402X.2021.1976953
Preclinical efficacy and safety of the Ty21a vaccine strain for intravesical immunotherapy of non-muscle-invasive bladder cancer	Domingos-Pereira, S and Cesson, V and Chevalier, MF and Derre, L and Jichlinski, P and Nardelli-Haefliger, D	10.1080/2162402X.2016.1265720
A new peptide vaccine OCV-501: in vitro pharmacology and phase 1 study in patients with acute myeloid leukemia	Kobayashi, Y and Sakura, T and Miyawaki, S and Toga, K and Sogo, S and Heike, Y	10.1007/s00262-017-1981-3
Immunopeptidomics-Guided Warehouse Design for Peptide-Based Immunotherapy in Chronic Lymphocytic Leukemia	Nelde, A and Maringer, Y and Bilich, T and Salih, HR and Roerden, M and Heitmann, JS and Marcu, A and Bauer, J and Neider	10.3389/fimmu.2021.705974
Immunogenicity and Safety of the BNT162b2 mRNA COVID-19 Vaccine in Patients with Melanoma Treated with Immunotherapy	Diamantopoulos, PT and Kontandreopoulou, CN and Gkoufa, A and Solomou, E and Anastasopoulou, A and Palli, E and Kou	10.3390/cancers14153791
Extended evaluation of a phase 1/2 trial on dosing, safety, immunogenicity, and overall survival after immunizations with an advanced-generation Ad5 [E1-, E2b-]-CEA(6D) vaccine in late-stage colorectal cancer	Balint, JP and Gabitzsch, ES and Rice, A and Latchman, Y and Xu, YN and Messerschmidt, GL and Chaudhry, A and Morse, M	10.1007/s00262-015-1706-4
Immunotheranostic microbubbles (iMBs)-a modular platform for dendritic cell vaccine delivery applied to breast cancer immunotherapy	Jugniot, N and Dahl, JJ and Paulmurugan, R	10.1186/s13046-022-02501-3
TCR diversity - a universal cancer immunotherapy biomarker?	McNeel, DG	10.1186/s40425-016-0175-4
Engineering dendritic cell vaccines to improve cancer immunotherapy	Perez, CR and De Palma, M	10.1038/s41467-019-13368-y
Identification of immunogenic MAGED4B peptides for vaccine development in oral cancer immunotherapy	Lim, KP and Chun, NAL and Gan, CP and Teo, SH and Rahman, ZAA and Abraham, MT and Zain, RB and Ponniah, S and Cheo	10.4161/hv.29226
T/Tn immunotherapy avoiding immune deviation	Son, HY and Apostolopoulos, V and Kim, CW	10.1177/0394632016674018
Immunological effects of vaccines combined with granulocyte colony-stimulating factor on a murine WEHI-3 leukemia model	Chen, JQ and Zhang, ML and Zhou, FL and Wang, J and Niu, B and Zhang, WG	10.3892/ol.2017.5731
A combination trial of vaccine plus ipilimumab in metastatic castration-resistant prostate cancer patients: immune correlates	Jochems, C and Tucker, JA and Tsang, KY and Madan, RA and Dahut, WL and Liewehr, DJ and Steinberg, SM and Gulley, JL ar	10.1007/s00262-014-1524-0
Multi-Epitope-Based Vaccines for Colon Cancer Treatment and Prevention	Corulli, LR and Cecil, DL and Gad, E and Koehnlein, M and Coveler, AL and Childs, JS and Lubet, RA and Disis, ML	10.3389/fimmu.2021.729809
WT1 epitope-specific IgG and IgM antibodies for immune-monitoring in patients with advanced sarcoma treated with a WT1 peptide cancer vaccine	Alzaaqi, S and Naka, N and Hamada, K and Hosen, N and Kanegae, M and Outani, H and Adachi, M and Imanishi, R and Mori	10.3892/ol.2022.13184
A Randomized Phase II Trial of mFOLFOX6+Bevacizumab Alone or with AdCEA Vaccine plus Avelumab Immunotherapy for Untreated Metastatic Colorectal Cancer	Redman, JM and Tsai, YT and Weinberg, BA and Donahue, RN and Gandhy, S and Gatti-Mays, ME and Sater, HA and Bilusic, I	10.1093/oncolo/oyab046
Long-term outcomes of patients with recurrent ovarian cancer treated with a polyvalent vaccine with bevacizumab combination	Kahn, RM and Ragupathi, G and Zhou, QC and Iasonos, A and Kravetz, S and Hensley, ML and Konner, JA and Makker, V and	10.1007/s00262-022-03225-1
Increased indoleamine 2,3-dioxygenase activity and expression in prostate cancer following targeted immunotherapy	Zahm, CD and Johnson, LE and McNeel, DG	10.1007/s00262-019-02394-w
Enhancement of Anti-Leukemia Immunity by Leukemia-Derived Exosomes Via Downregulation of TGF-beta 1 Expression	Huang, F and Wan, JB and Hu, WW and Hao, SG	10.1159/000484677
Integrity of plasma DNA is inversely correlated with vaccine-induced antitumor immunity in ovarian cancer patients	Waki, K and Yokomizo, K and Kawano, K and Tsuda, N and Komatsu, N and Yamada, A	10.1007/s00262-020-02599-4
Analysis of peripheral blood immune cells after prophylactic immunization with HPV-16/18 ASO4-adjuvanted vaccine	Hus, I and Gonet-Sebastianka, J and Surdacka, A and Bojarska-Junak, A and Rolinski, J	10.5604/17322693.1150785
RBD- specific Th1 responses are associated with vaccine-induced protection against SARS-CoV-2 infection in patients with hematological malignancies	Bigenwald, C and Haddad, Y and Thelemaque, C and Carrier, A and Birebent, R and Ly, P and Flament, C and Lahmar, I and	10.1080/2162402X.2022.2163785

Neoantigen discovery and applications in glioblastoma: An immunotherapy perspective	Wang, C and Yu, MC and Zhang, W	10.1016/j.canlet.2022.215945
Norcantharidin enhances antitumor immunity of GM-CSF prostate cancer cells vaccine by inducing apoptosis of regulatory Tcells	Mo, LJ and Zhang, XJ and Shi, XJ and Wei, LL and Zheng, DP and Li, HW and Gao, JM and Li, JL and Hu, ZM	10.1111/cas.13639
A Neoantigen-Based Peptide Vaccine for Patients With Advanced Pancreatic Cancer Refractory to Standard Treatment	Chen, ZL and Zhang, SS and Han, N and Jiang, JH and Xu, YY and Ma, DY and Lu, LT and Guo, XJ and Qiu, M and Huang, QX ar	10.3389/fimmu.2021.691605
Circulating protein and antibody biomarker for personalized cancer immunotherapy	Yuan, JD	10.1186/s40425-016-0150-0
PD-1 directed immunotherapy alters Tfh and humoral immune responses to seasonal influenza vaccine	Herati, RS and Knorr, DA and Vella, LA and Silva, LV and Chilukuri, L and Apostolidis, SA and Huang, AC and Muselman, A an	10.1038/s41590-022-01274-3
Therapeutic immunization and local low-dose tumor irradiation, a reinforcing combination	Draghiciu, O and Walczak, M and Hoogeboom, BN and Franken, KLMC and Melief, KJM and Nijman, HW and Daemen, T	10.1002/ijc.28418
Avelumab in Men With Metastatic Castration-Resistant Prostate Cancer, Enriched for Patients Treated Previously With a Therapeutic Cancer Vaccine	Madan, RA and Redman, JM and Karzai, F and Dahut, WL and Cordes, L and Fakhrejahani, F and Vu, T and Sheikh, N and Sch	10.1097/CJI.0000000000000459
Silencing B7-H1 enhances the anti-tumor effect of bladder cancer antigen-loaded dendritic cell vaccine in vitro	Wang, S and Wang, YH and Liu, J and Shao, SX and Li, XJ and Gao, JN and Niu, HT and Wang, XS	10.2147/OTT.S65367
Hypomethylating agent alters the immune microenvironment in acute myeloid leukaemia (AML) and enhances the immunogenicity of a dendritic cell/AML vaccine	Nahas, MR and Stroopinsky, D and Rosenblatt, J and Cole, L and Pyzer, AR and Anastasiadou, E and Sergeeva, A and Ephrair	10.1111/bjh.15818
SITC cancer immunotherapy resource document: a compass in the land of biomarker discovery	Hu-Lieskovan, S and Bhaumik, S and Dhodapkar, K and Grivel, JCJB and Gupta, S and Hanks, BA and Janetzki, S and Kleen, T	10.1136/jitc-2020-000705
Predictive biomarkers and effectiveness of MUC1-targeted dendritic-cell-based vaccine in patients with refractory non-small cell lung cancer	Teramoto, K and Ozaki, Y and Hanaoka, J and Sawai, S and Tezuka, N and Fujino, S and Daigo, Y and Kontani, K	10.1177/1758834016678375
LPS and IL-8 activated umbilical cord blood-derived neutrophils inhibit the progression of ovarian cancer	Liu, Q and Yang, WH and Luo, N and Liu, J and Wu, YL and Ding, JY and Li, CX and Cheng, ZP	10.7150/jca.41035
Peripheral gene signatures reveal distinct cancer patient immunotypes with therapeutic implications for autologous DC-based vaccines	Hensler, M and Rakova, J and Kasikova, L and Lanickova, T and Pasulka, J and Holicek, P and Hraska, M and Hrnciarova, T ar	10.1080/2162402X.2022.2101596
Distinct transcriptional changes in non-small cell lung cancer patients associated with multi-antigenic RNActive (R) CV9201 immunotherapy	Hong, HS and Koch, SD and Scheel, B and Gnad-Vogt, U and Schroder, A and Kallen, KJ and Wiegand, V and Backert, L and K	10.1080/2162402X.2016.1249560
Identification of Neoantigens in Cancer Cells as Targets for Immunotherapy	Okada, M and Shimizu, K and Fujii, SI	10.3390/ijms23052594
A phase 1/2 study combining gemcitabine, Pegintron and p53 SLP vaccine in patients with platinum-resistant ovarian cancer	Dijkgraaf, EM and Santegoets, SJAM and Reyners, AKL and Goedemans, R and Nijman, HW and van Poelgeest, MIE and van E	10.18632/oncotarget.4772
Newcastle disease virus co-expressing interleukin 7 and interleukin 15 modified tumor cells as a vaccine for cancer immunotherapy	Xu, XJ and Sun, Q and Mei, Y and Liu, YH and Zhao, LX	10.1111/cas.13468
Exploiting B-cell Receptor Stereotypy to Design Tailored Immunotherapy in Chronic Lymphocytic Leukemia	Rovida, A and Maccalli, C and Scarfo, L and Dellabona, P and Stamatopoulos, K and Ghia, P	10.1158/1078-0432.CCR-20-1632
Phase I trial of a recombinant yeast-CEA vaccine (GI-6207) in adults with metastatic CEA-expressing carcinoma	Bilusic, M and Heery, CR and Arlen, PM and Rauckhorst, M and Apelian, D and Tsang, KY and Tucker, JA and Jochems, C and	10.1007/s00262-013-1505-8
Final results regarding the addition of dendritic cell vaccines to neoadjuvant chemotherapy in early HER2-negative breast cancer patients: clinical and translational analysis	Santisteban, M and Solans, BP and Hato, L and Urrizola, A and Mejias, LD and Salgado, E and Sanchez-Bayona, R and Toledo	10.1177/17588359211064653
A Hybrid Glioma Tumor Cell Lysate Immunotherapy Vaccine Demonstrates Good Clinical Efficacy in the Rat Model	Li, XL and Zeng, S and He, HP and Zeng, X and Peng, LL and Chen, LG	10.2147/OTT.S259516
A randomized, phase II trial of adjuvant immunotherapy with durable TKI-free survival in patients with chronic phase CML	Webster, JA and Robinson, TM and Blackford, AL and Warlick, E and Ferguson, A and Borrello, I and Zahurak, M and Jones, RJ	10.1016/j.leukres.2021.106737
Exosomes: Small vesicles with big roles in cancer, vaccine development, and therapeutics	Thakur, A and Parra, DC and Motallebnejad, P and Brocchi, M and Chen, HJ	10.1016/j.bioactmat.2021.08.029
Spherical Nucleic Acid Vaccine Structure Markedly Influences Adaptive Immune Responses of Clinically Utilized Prostate Cancer Targets	Teplensky, MH and Dittmar, JW and Qin, L and Wang, SY and Evangelopoulos, M and Zhang, B and Mirkin, CA	10.1002/adhm.202101262
Cellular Immunotherapy for Hematologic Malignancies: Beyond Bone Marrow Transplantation	Cirillo, M and Tan, P and Sturm, M and Cole, C	10.1016/j.bbmt.2017.10.035
A novel multi-epitope vaccine from MMSA-1 and DKK1 for multiple myeloma immunotherapy	Lu, CY and Meng, S and Jin, YX and Zhang, WG and Li, ZF and Wang, F and Wang-Johanning, F and Wei, YC and Liu, HL and Tu	10.1111/bjh.14686
Activated B lymphocytes and tumor cell lysate as an effective cellular cancer vaccine	Oxley, KL and Hanson, BM and Zani, AN and Bishop, GA	10.1007/s00262-021-02914-7
Immunotherapy with HDC/IL-2 may be clinically efficacious in acute myeloid leukemia of normal karyotype	Nilsson, MS and Hallner, A and Brune, M and Nilsson, S and Thoren, FB and Martner, A and Hellstrand, K	10.1080/21645515.2019.1636598
Clinical outcomes of a novel therapeutic vaccine with Tax peptide-pulsed dendritic cells for adult T cell leukaemia/lymphoma in a pilot study	Suehiro, Y and Hasegawa, A and Iino, T and Sasada, A and Watanabe, N and Matsuoka, M and Takamori, A and Tanosaki, R a	10.1111/bjh.13302
Enhanced antitumor activity of a novel, oral, helper epitope-containing WT1 protein vaccine in a model of murine leukemia	Minagawa, H and Hashii, Y and Nakajima, H and Fujiki, F and Morimoto, S and Nakata, J and Shirakawa, T and Katayama, T a	10.1186/s12885-023-10547-5
The human anti-CD40 agonist antibody mitazalimab (ADC-1013; JNJ-64457107) activates antigen-presenting cells, improves expansion of antigen-specific T cells, and enhances anti-tumor efficacy of a model cancer vaccine in vivo	Deronic, A and Nilsson, A and Thagesson, M and Werchau, D and Smith, KE and Ellmark, P	10.1007/s00262-021-02932-5

PD-1 Blockade Overcomes Adaptive Immune Resistance in Treatment with Anchored-GM-CSF Bladder Cancer Cells Vaccine	Zhang, XJ and Shi, XJ and Li, JL and Mo, LJ and Hu, ZM and Gao, JM and Wu, SH and Long, ZL	10.7150/jca.25423
PD-1 blockage combined with vaccine therapy can facilitate immune infiltration in tumor microenvironment of Lynch syndrome colon cancer	Ye, K and Zhong, WJ and Wang, PC and Chen, YX and Chi, P	10.3389/fgene.2022.877833
Identification and in vitro validation of neoantigens for immune activation against high-risk pediatric leukemia cells	Thakur, S and Jain, M and Zhang, CF and Major, C and Bielamowicz, KJ and Lacayo, NJ and Vaske, O and Lewis, V and Murgu	10.1080/21645515.2021.2001243
Enhanced immunogenicity of leukemia-derived exosomes via transfection with lentiviral vectors encoding costimulatory molecules	Hu, WW and Huang, F and Ning, LX and Hao, J and Wan, JB and Hao, SG	10.1007/s13402-020-00535-3
Immune responses to SARS-CoV-2 in vaccinated patients receiving checkpoint blockade immunotherapy for cancer	Piening, A and Ebert, E and Khojandi, N and Alspach, E and Teague, RM	10.3389/fimmu.2022.1022732
Modulation of bone morphogenic protein signaling in T-cells for cancer immunotherapy	Kuczma, M and Kurczewska, A and Kraj, P	10.3109/1547691X.2013.864736
Demarcated thresholds of tumor-specific CD8 T cells elicited by MCMV-based vaccine vectors provide robust correlates of protection	Nejad, EB and Ratts, RB and Panagioti, E and Meyer, C and Oduro, JD and Cicin-Sain, L and Fruh, K and van der Burg, SH and	10.1186/s40425-019-0500-9
Dendritic cell vaccines containing lymphocytes produce improved immunogenicity in patients with cancer	Frank, MO and Kaufman, J and Parveen, S and Blachere, NE and Orange, DE and Darnell, RB	10.1186/s12967-014-0338-3
Human CLEC9A antibodies deliver Wilms' tumor 1 (WT1) antigen to CD141(+)dendritic cells to activate naive and memory WT1-specific CD8(+)T cells	Pearson, FE and Tullett, KM and Leal-Rojas, IM and Haigh, OL and Masterman, KA and Walpole, C and Bridgeman, JS and Mc	10.1002/cti2.1141
Epigenetic Potentiation of NY-ESO-1 Vaccine Therapy in Human Ovarian Cancer	Odunsi, K and Matsuzaki, J and James, SR and Mhawech-Fauceglia, P and Tsuji, T and Miller, A and Zhang, W and Akers, SN a	10.1158/2326-6066.CIR-13-0126
Vaccine Strategy in Melanoma	Kwak, M and Leick, KM and Melssen, MM and Slingluff, CL	10.1016/j.soc.2019.02.003
A pilot study of an autologous tumor-derived autophagosome vaccine with docetaxel in patients with stage IV non-small cell lung cancer	Sanborn, RE and Ross, HJ and Aung, S and Acheson, A and Moudgil, T and Puri, S and Hilton, T and Fisher, B and Coffey, T an	10.1186/s40425-017-0306-6
Generated SecPen_NY-ESO-1_ubiquitin-pulsed dendritic cell cancer vaccine elicits stronger and specific T cell immune responses	Yang, YK and Guo, XH and Hu, B and He, P and Jiang, XW and Wang, ZH and Zhu, HX and Hu, LN and Yu, MH and Feng, MQ	10.1016/j.apsb.2020.08.004
Immune epitopes identification and designing of a multi-epitope vaccine against bovine leukemia virus: a molecular dynamics and immune simulation approaches	Samad, A and Meghla, NS and Nain, Z and Karpinski, TM and Rahman, MS	10.1007/s00262-022-03181-w
High Therapeutic Efficacy of a New Survivin LSP-Cancer Vaccine Containing CD4(+) and CD8(+)T-Cell Epitopes	Onodi, F and Maherzi-Mechalikh, C and Mougel, A and Ben Hamouda, N and Taboas, C and Gueugnon, F and Tran, T and No	10.3389/fonc.2018.00517
Efficacy and safety profile of COVID-19 vaccine in cancer patients: a prospective, multicenter cohort study	Yasin, AI and Aydin, SG and Sumbul, B and Koral, L and Simsek, M and Geredeli, C and Ozturk, A and Perkin, P and Demirtas	10.2217/fon-2021-1248
A novel cancer immunotherapy utilizing autologous tumour tissue	Park, H and Gladstone, M and Shanley, C and Goodrich, R and Guth, A	10.1111/vox.12935
Neoantigen-based EpiGVAX vaccine initiates antitumor immunity in colorectal cancer	Kim, VM and Pan, XY and Soares, KC and Azad, NS and Ahuja, N and Gamper, CJ and Blair, AB and Muth, S and Ding, D and L	10.1172/jci.insight.136368
Quantitative analysis and clonal characterization of T-cell receptor beta repertoires in patients with advanced non-small cell lung cancer treated with cancer vaccine	Mai, T and Takano, A and Suzuki, H and Hirose, T and Mori, T and Teramoto, K and Kiyotani, K and Nakamura, Y and Daigo, Y	10.3892/ol.2017.6125
Therapeutic Efficacy of Delta-Like Ligand 4 Gene Vaccine Overexpression on Liver Cancer in Mice	Yu, Y and Zhao, Y and Zhou, GM and Wang, X	10.1177/1533033820942205
Imaging activated T cells predicts response to cancer vaccines	Alam, IS and Mayer, AT and Sagiv-Barfi, I and Wang, KZ and Vermesh, O and Czerwinski, DK and Johnson, EM and James, ML	10.1172/JCI98509
Blocking IL-10 signalling at the time of immunization does not increase unwanted side effects in mice	Ni, GY and Liao, ZW and Chen, S and Wang, TF and Yuan, JW and Pan, X and Mounsey, K and Cavezza, S and Liu, XS and Wei	10.1186/s12865-017-0224-x
Exploratory open-label clinical study to determine the S-588410 cancer peptide vaccine-induced tumor-infiltrating lymphocytes and changes in the tumor microenvironment in esophageal cancer patients	Daiko, H and Marafioti, T and Fujiwara, T and Shirakawa, Y and Nakatsura, T and Kato, K and Puccio, I and Hikichi, T and Yos	10.1007/s00262-020-02619-3
Prophylactic vaccines are potent activators of monocyte-derived dendritic cells and drive effective anti-tumor responses in melanoma patients at the cost of toxicity	Bol, KF and Aarntzen, EHJG and Pots, JM and Nordkamp, MAMO and van de Rakt, MWMM and Scharenborg, NM and de Boer	10.1007/s00262-016-1796-7
HLA Ligand Atlas: a benign reference of HLA-presented peptides to improve T-cell-based cancer immunotherapy	Marcu, A and Bichmann, L and Kuchenbecker, L and Kowalewski, DJ and Freudenmann, LK and Backert, L and Muhlenbruch	10.1136/jitc-2020-002071
A poly-neoantigen DNA vaccine synergizes with PD-1 blockade to induce T cell-mediated tumor control	Tondini, E and Arakelian, T and Oosterhuis, K and Camps, M and van Duikeren, S and Han, WD and Arens, R and Zondag, G a	10.1080/2162402X.2019.1652539
Multiple antigen-engineered DC vaccines with or without IFN alpha to promote antitumor immunity in melanoma	Butterfield, LH and Vujanovic, L and Santos, PM and Maurer, DM and Gambotto, A and Lohr, J and Li, CL and Waldman, J and	10.1186/s40425-019-0552-x
Divergent clinical outcomes in a phase 2B trial of the TLPLDC vaccine in preventing melanoma recurrence and the impact of dendritic cell collection methodology: a randomized clinical trial	Adams, AM and Carpenter, EL and Clifton, GT and Vreeland, TJ and Chick, RC and O'Shea, AE and McCarthy, PM and Bohan,	10.1007/s00262-022-03272-8
Antigen-specific immunotherapies for acute myeloid leukemia	Buckley, SA and Walter, RB	10.1182/asheducation-2015.1.584
Immunotherapy for neuro-oncology: the critical rationale for combinatorial therapy	Reardon, DA and Gilbert, MR and Wick, W and Liau, LD	10.1093/neuonc/nov178
The impact of leukapheresis on immune-cell number and function in patients with advanced cancer	Gulley, JL and Marte, J and Heery, CR and Madan, RA and Steinberg, SM and Leitman, SF and Tsang, KY and Schlom, J	10.1007/s00262-015-1738-9
Predictive biomarkers for the efficacy of peptide vaccine treatment: based on the results of a phase II study on advanced pancreatic cancer	Shindo, Y and Hazama, S and Suzuki, N and Iguchi, H and Uesugi, K and Tanaka, H and Aruga, A and Hatori, T and Ishizaki, H	10.1186/s13046-017-0509-1

Integrity of circulating cell-free DNA as a prognostic biomarker for vaccine therapy in patients with nonsmall cell lung cancer	Waki, K and Yokomizo, K and Yoshiyama, K and Takamori, S and Komatsu, N and Yamada, A	10.1080/08923973.2021.1872619
Peptide vaccine immunotherapy biomarkers and response patterns in pediatric gliomas	Muller, S and Agnihotri, S and Shoger, KE and Myers, MI and Smith, N and Chaparala, S and Villanueva, CR and Chattopadhy	10.1172/jci.insight.98791
A New Plasmacytoid Dendritic Cell-Based Vaccine in Combination with Anti-PD-1 Expands the Tumor-Specific CD8+T Cells of Lung Cancer Patients	Hannani, D and Leplus, E and Laurin, D and Caulier, B and Aspord, C and Madelon, N and Bourova-Flin, E and Brambilla, C a	10.3390/ijms24031897
Integrity of plasma cell-free DNA as a prognostic factor for vaccine therapy in patients with endometrial cancer	Waki, K and Yokomizo, K and Kawano, K and Tsuda, N and Komatsu, N and Yamada, A	10.3892/mco.2020.2191
Blood-derived dendritic cell vaccinations induce immune responses that correlate with clinical outcome in patients with chemo-naive castration-resistant prostate cancer	Westdorp, H and Creemers, JHA and van Oort, IM and Schreibelt, G and Gorris, MAJ and Mehra, N and Simons, M and de Go	10.1186/s40425-019-0787-6
Hollow microneedle-mediated micro-injections of a liposomal HPV E7(43-63) synthetic long peptide vaccine for efficient induction of cytotoxic and T-helper responses	van der Maaden, K and Heuts, J and Camps, M and Pontier, M and van Scheltinga, AT and Jiskoot, W and Ossendorp, F and B	10.1016/j.jconrel.2017.11.035
Cancer Vaccines Based on Fluorine-Modified KH-1 Elicit Robust Immune Response	Liu, Y and Li, BH and Zheng, XJ and Xiong, DC and Ye, XS	10.3390/molecules28041934
Antitumor effect of oral cancer vaccine with Bifidobacterium delivering WT1 protein to gut immune system is superior to WT1 peptide vaccine	Shirakawa, T and Kitagawa, K	10.1080/21645515.2017.1382787
Society for Immunotherapy of Cancer (SITC) clinical practice guideline on immune effector cell-related adverse events	Maus, MV and Alexander, S and Bishop, MR and Brudno, JN and Callahan, C and Davila, ML and Diamonte, C and Dietrich, J	10.1136/jitc-2020-001511
Evaluation of safety and efficacy of p53MVA vaccine combined with pembrolizumab in patients with advanced solid cancers	Chung, V and Kos, FJ and Hardwick, N and Yuan, Y and Chao, J and Li, D and Waisman, J and Li, M and Zurcher, K and Franke	10.1007/s12094-018-1932-2
Evaluation of hGM-CSF/hTNF alpha surface-modified prostate cancer therapeutic vaccine in the huPBL-SCID chimeric mouse model	Lai, SH and Huang, ZY and Guo, YT and Cui, YQ and Wang, L and Ren, WF and Ying, FR and Gao, H and He, LX and Zhou, TL a	10.1186/s13045-015-0175-8
An implantable blood clot-based immune niche for enhanced cancer vaccination	Fan, Q and Ma, QL and Bai, JY and Xu, JL and Fei, ZY and Dong, ZL and Maruyama, A and Leong, KW and Liu, Z and Wang, C	10.1126/sciadv.abb4639
Combination of immune checkpoint blockade and targeted gene regulation of angiogenesis for facilitating antitumor immunotherapy	Zhan, J and Zhang, ML and Zhou, LL and He, C	10.3389/fbioe.2023.1065773
Checkpoint blockade cancer immunotherapy targets tumour-specific mutant antigens	Gubin, MM and Zhang, XL and Schuster, H and Caron, E and Ward, JP and Noguchi, T and Ivanova, Y and Hundal, J and Arthu	10.1038/nature13988
MicroRNA-6826 and-6875 in plasma are valuable non-invasive biomarkers that predict the efficacy of vaccine treatment against metastatic colorectal cancer	Kijima, T and Hazama, S and Tsunedomi, R and Tanaka, H and Takenouchi, H and Kanekiyo, S and Inoue, Y and Nakashima,	10.3892/or.2016.5267
Conjugation of TLR7 Agonist Combined with Demethylation Treatment Improves Whole-Cell Tumor Vaccine Potency in Acute Myeloid Leukemia	Zhong, GC and Jin, GY and Zeng, W and Yu, CH and Li, Y and Zhou, J and Zhang, L and Yu, L	10.7150/ijms.49983
Tumour burden and antigen-specific T cell magnitude represent major parameters for clinical response to cancer vaccine and TCR-engineered T cell therapy	Mallet, M and Boulos, RE and Alcazer, V and Bonaventura, P and Estornes, Y and Chuvin, N and Depil, S	10.1016/j.ejca.2022.05.008
Plasma-Activated Medium Potentiates the Immunogenicity of Tumor Cell Lysates for Dendritic Cell-Based Cancer Vaccines	Tomic, S and Petrovic, A and Puac, N and Skoro, N and Bekic, M and Petrovic, ZL and Colic, M	10.3390/cancers13071626
A novel allogeneic off-the-shelf dendritic cell vaccine for post-remission treatment of elderly patients with acute myeloid leukemia	van de Loosdrecht, AA and van Wetering, S and Santegoets, SJAM and Singh, SK and Eeltink, CM and den Hartog, Y and Kop	10.1007/s00262-018-2198-9
Single dose denileukin diftitox does not enhance vaccine-induced T cell responses or effectively deplete Tregs in advanced melanoma: immune monitoring and clinical results of a randomized phase II trial	Luke, JJ and Zha, YY and Matijevich, K and Gajewski, TF	10.1186/s40425-016-0140-2
Development of Dendritic Cell-Based Immunotherapy Targeting Tumor Blood Vessels in a Mouse Model of Lung Metastasis	Nomura, T and Yamakawa, M and Shimaoka, T and Hirai, T and Koizumi, N and Maruyama, K and Utoguchi, N	10.1248/bpb.b18-00737
Identification of human leukemia antigen A*0201-restricted epitopes derived from epidermal growth factor pathway substrate number 8	Tang, BS and Zhou, WJ and Du, JW and He, YJ and Li, YH	10.3892/mmr.2015.3673
Cationic Liposomes Loaded with a Synthetic Long Peptide and Poly(I:C): a Defined Adjuvanted Vaccine for Induction of Antigen-Specific T Cell Cytotoxicity	Varypataki, EM and van der Maaden, K and Bouwstra, J and Ossendorp, F and Jiskoot, W	10.1208/s12248-014-9686-4
Toll-like receptor 9 agonist enhances anti-tumor immunity and inhibits tumor-associated immunosuppressive cells numbers in a mouse cervical cancer model following recombinant lipoprotein therapy	Chang, LS and Leng, CH and Yeh, YC and Wu, CC and Chen, HW and Huang, HM and Liu, SJ	10.1186/1476-4598-13-60
Improved vaccine efficacy of tumor exosome compared to tumor lysate loaded dendritic cells in mice	Gu, XY and Erb, U and Buchler, MW and Zoller, M	10.1002/ijc.29100
Protective Cancer Vaccine Using Genetically Modified Hematopoietic Stem Cells	Xiong, XF and Das, JK and Song, JY and Ni, B and Ren, XC and Yang, JM and Song, JX	10.3390/vaccines6030040
Neoantigen-reactive T cells exhibit effective anti-tumor activity against colorectal cancer	Yu, YJ and Zhang, J and Ni, LY and Zhu, YS and Yu, HJ and Teng, YY and Lin, LM and Xue, ZX and Xue, XY and Shen, X and Song	10.1080/21645515.2021.1891814
Immunologic hierarchy, class II MHC promiscuity, and epitope spreading of a melanoma helper peptide vaccine	Hu, YN and Petroni, GR and Olson, WC and Czarkowski, A and Smolkin, ME and Grosh, WW and Chianese-Bullock, KA and S	10.1007/s00262-014-1551-x
Protocol for SARS-CoV-2 post-vaccine surveillance study in Australian adults and children with cancer: an observational study of safety and serological and immunological response to SARS-CoV-2 vaccination (SerOzNET)	Body, A and Ahern, E and Lal, L and Gillett, K and Abdulla, H and Opat, S and O'Brien, T and Downie, P and Turville, S and Mu	10.1186/s12879-021-07019-1

Therapeutic vaccination with 4-1BB co-stimulation eradicates mouse acute myeloid leukemia	Kerage, D and Soon, MSF and Doff, BL and Kobayashi, T and Nissen, MD and Lam, PY and Leggatt, GR and Mattarollo, SR	10.1080/2162402X.2018.1486952
HER-2/neu vaccine-primed autologous T-cell infusions for the treatment of advanced stage HER-2/neu expressing cancers	Disis, ML and Dang, YS and Coveler, AL and Marzbani, E and Kou, ZC and Childs, JS and Fintak, P and Higgins, DM and Reich	10.1007/s00262-013-1489-4
Antibody and Plasmablast Response to 13-Valent Pneumococcal Conjugate Vaccine in Chronic Lymphocytic Leukemia Patients - Preliminary Report	Pasiarski, M and Rolinski, J and Grywalska, E and Stelmach-Goldys, A and Korona-Glowniak, I and Gozdz, S and Hus, I and M	10.1371/journal.pone.0114966
Complete regression of cutaneous metastases with systemic immune response in a patient with triple negative breast cancer receiving p53MVA vaccine with pembrolizumab	Yuan, Y and Kos, FJ and He, TF and Yin, HWH and Li, MS and Hardwick, N and Zurcher, K and Schmolze, D and Lee, P and Pil	10.1080/2162402X.2017.1363138
Anti-SARS-CoV-2 cellular response after 2 and 3 doses of BNT162b2 mRNA vaccine in lymphoma patients receiving anti-CD20 antibodies	Gressens, SB and Wiedemann, A and Dechenaud, M and Dupuis, J and Gallien, S and Melica, G and Haioun, C and Lemonni	10.1016/j.vaccine.2023.01.064
OX40 Stimulation Enhances Protective Immune Responses Induced After Vaccination With Attenuated Malaria Parasites	Othman, AS and Franke-Fayard, BM and Imai, T and van der Gracht, ETI and Redeker, A and Salman, AM and Marin-Mogollor	10.3389/fcimb.2018.00247
A novel potential effective strategy for enhancing the antitumor immune response in breast cancer patients using a viable cancer cell-dendritic cell-based vaccine	Abdellateif, MS and Shaarawy, SM and Kandeel, EZ and El-Habashy, AH and Salem, ML and El-Houseini, ME	10.3892/ol.2018.8631
Combining a Universal Telomerase Based Cancer Vaccine With Ipilimumab in Patients With Metastatic Melanoma-Five-Year Follow Up of a Phase I/IIa Trial	Aamdal, E and Inderberg, EM and Ellingsen, EB and Rasch, W and Brunsvig, PF and Aamdal, S and Heintz, KM and Vodak, D a	10.3389/fimmu.2021.663865
Vaccination with human amniotic epithelial cells confer effective protection in a murine model of Colon adenocarcinoma	Tabatabaei, M and Mosaffa, N and Ghods, R and Nikoo, S and Kazemnejad, S and Khanmohammadi, M and Mirzadeghan, E	10.1002/ijc.31159
Mature Dendritic Cells May Promote High-Avidity Tuning of Vaccine T Cell Responses	Kumbhari, A and Egelston, CA and Lee, PP and Kim, PS	10.3389/fimmu.2020.584680
A TLR3-Specific Adjuvant Relieves Innate Resistance to PD-L1 Blockade without Cytokine Toxicity in Tumor Vaccine Immunotherapy	Takeda, Y and Kataoka, K and Yamagishi, J and Ogawa, S and Seya, T and Matsumoto, M	10.1016/j.celrep.2017.05.015
Phase I study of an active immunotherapy for asymptomatic phase Lymphoplasmacytic lymphoma with DNA vaccines encoding antigen-chemokine fusion: study protocol	Thomas, SK and Cha, SC and Smith, DL and Kim, KH and Parshottam, SR and Rao, S and Popescu, M and Lee, VY and Neela	10.1186/s12885-018-4094-2
Attenuated plasmodium sporozoite expressing MAGE-A3 induces antigen-specific CD8+T cell response against lung cancer in mice	Zhou, D and Zheng, H and Liu, QX and Lu, X and Deng, XF and Jiang, L and Hou, B and Fu, Y and Zhu, F and Ding, Y and Xu, W	10.20892/j.issn.2095-3941.2018.0309
Vaccine-Induced Intratumoral Lymphoid Aggregates Correlate with Survival Following Treatment with a Neoadjuvant and Adjuvant Vaccine in Patients with Resectable Pancreatic Adenocarcinoma	Zheng, L and Ding, D and Edil, BH and Judkins, C and Durham, JN and Thomas, DL and Bever, KM and Mo, GL and Solt, SE an	10.1158/1078-0432.CCR-20-2974
Anti-tumour effect of in situ vaccines combined with VEGFR inhibitors in the treatment of metastatic cervical cancer	Liu, LX and Cai, LY and Du, XD and Zhao, JD and Zhao, Y and Zou, CY and Yu, SS and Zhang, CH and Ye, PP and Su, XP and Ya	10.1016/j.intimp.2021.108302
CD4/CD8 ratio is a prognostic factor in IgG nonresponders among peptide vaccine-treated ovarian cancer patients	Waki, K and Kawano, K and Tsuda, N and Komatsu, N and Yamada, A	10.1111/cas.14349
Personalized cancer vaccine strategy elicits polyfunctional T cells and demonstrates clinical benefits in ovarian cancer	Tanyi, JL and Chiang, CLL and Chiffelle, J and Thierry, AC and Baumgartener, P and Huber, F and Goepfert, C and Tarussio, D	10.1038/s41541-021-00297-5
The Ovarian Cancer Chemokine Landscape Is Conducive to Homing of Vaccine-Primed and CD3/CD28-Costimulated T Cells Prepared for Adoptive Therapy	Zsiros, E and Duttagupta, P and Dangaj, D and Li, HZ and Frank, R and Garrabrant, T and Hagemann, IS and Levine, BL and Ju	10.1158/1078-0432.CCR-14-2777
Immunogenicity of ChAdOx1-nCoV-19 vaccine in solid malignancy patients by treatment regimen versus healthy controls: A prospective, multicenter observational study	Teeyapun, N and Luangdilok, S and Pakvisal, N and Sainamthip, P and Mingmalairak, S and Poovorawan, N and Sitthideatph	10.1016/j.eclinm.2022.101608
CD40-activated B cells induce anti-tumor immunity in vivo	Wennhold, K and Weber, TM and Klein-Gonzalez, N and Thelen, M and Garcia-Marquez, M and Chakupurakal, G and Fiedler,	10.18632/oncotarget.7720
Effective control of acute myeloid leukaemia and acute lymphoblastic leukaemia progression by telomerase specific adoptive T-cell therapy	Sandri, S and De Sanctis, F and Lamolinara, A and Boschi, F and Poffe, O and Trovato, R and Fiore, A and Sartori, S and Sbarl	10.18632/oncotarget.18115
Generating Peripheral Blood Derived Lymphocytes Reacting Against Autologous Primary AML Blasts	Mehta, RS and Chen, XH and Antony, J and Boyiadzis, M and Szabolcs, P	10.1097/CJI.0000000000000107
Anti-PD-L1/PD-L2 therapeutic vaccination in untreated chronic lymphocytic leukemia patients with unmutated IgHV	Klausen, U and Grauslund, JH and Jorgensen, NGD and Ahmad, SM and Jonassen, M and Weis-Banke, SE and Martinenaite,	10.3389/fonc.2022.1023015
Rapid tumor regression in an Asian lung cancer patient following personalized neo-epitope peptide vaccination	Li, FG and Chen, CX and Ju, T and Gao, JQ and Yan, J and Wang, P and Xu, Q and Hwu, P and Du, XM and Lizee, G	10.1080/2162402X.2016.1238539
Vaccine effectiveness against COVID-19 breakthrough infections in patients with cancer (UKCCEP): a population-based test-negative case-control study	Lee, LYW and Starkey, T and Ionescu, MC and Little, M and Tilby, M and Tripathy, AR and Mckenzie, HS and Al-Hajji, Y and Ba	10.1016/S1470-2045(22)00202-9
Association of Systemic Inflammatory and Immune Indices With Survival in Canine Patients With Oral Melanoma, Treated With Experimental Immunotherapy Alone or Experimental Immunotherapy Plus Metronomic Chemotherapy	Garcia, JS and Nowosh, V and Lopez, RVM and Massoco, CD	10.3389/fvets.2022.888411
Analyses of Pretherapy Peripheral Immunoscore and Response to Vaccine Therapy	Farsaci, B and Donahue, RN and Grenga, I and Lepone, LM and Kim, PS and Dempsey, B and Siebert, JC and Ibrahim, NK and	10.1158/2326-6066.CIR-16-0037
Identificaiton of Novel Immunogenic Human Papillomavirus Type 16 E7-Specific Epitopes Restricted to HLA-A*33;03 for Cervical Cancer Immunotherapy	Kim, S and Chung, HW and Kong, HY and Lim, JB	10.3349/ymj.2017.58.1.43
Laser-facilitated epicutaneous immunotherapy with depigmented house dust mite extract alleviates allergic responses in a mouse model of allergic lung inflammation	Korotchenko, E and Moya, R and Scheiblhofer, S and Joubert, IA and Horejs-Hoeck, J and Hauser, M and Calzada, D and Irao	10.1111/all.14164

Autologous hybrid cell fusion vaccine in a spontaneous intermediate model of breast carcinoma	Bird, RC and DeInnocentes, P and Bird, AEC and Kabir, FML and Martinez-Romero, EG and Smith, AN and Smith, BF	10.4142/jvs.2019.20.e48
Identification by digital immunohistochemistry of intratumoral changes of immune infiltrates after vaccine in the absence of modifications of PBMC immune cell subsets	Farsaci, B and Jochems, C and Grenga, I and Donahue, RN and Tucker, JA and Pinto, PA and Merino, MJ and Heery, CR and M	10.1002/ijc.28743
A double-blind randomized comparative clinical trial to evaluate the safety and efficacy of dendritic cell vaccine loaded with WT1 peptides (TLP0-001) in combination with S-1 in patients with advanced pancreatic cancer refractory to standard chemotherapy	Katsuda, M and Miyazawa, M and Ojima, T and Katanuma, A and Hakamada, K and Sudo, K and Asahara, S and Endo, I and U	10.1186/s13063-019-3332-5
T-lymphocyte homing: an underappreciated yet critical hurdle for successful cancer immunotherapy	Sackstein, R and Schatton, T and Barthel, SR	10.1038/labinvest.2017.25
Phase 1/2 study of the WT1 peptide cancer vaccine WT4869 in patients with myelodysplastic syndrome	Ueda, Y and Ogura, M and Miyakoshi, S and Suzuki, T and Heike, Y and Tagashira, S and Tsuchiya, S and Ohyashiki, K and Mi	10.1111/cas.13409
Blocking antibodies induced by immunization with a hypoallergenic parvalbumin mutant reduce allergic symptoms in a mouse model of fish allergy	Freidl, R and Gstoettner, A and Baranyi, U and Swoboda, I and Stolz, F and Focke-Tejkl, M and Wekerle, T and van Ree, R and	10.1016/j.jaci.2016.10.018
Improving immunotherapy for colorectal cancer using dendritic cells combined with anti-programmed death-ligand in vitro	Hu, ZL and Ma, Y and Shang, ZY and Hu, SD and Liang, K and Liang, WT and Xing, XW and Wang, YF and Du, XH	10.3892/ol.2018.7978
Polymer Nanoparticle-Mediated Delivery of Oxidized Tumor Lysate-Based Cancer Vaccines	Berti, C and Graciotti, M and Boarino, A and Yakkala, C and Kandalaft, LE and Klok, HA	10.1002/mabi.202100356
Wilms Tumor Gene (WT1) Peptide- based Cancer Vaccine Combined With Gemcitabine for Patients With Advanced Pancreatic Cancer	Nishida, S and Koido, S and Takeda, Y and Homma, S and Komita, H and Takahara, A and Morita, S and Ito, T and Morimoto,	10.1097/CJI.0000000000000020
CD56(dim) CD16(-) Natural Killer Cell Profiling in Melanoma Patients Receiving a Cancer Vaccine and Interferon-alpha	Vujanovic, L and Chuckran, C and Lin, Y and Ding, F and Sander, CA and Santos, PM and Lohr, J and Mashadi-Hossein, A and	10.3389/fimmu.2019.00014
Case report: Long-term survival of a pancreatic cancer patient immunized with an SVN-2B peptide vaccine	Shima, H and Kutomi, G and Satomi, F and Imamura, M and Kimura, Y and Mizuguchi, T and Watanabe, K and Takahashi, A a	10.1007/s00262-018-2217-x
Immunotherapy Targeting HPV16/18 Generates Potent Immune Responses in HPV-Associated Head and Neck Cancer	Aggarwal, C and Cohen, RB and Morrow, MP and Kraynyak, KA and Sylvester, AJ and Knoblock, DM and Bauml, JM and Weins	10.1158/1078-0432.CCR-18-1763
Induction of Immune Response after Allogeneic Wilms' Tumor 1 Dendritic Cell Vaccination and Donor Lymphocyte Infusion in Patients with Hematologic Malignancies and Post-Transplantation Relapse	Shah, NN and Loeb, DM and Khuu, H and Stroncek, D and Ariyo, T and Raffeld, M and Delbrook, C and Mackall, CL and Wayr	10.1016/j.bbmt.2016.08.028
Alphavirus Replicon Particle Vaccine Breaks B Cell Tolerance and Rapidly Induces IgG to Murine Hematolymphoid Tumor Associated Antigens	Su, H and Imai, K and Jia, W and Li, ZG and DiCioccio, RA and Serody, JS and Poe, JC and Chen, BJ and Doan, PL and Sarantc	10.3389/fimmu.2022.865486
Rapid expansion in the WAVE bioreactor of clinical scale cells for tumor immunotherapy	Meng, YM and Sun, J and Hu, TT and Ma, YS and Du, TZ and Kong, CC and Zhang, GR and Yu, T and Piao, HZ	10.1080/21645515.2018.1480241
Idiotypic DNA vaccination for the treatment of multiple myeloma: safety and immunogenicity in a phase I clinical study	McCann, KJ and Godeseth, R and Chudley, L and Mander, A and Di Genova, G and Lloyd-Evans, P and Kerr, JP and Malykh, V	10.1007/s00262-015-1703-7
Combined immunotherapy encompassing intratumoral poly-ICLC, dendritic-cell vaccination and radiotherapy in advanced cancer patients	Rodriguez-Ruiz, ME and Perez-Gracia, JL and Rodriguez, I and Alfaro, C and Onate, C and Perez, G and Gil-Bazo, I and Benito	10.1093/annonc/mdy089
Immunotherapies for Pediatric Solid Tumors: A Targeted Update	Gupta, A and Cripe, TP	10.1007/s40272-021-00482-y
Blood Eosinophilia Is an on-Treatment Biomarker in Patients with Solid Tumors Undergoing Dendritic Cell Vaccination with Autologous Tumor-RNA	Moreira, A and Erdmann, M and Uslu, U and Vass, V and Schuler, G and Schuler-Thurner, B	10.3390/pharmaceutics12030210
Expression of Lymphocyte-Activation Gene 3 (LAG-3) Immune Checkpoint Receptor Identifies a Tumor-Reactive T Cell Population in the Peripheral Blood of Patients with Colorectal Cancer	Huang, LF and Qiao, GL and Wu, JP and Ren, J	10.12659/MSM.915741
Functional immune responses against SARS-CoV-2 variants of concern after fourth COVID-19 vaccine dose or infection in patients with blood cancer	Fendler, A and Shepherd, STC and Au, L and Wu, M and Harvey, R and Wilkinson, KA and Schmitt, AM and Tippu, Z and Shum	10.1016/j.xcrm.2022.100781
Programmed death-1 blockade enhances the antitumor effects of peptide vaccine-induced peptide-specific cytotoxic T lymphocytes	Sawada, Y and Yoshikawa, T and Shimomura, M and Iwama, T and Endo, I and Nakatsura, T	10.3892/ijo.2014.2737
Therapeutic Vaccines against Hepatocellular Carcinoma in the Immune Checkpoint Inhibitor Era: Time for Neoantigens?	Reparaz, D and Aparicio, B and Llopiz, D and Hervas-Stubbs, S and Sarobe, P	10.3390/ijms23042022
Gold Glyconanoparticles Combined with 91-99 Peptide of the Bacterial Toxin, Listeriolysin O, Are Efficient Immunotherapies in Experimental Bladder Tumors	Teran-Navarro, H and Zeoli, A and Salines-Cuevas, D and Marradi, M and Montoya, N and Gonzalez-Lopez, E and Ocejo-Viny	10.3390/cancers14102413
Assessing Neutralized Nicotine Distribution Using Mice Vaccinated with the Mucosal Conjugate Nicotine Vaccine	Fraleigh, NL and Lewicky, JD and Martel, AL and Diaz-Mitoma, F and Le, HT	10.3390/vaccines9020118
Senescent cancer cell-derived nanovesicle as a personalized therapeutic cancer vaccine	Hong, JHY and Jung, MY and Kim, C and Kang, MKY and Go, S and Sohn, H and Moon, S and Kwon, S and Song, SY and Kim, E	10.1038/s12276-023-00951-z
PAD-2-mediated citrullination of nucleophosmin provides an effective target for tumor immunotherapy	Choudhury, RH and Symonds, P and Paston, SJ and Daniels, I and Cook, KW and Gijon, M and Metheringham, RL and Brentv	10.1136/jitc-2021-003526
Cationic synthetic long peptides-loaded nanogels: An efficient therapeutic vaccine formulation for induction of T-cell responses	Kordalivand, N and Tondini, E and Lau, CYJ and Vermonden, T and Mastrobattista, E and Hennink, WE and Ossendorp, F and	10.1016/j.jconrel.2019.10.048
Improving cancer immunotherapy using nanomedicines: progress, opportunities and challenges	Martin, JD and Cabral, H and Stylianopoulos, T and Jain, RK	10.1038/s41571-019-0308-z
Clinical Implications of CD4(+)CD25(+)Foxp3(+)Regulatory T Cell Frequencies After CHP-MAGE-A4 Cancer Vaccination	Wada, M and Tsuchikawa, T and Kyogoku, N and Abiko, T and Miyauchi, K and Takeuchi, S and Kuwatani, T and Shichinohe, T	10.21873/anticanres.12368
Tumor immunology and cancer immunotherapy: summary of the 2014 SITC primer	Page, DB and Bourla, AB and Daniyan, A and Naidoo, J and Smith, E and Smith, M and Friedman, C and Khalil, DN and Funt,	10.1186/s40425-015-0072-2

Safety and immunogenicity of novel 5T4 viral vectored vaccination regimens in early stage prostate cancer: a phase I clinical trial	Cappuccini, F and Bryant, R and Pollock, E and Carter, L and Verrill, C and Hollidge, J and Poulton, I and Baker, M and Mittor	10.1136/jitc-2020-000928
Durable and dynamic hTERT immune responses following vaccination with the long-peptide cancer vaccine UV1: long-term follow-up of three phase I clinical trials	Ellingsen, EB and Aamdal, E and Guren, T and Lilleby, W and Brunsvig, PF and Mangsbo, SM and Aamdal, S and Hovig, E and	10.1136/jitc-2021-004345
Establishing the pig as a large animal model for vaccine development against human cancer	Overgaard, NH and Frosig, TM and Welner, S and Rasmussen, M and Ilsoe, M and Sorensen, MR and Andersen, MH and Buus	10.3389/fgene.2015.00286
Vaccination against PD-L1 with IO103 a Novel Immune Modulatory Vaccine in Basal Cell Carcinoma: A Phase IIa Study	Jorgensen, NG and Kaae, J and Grauslund, JH and Met, O and Nielsen, SL and Pedersen, AW and Svane, IM and Ehrnrooth, E	10.3390/cancers13040911
Role of Immunomodulation of BCG Therapy on AML Remission	Kennedy, A and Sahu, KK and Cerny, J	10.2147/IMCRJ.S296387
Antitumor Effects of DC Vaccine With ALA-PDT-Induced Immunogenic Apoptotic Cells for Skin Squamous Cell Carcinoma Mice	Zhang, HY and Wang, PR and Wang, XJ and Shi, L and Fan, ZX and Zhang, GL and Yang, DG and Bahavar, CF and Zhou, FF and	10.1177/1533033818785275
A pilot trial of vaccination with Carcinoembryonic antigen and Her2/neu peptides in advanced colorectal cancer	Lynch, KT and Squeo, GC and Kane, WJ and Meneveau, MO and Petroni, G and Olson, WC and Chianese-Bullock, KA and Sli	10.1002/ijc.33793
Toxicity evaluation of ConvitVax breast cancer immunotherapy	Duarte, MA and Carballo, JM and De Gouveia, YM and Garcia, A and Ruiz, D and Gledhill, T and Gonzalez-Marcano, E and Co	10.1038/s41598-021-91995-6
Safety and immunologic correlates of Melanoma GVAX, a GM-CSF secreting allogeneic melanoma cell vaccine administered in the adjuvant setting	Lipson, EJ and Sharfman, WH and Chen, SM and McMiller, TL and Pritchard, TS and Salas, JT and Sartorius-Mergenthaler, S a	10.1186/s12967-015-0572-3
Sarcosine promotes trafficking of dendritic cells and improves efficacy of anti-tumor dendritic cell vaccines via CXC chemokine family signaling	Dastmalchi, F and Karachi, A and Yang, CL and Azari, H and Sayour, EJ and Dechkovskaia, A and Vlasak, AL and Saia, ME and	10.1186/s40425-019-0809-4
Human heat shock protein-specific cytotoxic T lymphocytes display potent antitumour immunity in multiple myeloma	Li, R and Qian, JF and Zhang, WH and Fu, WJ and Du, J and Jiang, H and Zhang, H and Zhang, CY and Xi, H and Yi, Q and Hou,	10.1111/bjh.12943
Comparative immunological studies of tumor-associated Lewis X, Lewis Y, and KH-1 antigens	Guo, JT and Jiang, WJ and Li, QJ and Jaiswal, M and Guo, ZW	10.1016/j.carres.2020.107999
Silencing of Foxp3 enhances the antitumor efficacy of GM-CSF genetically modified tumor cell vaccine against B16 melanoma	Miguel, A and Sendra, L and Noe, V and Ciudad, CJ and Dasi, F and Hervas, D and Herrero, MJ and Alino, SF	10.2147/OTT.S104393
Remodeling the fibrotic tumor microenvironment of desmoplastic melanoma to facilitate vaccine immunotherapy	Zhu, HD and Liu, Q and Miao, L and Musetti, S and Huo, MR and Huang, L	10.1039/c9nr09610h
RAMIHM generates fully human monoclonal antibodies by rapid mRNA immunization of humanized mice and BCR-seq	Ren, P and Peng, L and Yang, LJ and Suzuki, K and Fang, ZH and Renauer, PA and Lin, QQ and Bai, MZ and Li, TQ and Clark, P	10.1016/j.chembiol.2022.12.005
Identification of T Cell Receptors Targeting a Neoantigen Derived from Recurrently Mutated FGFR3	Tate, T and Matsumoto, S and Nemoto, K and Leisegang, M and Nagayama, S and Obama, K and Nakamura, Y and Kiyotani,	10.3390/cancers15041031
Peptide vaccine targeting mutated GNAS: a potential novel treatment for pseudomyxoma peritonei	Flatmark, K and Torgunrud, A and Fleten, KG and Davidson, B and Juul, HV and Mensali, N and Lund-Andersen, C and Inderb	10.1136/jitc-2021-003109
Durable complete response to neoantigen-loaded dendritic-cell vaccine following anti-PD-1 therapy in metastatic gastric cancer	Guo, ZQ and Yuan, Y and Chen, C and Lin, J and Ma, QW and Liu, G and Gao, Y and Huang, Y and Chen, L and Chen, LZ and H	10.1038/s41698-022-00279-3
Intratumoral IFN-gamma or topical TLR7 agonist promotes infiltration of melanoma metastases by T lymphocytes expanded in the blood after cancer vaccine	Tran, CA and Lynch, KT and Meneveau, MO and Katyal, P and Olson, WC and Slingluff, CL	10.1136/jitc-2022-005952
A Pan-cancer Clinical Study of Personalized Neoantigen Vaccine Monotherapy in Treating Patients with Various Types of Advanced Solid Tumors	Fang, Y and Mo, F and Shou, JW and Wang, HM and Luo, K and Zhang, SS and Han, N and Li, HS and Ye, SL and Zhou, Z and C	10.1158/1078-0432.CCR-19-2881
HLA ligandome analysis identifies the underlying specificities of spontaneous antileukemia immune responses in chronic lymphocytic leukemia (CLL)	Kowalewski, DJ and Schuster, H and Backert, L and Berlin, C and Kahn, S and Kanz, L and Salih, HR and Rammensee, HG an	10.1073/pnas.1416389112
Immunogenic FEAT protein circulates in the bloodstream of cancer patients	Li, Y and Kobayashi, K and Mona, MM and Satomi, C and Okano, S and Inoue, H and Tani, K and Takahashi, A	10.1186/s12967-016-1034-2
Reprogramming Immune Response With Capsid-Optimized AAV6 Vectors for Immunotherapy of Cancer	Pandya, M and Britt, K and Hoffman, B and Ling, C and Aslanidi, GV	10.1097/CJI.0000000000000093
Tumor Cells Modified with Newcastle Disease Virus Expressing IL-24 as a Cancer Vaccine	Xu, XJ and Yi, C and Yang, XQ and Xu, JW and Sun, Q and Liu, YH and Zhao, LX	10.1016/j.omto.2019.06.001
Targeting the tumor mutanome for personalized vaccination in a TMB low non-small cell lung cancer	McCann, K and von Witzleben, A and Thomas, J and Wang, C and Wood, O and Singh, D and Boukas, K and Bendjama, K and	10.1136/jitc-2021-003821
Anti-tumor effect of the alphavirus-based virus-like particle vector expressing prostate-specific antigen in a HLA-DR transgenic mouse model of prostate cancer	Riabov, V and Tretyakova, I and Alexander, RB and Pushko, P and Klyushnenkova, EN	10.1016/j.vaccine.2015.08.062
In vitro and in vivo imaging of initial B-T-cell interactions in the setting of B-cell based cancer immunotherapy	Gonzalez, NK and Wennhold, K and Balkow, S and Kondo, E and Bolck, B and Weber, T and Garcia-Marquez, M and Grabbe,	10.1080/2162402X.2015.1038684
Generation of GM-CSF-producing antigen-presenting cells that induce a cytotoxic T cell-mediated antitumor response	Mashima, H and Zhang, R and Kobayashi, T and Hagiya, Y and Tsukamoto, H and Liu, TY and Iwamaa, T and Yamamoto, M ar	10.1080/2162402X.2020.1814620
Palm Tocotrienol-Adjuvanted Dendritic Cells Decrease Expression of the SATB1 Gene in Murine Breast Cancer Cells and Tissues	Hafid, SRA and Radhakrishnan, AK	10.3390/vaccines7040198
Effect of Different Bacillus Calmette-Guerin Substrains on Growth Inhibition of T24 Bladder Cancer Cells and Cytokines Secretion by BCG Activated Peripheral Blood Mononuclear Cells of PBMCs	Janaszek-Seydlitz, W and Prygiel, M and Bucholc, B and Wiatrzyk, A and Czajka, U and Gorska, P and Soliwoda, U	10.17219/acem/37330
T cell receptor repertoire features associated with survival in immunotherapy-treated pancreatic ductal adenocarcinoma	Hopkins, AC and Yarchoan, M and Durham, JN and Yusko, EC and Rytlewski, JA and Robins, HS and Laheru, DA and Le, DT ar	10.1172/jci.insight.122092

A phase I clinical study of immunotherapy for advanced colorectal cancers using carcinoembryonic antigen-pulsed dendritic cells mixed with tetanus toxoid and subsequent IL-2 treatment	Liu, KJ and Chao, TY and Chang, JY and Cheng, AL and Ch'ang, HJ and Kao, WY and Wu, YC and Yu, WL and Chung, TR and W	10.1186/s12929-016-0279-7
Streptavidin: A Novel Immunostimulant for the Selection and Delivery of Autologous and Syngeneic Tumor Vaccines	Weir, C and Hudson, AL and Moon, E and Ross, A and Alexander, M and Peters, L and Langova, V and Clarke, SJ and Pavlakis	10.1158/2326-6066.CIR-13-0157
Novel antigens in non-small cell lung cancer: SP17, AKAP4, and PTTG1 are potential immunotherapeutic targets	Mirandola, L and Figueroa, JA and Phan, TT and Grizzi, F and Kim, M and Rahman, RL and Jenkins, MR and Cobos, E and Jum	10.18632/oncotarget.2802
Myelodysplastic syndrome and immunotherapy novel to next in-line treatments	Linder, K and Lulla, P	10.1080/21645515.2021.1898307
Simplified Admix Archaeal Glycolipid Adjuvanted Vaccine and Checkpoint Inhibitor Therapy Combination Enhances Protection from Murine Melanoma	Stark, FC and Agbayani, G and Sandhu, JK and Akache, B and McPherson, C and Deschatelets, L and Dudani, R and Hewitt, I	10.3390/biomedicines7040091
Preparation of Triple-Negative Breast Cancer Vaccine through Electrofusion with Day-3 Dendritic Cells	Zhang, P and Yi, S and Li, X and Liu, R and Jiang, H and Huang, Z and Liu, Y and Wu, J and Huang, Y	10.1371/journal.pone.0102197
Longitudinal efficacy and toxicity of SARS-CoV-2 vaccination in cancer patients treated with immunotherapy	Spiliopoulou, P and van Rensburg, HJJ and Avery, L and Kulasingam, V and Razak, A and Bedard, P and Hansen, A and Chrus	10.1038/s41419-022-05548-4
Adjuvant Peptide Pulsed Dendritic Cell Vaccination in Addition to T Cell Adoptive Immunotherapy for Cytomegalovirus Infection in Allogeneic Hematopoietic Stem Cell Transplantation Recipients	Ma, CKK and Clancy, L and Simms, R and Burgess, J and Deo, S and Blyth, E and Micklethwaite, KR and Gottlieb, DJ	10.1016/j.bbmt.2017.08.028
Type I IFN blockade uncouples immunotherapy-induced antitumor immunity and autoimmune toxicity	Walsh, SR and Bastin, D and Chen, L and Nguyen, A and Storbeck, CJ and Lefebvre, C and Stojdl, D and Bramson, JL and Bel	10.1172/JCI121004
CD14 Expressing Precursors Give Rise to Highly Functional Conventional Dendritic Cells for Use as Dendritic Cell Vaccine	Plantinga, M and van den Beemt, DAMH and Dunnebach, E and Nierkens, S	10.3390/cancers13153818
IL-27 gene therapy induces depletion of Tregs and enhances the efficacy of cancer immunotherapy	Zhu, JM and Liu, JQ and Shi, M and Cheng, XH and Ding, M and Zhang, JCC and Davis, JP and Varikuti, S and Satoskar, AR and	10.1172/jci.insight.98745
Assessment of Immune Response Following Dendritic Cell-Based Immunotherapy in Pediatric Patients With Relapsing Sarcoma	Fedorova, L and Mudry, P and Pilatova, K and Selingerova, I and Merhautova, J and Rehak, Z and Valik, D and Hlavackova, E a	10.3389/fonc.2019.01169
Intranodal Administration of Neoantigen Peptide-loaded Dendritic Cell Vaccine Elicits Epitope-specific T Cell Responses and Clinical Effects in a Patient with Chemorefractory Ovarian Cancer with Malignant Ascites	Morisaki, T and Hikichi, T and Onishi, H and Morisaki, T and Kubo, M and Hirano, T and Yoshimura, S and Kiyotani, K and Nak	10.1080/08820139.2020.1778721
Analysis of the humoral and cellular immune response after a full course of BNT162b2 anti-SARS-CoV-2 vaccine in cancer patients treated with PD-1/PD-L1 inhibitors with or without chemotherapy: an update after 6 months of follow-up	Lasagna, A and Lilleri, D and Agustoni, F and Percivalle, E and Borgetto, S and Alessio, N and Comolli, G and Sarasini, A and	10.1016/j.esmoop.2021.100359
DNA-mediated adjuvant immunotherapy extends survival in two different mouse models of myeloid malignancies	Le Pogam, C and Patel, S and Gorombei, P and Guerenne, L and Krief, P and Omidvar, N and Tekin, N and Bernasconi, E and	10.18632/oncotarget.5572
Virotherapy as Potential Adjunct Therapy for Graft-Vs-Host Disease	Villa, NY and McFadden, G	10.1007/s40139-018-0186-6
COVID-19: Third dose booster vaccine effectiveness against breakthrough coronavirus infection, hospitalisations and death in patients with cancer: A population-based study	Lee, LYW and Ionescu, MC and Starkey, T and Little, M and Tilby, M and Tripathy, AR and Mckenzie, HS and Al-Hajji, Y and Ap	10.1016/j.ejca.2022.06.038
Post-release immune responses of Tasmanian devils vaccinated with an experimental devil facial tumour disease vaccine	Pye, R and Darby, J and Flies, AS and Fox, S and Carver, S and Elmer, J and Swift, K and Hogg, C and Pemberton, D and Wood	10.1071/WR20210
Pathogen boosted adoptive cell transfer immunotherapy to treat solid tumors	Xin, G and Schauder, DM and Jing, WQ and Jiang, AM and Joshi, NS and Johnson, B and Cui, WG	10.1073/pnas.1614315114
Pre-existing immune status associated with response to combination of sipuleucel-T and ipilimumab in patients with metastatic castration-resistant prostate cancer	Sinha, M and Zhang, L and Subudhi, S and Chen, B and Marquez, J and Liu, EV and Allaire, K and Cheung, A and Ng, S and Ng	10.1136/jitc-2020-002254
The association of clinical outcome and peripheral T-cell subsets in metastatic colorectal cancer patients receiving first-line FOLFIRI plus bevacizumab therapy	Roselli, M and Formica, V and Cereda, V and Jochems, C and Richards, J and Grenga, I and Orlandi, A and Ferroni, P and Gua	10.1080/2162402X.2016.1188243
IL-15/IL-15R alpha/CD80-expressing AML cell vaccines eradicate minimal residual disease in leukemic mice	Shi, YM and Dincheva-Vogel, L and Ayemoba, CE and Fung, JP and Bergamaschi, C and Pavlakis, GN and Farzaneh, F and Ga	10.1182/bloodadvances.2018019026
Dendritic cell vaccines targeting tumor blood vessel antigens in combination with dasatinib induce therapeutic immune responses in patients with checkpoint-refractory advanced melanoma	Storkus, WJ and Maurer, D and Lin, Y and Ding, F and Bose, A and Lowe, D and Rose, A and DeMark, M and Karapetyan, L and	10.1136/jitc-2021-003675
Biomimetic doxorubicin/ginsenoside co-loading nanosystem for chemoimmunotherapy of acute myeloid leukemia	Chen, M and Qiao, YY and Cao, J and Ta, L and Ci, TY and Ke, X	10.1186/s12951-022-01491-w
A phase I clinical study of a cocktail vaccine of Wilms' tumor 1 (WT1) HLA class I and II peptides for recurrent malignant glioma	Tsuboi, A and Hashimoto, N and Fujiki, F and Morimoto, S and Kagawa, N and Nakajima, H and Hosen, N and Nishida, S and	10.1007/s00262-018-2274-1
High-grade glioma associated immunosuppression does not prevent immune responses induced by therapeutic vaccines in combination with T-reg depletion	Lohr, M and Freitag, B and Technau, A and Krauss, J and Monoranu, CM and Rachor, J and Lutz, MB and Hagemann, C and Ke	10.1007/s00262-018-2214-0
Dendritic Cell-Based Immunotherapy in Advanced Sarcoma and Neuroblastoma Pediatric Patients: Anti-cancer Treatment Preceding Monocyte Harvest Impairs the Immunostimulatory and Antigen-Presenting Behavior of DCs and Manufacturing Process Outcome	Hlavackova, E and Pilatova, K and Cerna, D and Selingerova, I and Mudry, P and Mazanek, P and Fedorova, L and Merhautov	10.3389/fonc.2019.01034
Autologous dendritic cells pulsed with allogeneic tumour cell lysate induce tumour-reactive T-cell responses in patients with pancreatic cancer: A phase I study	Lau, SP and Klaase, L and Vink, M and Dumas, J and Bezemer, K and van Krimpen, A and van der Breggen, R and Wismans, L	10.1016/j.ejca.2022.03.015

Autophagosome-based strategy to monitor apparent tumor-specific CD8 T cells in patients with prostate cancer	van de Ven, R and Hilton, TL and Hu, HM and Dubay, CJ and Haley, D and Paustian, C and Puri, S and Urba, WJ and Curti, BD	10.1080/2162402X.2018.1466766
Cancer Vaccination against Extracellular Vimentin Efficiently Adjuvanted with Montanide ISA 720/CpG	van Loon, K and Huijbers, EJM and de Haan, JD and Griffioen, AW	10.3390/cancers14112593
Exposure to the antimicrobial peptide LL-37 produces dendritic cells optimized for immunotherapy	Findlay, EG and Currie, AJ and Zhang, AL and Ovciarikova, J and Young, LS and Stevens, H and McHugh, BJ and Canel, M and	10.1080/2162402X.2019.1608106
Expanded human blood-derived gamma delta T cells display potent antigen-presentation functions	Khan, MWA and Curbishley, SM and Chen, HC and Thomas, AD and Pircher, H and Mavilio, D and Steven, NM and Eberl, M a	10.3389/fimmu.2014.00344
Discovery and characterization of tumor antigens in hepatocellular carcinoma for mRNA vaccine development	Fu, JT and Chen, F and Lin, YJ and Gao, J and Chen, AN and Yang, J	10.1007/s00432-022-04325-2
Antibody responses to second doses of COVID-19 vaccination in lung cancer patients undergoing treatment	Narita, D and Ebina-Shibuya, R and Miyauchi, E and Tsukita, Y and Saito, R and Murakami, K and Kimura, N and Sugiura, H	10.1016/j.resinv.2022.11.005
Personalized neoantigen-based immunotherapy for advanced collecting duct carcinoma: case report	Zeng, YY and Zhang, W and Li, ZL and Zheng, YS and Wang, YC and Chen, G and Qiu, LM and Ke, K and Su, XP and Cai, ZX an	10.1136/jitc-2019-000217
Bladder cancer-associated cancer-testis antigen-derived long peptides encompassing both CTL and promiscuous HLA class II-restricted Th cell epitopes induced CD4(+) T cells expressing converged T-cell receptor genes in vitro	Tsuruta, M and Ueda, S and Yew, PY and Fukuda, I and Yoshimura, S and Kishi, H and Hamana, H and Hirayama, M and Yats	10.1080/2162402X.2017.1415687
Cationic nanoparticles enhance T cell tumor infiltration and antitumor immune responses to a melanoma vaccine	Smith, R and Wafa, EI and Geary, SM and Ebeid, K and Alhaj-Suliman, SO and Salem, AK	10.1126/sciadv.abk3150
Adaptive Immunity to Leukemia Is Inhibited by Cross-Reactive Induced Regulatory T Cells	Manlove, LS and Berquam-Vrieze, KE and Pauken, KE and Williams, RT and Jenkins, MK and Farrar, MA	10.4049/jimmunol.1501291
Assessment of the influence of peripheral blood mononuclear cell stimulation with Streptococcus pneumoniae polysaccharides on expression ofselected Toll-like receptors, activation markers and Fas antigen in patients with chronic lymphocytic leukemia	Grywalska, E and Hymos, A and Korona-Glowniak, I and Pasiarski, M and Stelmach-Goldys, A and Gozdz, S and Malm, A and	10.5604/17322693.1219399
Escherichia coli adhesion portion FimH functions as an adjuvant for cancer immunotherapy	Zhang, W and Xu, L and Park, HB and Hwang, J and Kwak, M and Lee, PCW and Liang, G and Zhang, XY and Xu, JQ and Jin, JO	10.1038/s41467-020-15030-4
Phase 1 open-label trial of intravenous administration of MVA-BN-brachyury-TRICOM vaccine in patients with advanced cancer	DeMaria, PJ and Lee-Wisdom, K and Donahue, RN and Madan, RA and Karzai, F and Schwab, A and Palena, C and Jochems,	10.1136/jitc-2021-003238
An oral WT1 protein vaccine composed of WT1-anchored, genetically engineered Bifidobacterium longum allows for intestinal immunity in mice with acute myeloid leukemia	Nakagawa, N and Hashii, Y and Kayama, H and Okumura, R and Nakajima, H and Minagawa, H and Morimoto, S and Fujiki, F	10.1007/s00262-022-03214-4
A Mutated Prostatic Acid Phosphatase (PAP) Peptide-Based Vaccine Induces PAP-Specific CD8(+) T Cells with Ex Vivo Cytotoxic Capacities in HHDII/DR1 Transgenic Mice	Vu, PL and Vadakekolathu, J and Idri, S and Nicholls, H and Cavaignac, M and Reeder, S and Khan, MA and Christensen, D ar	10.3390/cancers14081970
Combination of vasculature targeting, hypofractionated radiotherapy, and immune checkpoint inhibitor elicits potent antitumor immune response and blocks tumor progression	Pierini, S and Mishra, A and Perales-Linares, R and Uribe-Herranz, M and Beghi, S and Giglio, A and Pustylnikov, S and Costa	10.1136/jitc-2020-001636
Autologous dendritic cells and activated cytotoxic T-cells as combination therapy for breast cancer	Shevchenko, JA and Khristin, AA and Kurilin, VV and Kuznetsova, MS and Blinova, DD and Starostina, NM and Sidorov, SV an	10.3892/or.2019.7435
Efficacy of intracellular immune checkpoint-silenced DC vaccine	Wang, DH and Huang, XF and Hong, BX and Song, XT and Hu, LD and Jiang, M and Zhang, B and Ning, HM and Li, YH and Xu,	10.1172/jci.insight.98368
CRISPR/Cas9-based genome editing in the era of CAR T cell immunotherapy	Salas-Mckee, J and Kong, WM and Gladney, WL and Jadlowsky, JK and Plesa, G and Davis, MM and Fraietta, JA	10.1080/21645515.2019.1571893
Antibody-mediated delivery of a viral MHC-I epitope into the cytosol of target tumor cells repurposes virus-specific CD8(+) T cells for cancer immunotherapy	Jung, K and Son, MJ and Lee, SY and Kim, JA and Ko, DH and Yoo, S and Kim, CH and Kim, YS	10.1186/s12943-022-01574-0
Antibody responses after first and second Covid-19 vaccination in patients with chronic lymphocytic leukaemia	Parry, H and McIlroy, G and Bruton, R and Ali, M and Stephens, C and Damery, S and Otter, A and McSkeane, T and Rolfe, H a	10.1038/s41408-021-00528-x
Murine Th9 cells promote the survival of myeloid dendritic cells in cancer immunotherapy	Park, J and Li, HY and Zhang, MJ and Lu, Y and Hong, BX and Zheng, YH and He, J and Yang, J and Qian, JF and Yi, Q	10.1007/s00262-014-1557-4
Immunotherapy with Dendritic Cells Modified with Tumor-Associated Antigen Gene Demonstrates Enhanced Antitumor Effect Against Lung Cancer	Jiang, T and Chen, X and Zhou, W and Fan, GX and Zhao, PL and Ren, SX and Zhou, CC and Zhang, J	10.1016/j.tranon.2016.12.002
Microfluidic on-demand engineering of exosomes towards cancer immunotherapy	Zhao, Z and McGill, J and Gamero-Kubota, P and He, M	10.1039/c8lc01279b
Neoadjuvant in situ vaccination with cowpea mosaic virus as a novel therapy against canine inflammatory mammary cancer	Alonso-Miguel, D and Valdivia, G and Guerrera, D and Perez-Alenza, MD and Pantelyushin, S and Alonso-Diez, A and Beiss, V	10.1136/jitc-2021-004044
The 180 splice variant of NCAM-containing exon 18-is specifically expressed in small cell lung cancer cells	Vander Borght, A and Duysinx, M and Broers, JLV and Ummelen, M and Falkenberg, FW and Hahnel, C and van der Zeijst, BA	10.21037/tlcr.2018.03.03
Long-term response of metastatic renal clear cell carcinoma following a subcutaneous injection of mixed bacterial vaccine: a case report	Chen, JB and Lv, YY and Mu, F and Xu, KC	10.2147/OTT.S200414
Tumor-targeted delivery of sunitinib base enhances vaccine therapy for advanced melanoma by remodeling the tumor microenvironment	Huo, MR and Zhao, Y and Satterlee, AB and Wang, YH and Xu, Y and Huang, L	10.1016/j.jconrel.2016.11.013
Human blood myeloid and plasmacytoid dendritic cells cross activate each other and synergize in inducing NK cell cytotoxicity	van Beek, JJP and Gorris, MAJ and Skold, AE and Hatipoglu, I and Van Acker, HH and Smits, EL and de Vries, IJM and Bakdash	10.1080/2162402X.2016.1227902
Cross-presentation of a TAP-independent signal peptide induces CD8 T immunity to escaped cancers but necessitates anchor replacement	Marijt, KA and Griffioen, L and Blijleven, L and van der Burg, SH and van Hall, T	10.1007/s00262-021-02984-7
PPAR gamma Contributes to Immunity Induced by Cancer Cell Vaccines That Secrete GM-CSF	Goyal, G and Wong, K and Nirschl, CJ and Souders, N and Neuberg, D and Anandasabapathy, N and Dranoff, G	10.1158/2326-6066.CIR-17-0612
Predictive significance of T cell subset changes during ex vivo generation of adoptive cellular therapy products for the treatment of advanced non-small cell lung cancer	Huang, LF and Qiao, GL and Morse, MA and Wang, XL and Zhou, XN and Wu, JP and Hobeika, A and Ren, J and Lyerly, HK	10.3892/ol.2019.10964

Limiting glioma development by photodynamic therapy-generated macrophage vaccine and allo-stimulation: an in vivo histological study in rats	Madsen, SJ and Christie, C and Huynh, K and Peng, Q and Uzal, FA and Krasieva, TB and Hirschberg, H	10.1117/1.JBO.23.2.028001
The Genomic Landscape of Antigenic Targets for T Cell-Based Leukemia Immunotherapy	Hardy, MP and Vincent, K and Perreault, C	10.3389/fimmu.2019.02934
CD40 and CD80/86 signaling in cDC1s mediate effective neoantigen vaccination and generation of antigen-specific CX3CR1(+) CD8(+) T cells	Yamauchi, T and Hoki, T and Oba, T and Kajihara, R and Attwood, K and Cao, XF and Ito, F	10.1007/s00262-021-02969-6
Cancer/testis antigens expression during cultivation of melanoma and soft tissue sarcoma cells	Danilova, A and Misyurin, V and Novik, A and Girdyuk, D and Avdonkina, N and Nekhaeva, T and Emelyanova, N and Pipia, N	10.1186/s13569-020-0125-2
Chimeric Antigen Receptor T Cell Therapy in Hematology	Ataca, P and Arslan, O	10.4274/tjh.2015.0049
WT1 peptide vaccine in Montanide in contrast to poly ICLC, is able to induce WT1-specific immune response with TCR clonal enrichment in myeloid leukemia	Liu, HT and Zha, YY and Choudhury, N and Malnassy, G and Fulton, N and Green, M and Park, JH and Nakamura, Y and Larso	10.1186/s40164-018-0093-x
Prophylactic and therapeutic antileukemic effects induced by the AAC-11-derived Peptide RT53	Habault, J and Kaci, A and Pasquereau-Kotula, E and Fraser, C and Chomienne, C and Dombret, H and Braun, T and Pla, M a	10.1080/2162402X.2020.1728871
Identification of a novel HLA-A*02:01-restricted cytotoxic T lymphocyte epitope derived from the EML4-ALK fusion gene	Yoshimura, M and Tada, Y and Ofuzi, K and Yamamoto, M and Nakatsura, T	10.3892/or.2014.3198
mRNA-Based Anti-TCR CDR3 Tumour Vaccine for T-Cell Lymphoma	Tusup, M and Lauchli, S and Jarzebska, NT and French, LE and Chang, YT and Vonow-Eisenring, M and Su, A and Kundig, TM	10.3390/pharmaceutics13071040
Predicting combinations of immunomodulators to enhance dendritic cell-based vaccination based on a hybrid experimental and computational platform	Ahmed, R and Crespo, I and Tuyaerts, S and Bekkar, A and Graciotti, M and Xenarios, I and Kandalaft, LE	10.1016/j.csbj.2020.08.001
APOBEC3B-mediated corruption of the tumor cell immunopeptidome induces heteroclitic neoepitopes for cancer immunotherapy	Driscoll, CB and Schuelke, MR and Kottke, T and Thompson, JM and Wongthida, P and Tonne, JM and Huff, AL and Miller, A a	10.1038/s41467-020-14568-7
Identification of an immunogenic DKK1 long peptide for immunotherapy of human multiple myeloma	Li, R and Zheng, CY and Wang, Q and Bi, EG and Yang, MJ and Hou, J and Fu, WJ and Yi, Q and Qian, JF	10.3324/haematol.2019.236836
Targeted Programming of the Lymph Node Environment Causes Evolution of Local and Systemic Immunity	Andorko, JI and Gammon, JM and Tostanoski, LH and Zeng, Q and Jewell, CM	10.1007/s12195-016-0455-6
Phase I study to evaluate toxicity and feasibility of intratumoral injection of alpha-gal glycolipids in patients with advanced melanoma	Albertini, MR and Ranheim, EA and Zuleger, CL and Sondel, PM and Hank, JA and Bridges, A and Newton, MA and McFarland	10.1007/s00262-016-1846-1
Capability of Human Dendritic Cells Pulsed with Autologous Induced Pluripotent Stem Cell Lysate to Induce Cytotoxic T Lymphocytes against HLA-A33-Matched Cancer Cells	Nakazawa, T and Maeoka, R and Morimoto, T and Matsuda, R and Nakamura, M and Nishimura, F and Yamada, S and Nakag	10.3390/ijms232112992
Vaccines adjuvanted with an NKT cell agonist induce effective T-cell responses in models of CNS lymphoma	Grasso, C and Field, CS and Tang, CW and Ferguson, PM and Compton, BJ and Anderson, RJ and Painter, GF and Weinkove,	10.2217/imt-2019-0134
Exogenous Addition of Arachidonic Acid to the Culture Media Enhances the Functionality of Dendritic Cells for Their Possible Use in Cancer Immunotherapy	Kumar, J and Gurav, R and Kale, V and Limaye, L	10.1371/journal.pone.0111759
Immunomodulatory Effects of Stereotactic Body Radiotherapy and Vaccination with Heat-Killed Mycobacterium Obuense (IMM-101) in Patients with Locally Advanced Pancreatic Cancer	Land, FRV and Lau, SP and De Koning, W and Klaase, L and Vink, M and Van Krimpen, A and Dumas, J and Vadgama, D and N	10.3390/cancers14215299
Evaluation of prognostic significance of granulocyte-related factors in cancer patients undergoing personalized peptide vaccination	Sakamoto, S and Yoshitomi, M and Yutani, S and Terazaki, Y and Yoshiyama, K and Ioji, T and Matsueda, S and Yamada, A ar	10.1080/21645515.2015.1075107
Sensitive Detection and Analysis of Neoantigen-Specific T Cell Populations from Tumors and Blood	Peng, SM and Zaretsky, JM and Ng, AHC and Chour, W and Bethune, MT and Choi, J and Hsu, A and Holman, E and Ding, XZ a	10.1016/j.celrep.2019.07.106
cDC1 Vaccines Drive Tumor Rejection by Direct Presentation Independently of Host cDC1	Ferris, ST and Ohara, RA and Ou, FY and Wu, RE and Huang, X and Kim, S and Chen, J and Liu, TT and Schreiber, RD and Murp	10.1158/2326-6066.CIR-21-0865
Personal Neoantigen Cancer Vaccines: A Road Not Fully Paved	Fritsch, EF and Burkhardt, UE and Hacohen, N and Wu, CJ	10.1158/2326-6066.CIR-20-0526
SnoopLigase peptide-peptide conjugation enables modular vaccine assembly	Andersson, AMC and Buldun, CM and Pattinson, DJ and Draper, SJ and Howarth, M	10.1038/s41598-019-40985-w
Automated Flow Synthesis of Tumor Neoantigen Peptides for Personalized Immunotherapy	Truex, NL and Holden, RL and Wang, BY and Chen, PG and Hanna, S and Hu, ZT and Shetty, K and Olive, O and Neuberg, D a	10.1038/s41598-019-56943-5
IL-17 and IL-23 levels in patients with early-stage chronic lymphocytic leukemia	Bankir, M and Acik, DY	10.14744/nci.2020.02997
Engineered red blood cells (activating antigen carriers) drive potent T cell responses and tumor regression in mice	Blagovic, K and Smith, C and Ramakrishnan, A and Moore, L and Soto, D and Thompson, Z and Stockmann, A and Kruszelnic	10.3389/fimmu.2022.1015585
Impaired neutralisation of SARS-CoV-2 delta variant in vaccinated patients with B cell chronic lymphocytic leukaemia	Parry, H and McIlroy, G and Bruton, R and Damery, S and Tyson, G and Logan, N and Davis, C and Willett, B and Zuo, JM and	10.1186/s13045-021-01219-7
Blinatumomab as bridging therapy in paediatric B-cell acute lymphoblastic leukaemia complicated by invasive fungal disease	Yeoh, DK and Blyth, CC and Kotecha, RS	10.1111/bjh.18314
Red blood cell-derived nanoerythrosome for antigen delivery with enhanced cancer immunotherapy	Han, X and Shen, SF and Fan, Q and Chen, GJ and Archibong, E and Dotti, G and Liu, Z and Gu, Z and Wang, C	10.1126/sciadv.aaw6870
Selection and T-cell antigenicity of synthetic long peptides derived from SARS-CoV-2	Piadel, K and Haybatollahi, A and Dalgleish, AG and Smith, PL	10.1099/jgv.0.001698
Anti-CD73 antibody activates human B cells, enhances humoral responses and induces redistribution of B cells in patients with cancer	Miller, RA and Luke, JJ and Hu, SS and Mahabhashyam, S and Jones, WB and Marron, T and Merchan, JR and Hughes, BGM ar	10.1136/jitc-2022-005802
Divergent HIV-1-Directed Immune Responses Generated by Systemic and Mucosal Immunization with Replicating Single-Cycle Adenoviruses in Rhesus Macaques	Matchett, WE and Anguiano-Zarate, SS and Nehete, PN and Shelton, K and Nehete, BP and Yang, GJ and Dorta-Estremera, S	10.1128/JVI.02016-18
Autologous Hematopoietic Stem Cells Are a Preferred Source to Generate Dendritic Cells for Immunotherapy in Multiple Myeloma Patients	Shinde, P and Melinkeri, S and Santra, MK and Kale, V and Limaye, L	10.3389/fimmu.2019.01079
A cancer vaccine approach for personalized treatment of Lynch Syndrome	Majumder, S and Shah, R and Elias, J and Manoharan, M and Shah, P and Kumari, A and Chakraborty, P and Kode, V and Mis	10.1038/s41598-018-30466-x

Dynamic and specific immune responses against multiple tumor antigens were elicited in patients with hepatocellular carcinoma after cell-based immunotherapy	Han, YY and Wu, YT and Yang, C and Huang, J and Guo, YB and Liu, L and Chen, P and Wu, DY and Liu, JY and Li, J and Zhou,	10.1186/s12967-017-1165-0
A randomized phase III trial of personalized peptide vaccination for castration-resistant prostate cancer progressing after docetaxel	Noguchi, M and Fujimoto, K and Arai, G and Uemura, H and Hashine, K and Matsumoto, H and Fukasawa, S and Kohjimoto,	10.3892/or.2020.7847
Robust Anti-Tumor T Cell Response with Efficient Intratumoral Infiltration by Nanodisc Cancer Immunotherapy	Kuai, R and Singh, PB and Sun, XQ and Xu, C and Najafabadi, AH and Scheetz, L and Yuan, WM and Xu, Y and Hong, H and K	10.1002/adtp.202000094
This is not a pipe - But how harmful is electronic cigarette smoke?	Hafner, SJ	10.1016/j.bj.2021.05.006
Glycolipid-peptide conjugate vaccines enhance CD8(+) T cell responses against human viral proteins	Speir, M and Authier-Hall, A and Brooks, CR and Farrand, KJ and Compton, BJ and Anderson, RJ and Heiser, A and Osmond,	10.1038/s41598-017-14690-5
Immune correlates of clinical benefit in a phase I study of hyperthermia with adoptive T cell immunotherapy in patients with solid tumors	Qiao, GL and Wang, XL and Zhou, XN and Morse, MA and Wu, JP and Wang, S and Song, YG and Jiang, N and Zhao, YJ and Zh	10.1080/02656736.2019.1647350
Gene-modified hematopoietic stem cells for cancer immunotherapy	Larson, S and De Oliveira, SN	10.4161/hv.27637
Limited T cell response to SARS-CoV-2 mRNA vaccine among patients with cancer receiving different cancer treatments	Cortes, A and Casado, JL and Longo, F and Serrano, JJ and Saavedra, C and Velasco, H and Martin, A and Chamorro, J and R	10.1016/j.ejca.2022.02.017
Characterization of the T cell receptor repertoire and melanoma tumor microenvironment upon combined treatment with ipilimumab and hTERT vaccination	Ellingsen, EB and Bounova, G and Kerzeli, I and Anzar, I and Simnica, D and Aamdal, E and Guren, T and Clancy, T and Mezh	10.1186/s12967-022-03624-z
Tumor-Derived Membrane Vesicles from the IL-2 Overexpression Melanoma Cells Affect on the Expression of Surface Markers of Human Peripheral Blood Mononuclear Cells In Vitro	Filin, IY and Kitaeva, KV and Chulpanova, DS and Rizvanov, AA and Akhmetzyanova, ER and Solovyeva, VV	10.1007/s12668-022-01044-3
Vaccine Development against the Renin-Angiotensin System for the Treatment of Hypertension	Azegami, T and Itoh, H	10.1155/2019/9218531
Dysregulated NF-kappa B-Dependent ICOSL Expression in Human Dendritic Cell Vaccines Impairs T-cell Responses in Patients with Melanoma	Maurer, DM and Adamik, J and Santos, PM and Shi, J and Shurin, MR and Kirkwood, JM and Storkus, WJ and Butterfield, LH	10.1158/2326-6066.CIR-20-0274
Route of antigen delivery impacts the immunostimulatory activity of dendritic cell-based vaccines for hepatocellular carcinoma	Pardee, AD and Yano, H and Weinstein, AM and Ponce, AAK and Ethridge, AD and Normolle, DP and Vujanovic, L and Mizeje	10.1186/s40425-015-0077-x
Pilot Acute Safety Evaluation of Innocell (TM) Cancer Immunotherapy in Canine Subjects	Goodrich, RP and Weston, J and Hartson, L and Griffin, L and Guth, A	10.1155/2020/7142375
Umbilical cord blood-derived CD11c(+) dendritic cells could serve as an alternative allogeneic source of dendritic cells for cancer immunotherapy	Kumar, J and Kale, V and Limaye, L	10.1186/s13287-015-0160-8
Pretreatment antigen-specific immunity and regulation - association with subsequent immune response to anti-tumor DNA vaccination	Johnson, LE and Olson, BM and McNeel, DG	10.1186/s40425-017-0260-3
Dendritic Cell/Cytokine-Induced Killer Cell Immunotherapy Combined with S-1 in Patients with Advanced Pancreatic Cancer: A Prospective Study	Jiang, N and Qiao, GL and Wang, XL and Morse, MA and Gwin, WR and Zhou, L and Song, YG and Zhao, YJ and Chen, F and Z	10.1158/1078-0432.CCR-17-0492
Generation of Tumor-Specific Cytotoxic T Cells From Blood via In Vitro Expansion Using Autologous Dendritic Cells Pulsed With Neoantigen-Coupled Microbeads	Kiessling, A and Ramanathan, K and Nilsson, OB and Notari, L and Renken, S and Kiessling, R and Gronlund, H and Wickstro	10.3389/fonc.2022.866763
Vaccine Protection of Leukopenic Mice against Staphylococcus aureus Bloodstream Infection	Rauch, S and Gough, P and Kim, HK and Schneewind, O and Missiakas, D	10.1128/IAI.02328-14
Development of a novel immunoproteasome digestion assay for synthetic long peptide vaccine design	Wada, H and Shimizu, A and Osada, T and Tanaka, Y and Fukaya, S and Sasaki, E	10.1371/journal.pone.0199249
Immunological analysis of phase II glioblastoma dendritic cell vaccine (Audencel) trial: immune system characteristics influence outcome and Audencel up-regulates Th1-related immunovariables	Erhart, F and Buchroithner, J and Reitermaier, R and Fischhuber, K and Klingenbrunner, S and Sloma, I and Hibsh, D and Koz	10.1186/s40478-018-0621-2
Maturation of dendritic cells by pullulan promotes anti-cancer effect	Zhang, W and Yu, XQ and Kwak, M and Xu, L and Zhang, LJ and Yu, Q and Jin, JO	10.18632/oncotarget.10183
The cancer-testis antigen, sperm protein 17, a new biomarker and immunological target in head and neck squamous cell carcinoma	Schutt, CA and Mirandola, L and Figueroa, JA and Nguyen, DD and Cordero, J and Bumm, K and Judson, BL and Chiriva-Inter	10.18632/oncotarget.22213
Large-scale expansion of gamma delta T cells and peptide-specific cytotoxic T cells using zoledronate for adoptive immunotherapy	Yoshikawa, T and Takahara, M and Tomiyama, M and Nieda, M and Maekawa, R and Nakatsura, T	10.3892/ijo.2014.2634
Hematopoietic stem cell-derived myeloid and plasmacytoid DC-based vaccines are highly potent inducers of tumor-reactive T cell and NK cell responses ex vivo	Thordardottir, S and Schaap, N and Louer, E and Kester, MGD and Falkenburg, JHF and Jansen, J and Radstake, TRD and Hob	10.1080/2162402X.2017.1285991
NKT cell-targeted vaccination plus anti-4-1BB antibody generates persistent CD8 T cell immunity against B cell lymphoma	Kobayashi, T and Doff, BL and Rearden, RC and Leggatt, GR and Mattarollo, SR	10.4161/2162402X.2014.990793
Targeting DNGR-1 (CLEC9A) with antibody/MUC1 peptide conjugates as a vaccine for carcinomas	Picco, G and Beatson, R and Taylor-Papadimitriou, J and Burchell, JM	10.1002/eji.201344076
The metabolic enzyme arginase-2 is a potential target for novel immune modulatory vaccines	Weis-Banke, SE and Hubbe, ML and Holmstrom, MO and Jorgensen, MA and Bendtsen, SK and Martinenaite, E and Carretta,	10.1080/2162402X.2020.1771142
Emerging Opportunities and Challenges in Cancer Immunotherapy	Whiteside, TL and Demaria, S and Rodriguez-Ruiz, ME and Zarour, HM and Melero, I	10.1158/1078-0432.CCR-16-0049
Association of SARS-CoV-2 Spike Protein Antibody Vaccine Response With Infection Severity in Patients With Cancer A National COVID Cancer Cross-sectional Evaluation	Lee, LYW and Tilby, M and Starkey, T and Ionescu, MC and Burnett, A and Hattersley, R and Khan, S and Little, M and Liu, JKH	10.1001/jamaoncol.2022.5974
An Autologous Dendritic Cell Vaccine Promotes Anticancer Immunity in Patients with Ovarian Cancer with Low Mutational Burden and Cold Tumors	Fucikova, J and Hensler, M and Kasikova, L and Lanickova, T and Pasulka, J and Rakova, J and Drozenova, J and Fredriksen, T	10.1158/1078-0432.CCR-21-4413
Neoadjuvant PROSTVAC prior to radical prostatectomy enhances T-cell infiltration into the tumor immune microenvironment in men with prostate cancer	Sater, HA and Marte, JL and Donahue, RN and Walter-Rodriguez, B and Heery, CR and Steinberg, SM and Cordes, LM and Ch	10.1136/jitc-2020-000655

Cowpea mosaic virus stimulates antitumor immunity through recognition by multiple MYD88-dependent toll-like receptors	Mao, CK and Beiss, V and Fields, J and Steinmetz, NF and Fiering, S	10.1016/j.biomaterials.2021.120914
Cancer treatment in the lymphatic system: A prospective targeting employing nanostructured systems	Yukuyama, MN and de Araujo, GLB and de Souza, A and Lobenberg, R and Barbosa, EJ and Henostroza, MAB and da Rocha, I	10.1016/j.ijpharm.2020.119697
TriKEs and BiKEs join CARs on the cancer immunotherapy highway	Tay, SS and Carol, H and Biro, M	10.1080/21645515.2016.1198455
Melanoma Immunotherapy in Mice Using Genetically Engineered Pluripotent Stem Cells	Haque, M and Song, JY and Fino, K and Sandhu, P and Wang, YF and Ni, B and Fang, DY and Song, JX	10.3727/096368916X690467
Exploring the Potential Use of a PBMC-Based Functional Assay to Identify Predictive Biomarkers for Anti-PD-1 Immunotherapy	Bacot, SM and Harper, TA and Matthews, RL and Fennell, CJ and Akue, A and KuKuruga, MA and Lee, S and Wang, T and Feld	10.3390/ijms21239023
Phenotypic and genetic evaluation of human monocyte-derived dendritic cells generated from whole blood for immunotherapy	Hussein, YM and Hendawy, DM and Alghamdy, AN and Raafat, N	10.1186/s43042-021-00168-7
Delivery of TLR7 agonist to monocytes and dendritic cells by DCIR targeted liposomes induces robust production of anti-cancer cytokines	Klauber, TCB and Laursen, JM and Zucker, D and Brix, S and Jensen, SS and Andresen, TL	10.1016/j.actbio.2017.01.072
Therapeutic efficacy of combined vaccination against tumor pericyte-associated antigens DLK1 and DLK2 in mice	Fabian, KPL and Chi-Sabins, N and Taylor, JL and Fecek, R and Weinstein, A and Storkus, WJ	10.1080/2162402X.2017.1290035
Non-clinical safety evaluation of repeated intramuscular administration of the AS15 immunostimulant combined with various antigens in rabbits and cynomolgus monkeys	Gacron, N and Silvano, J and Kuper, CF and Baudson, N and Gerard, C and Forster, R and Segal, L	10.1002/jat.3167
A new synthetic toll-like receptor 1/2 ligand is an efficient adjuvant for peptide vaccination in a human volunteer	Rammensee, HG and Wiesmuller, KH and Chandran, PA and Zelba, H and Rusch, E and Gouttefangeas, C and Kowalewski,	10.1186/s40425-019-0796-5
Identification of HLA-A*1101-restricted cytotoxic T lymphocyte epitopes derived from epidermal growth factor pathway substrate number 8	Lu, HF and Tang, BS and He, YJ and Zhou, WJ and Qiu, JL and Li, YH	10.3892/mmr.2016.5888
NIPU: a randomised, open-label, phase II study evaluating nivolumab and ipilimumab combined with UV1 vaccination as second line treatment in patients with malignant mesothelioma	Haakensen, VD and Nowak, AK and Ellingsen, EB and Farooqi, SJ and Bjaanaes, MM and Horndalsveen, H and Mcculloch, T a	10.1186/s12967-021-02905-3
Mannose-Modified Liposome Co-Delivery of Human Papillomavirus Type 16 E7 Peptide and CpG Oligodeoxynucleotide Adjuvant Enhances Antitumor Activity Against Established Large TC-1 Grafted Tumors in Mice	Zhao, Y and Wang, H and Yang, Y and Jia, WD and Su, T and Che, YX and Feng, YX and Yuan, XM and Wang, XL	10.2147/IJN.S275670
Harnessing Unconventional T Cells for Immunotherapy of Tuberculosis	La Manna, MP and Orlando, V and Tamburini, B and Badami, GD and Dieli, F and Caccamo, N	10.3389/fimmu.2020.02107
TNP-470 skews DC differentiation to Th1-stimulatory phenotypes and can serve as a novel adjuvant in a cancer vaccine	Ho, DHH and Wong, RHF	10.1182/bloodadvances.2017013433
Investigation of factors associated with reduced clinical benefits of personalized peptide vaccination for pancreatic cancer	Uchino, Y and Muroya, D and Yoshitomi, M and Shichijo, S and Yamada, A and Sasada, T and Yamada, T and Okuda, K and It	10.3892/mco.2020.2201
Immunological ignorance is an enabling feature of the oligo-clonal T cell response to melanoma neoantigens	Linette, GP and Becker-Hapak, M and Skidmore, ZL and Baroja, ML and Xu, C and Hundal, J and Spencer, DH and Fu, WX and	10.1073/pnas.1906026116
Efficient Eradication of Established Tumors in Mice with Cationic Liposome-Based Synthetic Long-Peptide Vaccines	Varypataki, EM and Benne, N and Bouwstra, J and Jiskoot, W and Ossendorp, F	10.1158/2326-6066.CIR-16-0283
The distribution and function of human memory T cell subsets in lung cancer	Sheng, SY and Gu, Y and Lu, CG and Zou, JY and Hong, H and Wang, RF	10.1007/s12026-016-8882-y
Quantification and characterization of granulocyte macrophage colony-stimulating factor activated human peripheral blood mononuclear cells by fluorine-19 cellular MRI in an immunocompromised mouse model	Fink, C and Smith, M and Sehl, OC and Gaudet, JM and Meagher, TC and Sheikh, NA and Dikeakos, JD and Rieder, MJ and Fo	10.1016/j.diii.2020.02.004
Peptide Super-Agonist Enhances T-Cell Responses to Melanoma	Galloway, SAE and Dolton, G and Attaf, M and Wall, A and Fuller, A and Rius, C and Bianchi, V and Theaker, S and Lloyd, A ar	10.3389/fimmu.2019.00319
Low-Dose Cyclophosphamide Induces Antitumor T-Cell Responses, which Associate with Survival in Metastatic Colorectal Cancer	Scurr, M and Pembroke, T and Bloom, A and Roberts, D and Thomson, A and Smart, K and Bridgeman, H and Adams, R and E	10.1158/1078-0432.CCR-17-0895
Immunosuppressive CD14(+)HLA-DRlo/neg monocytes are elevated in pancreatic cancer and "primed" by tumor-derived exosomes	Javeed, N and Gustafson, MP and Dutta, SK and Lin, Y and Bamlet, WR and Oberg, AL and Petersen, GM and Chari, ST and D	10.1080/2162402X.2016.1252013
The Safety and Immunological Effects of rAd5-EBV-LMP2 Vaccine in Nasopharyngeal Carcinoma Patients: A Phase I Clinical Trial and Two-Year Follow-Up	Si, YF and Deng, ZX and Lan, GP and Du, HJ and Wang, YL and Si, JY and Wei, JZ and Weng, JJ and Qin, YD and Huang, B and Y	10.1248/cpb.c16-00114
The Superior Ability of Human BDCA3(+)(CD141(+)) Dendritic Cells (DCs) to Cross-Present Antigens Derived From Necrotic Lung Cancer Cells	Gu, FF and Zhang, K and Ma, LL and Liu, YY and Li, C and Hu, Y and Yang, QF and Liang, JY and Zeng, YL and Wang, Y and Liu,	10.3389/fimmu.2020.01267
SV-BR-1-GM, a Clinically Effective GM-CSF-Secreting Breast Cancer Cell Line, Expresses an Immune Signature and Directly Acticates CD4(+) T Lymphocytes	Lacher, MD and Bauer, G and Fury, B and Graeve, S and Fledderman, EL and Petrie, TD and Coleal-Bergum, DP and Hackett,	10.3389/fimmu.2018.00776
An Anticancer Drug Cocktail of Three Kinase Inhibitors Improved Response to a Dendritic Cell-Based Cancer Vaccine	Guo, JT and Muse, E and Christians, AJ and Swanson, SJ and Davila, E	10.1158/2326-6066.CIR-18-0684
Clinical research of genetically modified dendritic cells in combination with cytokine-induced killer cell treatment in advanced renal cancer	Wang, DH and Zhang, B and Gao, HY and Ding, GL and Wu, Q and Zhang, JC and Liao, L and Chen, H	10.1186/1471-2407-14-251
Systems vaccinology of the BNT162b2 mRNA vaccine in humans	Arunachalam, PS and Scott, MKD and Hagan, T and Li, CF and Feng, YP and Wimmers, F and Grigoryan, L and Trisal, M and E	10.1038/s41586-021-03791-x

Polyelectrolyte Multilayers Assembled Entirely from Immune Signals on Gold Nanoparticle Templates Promote Antigen-Specific T Cell Response	Zhang, PP and Chiu, YC and Tostanoski, LH and Jewell, CM	10.1021/acsnano.5b02153
Phenotypic and Functional Dysregulated Blood NK cells in colorectal cancer Patients can Be activated by cetuximab Plus IL-2 or IL-15	Rocca, YS and Roberti, MP and Julia, EP and Pampena, MB and Bruno, L and Rivero, S and Huertas, E and Loria, FS and Pairo	10.3389/fimmu.2016.00413
WT1-specific TCRs directed against newly identified peptides install antitumor reactivity against acute myeloid leukemia and ovarian carcinoma	van Amerongen, RA and Hagedoorn, RS and Remst, DFG and Assendelft, DC and van der Steen, DM and Wouters, AK and va	10.1136/jitc-2021-004409
Therapeutic Peptide Vaccine-Induced CD8 T Cells Strongly Modulate Intratumoral Macrophages Required for Tumor Regression	van der Sluis, TC and Sluijter, M and van Duikeren, S and West, BL and Melief, CJM and Arens, R and van der Burg, SH and va	10.1158/2326-6066.CIR-15-0052
Arginase 1-Based Immune Modulatory Vaccines Induce Anticancer Immunity and Synergize with Anti-PD-1 Checkpoint Blockade	Jorgensen, MA and Ugel, S and Hubbe, ML and Carretta, M and Perez-Penco, M and Weis-Banke, SE and Martinenaite, E and	10.1158/2326-6066.CIR-21-0280
Human umbilical vein endothelial cell vaccine suppresses the angiogenesis of esophageal squamous cell carcinoma in a humanized mouse model	Liu, HF and Zhao, JM and Yang, Y and Jin, GG and Zhang, XY and Wang, DY and Xie, C and Liu, KD and Chen, XH and Li, X and	10.3892/or.2018.6653
The Scottish COVID Cancer Immunity Prevalence Study: A Longitudinal Study of SARS-CoV-2 Immune Response in Patients Receiving Anti-Cancer Treatment	Purshouse, K and Thomson, JP and Vallet, M and Alexander, L and Bonisteel, I and Brennan, M and Cameron, DA and Figuer	10.1093/oncolo/oyac257
Exploiting a new strategy to induce immunogenic cell death to improve dendritic cell-based vaccines for lymphoma immunotherapy	Montico, B and Lapenta, C and Ravo, M and Martorelli, D and Muraro, E and Zeng, B and Comaro, E and Spada, M and Donat	10.1080/2162402X.2017.1356964
Angiogenic Cytokines Are Antibody Targets During Graft-versus-Leukemia Reactions	Piesche, M and Ho, VT and Kim, H and Nakazaki, Y and Nehil, M and Yaghi, NK and Kolodin, D and Weiser, J and Altevogt, P a	10.1158/1078-0432.CCR-14-1956
Tetanus toxoid and CCL3 improve dendritic cell vaccines in mice and glioblastoma patients	Mitchell, DA and Batich, KA and Gunn, MD and Huang, MN and Sanchez-Perez, L and Nair, SK and Congdon, KL and Reap, E/	10.1038/nature14320
Audencel Immunotherapy Based on Dendritic Cells Has No Effect on Overall and Progression-Free Survival in Newly Diagnosed Glioblastoma: A Phase II Randomized Trial	Buchroithner, J and Erhart, F and Pichler, J and Widhalm, G and Preusser, M and Stockhammer, G and Nowosielski, M and Ig	10.3390/cancers10100372
Antitumor DNA vaccination against the Sox2 transcription factor	Polakova, I and Duskova, M and Smahel, M	10.3892/ijo.2014.2402
Cancer Vaccine Therapy Using Carcinoembryonic Antigen - expressing Dendritic Cells generated from Induced Pluripotent Stem Cells	Kitadani, J and Ojima, T and Iwamoto, H and Tabata, H and Nakamori, M and Nakamura, M and Hayata, K and Katsuda, M an	10.1038/s41598-018-23120-z
Selective targeting of Toll-like receptors and OX40 inhibit regulatory T-cell function in follicular lymphoma	Voo, KS and Foglietta, M and Percivalle, E and Chu, FL and Nattamai, D and Harline, M and Lee, ST and Bover, L and Lin, HY	10.1002/ijc.28937
Pre-clinical data supporting immunotherapy for HIV using CMV-HIV-specific CAR T cells with CMV vaccine	Guan, M and Lim, L and Holguin, L and Han, TX and Vyas, V and Urak, R and Miller, A and Browning, DL and Echavarria, L and	10.1016/j.omtm.2022.04.007
Contribution of pre-existing neoantigen-specific T cells to a durable complete response after tumor-pulsed dendritic cell vaccine plus nivolumab therapy in a patient with metastatic salivary duct carcinoma	Ichimiya, S and Fujimura, A and Masuda, M and Masuda, S and Yasumatsu, R and Umebayashi, M and Tanaka, H and Koya, I	10.1080/08820139.2021.1973491
A conjugate of octamer-binding transcription factor 4 and toll-like receptor 7 agonist prevents the growth and metastasis of testis embryonic carcinoma	Lin, GM and Wang, XM and Yi, WX and Zhang, CX and Xu, GX and Zhu, XM and Cai, ZM and Liu, Y and Diao, YW and Lin, MCM	10.1186/s12967-015-0524-y
Synergistic anti-tumor effects of dasatinib and dendritic cell vaccine on metastatic breast cancer in a mouse model	Song, NN and Guo, HL and Ren, J and Hao, SH and Wang, XC	10.3892/ol.2018.8188
Immunotherapy in gliomas: Are we reckoning without the innate immunity?	Vismara, MFM and Donato, A and Malara, N and Presta, I and Donato, G	10.1177/2058738419843378
Immune monitoring and TCR sequencing of CD4 T cells in a long term responsive patient with metastasized pancreatic ductal carcinoma treated with individualized, neoepitope-derived multipeptide vaccines: a case report	Sonntag, K and Hashimoto, H and Eyrich, M and Menzel, M and Schubach, M and Docker, D and Battke, F and Courage, C ar	10.1186/s12967-018-1382-1
Zoledronic acid renders human M1 and M2 macrophages susceptible to V delta 2(+) gamma delta T cell cytotoxicity in a perforin-dependent manner	Fowler, DW and Copier, J and Dalgleish, AG and Bodman-Smith, MD	10.1007/s00262-017-2011-1
A plant-expressed conjugate vaccine breaks CD4(+) tolerance and induces potent immunity against metastatic Her2(+) breast cancer	Chotprakaikiat, W and Allen, A and Bui-Minh, D and Harden, E and Jobsri, J and Cavallo, F and Gleba, Y and Stevenson, FK ar	10.1080/2162402X.2016.1166323
Functional T cells targeting tumor-associated antigens are predictive for recurrence-free survival of patients with radically operated non-small cell lung cancer	Safi, S and Yamauchi, Y and Rathinasamy, A and Stamova, S and Eichhorn, M and Warth, A and Rauch, G and Dienemann, H	10.1080/2162402X.2017.1360458
Immunogenicity And Safety Of The 13-Valent Pneumococcal Conjugate Vaccine In Patients With Monoclonal Gammopathy Of Undetermined Significance - Relationship With Selected Immune And Clinical Parameters	Pasiarski, M and Sosnowska-Pasiarska, B and Grywalska, E and Stelmach-Goldys, A and Kowalik, A and Gozdz, S and Rolins	10.2147/CIA.S220423
Quantitative and qualitative impairments in dendritic cell subsets of patients with ovarian or prostate cancer	Mastelic-Gavillet, B and Sarivalasis, A and Lozano, LE and Wyss, T and Inoges, S and de Vries, IJM and Dartiguenave, F and Ji	10.1016/j.ejca.2020.04.036
In vitro Evidence of Human Immune Responsiveness Shows the Improved Potential of a Recombinant BCG Strain for Bladder Cancer Treatment	Rodriguez, D and Goulart, C and Pagliarone, AC and Silva, EP and Cunegundes, PS and Nascimento, IP and Borra, RC and D	10.3389/fimmu.2019.01460
Dendritic cell vaccination as postremission treatment to prevent or delay relapse in acute myeloid leukemia	Anguille, S and Van de Velde, AL and Smits, EL and Van Tendeloo, VF and Juliusson, G and Cools, N and Nijs, G and Stein, B	10.1182/blood-2017-04-780155

Comprehensive Analysis of the Immunogenomics of Triple-Negative Breast Cancer Brain Metastases From LCCC1419	Routh, ED and Van Swearingen, AED and Sambade, MJ and Vensko, S and McClure, MB and Woodcock, MG and Chai, SJ and	10.3389/fonc.2022.818693
Adaptive immunity and neutralizing antibodies against SARS-CoV-2 variants of concern following vaccination in patients with cancer: the CAPTURE study	Fendler, A and Shepherd, STC and Au, L and Wilkinson, KA and Wu, M and Byrne, F and Cerrone, M and Schmitt, AM and Joh	10.1038/s43018-021-00274-w
Development of a personalized dendritic cell vaccine and single-cell RNA sequencing-guided assessment of its cell type composition	Li, QL and Yang, C and Tian, H and Jiang, JF and Li, P and Zhu, XF and Lei, TJ and Yin, RT and Ding, P and Bai, P and Li, QT	10.1016/j.jcyt.2022.10.013
In vitro and in vivo Evidence on Intra-tumor Injection of Allogeneic Serum for Immunotherapy in a Mouse Model of Colon Cancer	Basirat, E and Dehghan, D and Abbasi, A and Pakravan, N	10.18502/ijaai.v21i5.11042
CD24 (+) MDSC-DCs Induced by CCL5-Deficiency Showed Improved Antitumor Activity as Tumor Vaccines	Huang, L and Ding, ZQ and Zhang, Y	10.1055/s-0042-1743569
Engineered Cell-Membrane-Coated Nanoparticles Directly Present Tumor Antigens to Promote Anticancer Immunity	Jiang, Y and Krishnan, N and Zhou, JR and Chekuri, S and Wei, XL and Kroll, AV and Yu, CL and Duan, YO and Gao, WW and F	10.1002/adma.202001808
A cloning and expression system to probe T-cell receptor specificity and assess functional avidity to neoantigens	Hu, ZT and Anandappa, AJ and Sun, J and Kim, J and Leet, DE and Bozym, DJ and Chen, C and Williams, L and Shukla, SA and	10.1182/blood-2018-04-843763
Characteristics of a Novel Target Antigen against Myeloma Cells for Immunotherapy	Matsushita, M and Saito, S and Yokoe, S and Ichikawa, D and Hattori, Y	10.3390/vaccines8040579
Combining DNA Vaccine and AIDA-1 in Attenuated Salmonella Activates Tumor-Specific CD4(+) and CD8(+) T-cell Responses	Mei, Y and Zhao, LX and Liu, YH and Gong, HL and Song, Y and Lei, L and Zhu, Y and Jin, ZQ and Ma, SB and Hu, B and Sun, Q	10.1158/2326-6066.CIR-16-0240-T
Compromised functionality of monocyte-derived dendritic cells in multiple myeloma patients may limit their use in cancer immunotherapy	Shinde, P and Fernandes, S and Melinkeri, S and Kale, V and Limaye, L	10.1038/s41598-018-23943-w
CD8 T cell function and cross-reactivity explored by stepwise increased peptide-HLA versus TCR affinity	Baumgaertner, P and Schmidt, J and Costa-Nunes, CM and Bordry, N and Guillaume, P and Luescher, I and Speiser, DE and	10.3389/fimmu.2022.973986
BCG-activation of leukocytes is sufficient for the generation of donor-independent innate anti-tumor NK and gamma delta T-cells that can be further expanded in vitro	Esteso, G and Felgueres, MJ and Garcia-Jimenez, AF and Reyburn-Vales, C and Benguria, A and Vazquez, E and Reyburn, HT	10.1080/2162402X.2022.2160094
Fucosylation Enhances the Efficacy of Adoptively Transferred Antigen-Specific Cytotoxic T Lymphocytes	Alatrash, G and Qiao, N and Zhang, M and Zope, M and Perakis, AA and Sukhumalchandra, P and Philips, AV and Garber, HR	10.1158/1078-0432.CCR-18-1527
Identification and characterization of agonist epitopes of the MUC1-C oncoprotein	Jochems, C and Tucker, JA and Vergati, M and Boyerinas, B and Gulley, JL and Schlom, J and Tsang, KY	10.1007/s00262-013-1494-7
Synergy of Immune Checkpoint Blockade with a Novel Synthetic Consensus DNA Vaccine Targeting TERT	Duperret, EK and Wise, MC and Trautz, A and Villarreal, DO and Ferraro, B and Walters, J and Yan, J and Khan, A and Mastell	10.1016/j.ymthe.2017.11.010
Tumor treatment by pHLIP-targeted antigen delivery	DuPont, M and Visca, H and Moshnikova, A and Engelman, DM and Reshetnyak, YK and Andreev, OA	10.3389/fbioe.2022.1082290
Biomarkers for predicting tumor response to PD-1 inhibitors in patients with advanced pancreatic cancer	Qiu, X and Shi, Z and Tong, F and Lu, CC and Zhu, YH and Wang, QL and Gu, Q and Qian, XP and Meng, FY and Liu, BR and Du	10.1080/21645515.2023.2178791
Using Quantitative Seroproteomics to Identify Antibody Biomarkers in Pancreatic Cancer	Jhaveri, DT and Kim, MS and Thompson, ED and Huang, LQ and Sharma, R and Klein, AP and Zheng, L and Le, DT and Laheru	10.1158/2326-6066.CIR-15-0200-T
A Phase I Study of Alpha-1,3-Galactosyltransferase-Expressing Allogeneic Renal Cell Carcinoma Immunotherapy in Patients with Refractory Metastatic Renal Cell Carcinoma	Hahn, AW and Drake, C and Denmeade, SR and Zakharia, Y and Maughan, BL and Kennedy, E and Link, C and Vahanian, N a	10.1634/theoncologist.2019-0599
Targeted Delivery of BZLF1 to DEC205 Drives EBV-Protective Immunity in a Spontaneous Model of EBV-Driven Lymphoproliferative Disease	Ahmed, EH and Brooks, E and Sloan, S and Schlotter, S and Jeney, F and Hale, C and Mao, C and Zhang, XL and McLaughlin,	10.3390/vaccines9060555
Novel TLR2-binding adjuvant induces enhanced T cell responses and tumor eradication	Zom, GG and Willems, MMJHP and Khan, S and van der Sluis, TC and Kleinovink, JW and Camps, MGM and van der Marel, G	10.1186/s40425-018-0455-2
A phase 1 trial extension to assess immunologic efficacy and safety of prime-boost vaccination with VXM01, an oral T cell vaccine against VEGFR2, in patients with advanced pancreatic cancer	Schmitz-Winnenthal, FH and Hohmann, N and Schmidt, T and Podola, L and Friedrich, T and Lubenau, H and Springer, M an	10.1080/2162402X.2017.1303584
Neoantigen vaccination induces clinical and immunologic responses in non-small cell lung cancer patients harboring EGFR mutations	Li, FG and Deng, LG and Jackson, KR and Talukder, AH and Katailiha, AS and Bradley, SD and Zou, QW and Chen, CX and Hu	10.1136/jitc-2021-002531
An immunogenic personal neoantigen vaccine for patients with melanoma	Ott, PA and Hu, ZT and Keskin, DB and Shukla, SA and Sun, J and Bozym, DJ and Zhang, WD and Luoma, A and Giobbie-Hurd	10.1038/nature22991
Safety and efficacy of autologous tumour cell vaccines as a cancer therapeutic to treat solid tumours and haematological malignancies: a meta-analysis protocol for two systematic reviews	Khan, ST and Montroy, J and Forbes, N and Bastin, D and Kennedy, MA and Diallo, JS and Kekre, N and Fergusson, DA and La	10.1136/bmjopen-2019-034714
The prognostic value of peripheral CD4(+)CD25(+) T lymphocytes among early stage and triple negative breast cancer patients receiving dendritic cells-cytokine induced killer cells infusion	Song, QK and Ren, J and Zhou, XN and Wang, XL and Song, GH and Di, LJ and Yu, J and Hobeika, A and Morse, MA and Yuan,	10.18632/oncotarget.5534
Novel IL-15 dendritic cells have a potent immunomodulatory effect in immunotherapy of multiple myeloma	Chu, TH and Vo, MC and Lakshmi, TJ and Ahn, SY and Kim, M and Song, GY and Yang, DH and Ahn, JS and Kim, HJ and Jung, S	10.1016/j.tranon.2022.101413
Fifteen-year follow-up of relapsed indolent non-Hodgkin lymphoma patients vaccinated with tumor-loaded dendritic cells	Fuca, G and Ambrosini, M and Agnelli, L and Brich, S and Sgambelluri, F and Mortarini, R and Pupa, SM and Magni, M and De	10.1136/jitc-2020-002240
Standard radiotherapy but not chemotherapy impairs systemic immunity in non-small cell lung cancer	Yazdi, MT and Schinkelshoek, MS and Loof, NM and Taube, C and Hiemstra, PS and Welters, MJP and van der Burg, SH	10.1080/2162402X.2016.1255393

Addition of anti-estrogen therapy to anti-HER2 dendritic cell vaccination improves regional nodal immune response and pathologic complete response rate in patients with ERpos/HER2(pos) early breast cancer	Lowenfeld, L and Zaheer, S and Oechsle, C and Fracol, M and Datta, J and Xu, SW and Fitzpatrick, E and Roses, RE and Fishe	10.1080/2162402X.2016.1207032
Poly(anhydride) nanoparticles act as effective adjuvants to elicit a persistent immune response	Liu, CX and Shen, QX and Zheng, WW and Lv, Y and Chen, XY and Li, XH and Zhu, QQ and Guo, XL and Ge, RS and Li, C	10.1039/c7ra11891k
HPV-16 E6/E7 DNA tattoo vaccination using genetically optimized vaccines elicit clinical and immunological responses in patients with usual vulvar intraepithelial neoplasia (uVIN): a phase I/II clinical trial	Bakker, NAM and Rotman, J and van Beurden, M and Zijlmans, HJM and van Ruiten, M and Samuels, S and Nuijen, B and Beij	10.1136/jitc-2021-002547
A modified HLA-A*0201-restricted CTL epitope from human oncoprotein (hPEBP4) induces more efficient antitumor responses	Sun, WH and Shi, JY and Wu, J and Zhang, JC and Chen, HB and Li, YY and Liu, SX and Wu, YF and Tian, ZG and Cao, XT and L	10.1038/cmi.2017.155
Initial observations on age, gender, BMI and hypertension in antibody responses to SARS-CoV-2 BNT162b2 vaccine	Pellini, R and Venuti, A and Pimpinelli, F and Abril, E and Blandino, G and Campo, F and Conti, L and De Virgilio, A and De Ma	10.1016/j.eclinm.2021.100928
Expansion of a BDCA1(+)CD14(+) Myeloid Cell Population in Melanoma Patients May Attenuate the Efficacy of Dendritic Cell Vaccines	Bakdash, G and Buschow, SI and Gorris, MAJ and Halilovic, A and Hato, SV and Skold, AE and Schreibelt, G and Sittig, SP an	10.1158/0008-5472.CAN-15-1695
Mycolicibacterium brumae is a Safe and Non-Toxic Immunomodulatory Agent for Cancer Treatment	Bach-Griera, M and Campo-Perez, V and Barbosa, S and Traserra, S and Guallar-Garrido, S and Moya-Anderico, L and Herrer	10.3390/vaccines8020198
Peptide Vaccination Against PD-L1 With IO103 a Novel Immune Modulatory Vaccine in Multiple Myeloma: A Phase I First-in-Human Trial	Jorgensen, NG and Klausen, U and Grauslund, JH and Helleberg, C and Aagaard, TG and Do, TH and Ahmad, SM and Olsen,	10.3389/fimmu.2020.595035
IFN-gamma treatment protocol for MHC-I-lo/PD-L1(+)pancreatic tumor cells selectively restores their TAP-mediated presentation competence and CD8 T-cell priming potential	Stifter, K and Krieger, J and Ruths, L and Gout, J and Mulaw, M and Lechel, A and Kleger, A and Seufferlein, T and Wagner, M	10.1136/jitc-2020-000692
DNA and modified vaccinia Ankara prime-boost vaccination generates strong CD8(+) T cell responses against minor histocompatibility antigen HA-1	Eldershaw, SA and Pearce, H and Inman, CF and Piper, KP and Abbotts, B and Stephens, C and Nicol, S and Croft, W and Po	10.1111/bjh.17495
Hybrid membrane-coated nanosuspensions for multi-modal anti-glioma therapy via drug and antigen delivery	Hao, WY and Cui, YX and Fan, YY and Chen, MY and Yang, GB and Wang, YL and Yang, MY and Li, ZP and Gong, W and Yang,	10.1186/s12951-021-01110-0
HPV16 E6-specific T cell response and HLA-A alleles are related to the prognosis of patients with cervical cancer	Cai, HC and Feng, YN and Fan, PW and Guo, YP and Kuerban, G and Chang, C and Yao, X and Peng, YC and Wang, RZ	10.1186/s13027-021-00395-y
Vaccination with poly (IC: LC) and peptide-pulsed autologous dendritic cells in patients with pancreatic cancer	Mehrotra, S and Britten, CD and Chin, S and Garrett-Mayer, E and Cloud, CA and Li, ML and Scurti, G and Salem, ML and Nel	10.1186/s13045-017-0459-2
HBVsvp-Pulsed Dendritic Cell Immunotherapy Induces Th1 Polarization and Hepatitis B Virus-Specific Cytotoxic T Lymphocytes Production	Farag, MMS and Suef, RA and Al-Toukhy, GM and Selim, MA and Elbahnasawy, MA and El Sharkawy, N and Ezzat, S and Sheb	10.2147/IDR.S265681
mRNA-transfected dendritic cell vaccine in combination with metronomic cyclophosphamide as treatment for patients with advanced malignant melanoma	Borch, TH and Engell-Noerregaard, L and Zeeberg Iversen, T and Ellebaek, E and Met, O and Hansen, M and Andersen, MH a	10.1080/2162402X.2016.1207842
Large-scale manufacturing and characterization of CMV-CD19CAR T cells	Wang, XL and Urak, R and Walter, M and Guan, M and Han, TX and Vyas, V and Chien, SH and Gittins, B and Clark, MC and M	10.1136/jitc-2021-003461
NY-ESO-1 Vaccination in Combination with Decitabine Induces Antigen-Specific T-lymphocyte Responses in Patients with Myelodysplastic Syndrome	Griffiths, EA and Srivastava, P and Matsuzaki, J and Brumberger, Z and Wang, ES and Kocent, J and Miller, A and Roloff, GW a	10.1158/1078-0432.CCR-17-1792
Co-delivery of the NKT agonist -galactosylceramide and tumor antigens to cross-priming dendritic cells breaks tolerance to self-antigens and promotes antitumor responses	Ghinnagow, R and De Meester, J and Cruz, LJ and Aspord, C and Corgnac, S and Macho-Fernandez, E and Soulard, D and Fo	10.1080/2162402X.2017.1339855
Therapeutic Immune Modulation against Solid Cancers with Intratumoral Poly-ICLC: A Pilot Trial	Kyi, C and Roudko, V and Sabado, R and Saenger, Y and Loging, W and Mandeli, J and Thin, TH and Lehrer, D and Donovan,	10.1158/1078-0432.CCR-17-1866
Neoantigen vaccine generates intratumoral T cell responses in phase Ib glioblastoma trial	Keskin, DB and Anandappa, AJ and Sun, J and Tirosh, I and Mathewson, ND and Li, SQ and Oliveira, G and Giobbie-Hurder, A	10.1038/s41586-018-0792-9
Dendritic Cells Pulsed with Leukemia Cell-Derived Exosomes More Efficiently Induce Antileukemic Immunities	Yao, Y and Wang, C and Wei, W and Shen, C and Deng, XH and Chen, LJ and Ma, LY and Hao, SG	10.1371/journal.pone.0091463
A Novel Identified Necroptosis-Related Risk Signature for Prognosis Prediction and Immune Infiltration Indication in Acute Myeloid Leukemia Patients	Sun, Y and Wang, RH and Xie, SF and Wang, YL and Liu, H	10.3390/genes13101837
Lipid Accumulation in Peripheral Blood Dendritic Cells and Anticancer Immunity in Patients with Lung Cancer	Arai, R and Soda, S and Okutomi, T and Morita, H and Ohmi, F and Funakoshi, T and Takemasa, A and Ishii, Y	10.1155/2018/5708239
Locally Delivered CD40 Agonist Antibody Accumulates in Secondary Lymphoid Organs and Eradicates Experimental Disseminated Bladder Cancer	Sandin, LC and Orlova, A and Gustafsson, E and Ellmark, P and Tolmachev, V and Totterman, TH and Mangsbo, SM	10.1158/2326-6066.CIR-13-0067
MART-1 peptide vaccination plus IMP321 (LAG-3Ig fusion protein) in patients receiving autologous PBMCs after lymphodepletion: results of a Phase I trial	Romano, E and Michielin, O and Voelter, V and Laurent, J and Bichat, H and Stravodimou, A and Romero, P and Speiser, DE	10.1186/1479-5876-12-97
Pattern recognition receptor expression and maturation profile of dendritic cell subtypes in human tonsils and lymph nodes	Askmyr, D and Abolhalaj, M and Jimenez, DG and Greiff, L and Lindstedt, M and Lundberg, K	10.1016/j.humimm.2021.08.007
T cells of colorectal cancer patients' stimulated by neoantigenic and cryptic peptides better recognize autologous tumor cells	Schwarz, S and Schmitz, J and Loffler, MW and Ghosh, M and Rammensee, HG and Olshvang, E and Markel, M and Mockel-T	10.1136/jitc-2022-005651
Melanoma antigen family A4 protein produced by transgenic silkworms induces antitumor immune responses	Motokawa, Y and Kokubo, M and Kuwabara, N and Tatematsu, KI and Sezutsu, H and Takahashi, H and Sakakura, K and Chik	10.3892/etm.2018.5703

Combined TCR Repertoire Profiles and Blood Cell Phenotypes Predict Melanoma Patient Response to Personalized Neoantigen Therapy plus Anti-PD-1	Poran, A and Scherer, J and Bushway, ME and Besada, R and Balogh, KN and Wanamaker, A and Williams, RG and Prabhaka	10.1016/j.xcrm.2020.100141
Combination vaccine based on citrullinated vimentin and enolase peptides induces potent CD4-mediated anti-tumor responses	Brentville, VA and Metheringham, RL and Daniels, I and Atabani, S and Symonds, P and Cook, KW and Vankemmelbeke, M a	10.1136/jitc-2020-000560
6-Thioguanine-loaded polymeric micelles deplete myeloid-derived suppressor cells and enhance the efficacy of T cell immunotherapy in tumor-bearing mice	Jeanbart, L and Kourtis, IC and van der Vlies, AJ and Swartz, MA and Hubbell, JA	10.1007/s00262-015-1702-8
Heparanase and cancer progression: New directions, new promises	Arvatz, G and Weissmann, M and Ilan, N and Vlodavsky, I	10.1080/21645515.2016.1171442
Vaccine-Induced Tumor Necrosis Factor-Producing T Cells Synergize with Cisplatin to Promote Tumor Cell Death	van der Sluis, TC and van Duikeren, S and Huppelschoten, S and Jordanova, ES and Nejad, EB and Sloots, A and Boon, L and	10.1158/1078-0432.CCR-14-2142
Combined proteomics/miRNomics of dendritic cell immunotherapy-treated glioblastoma patients as a screening for survival-associated factors	Erhart, F and Hackl, M and Hahne, H and Buchroithner, J and Meng, C and Klingenbrunner, S and Reitermaier, R and Fischhu	10.1038/s41541-019-0149-x
Selective colorectal cancer cell lysates enhance the immune function of mature dendritic cells in vitro	Chen, LH and Meng, D and Zhao, L and Liu, R and Bai, PS and Wang, L and Xiao, JX	10.3892/mmr.2014.2930
Prognostic impact of high levels of circulating plasmacytoid dendritic cells in breast cancer	Bailur, JK and Gueckel, B and Pawelec, G	10.1186/s12967-016-0905-x
Antitumor Activity of Lentivirus-mediated Interleukin-12 Gene Modified Dendritic Cells in Human Lung Cancer in Vitro	Ali, HAA and Di, J and Mei, W and Zhang, YC and Li, Y and Du, ZW and Zhang, GZ	10.7314/APJCP.2014.15.2.611
The interplay between cholesterol (and other metabolic conditions) and immune-checkpoint immunotherapy: shifting the concept from the "inflamed tumor" to the "inflamed patient"	Bersanelli, M and Cortellini, A and Buti, S	10.1080/21645515.2020.1852872
Immune Response Generated With the Administration of Autologous Dendritic Cells Pulsed With an Allogenic Tumoral Cell-Lines Lysate in Patients With Newly Diagnosed Diffuse Intrinsic Pontine Glioma	Benitez-Ribas, D and Cabezon, R and Florez-Grau, G and Molero, MC and Puerta, P and Guillen, A and Paco, S and Carcabos	10.3389/fonc.2018.00127
Primary Human Dendritic Cells and Whole-Blood Based Assays to Evaluate Immuno-Modulatory Properties of Heat-Killed Commensal Bacteria	Norton, JE and Kommineni, S and Akrivoulis, P and Gutierrez, DA and Hazuda, DJ and Swaminathan, G	10.3390/vaccines9030225
Melanoma cell lysate induces CCR7 expression and in vivo migration to draining lymph nodes of therapeutic human dendritic cells	Gonzalez, FE and Ortiz, C and Reyes, M and Dutzan, N and Patel, V and Pereda, C and Gleisner, MA and Lopez, MN and Gutk	10.1111/imm.12264
The power of combining adoptive cell therapy (ACT) and pathogen-boosted vaccination to treat solid tumors	Zander, R and Cui, WG	10.1080/21645515.2017.1349046
Targeting the tumor microenvironment to improve natural killer cell-based immunotherapies: On being in the right place at the right time, with resilience	Murray, S and Lundqvist, A	10.1080/21645515.2015.1096458
CD8(+) T Cell Senescence: Lights and Shadows in Viral Infections, Autoimmune Disorders and Cancer	Tedeschi, V and Paldino, G and Kunkl, M and Paroli, M and Sorrentino, R and Tuosto, L and Fiorillo, MT	10.3390/ijms23063374
Fungal mannosylation enhances human papillomavirus 16 E7 therapeutic immunity against TC-1 tumors	Wang, ZL and Wei, CH and Zhang, YJ and Wang, W and Zhou, Z and Xiao, GF	10.3892/or.2017.6083
Self-Assembling Peptide Epitopes as Novel Platform for Anticancer Vaccination	Rad-Malekshahi, M and Fransen, MF and Krawczyk, M and Mansourian, M and Bourajjaj, M and Chen, J and Ossendorp, F and	10.1021/acs.molpharmaceut.6b01003
A simple, clinically relevant therapeutic vaccine shows long-term protection in an aggressive, delayed-treatment B lymphoma model	Pradhan, P and Leleux, J and Liu, JY and Roy, K	10.1172/jci.insight.92522
BNT162b2 COVID-19 vaccine is significantly less effective in patients with hematologic malignancies	Tzarfati, KH and Gutwein, O and Apel, A and Rahimi-Levene, N and Sadovnik, M and Harel, L and Benveniste-Levkovitz, P and	10.1002/ajh.26284
The combined molecular adjuvant CASAC enhances the CD8+T cell response to a tumor-associated self-antigen in aged, immunosenescent mice	Tye, GJ and Ioannou, K and Amofah, E and Quartey-Papafio, R and Westrop, SJ and Krishnamurthy, P and Noble, A and Harris	10.1186/s12979-015-0033-0
Comparison of four methods of colon cancer cell lysates preparation for ex vivo maturation of dendritic cells	Roufarshbaf, M and Esmaeil, N and Akbari, V	10.4103/1735-5362.329925
The effects of gemcitabine and capecitabine combination chemotherapy and of low-dose adjuvant GM-CSF on the levels of myeloid-derived suppressor cells in patients with advanced pancreatic cancer	Annels, NE and Shaw, VE and Gabitass, RF and Billingham, L and Corrie, P and Eatock, M and Valle, J and Smith, D and Wad	10.1007/s00262-013-1502-y
Lactobacillus buchneri S-layer as carrier for an Ara h 2-derived peptide for peanut allergen-specific immunotherapy	Anzengruber, J and Bublin, M and Bonisch, E and Janesch, B and Tscheppe, A and Braun, ML and Varga, EM and Hafner, C an	10.1016/j.molimm.2017.02.005
Identification of biomarkers for personalized peptide vaccination in 2,588 cancer patients	Suekane, S and Yutani, S and Yamada, A and Sasada, T and Matsueda, S and Takamori, S and Toh, U and Kawano, K and Yos	10.3892/ijo.2020.5019
Development of an 8-color antibody panel for functional phenotyping of human CD8+cytotoxic T cells from peripheral blood mononuclear cells	Patel, T and Cunningham, A and Holland, M and Daley, J and Lazo, S and Hodi, FS and Severgnini, M	10.1007/s10616-017-0106-3
Multi-Dimensional Flow Cytometry Analyses Reveal a Dichotomous Role for Nitric Oxide in Melanoma Patients Receiving Immunotherapy	Garg, SK and Ott, MJ and Mostofa, AGM and Chen, ZH and Chen, YA and Kroeger, J and Cao, BW and Mailloux, AW and Agrav	10.3389/fimmu.2020.00164
Survival of metastatic melanoma patients after dendritic cell vaccination correlates with expression of leukocyte phosphatidylethanolamine-binding protein 1/Raf kinase inhibitory protein	Buschow, SI and Ramazzotti, M and Reinieren-Beeren, IMJ and Heinzerling, LM and Westdorp, H and Stefanini, I and Beltram	
Human papilloma virus-specific T cells can be generated from naive T cells for use as an immunotherapeutic strategy for immunocompromised patients	McCormack, SE and Cruz, CRY and Wright, KE and Powell, AB and Lang, HL and Trimble, C and Keller, MD and Fuchs, E and	10.1016/j.jcyt.2017.11.010

Proteogenomic discovery of neoantigens facilitates personalized multi-antigen targeted T cell immunotherapy for brain tumors	Rivero-Hinojosa, S and Grant, M and Panigrahi, A and Zhang, HZ and Caisova, V and Bollard, CM and Rood, BR	10.1038/s41467-021-26936-y
Frequencies of an Immunogenic HER-2/neu Epitope of CD8+T Lymphocytes Predict Favorable Clinical Outcomes in Prostate Cancer	Goulielmaki, M and Stokidis, S and Anagnostou, T and Voutsas, IF and Gritzapis, AD and Baxevanis, CN and Fortis, SP	10.3390/ijms24065954
Evaluation of Selected Immunomodulatory Glycoproteins as an Adjunct to Cancer Immunotherapy	Sekhon, BK and Roubin, RH and Li, YM and Devi, PB and Nammi, S and Fan, K and Sze, DMY	10.1371/journal.pone.0146881
Vaccination with LAG-3Ig (IMP321) and Peptides Induces Specific CD4 and CD8 T-Cell Responses in Metastatic Melanoma Patients-Report of a Phase I/IIa Clinical Trial	Legat, A and Maby-El Hajjami, H and Baumgaertner, P and Cagnon, L and Maillard, SA and Geldhof, C and Iancu, EM and Leb	10.1158/1078-0432.CCR-15-1212
The oncogenic fusion protein DNAJB1-PRKACA can be specifically targeted by peptide-based immunotherapy in fibrolamellar hepatocellular carcinoma	Bauer, J and Kohler, N and Maringer, Y and Bucher, P and Bilich, T and Zwick, M and Dicks, S and Nelde, A and Dubbelaar, M	10.1038/s41467-022-33746-3
Engineered red blood cells as an off-the-shelf allogeneic anti-tumor therapeutic	Zhang, XQ and Luo, MY and Dastagir, SR and Nixon, M and Khamhoung, A and Schmidt, A and Lee, A and Subbiah, N and Mc	10.1038/s41467-021-22898-3
Potential Utility of Induced Translocation of Engineered Bacteria as a Therapeutic Agent for Mounting a Personalized Neoantigen-Based Tumor Immune Response	Luengo-Gil, G and Conesa-Zamora, P	10.1002/gch2.202100051
A Phase I trial using local regional treatment, nonlethal irradiation, intratumoral and systemic polyinosinic-polycytidylic acid polylysine carboxymethylcellulose to treat liver cancer: in search of the abscopal effect	de la Torre, AN and Contractor, S and Castaneda, I and Cathcart, CS and Razdan, D and Klyde, D and Kisza, P and Gonzales,	10.2147/JHC.S136652
Identification of Novel HLA Class II-Restricted Neoantigens Derived from Driver Mutations	Iizumi, S and Ohtake, J and Murakami, N and Kouro, T and Kawahara, M and Isoda, F and Hamana, H and Kishi, H and Nakar	10.3390/cancers11020266
Soluble HLA peptidome of pleural effusions is a valuable source for tumor antigens	Khazan-Kost, S and Cafri, G and Kadosh, DM and Mooshayef, N and Chatterji, S and Dominissini, D and Manor, S and Zisser	10.1136/jitc-2021-003733
A pilot Phase I study combining peptide-based vaccination and NGR-hTNF vessel targeting therapy in metastatic melanoma	Parmiani, G and Pilla, L and Corti, A and Doglioni, C and Cimminiello, C and Bellone, M and Parolini, D and Russo, V and Ca	10.4161/21624011.2014.963406
Mycobacteria-Specific T Cells May Be Expanded From Healthy Donors and Are Near Absent in Primary Immunodeficiency Disorders	Patel, S and Lang, HL and Sani, G and Freeman, AF and Leiding, J and Hanley, PJ and Cruz, CR and Grant, M and Wang, YF ar	10.3389/fimmu.2019.00621
In Vivo Administration of Recombinant Human Granulocyte Colony-Stimulating Factor Increases the Immune Effectiveness of Dendritic Cell-Based Cancer Vaccination	Shimodaira, S and Yanagisawa, R and Koya, T and Hirabayashi, K and Higuchi, Y and Sakamoto, T and Togi, M and Kato, T an	10.3390/vaccines7030120
The Application of Cytidyl Guanosyl Oligodeoxynucleotide Can Affect the Antitumor Immune Response Induced by a Combined Protocol of Cryoablation and Dendritic Cells in Lewis Lung Cancer Model	Zhang, M and Yin, TQ and Lu, Y and Feng, HS	10.12659/MSM.898194
Therapy of genomic unstable solid tumours (WHO grade 3/4?in clinical stage III/IV using individualised neoantigen tumour peptides-INP trial (individualised neoantigen tumour peptides immunotherapy): study protocol for an open-label, non-randomised, prospective, single-arm trial	Wang, L and Tang, JX and Chen, X and Zhao, J and Tang, WY and Liao, B and Nian, WQ	10.1136/bmjopen-2021-055742
Chimeric antigen receptor T cells targeting HERV-K inhibit breast cancer and its metastasis through downregulation of Ras	Zhou, FL and Krishnamurthy, J and Wei, YC and Li, M and Hunt, K and Johanning, GL and Cooper, LJN and Wang-Johanning, I	10.1080/2162402X.2015.1047582
Persistent hepatitis C viral replication despite priming of functional CD8(+) T cells by combined therapy with a vaccine and a direct-acting antiviral	Callendret, B and Eccleston, HB and Satterfield, W and Capone, S and Folgori, A and Cortese, R and Nicosia, A and Walker,	10.1002/hep.28309
Immune responses against autologous tumor and human papilloma virus in lymph nodes from patients with penile cancer	Zhang, L and Zirakzadeh, AA and Rosvall, J and Hedlund, M and Hu, PS and Riklund, K and Sherif, A and Winqvist, O	10.4111/icu.20200116
Liquid biopsy-based identification of prognostic and immunotherapeutically relevant gene signatures in lower grade glioma	Wu, CW and Long, WY and Qin, CY and Wang, XY and Li, YZ and Xiao, K and Li, Y and Liu, Q	10.1186/s40537-023-00686-8
Low-molecular-weight polysaccharides from Agaricus blazei Murrill modulate the Th1 response in cancer immunity	Jiang, LY and Yu, ZP and Lin, Y and Cui, LR and Yao, SJ and Lv, LY and Liu, JC	10.3892/ol.2018.7794
Broad immune activation underlies shared set point signatures for vaccine responsiveness in healthy individuals and disease activity in patients with lupus	Kotliarov, Y and Sparks, R and Martins, AJ and Mule, MP and Lu, Y and Goswami, M and Kardava, L and Banchereau, R and Pa	10.1038/s41591-020-0769-8
Mutational landscape of the transcriptome offers putative targets for immunotherapy of myeloproliferative neoplasms	Schischlik, F and Jager, R and Rosebrock, F and Hug, E and Schuster, M and Holly, R and Fuchs, E and Feenstra, JDM and Bo	10.1182/blood.2019000519
Preclinical models for prediction of immunotherapy outcomes and immune evasion mechanisms in genetically heterogeneous multiple myeloma	Larrayoz, M and Garcia-Barchino, MJ and Celay, J and Etxebeste, A and Jimenez, M and Perez, C and Ordonez, R and Cobale	10.1038/s41591-022-02178-3
Local and systemic XAGE-1b-specific immunity in patients with lung adenocarcinoma	Yazdi, MT and Loof, NM and Franken, KLMC and Taube, C and Oostendorp, J and Hiemstra, PS and Welters, MJP and van der	10.1007/s00262-015-1716-2
Tim-3/galectin-9 pathway and mMDSC control primary and secondary resistances to PD-1 blockade in lung cancer patients	Limagne, E and Richard, C and Thibaudin, M and Fumet, JD and Truntzer, C and Lagrange, A and Favier, L and Coudert, B and	10.1080/2162402X.2018.1564505
Vaccination with autologous dendritic cells loaded with autologous tumor lysate or homogenate combined with immunomodulating radiotherapy and/or preleukapheresis IFN-alpha in patients with metastatic melanoma: a randomised "proof-of-principle" phase II study	de Rosa, F and Ridolfi, L and Ridolfi, R and Gentili, G and Valmorri, L and Nanni, O and Petrini, M and Fiammenghi, L and Gra	10.1186/1479-5876-12-209
Invariant NKT cells are resistant to circulating CD15(+) myeloid-derived suppressor cells in patients with head and neck cancer	Horinaka, A and Sakurai, D and Ihara, F and Makita, Y and Kunii, N and Motohashi, S and Nakayama, T and Okamoto, Y	10.1111/cas.12866
Identifying predictive biomarkers of CIMAvaxEGF success in non-small cell lung cancer patients	Lorenzo-Luaces, P and Sanchez, L and Saavedra, D and Crombet, T and Van der Elst, W and Alonso, A and Molenberghs, G a	10.1186/s12885-020-07284-4

Dual effect of DLBCL-derived EXOs in lymphoma to improve DC vaccine efficacy in vitro while favor tumorgenesis in vivo	Chen, ZZ and You, LS and Wang, L and Huang, XB and Liu, H and Wei, JY and Zhu, L and Qian, WB	10.1186/s13046-018-0863-7
Human CD141(+) dendritic cells (cDC1) are impaired in patients with advanced melanoma but can be targeted to enhance anti-PD-1 in a humanized mouse model	Lee, YS and O'Brien, LJ and Walpole, CM and Pearson, FE and Leal-Rojas, IM and Masterman, KA and Atkinson, V and Barbou	10.1136/jitc-2020-001963
Mass cytometry detects H3.3K27M-specific vaccine responses in diffuse midline glioma	Mueller, S and Taitt, JM and Villanueva-Meyer, JE and Bonner, ER and Nejo, T and Lulla, RR and Goldman, S and Banerjee, A a	10.1172/JCI140378
Expression of placenta-specific 1 and its potential for eliciting anti-tumor helper T-cell responses in head and neck squamous cell carcinoma	Hayashi, R and Nagato, T and Kumai, T and Ohara, K and Ohara, M and Ohkuri, T and Hirata-Nozaki, Y and Harabuchi, S and	10.1080/2162402X.2020.1856545
Phase I clinical trial of autologous NK cell therapy using novel expansion method in patients with advanced digestive cancer	Sakamoto, N and Ishikawa, T and Kokura, S and Okayama, T and Oka, K and Ideno, M and Sakai, F and Kato, A and Tanabe, M	10.1186/s12967-015-0632-8
Enhancing the treatment effect on melanoma by heat shock protein 70-peptide complexes purified from human melanoma cell lines	Gao, YW and Gao, WS and Chen, X and Cha, NE and Wang, XL and Jia, XD and Wang, BP and Ren, M and Ren, J	10.3892/or.2016.4947
Persistence of long-lived plasma cells and humoral immunity in individuals responding to CD19-directed CAR T-cell therapy	Bhoj, VG and Arhontoulis, D and Wertheim, G and Capobianchi, J and Callahan, CA and Ellebrecht, CT and Obstfeld, AE and	10.1182/blood-2016-01-694356
Protective effect of cytotoxic T lymphocytes targeting HTLV-1 bZIP factor	Sugata, K and Yasunaga, J and Mitobe, Y and Miura, M and Miyazato, P and Kohara, M and Matsuoka, M	10.1182/blood-2015-04-641118
Cytotoxicity of Human Endogenous Retrovirus K-Specific T Cells toward Autologous Ovarian Cancer Cells	Rycaj, K and Plummer, JB and Yin, BN and Li, M and Garza, J and Radvanyi, L and Ramondetta, LM and Lin, K and Johanning,	10.1158/1078-0432.CCR-14-0388
ILC2-modulated T cell-to-MDSC balance is associated with bladder cancer recurrence	Chevalier, MF and Trabanelli, S and Racle, J and Salome, B and Cesson, V and Gharbi, D and Bohner, P and Domingos-Perei	10.1172/JCI89717
Anti-prostate cancer effects of CTL cell induction in vitro by recombinant adenovirus mediated PSMA/4-1 BBL dendritic cells: an immunotherapy study	Sui, CG and Wu, D and Meng, FD and Yang, MH and Jiang, YH	10.4238/2015.June.29.14
Understanding CD8(+) T-cell responses toward the native and alternate HLA-A*02:01-restricted WT1 epitope	Nguyen, THO and Tan, ACL and Xiang, SD and Goubier, A and Harland, KL and Clemens, EB and Plebanski, M and Kedzierska	10.1038/cti.2017.4
Breast cancer is marked by specific, Public T-cell receptor CDR3 regions shared by mice and humans	Gordin, M and Philip, H and Zilberberg, A and Gidoni, M and Margalit, R and Clouser, C and Adams, K and Vigneault, F and Co	10.1371/journal.pcbi.1008486
Carcinogenesis of renal cell carcinoma reflected in HLA ligands: A novel approach for synergistic peptide vaccination design	Klatt, MG and Kowalewski, DJ and Schuster, H and Di Marco, M and Hennenlotter, J and Stenzl, A and Rammensee, HG and S	10.1080/2162402X.2016.1204504
Immunomodulatory Effects of Newcastle Disease Virus AF2240 Strain on Human Peripheral Blood Mononuclear Cells	Lam, HY and Yusoff, K and Yeap, SK and Subramani, T and Abd-Aziz, S and Omar, AR and Alitheen, NB	10.7150/ijms.8170
F-19-perfluorocarbon-labeled human peripheral blood mononuclear cells can be detected &ITin vivo&IT using clinical MRI parameters in a therapeutic cell setting	Fink, C and Gaudet, JM and Fox, MS and Bhatt, S and Viswanathan, S and Smith, M and Chin, J and Foster, PJ and Dekaban,	10.1038/s41598-017-19031-0
Vaccine-induced ICOS(+)CD38(+) circulating Tfh are sensitive biosensors of age-related changes in inflammatory pathways	Herati, RS and Silva, LV and Vella, LA and Muselman, A and Alanio, C and Bengsch, B and Kurupati, RK and Kannan, S and M	10.1016/j.xcrm.2021.100262
Nano-pulse stimulation induces potent immune responses, eradicating local breast cancer while reducing distant metastases	Guo, SQ and Jing, Y and Burcus, NI and Lassiter, BP and Tanaz, R and Heller, R and Beebe, SJ	10.1002/ijc.31071
Novel immunomodulatory properties of low dose cytarabine entrapped in a mannosylated cationic liposome	Martel, AL and Fraleigh, NL and Picard, E and Lewicky, JD and Pawelec, G and Lee, H and Ma, GW and Mousavifar, L and Roy	10.1016/j.ijpharm.2021.120849
A phase II trial of autologous dendritic cell vaccination and radiochemotherapy following fluorescence-guided surgery in newly diagnosed glioblastoma patients	Inoges, S and Tejada, S and de Cerio, ALD and Perez-Larraya, JG and Espinos, J and Idoate, MA and Dominguez, PD and de E	10.1186/s12967-017-1202-z
Preexisting Levels of CD4 T Cells Expressing PD-1 Are Related to Overall Survival in Prostate Cancer Patients Treated with Ipilimumab	Kwek, SS and Lewis, J and Zhang, L and Weinberg, V and Greaney, SK and Harzstark, AL and Lin, AM and Ryan, CJ and Small,	10.1158/2326-6066.CIR-14-0227
First-in-human study of TK-positive oncolytic vaccinia virus delivered by adipose stromal vascular fraction cells	Minev, BR and Lander, E and Feller, JF and Berman, M and Greenwood, BM and Minev, I and Santidrian, AF and Nguyen, D an	10.1186/s12967-019-2011-3
Pilot trial of K562/GM-CSF whole-cell vaccination in MDS patients	Robinson, TM and Prince, GT and Thoburn, C and Warlick, E and Ferguson, A and Kasamon, YL and Borrello, IM and Hess, A a	10.1080/10428194.2018.1443449
Viral Molecular Mimicry Influences the Antitumor Immune Response in Murine and Human Melanoma	Chiaro, J and Kasanen, HHE and Whalley, T and Capasso, C and Gronholm, M and Feola, S and Peltonen, K and Hamdan, F a	10.1158/2326-6066.CIR-20-0814
Endogenous TLR2 ligand embedded in the catalytic region of human cysteinyl-tRNA synthetase 1	Cho, S and Kim, SB and Lee, Y and Song, EC and Kim, U and Kim, HY and Suh, JH and Goughnour, PC and Kim, Y and Yoon, I	10.1136/jitc-2019-000277
Evaluation of Seropositivity Following BNT162b2 Messenger RNA Vaccination for SARS-CoV-2 in Patients Undergoing Treatment for Cancer	Massarweh, A and Eliakim-Raz, N and Stemmer, A and Levy-Barda, A and Yust-Katz, S and Zer, A and Benouaich-Amiel, A an	10.1001/jamaoncol.2021.2155
Antitumor effect of recombinant Mycobacterium smegmatis expressing MAGEA3 and SSX2 fusion proteins	Jian, W and Li, X and Kang, J and Lei, YF and Bai, YL and Xue, Y	10.3892/etm.2018.6425
Regulatory T cells, inherited variation, and clinical outcome in epithelial ovarian cancer	Knutson, KL and Maurer, MJ and Preston, CC and Moysich, KB and Goergen, K and Hawthorne, KM and Cunningham, JM and	10.1007/s00262-015-1753-x
Shared Immunogenic Poly-Epitope Frameshift Mutations in Microsatellite Unstable Tumors	Roudko, V and Bozkus, CC and Orfanelli, T and McClain, CB and Carr, C and O'Donnell, T and Chakraborty, L and Samstein,	10.1016/j.cell.2020.11.004
Vaccination with autologous myeloblasts admixed with GM-K562 cells in patients with advanced MDS or AML after allogeneic HSCT	Ho, VT and Kim, HT and Bavli, N and Mihm, M and Pozdnyakova, O and Piesche, M and Daley, H and Reynolds, C and Souder	10.1182/bloodadvances.2017009084
Naltrexone Inhibits IL-6 and TNF alpha Production in Human Immune Cell Subsets following Stimulation with Ligands for Intracellular Toll-Like Receptors	Cant, R and Dalgleish, AG and Allen, RL	10.3389/fimmu.2017.00809

Cells to prevent/treat relapse following allogeneic stem cell transplantation	Dietz, AC and Wayne, AS	10.1182/asheducation-2017.1.708
Precancer Atlas to Drive Precision Prevention Trials	Spira, A and Yurgelun, MB and Alexandrov, L and Rao, A and Bejar, R and Polyak, K and Giannakis, M and Shilatifard, A and F	10.1158/0008-5472.CAN-16-2346
Utilizing the nanosecond pulse technique to improve antigen intracellular delivery and presentation to treat tongue squamous cell carcinoma	Gao, C and Zhang, XX and Chen, J and Zhao, JY and Liu, YM and Zhang, J and Wang, J	10.4317/medoral.22227
TriMix and tumor antigen mRNA electroporated dendritic cell vaccination plus ipilimumab: link between T-cell activation and clinical responses in advanced melanoma	De Keersmaecker, B and Claerhout, S and Carrasco, J and Bar, I and Corthals, J and Wilgenhof, S and Neyns, B and Thielem	10.1136/jitc-2019-000329
Unexpected neurologic complications following a novel lymphoma treatment 'expected' to give rise to neurologic toxicity	Kersten, MJ and van Ettekoven, CN and Heijink, DM	10.1136/bcr-2019-229946
CAR-T Cells with Phytohemagglutinin (PHA) Provide Anti-Cancer Capacity with Better Proliferation, Rejuvenated Effector Memory, and Reduced Exhausted T Cell Frequencies	Gulden, G and Sert, B and Teymur, T and Ay, Y and Tiryaki, NN and Mishra, AK and Ovali, E and Tarhan, N and Tastan, C	10.3390/vaccines11020313
PD-1 blockade in subprimed CD8 cells induces dysfunctional PD-1(+)CD38(hi) cells and anti-PD-1 resistance	Verma, V and Shrimalim, RK and Ahmadm, S and Dai, WJ and Wang, H and Lu, SM and Nandrelm, R and Gaur, P and Lopez,	10.1038/s41590-019-0441-y
CAR T-cell therapy for pancreatic cancer	DeSelm, CJ and Tano, ZE and Varghese, AM and Adusumilli, PS	10.1002/jso.24627
SARS-CoV-2-specific T cells are rapidly expanded for therapeutic use and target conserved regions of the membrane protein	Keller, MD and Harris, KM and Jensen-Wachspress, MA and Kankate, V and Lang, HL and Lazarski, CA and Durkee-Shock, J a	10.1182/blood.2020008488
Modified dendritic cell-derived exosomes activate both NK cells and T cells through the NKG2D/NKG2D-L pathway to kill CML cells with or without T315I mutation	Du, ZY and Huang, ZL and Chen, X and Jiang, GY and Peng, YH and Feng, WL and Huang, NS	10.1186/s40164-022-00289-8
Anti-Gr-1 Antibody Provides Short-Term Depletion of MDSC in Lymphodepleted Mice with Active-Specific Melanoma Therapy	Rose, P and van den Engel, NK and Kovacs, JR and Hatz, RA and Boon, L and Winter, H	10.3390/vaccines10040560
Pancreatic cancer counterattack: MUC4 mediates Fas-independent apoptosis of antigen-specific cytotoxic T lymphocyte	Zhu, Y and Zhang, JJ and Liang, WB and Zhu, R and Wang, B and Miao, Y and Xu, ZK	10.3892/or.2014.3016
IL-7 signaling imparts polyfunctionality and stemness potential to CD4(+) T cells	Ding, ZC and Liu, CF and Cao, Y and Habtetsion, T and Kuczma, M and Pi, WH and Kong, H and Cacan, E and Greer, SF and C	10.1080/2162402X.2016.1171445
RNA Virus Gene Signatures Detected in Patients With Cardiomyopathy After Chemotherapy; A Pilot Study	Varkoly, K and Tan, SY and Beladi, R and Fonseca, D and Zanetti, IR and Kraberger, S and Shah, CT and Yaron, JR and Zhang,	10.3389/fcvm.2022.821162
CIMT 2017: Anniversary symposium - Report on the 15th CIMT Annual Meeting of the Association for Cancer Immunotherapy	Kranz, LM and Beck, JD and Grunwitz, C and Hotz, C and Vormehr, M and Diken, M	10.1080/21645515.2017.1340138
The cytotoxic molecule granulysin is capable of inducing either chemotaxis or fugetaxis in dendritic cells depending on maturation: a role for V delta 2(+) gamma delta T cells in the modulation of immune response to tumour?	Sparrow, EL and Fowler, DW and Fenn, J and Caron, J and Copier, J and Dalgleish, AG and Bodman-Smith, MD	10.1111/imm.13248
CyTOF analysis identifies unusual immune cells in urine of BCG-treated bladder cancer patients	Castellano, E and Samba, C and Esteso, G and Simpson, L and Vendrame, E and Garcia-Cuesta, EM and Lopez-Cobo, S and	10.3389/fimmu.2022.970931
Efficient Induction of Antitumor Immunity by Synthetic Toll-like Receptor Ligand-Peptide Conjugates	Zom, GG and Khan, S and Britten, CM and Sommandas, V and Camps, MGM and Loof, NM and Budden, CF and Meeuwenoo	10.1158/2326-6066.CIR-13-0223
Dual-specific Chimeric Antigen Receptor T Cells and an Indirect Vaccine Eradicate a Variety of Large Solid Tumors in an Immunocompetent, Self-antigen Setting	Slaney, CY and von Scheidt, B and Davenport, AJ and Beavis, PA and Westwood, JA and Mardiana, S and Tscharke, DC and E	10.1158/1078-0432.CCR-16-1860
Induction of eEF2-specific antitumor CTL responses in vivo by vaccination with eEF2-derived 9mer-peptides	Nakajima, H and Murakami, Y and Morii, E and Akao, T and Tatsumi, N and Odajima, S and Fukuda, M and Machitani, T and I	10.3892/or.2016.4589
Inhibition of lung tumorigenesis by a small molecule CA170 targeting the immune checkpoint protein VISTA	Pan, J and Chen, Y and Zhang, Q and Khatun, A and Palen, K and Xin, G and Wang, L and Yang, CAJ and Johnson, BD and Mye	10.1038/s42003-021-02381-x
Naturally produced type I IFNs enhance human myeloid dendritic cell maturation and IL-12p70 production and mediate elevated effector functions in innate and adaptive immune cells	Skold, AE and Mathan, TSM and van Beek, JJP and Florez-Grau, G and van den Beukel, MD and Sittig, SP and Wimmers, F and	10.1007/s00262-018-2204-2
Characterization of neoantigen-specific T cells in cancer resistant to immune checkpoint therapies	Li, SM and Simoni, Y and Zhuang, S and Gabel, A and Ma, SK and Chee, J and Islas, L and Cessna, A and Creaney, J and Brad	10.1073/pnas.2025570118
Dendritic Cells Transfected with a DNA Construct Encoding Tumour-associated Antigen Epitopes Induce a Cytotoxic Immune Response Against Autologous Tumour Cells in a Culture of Mononuclear Cells from Colorectal Cancer Patients	Kulikova, EV and Kurilin, VV and Shevchenko, JA and Obleukhova, IA and Khrapov, EA and Boyarskikh, UA and Filipenko, ML	10.1111/sji.12311
Monocytes Exposed to Immune Complexes Reduce pDC Type 1 Interferon Response to Vidutolimod	Sabree, SA and Lemke-Miltner, CD and Blackwell, SE and Yin, CB and Bossler, A and Ebeid, K and Salem, AK and Weiner, GJ	10.3390/vaccines9090982
Engineering extracellular vesicles as novel treatment options: exploiting herpesviral immunity in CLL	Gartner, K and Luckner, M and Wanner, G and Zeidler, R	10.1080/20013078.2019.1573051
Arginase-2-specific cytotoxic T cells specifically recognize functional regulatory T cells	Weis-Banke, SE and Lisle, TL and Perez-Penco, M and Schina, A and Hubbe, ML and Siersbaek, M and Holmstrom, MO and J	10.1136/jitc-2022-005326
Identification of T-cell Receptors Targeting KRAS-Mutated Human Tumors	Wang, QJ and Yu, ZY and Griffith, K and Hanada, K and Restifo, NP and Yang, JC	10.1158/2326-6066.CIR-15-0188
Antitumor effect of dendritic cells transfected with prostate-specific membrane antigen recombinant adenovirus on prostate cancer: An in vitro study	Meng, FD and Wang, S and Jiang, YH and Sui, CG	10.3892/mmr.2016.4754
Single-Cell Immune Competency Signatures Associate with Survival in Phase II GVAX and CRS-207 Randomized Studies in Patients with Metastatic Pancreatic Cancer	Nair, N and Chen, SY and Lemmens, E and Chang, S and Le, DT and Jaffee, EM and Murphy, A and Whiting, C and Muller, T a	10.1158/2326-6066.CIR-19-0650
Cell Surface-Associated Anti-MUC1-Derived Signal Peptide Antibodies: Implications for Cancer Diagnostics and Therapy	Kovjazin, R and Horn, G and Smorodinsky, NI and Shapira, MY and Carmon, L	10.1371/journal.pone.0085400

Association of WT1 IgG antibody against WT1 peptide with prolonged survival in glioblastoma multiforme patients vaccinated with WT1 peptide	Oji, Y and Hashimoto, N and Tsuboi, A and Murakami, Y and Iwai, M and Kagawa, N and Chiba, Y and Izumoto, S and Elissee	10.1002/ijc.30182
Cancer testis antigen Sperm Protein 17 as a new target for triple negative breast cancer immunotherapy	Mirandola, L and Pedretti, E and Figueroa, JA and Chiaramonte, R and Colombo, M and Chapman, C and Grizzi, F and Patrin	10.18632/oncotarget.20102
Alpha-type-1 Polarized Dendritic Cell-based Vaccination in Newly Diagnosed High-grade Glioma: A Phase II Clinical Trial	Mitsuya, K and Akiyama, Y and Iizuka, A and Miyata, H and Deguchi, S and Hayashi, N and Maeda, C and Kondou, R and Kan	10.21873/anticanres.14669
Efficacy of granulocyte-macrophage colony-stimulating factor combined with metronomic paclitaxel in the treatment of Lewis lung carcinoma transplanted in mice	Zhu, NP and Qin, RS and Zhang, Q and Fu, SZ and Liu, SS and Chen, Y and Fan, J and Han, YW	10.18632/oncotarget.23530
Anti-tumor activity of dendritic cell-cytokine induced killer cells (DC-CIKs) sensitized to HER2 against HER-positive breast cancer cells	Wen, YY and Hu, XS	10.4238/gmr.15027853
Nasopharyngeal Carriage of Pneumococcus in Children in England up to 10 Years After 13-Valent Pneumococcal Conjugate Vaccine Introduction: Persistence of Serotypes 3 and 19A and Emergence of 7C	Tiley, KS and Ratcliffe, H and Voysey, M and Jefferies, K and Sinclair, G and Carr, M and Colin-Jones, R and Smith, D and Bow	10.1093/infdis/jiac376
A Novel DNA Vaccine Platform Enhances Neo-antigen-like T Cell Responses against WT1 to Break Tolerance and Induce Anti-tumor Immunity	Walters, JN and Ferraro, B and Duperret, EK and Kraynyak, KA and Chu, JM and Saint-Fleur, A and Yan, J and Levitsky, H and	10.1016/j.ymthe.2017.01.022
Suppression of TGF-beta and IL-10 receptors on self-differentiated dendritic cells by short-hairpin RNAs enhanced activation of effector T-cells against cholangiocarcinoma cells	Thepmalee, C and Panya, A and Sujjitjoon, J and Sawasdee, N and Poungvarin, N and Junking, M and Yenchitsomanus, PT	10.1080/21645515.2019.1701913
A Comprehensive Preclinical Model Evaluating the Recombinant PRAME Antigen Combined With the AS15 Immunostimulant to Fight Against PRAME-expressing Tumors	Gerard, C and Baudson, N and Ory, T and Segal, L and Louahed, J	10.1097/CJI.0000000000000095
Stem Cell-Derived Viral Ag-Specific T Lymphocytes Suppress HBV Replication in Mice	Xiong, XF and Lei, FY and Haque, M and Song, JX	10.3791/60043
T-ALL Cells as Tool Cells for CAR T Therapy	Ren, AQ and Zhao, Y and Zhu, HC	10.3390/vaccines11040854
Deconvolution of the Response to Bacillus Calmette-Guerin Reveals NF-kappa B-Induced Cytokines As Autocrine Mediators of Innate Immunity	Bisiaux, A and Boussier, J and Duffy, D and Quintana-Murci, L and Fontes, M and Albert, ML and Milieu Interieur Consortium	10.3389/fimmu.2017.00796
Toward harmonized phenotyping of human myeloid-derived suppressor cells by flow cytometry: results from an interim study	Mandruzzato, S and Brandau, S and Britten, CM and Bronte, V and Damuzzo, V and Gouttefangeas, C and Maurer, D and Ott	10.1007/s00262-015-1782-5
Host genetics and tumor environment determine the functional impact of neutrophils in mouse tumor models	van Elsas, M and Kleinovink, JW and Moerland, M and Feiss, G and Beyrend, G and Arens, R and Mei, HL and Nibbering, PH a	10.1136/jitc-2020-000877
Monitoring of dynamic changes in Keyhole Limpet Hemocyanin (KLH)-specific B cells in KLH-vaccinated cancer patients	Wimmers, F and de Haas, N and Scholzen, A and Schreibelt, G and Simonetti, E and Eleveld, MJ and Brouwers, HMLM and B	10.1038/srep43486
Myeloid-derived suppressor cells are increased and correlated with type 2 immune responses, malnutrition, inflammation, and poor prognosis in patients with breast cancer	Gonda, K and Shibata, M and Ohtake, T and Matsumoto, Y and Tachibana, K and Abe, N and Ohto, H and Sakurai, K and Take	10.3892/ol.2017.6305
Targeting LAG-3 and PD-1 to Enhance T Cell Activation by Antigen-Presenting Cells	Lichtenegger, FS and Rothe, M and Schnorfeil, FM and Deiser, K and Krupka, C and Augsberger, C and Schluter, M and Neitz	10.3389/fimmu.2018.00385
Humoral response to a viral glycan correlates with survival on PROSTVAC-VF	Campbell, CT and Gulley, JL and Oyelaran, O and Hodge, JW and Schlom, J and Gildersleeve, JC	10.1073/pnas.1314722111
Human CLEC9A antibodies deliver NY-ESO-1 antigen to CD141(+) dendritic cells to activate naive and memory NY-ESO1-specific CD8(+) T cells	Masterman, KA and Haigh, OL and Tullett, KM and Leal-Rojas, IM and Walpole, C and Pearson, FE and Cebon, J and Schmid	10.1136/jitc-2020-000691
Simple paired heavy- and light-chain antibody repertoire sequencing using endoplasmic reticulum microsomes	Devulapally, PR and Burger, J and Mielke, T and Konthur, Z and Lehrach, H and Yaspo, ML and Glokler, J and Warnatz, HJ	10.1186/s13073-018-0542-5
Human leukemia antigen-A*0201-restricted epitopes of human endogenous retrovirus W family envelope (HERV-W env) induce strong cytotoxic T lymphocyte responses	Tu, XN and Li, S and Zhao, LJ and Xiao, R and Wang, XL and Zhu, F	10.1007/s12250-017-3984-9
Surrogate in vitro activation of innate immunity synergizes with interleukin-7 to unleash rapid antigen-driven outgrowth of CD4+and CD8+human peripheral blood T-cells naturally recognizing MUC1, HER2/neu and other tumor-associated antigens	Pathangey, LB and McCurry, DB and Gendler, SJ and Dominguez, AL and Gorman, JE and Pathangey, G and Mihalik, LA and D	10.18632/oncotarget.13911
Anti-CD40-induced inflammatory E-cadherin plus dendritic cells enhance T cell responses and antitumour immunity in murine Lewis lung carcinoma	Zhang, Y and Hu, XY and Hu, Y and Teng, K and Zhang, K and Zheng, YM and Hong, XH and Yu, KW and Wang, Y and Liu, L	10.1186/s13046-015-0126-9
Personal neoantigen vaccines induce persistent memory T cell responses and epitope spreading in patients with melanoma	Hu, ZT and Leet, DE and Allesoe, RL and Oliveira, G and Li, SQ and Luoma, AM and Liu, JY and Forman, J and Huang, T and Io	10.1038/s41591-020-01206-4
Norovirus-Specific CD8(+) T Cell Responses in Human Blood and Tissues	Pattekar, A and Mayer, LS and Lau, CW and Liu, CY and Palko, O and Bewtra, M and Consortium, H and Lindesmith, LC and	10.1016/j.jcmgh.2020.12.012
Gonadotropin-mediated chemoresistance: Delineation of molecular pathways and targets	Sahoo, S and Singh, P and Kalha, B and Singh, O and Pal, R	10.1186/s12885-015-1938-x
Targeting myeloid derived suppressor cells with all-trans retinoic acid is highly time-dependent in therapeutic tumor vaccination	Heine, A and Flores, C and Gevensleben, H and Diehl, L and Heikenwalder, M and Ringelhan, M and Janssen, KP and Nitsch	10.1080/2162402X.2017.1338995
Monocytes complexed to platelets differentiate into functionally deficient dendritic cells	Singh, MV and Suwunnakorn, S and Simpson, SR and Weber, EA and Singh, VB and Kalinski, P and Maggirwar, SB	10.1002/JLB.3A0620-460RR
Emerging trends and research foci of oncolytic virotherapy for central nervous system tumors: A bibliometric study	Cheng, KM and Zhang, H and Guo, Q and Zhai, PF and Zhou, Y and Yang, WG and Wang, YL and Lu, YQ and Shen, ZF and Wu,	10.3389/fimmu.2022.975695

Intratumorally delivered formulation, INT230-6, containing potent anticancer agents induces protective T cell immunity and memory	Bloom, AC and Bender, LH and Tiwary, S and Pasquet, L and Clark, K and Jiang, TB and Xia, Z and Morales-Kastresana, A and	10.1080/2162402X.2019.1625687
Overcoming target epitope masking resistance that can occur on low-antigen-expresser AML blasts after IL-1RAP chimeric antigen receptor T cell therapy using the inducible caspase 9 suicide gene safety switch	Warda, W and Da Rocha, MN and Trad, R and Haderbache, R and Salma, Y and Bouquet, L and Roussel, X and Nicod, C and	10.1038/s41417-020-00284-3
An allergen-fused dendritic cell-binding peptide enhances in vitro proliferation of equine T-cells and cytokine production	Ziegler, A and Olzhausen, J and Hamza, E and Stojiljkovic, A and Stoffel, MH and Garbani, M and Rhyner, C and Marti, E	10.1016/j.vetimm.2021.110351
Presence of T cells directed against CD20-derived peptides in healthy individuals and lymphoma patients	Milcent, B and Josseaume, N and Riller, Q and Giglioli, I and Rabia, E and Deligne, C and Latouche, JB and Hamieh, M and C	10.1007/s00262-019-02389-7
Immune Correlates of GM-CSF and Melanoma Peptide Vaccination in a Randomized Trial for the Adjuvant Therapy of Resected High-Risk Melanoma (E4697)	Butterfield, LH and Zhao, FM and Lee, S and Tarhini, AA and Margolin, KA and White, RL and Atkins, MB and Cohen, GI and W	10.1158/1078-0432.CCR-16-3016
Multifaceted glycoadjuvant@AuNPs inhibits tumor metastasis through promoting T cell activation and remodeling tumor microenvironment	Xu, XJ and Gan, MF and Ge, YZ and Yi, C and Feng, TN and Liu, MJ and Wu, CH and Chen, X and Zhang, WD and Zhao, LX and	10.1186/s12951-021-01129-3
Detecting T cell receptors involved in immune responses from single repertoire snapshots	Pogorelyy, MV and Minervina, AA and Shugay, M and Chudakov, DM and Lebedev, YB and Mora, T and Walczak, AM	10.1371/journal.pbio.3000314
Identification of patient-specific and tumor-shared T cell receptor sequences in renal cell carcinoma patients	Massa, C and Robins, H and Desmarais, C and Riemann, D and Fahldieck, C and Fornara, P and Seliger, B	10.18632/oncotarget.15064
A phase II trial of recombinant MAGE-A3 protein with immunostimulant AS15 in combination with high-dose Interleukin-2 (HDIL2) induction therapy in metastatic melanoma	McQuade, JL and Homsi, J and Torres-Cabala, CA and Bassett, R and Popuri, RM and James, ML and Vence, LM and Hwu, WJ	10.1186/s12885-018-5193-9
Predictors of Response to Autologous Dendritic Cell Therapy in Glioblastoma Multiforme	Jan, CI and Tsai, WC and Harn, HJ and Shyu, WC and Liu, MC and Lu, HM and Chiu, SC and Cho, DY	10.3389/fimmu.2018.00727
Harmonisation of short-term in vitro culture for the expansion of antigen-specific CD8(+) T cells with detection by ELISPOT and HLA-multimer staining	Chudley, L and McCann, KJ and Coleman, A and Cazaly, AM and Bidmon, N and Britten, CM and van der Burg, SH and Goutte	10.1007/s00262-014-1593-0
CMVpp65 Vaccine Enhances the Antitumor Efficacy of Adoptively Transferred CD19-Redirected CMV-Specific T Cells	Wang, XL and Wong, CW and Urak, R and Mardiros, A and Budde, LE and Chang, WC and Thomas, SH and Brown, CE and La	10.1158/1078-0432.CCR-14-2920
Cytokine-induced killer cell therapy for modulating regulatory T cells in patients with non-small cell lung cancer	Yu, BD and Wang, JL and He, C and Wang, W and Tang, JL and Zheng, RH and Zhou, CZ and Zhang, HH and Fu, ZP and Li, QS	10.3892/etm.2017.4562
Emerging immuno-oncology targets in Myelodysplastic Syndromes (MDS)	Mann, M and Brunner, AM	10.1016/j.currproblcancer.2021.100824
Accurate detection of tumor-specific gene fusions reveals strongly immunogenic personal neo-antigens	Weber, D and Ibn-Salem, J and Sorn, P and Suchan, M and Holtstrater, C and Lahrmann, U and Vogler, I and Schmoldt, K and	10.1038/s41587-022-01247-9
Survivin-specific CD4+ T cells are decreased in patients with survivin-positive myeloma	Locke, FL and Menges, M and Veerapathran, A and Coppola, D and Gabrilovich, D and Anasetti, C	10.1186/s40425-015-0065-1
The Detection of Immunity against WT1 and SMAD4(P130L) of EpCAM(+) Cancer Cells in Malignant Pleural Effusion	Koya, T and Niida, Y and Togi, M and Yoshida, K and Sakamoto, T and Ura, H and Togi, S and Kato, T and Yamada, S and Sugiy	10.3390/ijms232012177
Development of a Minor Histocompatibility Antigen Vaccine Regimen in the Canine Model of Hematopoietic Cell Transplantation	Rosinski, SL and Stone, B and Graves, SS and Fuller, DH and De Rosa, SC and Spies, GA and Mize, GJ and Fuller, JT and Storb	10.1097/TP.0000000000000744
HER2-antigen-specific humoral immune response in breast cancer lymphocytes transplanted in hu-PBL hIL-4 NOG mice	Ohno, Y and Ohshima, S and Miyamoto, A and Kametani, F and Ito, R and Tsuda, B and Kasama, Y and Nakada, S and Kashiw	10.1038/s41598-021-92311-y
Natural and cryptic peptides dominate the immunopeptidome of atypical teratoid rhabdoid tumors	Marcu, A and Schlosser, A and Keupp, A and Trautwein, N and Johann, P and Wolfl, M and Lager, J and Monoranu, CM and W	10.1136/jitc-2021-003404
Neddylation tunes peripheral blood mononuclear cells immune response in COVID-19 patients	Serrano-Macia, M and Lachiondo-Ortega, S and Iruzubieta, P and Goikoetxea-Usandizaga, N and Bosch, A and Egia-Mendiku	10.1038/s41420-022-01115-0
Anti-tumor immunity induced by ectopic expression of viral antigens is transient and limited by immune escape	Dharmaraj, N and Piotrowski, SL and Huang, C and Newton, JM and Golfman, LS and Hanoteau, A and Koshy, ST and Li, AW	10.1080/2162402X.2019.1568809
A nomogram for predicting mortality in patients with COVID-19 and solid tumors: a multicenter retrospective cohort study	Liu, C and Li, L and Song, KH and Zhan, ZY and Yao, Y and Gong, HY and Chen, Y and Wang, Q and Dong, XR and Xie, ZB and	10.1136/jitc-2020-001314
In vivo imaging of the immune response upon systemic RNA cancer vaccination by FDG-PET	Pektor, S and Hilscher, L and Walzer, KC and Miederer, I and Bausbacher, N and Loquai, C and Schreckenberger, M and Sah	10.1186/s13550-018-0435-z
Single-cell TCRseq: paired recovery of entire T-cell alpha and beta chain transcripts in T-cell receptors from single-cell RNAseq	Redmond, D and Poran, A and Elemento, O	10.1186/s13073-016-0335-7
Phase I study with ONCOS-102 for the treatment of solid tumors - an evaluation of clinical response and exploratory analyses of immune markers	Ranki, T and Pesonen, S and Hemminki, A and Partanen, K and Kairemo, K and Alanko, T and Lundin, J and Linder, N and Tur	10.1186/s40425-016-0121-5
Molecular Characterization of Antigen-Peptide Pulsed Dendritic Cells: Immature Dendritic Cells Develop a Distinct Molecular Profile when Pulsed with Antigen Peptide	Yang, AX and Chong, NJ and Jiang, YF and Catalano, J and Puri, RK and Khleif, SN	10.1371/journal.pone.0086306
Intratumorally injected pro-inflammatory allogeneic dendritic cells as immune enhancers: a first-in-human study in unfavourable risk patients with metastatic renal cell carcinoma	Laurell, A and Lonnemark, M and Brekkan, E and Magnusson, A and Tolf, A and Wallgren, AC and Andersson, B and Adamson	10.1186/s40425-017-0255-0
COVID-19 convalescent plasma as long-term therapy in immunodeficient patients?	Rnjak, D and Ravlic, S and Sola, AM and Halassy, B and Semnicki, J and Superba, M and Hecimovic, A and Kurolt, IC and Kur	10.1016/j.tracli.2021.04.004
An Exploratory Analysis of Changes in Circulating Plasma Protein Profiles Following Image-Guided Ablation of Renal Tumours Provides Evidence for Effects on Multiple Biological Processes	Wah, TM and Zhong, JM and Wilson, M and Vasudev, NS and Banks, RE	10.3390/cancers13236037

Hypofractionated Irradiation Suppressed the Off-Target Mouse Hepatocarcinoma Growth by Inhibiting Myeloid-Derived Suppressor Cell-Mediated Immune Suppression	Chen, JY and Wang, Z and Ding, YX and Huang, F and Huang, WK and Lan, RL and Chen, RQ and Wu, B and Fu, LX and Yang,	10.3389/fonc.2020.00004
BCMA peptide-engineered nanoparticles enhance induction and function of antigen-specific CD8(+) cytotoxic T lymphocytes against multiple myeloma: clinical applications	Bae, J and Parayath, N and Ma, WX and Amiji, M and Munshi, N and Anderson, KC	10.1038/s41375-019-0540-7
High-Dimensional Phenotypic Mapping of Human Dendritic Cells Reveals Interindividual Variation and Tissue Specialization	Alcantara-Hernandez, M and Leylek, R and Wagar, LE and Engleman, EG and Keler, T and Marinkovich, MP and Davis, MM an	10.1016/j.immuni.2017.11.001
Mannosylated T/Tn with Freund's adjuvant induces cellular immunity	Son, HY and Apostolopoulos, V and Kim, CW	10.1177/0394632017742504
First-in-human study of WT1 recombinant protein vaccination in elderly patients with AML in remission: a single-center experience	Kreutmair, S and Pfeifer, D and Waterhouse, M and Takacs, F and Graessel, L and Dohner, K and Duyster, J and Illert, AL and	10.1007/s00262-022-03202-8
Catalytic Immunoglobulin Gene Delivery in a Mouse Model of Alzheimer's Disease: Prophylactic and Therapeutic Applications	Kou, JH and Yang, JL and Lim, JE and Pattanayak, A and Song, M and Planque, S and Paul, S and Fukuchi, KI	10.1007/s12035-014-8691-z
Dasatinib promotes the expansion of a therapeutically superior T-cell repertoire in response to dendritic cell vaccination against melanoma	Lowe, DB and Bose, A and Taylor, JL and Tawbi, H and Lin, Y and Kirkwood, JM and Storkus, WJ	10.4161/onci.27589
Endogenous Neoantigen-Specific CD8 T Cells Identified in Two Glioblastoma Models Using a Cancer Immunogenomics Approach	Johanns, TM and Ward, JP and Miller, CA and Wilson, C and Kobayashi, DK and Bender, D and Fu, YJ and Alexandrov, A and N	10.1158/2326-6066.CIR-16-0156
Exosomes derived from rAAV/AFP-transfected dendritic cells elicit specific T cell-mediated immune responses against hepatocellular carcinoma	Li, JY and Huang, SL and Zhou, ZF and Lin, WS and Chen, SP and Chen, MS and Ye, YB	10.2147/CMAR.S178326
Oncolytic Vaccinia Virus Gene Modification and Cytokine Expression Effects on Tumor Infection, Immune Response, and Killing	Inoue, T and Byrne, T and Inoue, M and Tait, ME and Wall, P and Wang, A and Dermyer, MR and Laklai, H and Binder, JJ and L	10.1158/1535-7163.MCT-20-0863
Complete and long-lasting clinical responses in immune checkpoint inhibitor-resistant, metastasized melanoma treated with adoptive T cell transfer combined with DC vaccination	Lovgren, T and Wolodarski, M and Wickstrom, S and Edback, U and Wallin, M and Martell, E and Markland, K and Blomberg,	10.1080/2162402X.2020.1792058
Combined TLR-3/TLR-8 Signaling in the Presence of alpha-Type-1 Cytokines Represents a Novel and Potent Dendritic Cell Type-1, Anti-Cancer Maturation Protocol	Fevzer, T and Pozenel, P and Zajc, K and Tesic, N and Svajger, U	10.3390/cells11050835
TruNeo: an integrated pipeline improves personalized true tumor neoantigen identification	Tang, YX and Wang, Y and Wang, JQ and Li, M and Peng, LM and Wei, GC and Zhang, YX and Li, J and Gao, ZB	10.1186/s12859-020-03869-9
Characterization of Postinfusion Phenotypic Differences in Fresh Versus Cryopreserved TCR Engineered Adoptive Cell Therapy Products	Nowicki, TS and Escuin-Ordinas, H and Avramis, E and Chmielowski, B and Chodon, T and Berent-Maoz, B and Wang, XY and	10.1097/CJI.0000000000000216
Peptide Processing Is Critical for T-Cell Memory Inflation and May Be Optimized to Improve Immune Protection by CMV-Based Vaccine Vectors	Dekhtiarenko, I and Ratts, RB and Blatnik, R and Lee, LN and Fischer, S and Borkner, L and Oduro, JD and Marandu, TF and H	10.1371/journal.ppat.1006072
Anti-SARS-CoV-2 antibody-containing plasma improves outcome in patients with hematologic or solid cancer and severe COVID-19: a randomized clinical trial	Denkinger, CM and Janssen, M and Schakel, U and Gall, J and Leo, A and Stelmach, P and Weber, SF and Krisam, J and Baur	10.1038/s43018-022-00503-w
Predictors of Epstein-Barr virus serostatus in young people in England	Winter, JR and Taylor, GS and Thomas, OG and Jackson, C and Lewis, JEA and Stagg, HR	10.1186/s12879-019-4578-y
Complementation of Antigen-presenting Cells to Generate T Lymphocytes With Broad Target Specificity	Ngo, MC and Ando, J and Leen, AM and Ennamuri, S and Lapteva, N and Vera, JF and Min-Venditti, A and Mims, MP and Hesl	10.1097/CJI.0000000000000014
Clonal replacement of tumor-specific T cells following PD-1 blockade	Yost, KE and Satpathy, AT and Wells, DK and Qi, YY and Wang, CL and Kageyama, R and McNamara, KL and Granja, JM and S	10.1038/s41591-019-0522-3
A Non-interventional Clinical Trial Assessing Immune Responses After Radiofrequency Ablation of Liver Metastases From Colorectal Cancer	Loffler, MW and Nussbaum, B and Jager, G and Jurmeister, PS and Budczies, J and Pereira, PL and Clasen, S and Kowalewsk	10.3389/fimmu.2019.02526
Oxidized-Desialylated Low-Density Lipoprotein Inhibits the Antitumor Functions of Lymphokine Activated Killer Cells	De Leon, JSAD and Glenn, HL and Knappenberger, M and Borges, CR	10.7150/jca.55526
Intradermal vaccination of HPV-16 E6 synthetic peptides conjugated to an optimized Toll-like receptor 2 ligand shows safety and potent T cell immunogenicity in patients with HPV-16 positive (pre-)malignant lesions	Speetjens, FM and Welters, MJP and Slingerland, M and van Poelgeest, MIE and van Steenwijk, PJD and Roozen, I and Boeke	10.1136/jitc-2022-005016
Cytofast: A workflow for visual and quantitative analysis of flow andmass cytometry data to discover immune signatures and correlations	Beyrend, G and Stam, K and Hollt, T and Ossendorp, F and Arens, R	10.1016/j.csbj.2018.10.004
Spontaneous tumor regression following COVID-19 vaccination	de Sousa, LG and McGrail, DJ and Li, KY and Marques-Piubelli, ML and Gonzalez, C and Dai, H and Ferri-Borgogno, S and Go	10.1136/jitc-2021-004371
Identification of TCR repertoires in functionally competent cytotoxic T cells cross-reactive to SARS-CoV-2	Shimizu, K and Iyoda, T and Sanpei, A and Nakazato, H and Okada, M and Ueda, S and Kato-Murayama, M and Murayama, K	10.1038/s42003-021-02885-6
Peptide vaccination activating Galectin-3-specific T cells offers a novel means to target Galectin-3-expressing cells in the tumor microenvironment	Bendtsen, SK and Perez-Penco, M and Hubbe, ML and Martinenaite, E and Holmstrom, MO and Weis-Banke, SE and Jorgens	10.1080/2162402X.2022.2026020
Therapeutic efficacy of dendritic cell injection in advanced hepatocellular carcinoma: the role of natural killer and T lymphocytes	Zaghloul, A and Rashad, K and Gabr, H and Nabil, A and Abdel-Moneim, A	10.5114/ceh.2022.116999
Type I Interferons and Cancer: An Evolving Story Demanding Novel Clinical Applications	Arico, E and Castiello, L and Capone, I and Gabriele, L and Belardelli, F	10.3390/cancers11121943
T-Cell Therapy: Options for Infectious Diseases	Parida, SK and Poiret, T and Zhenjiang, L and Meng, QD and Heyckendorf, J and Lange, C and Ambati, AS and Rao, MV and V	10.1093/cid/civ615
CAR T cells targeting the ganglioside NGcGM3 control ovarian tumors in the absence of toxicity against healthy tissues	Cribioli, E and Attianese, GMPG and Coukos, G and Irving, M	10.3389/fimmu.2022.951143
DNA-Barcoded pMHC Tetramers for Detection of Single Antigen-Specific T Cells by Digital PCR	Dahotre, SN and Chang, YM and Romanov, AM and Kwong, GA	10.1021/acs.analchem.8b04153

A randomised controlled trial of long NY-ESO-1 peptide-pulsed autologous dendritic cells with or without alpha-galactosylceramide in high-risk melanoma	Dasyam, N and Sharples, KJ and Barrow, C and Huang, Y and Bauer, E and Mester, B and Wood, CE and Authier-Hall, A and D	10.1007/s00262-023-03400-y
Immune Checkpoint Blockade Enhances Shared Neoantigen-Induced T-cell Immunity Directed against Mutated Calreticulin in Myeloproliferative Neoplasmas	Bozkus, CC and Roudko, V and Finnigan, JP and Mascarenhas, J and Hoffman, R and Iancu-Rubin, C and Bhardwaj, N	10.1158/2159-8290.CD-18-1356
An Adaptable Antibody-Based Platform for Flexible Synthetic Peptide Delivery Built on Agonistic CD40 Antibodies	Eltahir, M and Lauren, I and Lord, M and Chourlia, A and Dahllund, L and Olsson, A and Saleh, A and Ytterberg, AJ and Lindqv	10.1002/adtp.202200008
A universal MHCII technology platform to characterize antigen-specific CD4+ T cells	Vyasamneni, R and Kohler, V and Karki, B and Mahimkar, G and Esaulova, E and McGee, J and Kallin, D and Sheen, JH and H	10.1016/j.crmeth.2022.100388
A Leucine Zipper Dimerization Strategy to Generate Soluble T Cell Receptors Using the Escherichia coli Expression System	Zhang, AEL and Piechocka-Trocha, A and Li, XL and Walker, BD	10.3390/cells11030312
Linking T cell epitopes to a common linear B cell epitope: A targeting and adjuvant strategy to improve T cell responses	Mangsbo, SM and Fletcher, EAK and van Maren, WWC and Redeker, A and Cordfunke, RA and Dillmann, I and Dinkelaar, J ar	10.1016/j.molimm.2017.11.004
Identification of Tumor Antigens Among the HLA Peptidomes of Glioblastoma Tumors and Plasma	Shraibman, B and Barnea, E and Kadosh, DM and Haimovich, Y and Slobodin, G and Rosner, I and Lopez-Larrea, C and Hilf,	10.1074/mcp.RA119.001524
Effect of cytokine-induced killer cells combined with dendritic cells on the survival rate and expression of 14-3-3 zeta and p-Bad proteins in Lewis lung cancer cell lines	Hou, Y and Zang, DY and Li, XM and Li, FZ	10.3892/ol.2018.8834
Alloantigen-activated (AAA) CD4(+) T cells reinvigorate host endogenous T cell immunity to eliminate pre-established tumors in mice	Mochizuki, K and Kobayashi, S and Takahashi, N and Sugimoto, K and Sano, H and Ohara, Y and Mineishi, S and Zhang, Y an	10.1186/s13046-021-02102-6
HBV-Derived Synthetic Long Peptide Can Boost CD4(+) and CD8(+) T-Cell Responses in Chronic HBV Patients Ex Vivo	Dou, YY and van Montfoort, N and van den Bosch, A and de Man, RA and Zom, GG and Krebber, WJ and Melief, CJM and Busc	10.1093/infdis/jix614
Targeting CLEC9A delivers antigen to human CD141(+) DC for CD4(+) and CD8(+) T cell recognition	Tullett, KM and Rojas, IML and Minoda, Y and Tan, PS and Zhang, JG and Smith, C and Khanna, R and Shortman, K and Cami	10.1172/jci.insight.87102
Effective chemoimmunotherapy by co-delivery of doxorubicin and immune adjuvants in biodegradable nanoparticles	Da Silva, CG and Camps, MGM and Li, TMWY and Zerrillo, L and Lowik, CW and Ossendorp, F and Cruz, LJ	10.7150/thno.34429
p38 MAPK-inhibited dendritic cells induce superior antitumour immune responses and overcome regulatory T-cell-mediated immunosuppression	Lu, Y and Zhang, MJ and Wang, SQ and Hong, BX and Wang, ZQ and Li, HY and Zheng, YH and Yang, J and Davis, RE and Qian	10.1038/ncomms5229
Results of the ADAPT Phase 3 Study of Rocapuldencel-T in Combination with Sunitinib as First-Line Therapy in Patients with Metastatic Renal Cell Carcinoma	Figlin, RA and Tannir, NM and Uzzo, RG and Tykodi, SS and Chen, DYT and Master, V and Kapoor, A and Vaena, D and Lowrar	10.1158/1078-0432.CCR-19-2427
Checkpoint inhibition in combination with an immunoboost of external beam radiotherapy in solid tumors (CHEERS): study protocol for a phase 2, open-label, randomized controlled trial	Spaas, M and Sundahl, N and Hulstaert, E and Kruse, V and Rottey, S and De Maeseneer, D and Surmont, V and Meireson, A	10.1186/s12885-021-08088-w
Notch-Mediated Generation of Monocyte-Derived Langerhans Cells: Phenotype and Function	Bellmann, L and Zelle-Rieser, C and Milne, P and Resteu, A and Tripp, CH and Hermann-Kleiter, N and Zaderer, V and Wilflin	10.1016/j.jid.2020.05.098
Diversity and divergence of the glioma-infiltrating T-cell receptor repertoire	Sims, JS and Grinshpun, B and Feng, YP and Ung, TH and Neira, JA and Samanamud, JL and Canoll, P and Shen, YF and Sims	10.1073/pnas.1601012113
Systemic delivery of TNF-armed myxoma virus plus immune checkpoint inhibitor eliminates lung metastatic mouse osteosarcoma	Christie, JD and Appel, N and Canter, H and Achi, JG and Elliott, NM and de Matos, AL and Franco, L and Kilbourne, J and Lov	10.1016/j.omto.2021.07.014
Immune landscape and in vivo immunogenicity of NY-ESO-1 tumor antigen in advanced neuroblastoma patients	Camisaschi, C and Renne, SL and Beretta, V and Rini, F and Spagnuolo, RD and Tuccitto, A and Podda, MG and Parmiani, G	10.1186/s12885-018-4910-8
Feasibility and Immune Response of WT1 Peptide Vaccination in Combination with OK-432 for Paediatric Solid Tumors	Hirabayashi, K and Yanagisawa, R and Saito, S and Higuchi, Y and Koya, T and Sano, K and Koido, S and Okamoto, M and Su	10.21873/anticanres.12465
Biomarkers and risk factors for the early prediction of immune-related adverse events: a review	Zhang, Y and Zhang, XL and Li, WL and Du, YY and Hu, WQ and Zhao, J	10.1080/21645515.2021.2018894
Prostaglandin E-2 in a TLR3-and 7/8-agonist-based DC maturation cocktail generates mature, cytokine-producing, migratory DCs but impairs antigen cross-presentation to CD8(+) T cells	Gierlich, P and Lex, V and Technau, A and Keupp, A and Morper, L and Glunz, A and Sennholz, H and Rachor, J and Sauer, S a	10.1007/s00262-019-02470-1
Autologous Transplantation Using Donor Leukocytes Loaded Ex Vivo with Oncolytic Myxoma Virus Can Eliminate Residual Multiple Myeloma	Villa, N and Rahman, MM and Mamola, J and D'Isabella, J and Goras, E and Kilbourne, J and Lowe, K and Daggett-Vondras, J	10.1016/j.omto.2020.06.011
The IDO1 selective inhibitor epacadostat enhances dendritic cell immunogenicity and lytic ability of tumor antigen-specific T cells	Jochems, C and Fantini, M and Fernando, RI and Kwilas, AR and Donahue, RN and Lepone, LM and Grenga, I and Kim, YS and	10.18632/oncotarget.9326
Targeted Activation of Toll-Like Receptors: Conjugation of a Toll-Like Receptor 7 Agonist to a Monoclonal Antibody Maintains Antigen Binding and Specificity	Gadd, AJR and Greco, F and Cobb, AJA and Edwards, AD	10.1021/acs.bioconjchem.5b00302
A large peptidome dataset improves HLA class I epitope prediction across most of the human population	Sarkizova, S and Klaeger, S and Le, PM and Li, LW and Oliveira, G and Keshishian, H and Hartigan, CR and Zhang, WD and Br	10.1038/s41587-019-0322-9
Cellular Immune Responses for Squamous Cell Carcinoma Antigen Recognized by T Cells 3 in Patients with Hepatocellular Carcinoma	Kaji, K and Mizukoshi, E and Yamashita, T and Arai, K and Sunagozaka, H and Fushimi, K and Nakagawa, H and Yamada, K ar	10.1371/journal.pone.0170291
Human V gamma 9/V delta 2 T cells: Innate adaptors of the immune system	Tyler, CJ and Doherty, DG and Moser, B and Eberl, M	10.1016/j.cellimm.2015.01.008
Clinical Dosing Regimen of Selinexor Maintains Normal Immune Homeostasis and T-cell Effector Function in Mice: Implications for Combination with Immunotherapy	Tyler, PM and Servos, MM and de Vries, RC and Klebanov, B and Kashyap, T and Sacham, S and Landesman, Y and Dougan, I	10.1158/1535-7163.MCT-16-0496
Incidence determinants and serological correlates of reactive symptoms following SARS-CoV-2 vaccination	Holt, H and Jolliffe, DA and Talaei, M and Faustini, S and Vivaldi, G and Greenig, M and Richter, AG and Lyons, RA and Griffith	10.1038/s41541-023-00614-0
Neutrophil-Based Drug Delivery Systems	Chu, DF and Dong, XY and Shi, XT and Zhang, CY and Wang, ZJ	10.1002/adma.201706245

Vaccination against Oncoproteins of HPV16 for Noninvasive Vulvar/Vaginal Lesions: Lesion Clearance Is Related to the Strength of the T-Cell Response	van Poelgeest, MIE and Welters, MJP and Vermeij, R and Stynenbosch, LFM and Loof, NM and Berends-van der Meer, DMA ar	10.1158/1078-0432.CCR-15-2594
Achieving dendritic cell subset-specific targeting in vivo by site-directed conjugation of targeting antibodies to nanocarriers	Simon, J and Fichter, M and Kuhn, G and Brueckner, M and Kappel, C and Schunke, J and Klaus, T and Grabbe, S and Landfe	10.1016/j.nantod.2022.101375
Card9 controls Dectin-1-induced T-cell cytotoxicity and tumor growth in mice	Haas, T and Heidegger, S and Wintges, A and Bscheider, M and Bek, S and Fischer, JC and Eisenkolb, G and Schmickl, M and	10.1002/eji.201646775
Randomized phase II trial of lymphodepletion plus adoptive cell transfer of tumor-infiltrating lymphocytes, with or without dendritic cell vaccination, in patients with metastatic melanoma	Saberian, C and Amaria, RN and Najjar, AM and Radvanyi, LG and Haymaker, CL and Forget, MA and Bassett, RL and Faria, S	10.1136/jitc-2021-002449
Cell and fluid sampling microneedle patches for monitoring skin-resident immunity	Mandal, A and Boopathy, AV and Lam, LKW and Moynihan, KD and Welch, ME and Bennett, NR and Turvey, ME and Thai, N a	10.1126/scitranslmed.aar2227
Dendritic Cell Vaccination Enhances Immune Responses and Induces Regression of HER2(pos) DCIS Independent of Route: Results of Randomized Selection Design Trial	Lowenfeld, L and Mick, R and Datta, J and Xu, SW and Fitzpatrick, E and Fisher, CS and Fox, KR and DeMichele, A and Zhang,	10.1158/1078-0432.CCR-16-1924
Prediction and identification of human leukocyte antigen-A2-restricted cytotoxic T lymphocyte epitope peptides from the human papillomavirus 58 E7 protein	Wang, H and Chen, LL and Ma, WH and Zeng, Y and Qin, L and Chen, MJ and Li, L	10.3892/ol.2018.8875
Adoptive Transfer of MART-1 T-Cell Receptor Transgenic Lymphocytes and Dendritic Cell Vaccination in Patients with Metastatic Melanoma	Chodon, T and Comin-Anduix, B and Chmielowski, B and Koya, RC and Wu, ZQ and Auerbach, M and Ng, C and Avramis, E a	10.1158/1078-0432.CCR-13-3017
Assessing the impact of AGS-004, a dendritic cell-based immunotherapy, and vorinostat on persistent HIV-1 Infection	Gay, CL and Kuruc, JD and Falcinelli, SD and Warren, JA and Reifeis, SA and Kirchherr, JL and James, KS and Dewey, MG and	10.1038/s41598-020-61878-3
Twelve-year survival and immune correlates in dendritic cell-vaccinated melanoma patients	Gross, S and Erdmann, M and Haendle, I and Voland, S and Berger, T and Schultz, E and Strasser, E and Dankerl, P and Janka	10.1172/jci.insight.91438
A cytotoxic-skewed immune set point predicts low neutralizing antibody levels after Zika virus infection	McCarthy, EE and Odorizzi, PM and Lutz, E and Smullin, CP and Tenvooren, I and Stone, M and Simmons, G and Hunt, PW ar	10.1016/j.celrep.2022.110815
Human papillomavirus type 16 viral load is decreased following a therapeutic vaccination	Coleman, HN and Greenfield, WW and Stratton, SL and Vaughn, R and Kieber, A and Moerman-Herzog, AM and Spencer, HJ	10.1007/s00262-016-1821-x
Antigen-Presenting Human gamma delta T Cells Promote Intestinal CD4(+) T Cell Expression of IL-22 and Mucosal Release of Calprotectin	Tyler, CJ and McCarthy, NE and Lindsay, JO and Stagg, AJ and Moser, B and Eberl, M	10.4049/jimmunol.1700003
T-Cell Therapeutics Targeting Human Parainfluenza Virus 3 Are Broadly Epitope Specific and Are Cross Reactive With Human Parainfluenza Virus 1	Harris, KM and Horn, SE and Grant, ML and Lang, HL and Sani, G and Jensen-Wachspress, MA and Kankate, VV and Datar, A	10.3389/fimmu.2020.575977
GM-CSF, Flt3-L and IL-4 affect viability and function of conventional dendritic cell types 1 and 2	Lellahi, SM and Azeem, W and Hua, YP and Gabriel, B and Rye, KP and Reikvam, H and Kalland, KH	10.3389/fimmu.2022.1058963
beta-Catenin mediates tumor-induced immunosuppression by inhibiting cross-priming of CD8(+) T cells	Liang, XJ and Fu, CM and Cui, WG and Ober-Blobaum, JL and Zahner, SP and Shrikant, PA and Clausen, BE and Flavell, RA ar	10.1189/jlb.0613330
Development and validation of a Luminex assay for detection of a predictive biomarker for PROSTVAC-VF therapy	Lucas, JL and Tacheny, EA and Ferris, A and Galusha, M and Srivastava, AK and Ganguly, A and Williams, PM and Sachs, MC	10.1371/journal.pone.0182739
Bet v 1-displaying elastin like polypeptide nanoparticles induce a strong humoral and weak CD4+T-cell response against Bet v 1 in a murine immunogenicity model	van Strien, J and Warmenhoven, H and Logiantara, A and Makurat, M and Aglas, L and Bethanis, A and Leboux, R and van Rijt	10.3389/fimmu.2022.1006776
Enhanced antitumor effect of cytotoxic T lymphocytes induced by dendritic cells pulsed with colorectal cancer cell lysate expressing alpha-Gal epitopes	Xing, XW and Zou, ZY and He, CZ and Hu, ZL and Liang, K and Liang, WT and Wang, YF and Du, XH	10.3892/ol.2019.10376
Pilot study of intratumoral injection of recombinant heat shock protein 70 in the treatment of malignant brain tumors in children	Shevtsov, MA and Kim, AV and Samochernych, KA and Romanova, IV and Margulis, BA and Guzhova, IV and Yakovenko, IV ar	10.2147/OTT.S62764
Vaccination Ameliorates Cellular Inflammatory Responses in SARS-CoV-2 Breakthrough Infections	Huapaya, JA and Higgins, J and Kanth, S and Demirkale, CY and Gairhe, S and Aboye, EA and Regenold, D and Sahagun, SJ a	10.1093/infdis/jiad045
Predicting HLA class II antigen presentation through integrated deep learning	Chen, BB and Khodadoust, MS and Olsson, N and Wagar, LE and Fast, E and Liu, CL and Muftuoglu, Y and Sworder, BJ and D	10.1038/s41587-019-0280-2
T follicular helper cells in human efferent lymph retain lymphoid characteristics	Vella, LA and Buggert, M and Manne, S and Herati, RS and Sayin, I and Kuri-Cervantes, L and Brody, IB and O'Boyle, KC and K	10.1172/JCI125628
Alternative Evaluation of an ELISPOT Assay Using Cytokine Activity as a Novel Parameter	Maeda, C and Iizuka, A and Miyata, H and Kondou, R and Ashizawa, T and Kanematsu, A and Watanabe, K and Deguchi, S an	10.21873/anticanres.15175
Induction of antigen-specific cytotoxic T lymphocytes by fusion cells generated from allogeneic plasmacytoid dendritic and tumor cells	Koido, S and Homma, S and Kan, S and Takakura, K and Namiki, Y and Kobayashi, H and Ito, Z and Uchiyama, K and Kajihara	10.3892/ijo.2014.2433
Position-Scanning Peptide Libraries as Particle Immunogens for Improving CD8(+) T-Cell Responses	He, XD and Zhou, SQ and Quinn, B and Huang, WC and Jahagirdar, D and Vega, M and Ortega, J and Long, MD and Ito, F and	10.1002/advs.202103023
TLR7/8 agonist treatment induces an increase in bone marrow resident dendritic cells and hematopoietic progenitor expansion and mobilization	Li, SD and Yao, JC and Li, JT and Schmidt, AP and Link, DC	10.1016/j.exphem.2021.02.001
Anticancer effects of a single intramuscular dose of a minicircle DNA vector expressing anti-CD3/CD20 in a xenograft mouse model	Pang, XJ and Chen, GC and Huang, P and Zhang, PF and Liu, J and Hou, XH and He, CY and Chen, P and Xie, YW and Zhao, J a	10.1016/j.omto.2022.02.014
Expression of tdTomato and luciferase in a murine lung cancer alters the growth and immune microenvironment of the tumor	Huang, L and Bommireddy, R and Munoz, LE and Guin, RN and Wei, CY and Ruggieri, A and Menon, AP and Li, XX and Shanm	10.1371/journal.pone.0254125
Activated integrins identify functional antigen-specific CD8(+) T cells within minutes after antigen stimulation	Dimitrov, S and Gouttefangeas, C and Besedovsky, L and Jensen, ATR and Chandran, PA and Rusch, E and Businger, R and S	10.1073/pnas.1720714115
Identification and HLA-Tetramer-Validation of Human CD4(+) and CD8(+) T Cell Responses against HCMV Proteins IE1 and IE2	Braendstrup, P and Mortensen, BK and Justesen, S and Osterby, T and Rasmussen, M and Hansen, AM and Christiansen, CB	10.1371/journal.pone.0094892
Hyperimmune plasma in three immuno-deficient patients affected by non-severe, prolonged COVID-19: a single-center experience	Cusi, MG and Conticini, E and Gandolfo, C and Anichini, G and Savellini, GG and Valente, S and Franchi, F and Scolletta, S a	10.1186/s12879-021-06321-2

Effects of therapeutic vaccination on the control of SIV in rhesus macaques with variable responsiveness to antiretroviral drugs	Tunggal, HC and Munson, PV and O'Connor, MA and Hajari, N and Dross, SE and Bratt, D and Fuller, JT and Bagley, K and Ful	10.1371/journal.pone.0253265
In silico identification and experimental validation of cellular uptake by a new cell penetrating peptide P1 derived from MARCKS	Chen, LL and Guo, XL and Wang, LD and Geng, JP and Wu, J and Hu, B and Wang, T and Li, JS and Liu, CB and Wang, H	10.1080/10717544.2021.1960922
High-throughput Screening of Human Tumor Antigen-specific CD4 T Cells, Including Neoantigen-reactive T Cells	Costa-Nunes, C and Cachot, A and Bobisse, S and Arnaud, M and Genolet, R and Baumgaertner, P and Speiser, DE and Alve	10.1158/1078-0432.CCR-18-1356
Identification of CD56(dim) subpopulation marked with high expression of GZMB/PRF1/PI-9 in CD56(+) interferon-alpha-induced dendritic cells	Watanabe, A and Togi, M and Koya, T and Taniguchi, M and Sakamoto, T and Iwabuchi, K and Kato, T and Shimodaira, S	10.1111/gtc.12844
COVID-19 vaccination in haematology patients: an Australian and New Zealand consensus position statement	McCaughan, G and Di Ciaccio, P and Ananda-Rajah, M and Gilroy, N and MacIntyre, R and Teh, B and Weinkove, R and Curn	10.1111/imj.15247
Rapid Expansion of Highly Functional Antigen-Specific T Cells from Patients with Melanoma by Nanoscale Artificial Antigen-Presenting Cells	Ichikawa, J and Yoshida, T and Isser, A and Laino, AS and Vassallo, M and Woods, D and Kim, S and Oelke, M and Jones, K ar	10.1158/1078-0432.CCR-19-3487
Immune expression in children with Wilms tumor: a pilot study	Holl, EK and Routh, JC and Johnston, AW and Frazier, V and Rice, HE and Tracy, ET and Nair, SK	10.1016/j.jpurol.2019.03.011
High affinity anti-TIM-3 and anti-KIR monoclonal antibodies cloned from healthy human individuals	Ryser, S and Estelles, A and Tenorio, E and Kauvar, LM and Gishizky, ML	10.1371/journal.pone.0181464
Isolation and light chain shuffling of a Plasmodium falciparum AMA1-specific human monoclonal antibody with growth inhibitory activity	Seidel-Greven, M and Addai-Mensah, O and Spiegel, H and Dipah, GNC and Schmitz, S and Breuer, G and Frempong, M and	10.1186/s12936-020-03548-3
Type 1 Diabetes Mellitus Donor Mesenchymal. Stromal Cells Exhibit Comparable Potency to Healthy Controls In Vitro	Davies, LC and Alm, JJ and Heldring, N and Moll, G and Gavin, C and Batsis, I and Qian, H and Sigvardsson, M and Nilsson, B	10.5966/sctm.2015-0272
Defining the Pharmacodynamic Profile and Therapeutic Index of NHS-IL12 Immunocytokine in Dogs with Malignant Melanoma	Paoloni, M and Mazcko, C and Selting, K and Lana, S and Barber, L and Phillips, J and Skorupski, K and Vail, D and Wilson, H	10.1371/journal.pone.0129954
Preclinical Pharmacokinetics, Biodistribution, and Acute Toxicity Evaluation of Caerin 1.9 Peptide in Sprague Dawley Rats	Yang, XD and Li, JJ and Chen, S and Xiao, LY and Cao, DM and Wu, XL and Li, HJ and Ni, GY and Wang, TF and Chen, GQ and	10.1155/2022/9869293
beta-Catenin in dendritic cells exerts opposite functions in cross-priming and maintenance of CD8(+) T cells through regulation of IL-10	Fu, CM and Liang, XJ and Cui, WG and Ober-Blobaum, JL and Vazzana, J and Shrikant, PA and Lee, KP and Clausen, BE and N	10.1073/pnas.1414167112
Persistent SARS-CoV-2 infection in patients with secondary antibody deficiency: successful clearance following combination casirivimab and imdevimab (REGN-COV2) monoclonal antibody therapy	Taha, Y and Wardle, H and Evans, AB and Hunter, ER and Marr, H and Osborne, W and Bashton, M and Smith, D and Burton-I	10.1186/s12941-021-00491-2
A phase I trial of cyclosporine for hospitalized patients with COVID-19	Blumberg, EA and Noll, JH and Tebas, P and Fraietta, JA and Frank, I and Marshall, A and Chew, A and Veloso, EA and Carulli	10.1172/jci.insight.155682
The Quantity of Autocrine IL-2 Governs the Expansion Potential of CD8(+) T Cells	Redeker, A and Welten, SPM and Baert, MRM and Vloemans, SA and Tiemessen, MM and Staal, FJT and Arens, R	10.4049/jimmunol.1501083
Expression of human endogenous retrovirus-K is strongly associated with the basal-like breast cancer phenotype	Johanning, GL and Malouf, GG and Zheng, XF and Esteva, FJ and Weinstein, JN and Wang-Johanning, F and Su, XP	10.1038/srep41960
Identification of Highly Cross-Reactive Mimotopes for a Public T Cell Response in Murine Melanoma	Grace, BE and Backlund, CM and Morgan, DM and Kang, BH and Singh, NK and Huisman, BD and Rappazzo, CG and Moynih	10.3389/fimmu.2022.886683
Specific Amyloid beta Clearance by a Catalytic Antibody Construct	Planque, SA and Nishiyama, Y and Sonoda, S and Lin, Y and Taguchi, H and Hara, M and Kolodziej, S and Mitsuda, Y and Gor	10.1074/jbc.M115.641738
Peptide vaccination in the presence of adjuvants in patients after hematopoietic stem cell transplantation with CD4+T cell reconstitution elicits consistent CD8+T cell responses	Schmitt, M and Schmitt, A and Wiesneth, M and Huckelhoven, A and Wu, ZG and Kuball, J and Wang, L and Schauwecker, P	10.7150/thno.18301
Antimelanoma CTL recognizes peptides derived from an ORF transcribed from the antisense strand of the 3 ' untranslated region of TRIT1	Swoboda, RK and Somasundaram, R and Caputo-Gross, L and Marincola, FM and Robbins, P and Herlyn, M and Herlyn, D	10.1038/mto.2014.9
Optimizing Flow Cytometric Analysis of Immune Cells in Samples Requiring Cryopreservation from Tumor-Bearing Mice	Carlson, PM and Mohan, M and Patel, RB and Birstler, J and Nettenstrom, L and Sheerar, D and Fox, K and Rodriguez, M and	10.4049/jimmunol.2000656
Maternal allergen-specific IgG might protect the child against allergic sensitization	Lupinek, C and Hochwallner, H and Johansson, C and Mie, A and Rigler, E and Scheynius, A and Alm, J and Valenta, R	10.1016/j.jaci.2018.11.051
Temporal dynamics of persistent germinal centers and memory B cell differentiation following respiratory virus infection	Yewdell, WT and Smolkin, RM and Belcheva, KT and Mendoza, A and Michaels, AJ and Cols, M and Angeletti, D and Yewdell,	10.1016/j.celrep.2021.109961
CAR-NK Cells Effectively Target SARS-CoV-2-Spike-Expressing Cell Lines In Vitro	Ma, MT and Badeti, S and Chen, CH and Kim, J and Choudhary, A and Honnen, B and Reichman, C and Calianese, D and Pin	10.3389/fimmu.2021.652223
Anti-bacterial antibodies in multiple myeloma patients at disease presentation, in response to therapy and in remission: implications for patient management	Chicca, IJ and Heaney, JLJ and Iqbal, G and Dunn, JA and Bowcock, S and Pratt, G and Yong, KL and Planche, TD and Richter	10.1038/s41408-020-00370-7
Plasmacytoid dendritic cells cross-prime naive CD8 T cells by transferring antigen to conventional dendritic cells through exosomes	Fu, CM and Peng, P and Loschko, J and Feng, L and Pham, P and Cui, WG and Lee, KP and Krug, AB and Jiang, AM	10.1073/pnas.2002345117
Antitumor activity and immunogenicity of recombinant vaccinia virus expressing HPV 16 E7 protein SigE7LAMP is enhanced by high-level coexpression of IGFBP-3	Musil, J and Kutinova, L and Zurkova, K and Hainz, P and Babiarova, K and Krystofova, J and Nemeckova, S	10.1038/cgt.2014.6
CD8(+) T cell response to adenovirus vaccination and subsequent suppression of tumor growth: modeling, simulation and analysis	Wang, Q and Klinke, DJ and Wang, ZJ	10.1186/s12918-015-0168-9
Pilot and Feasibility Trial Evaluating Immuno-Gene Therapy of Malignant Mesothelioma Using Intrapleural Delivery of Adenovirus-IFN alpha Combined with Chemotherapy	Sterman, DH and Alley, E and Stevenson, JP and Friedberg, J and Metzger, S and Recio, A and Moon, EK and Haas, AR and Va	10.1158/1078-0432.CCR-15-2133
A Novel Approach for the Treatment of T Cell Malignancies: Targeting T Cell Receptor V beta Families	Wang, J and Urbanska, K and Sharma, P and Nejati, R and Shaw, L and Lim, MS and Schuster, SJ and Powell, DJ	10.3390/vaccines8040631

Engineered CAR T Cells Targeting the Cancer-Associated Tn-Glycoform of the Membrane Mucin MUC1 Control Adenocarcinoma	Posey, AD and Schwab, RD and Boesteanu, AC and Steentoft, C and Mandel, U and Engels, B and Stone, JD and Madsen, TD	10.1016/j.immuni.2016.05.014
Characterization of a novel inhibitory human monoclonal antibody directed against Plasmodium falciparum Apical Membrane Antigen 1	Maskus, DJ and Krolik, M and Bethke, S and Spiegel, H and Kapelski, S and Seidel, M and Addai-Mensah, O and Reimann, A a	10.1038/srep39462
A New Hope for CD56(neg)CD16(pos) NK Cells as Unconventional Cytotoxic Mediators: An Adaptation to Chronic Diseases	Forconi, CS and Oduor, CI and Oluoch, PO and Ong'echa, JM and Munz, C and Bailey, JA and Moormann, AM	10.3389/fcimb.2020.00162
HSP110 sustains chronic NF-kappa B signaling in activated B-cell diffuse large B-cell lymphoma through MyD88 stabilization	Boudesco, C and Verhoeyen, E and Martin, L and Chassagne-Clement, C and Salmi, L and Mhaidly, R and Pangault, C and F	10.1182/blood-2017-12-819706
Diphtheria-toxin based anti-human CCR4 immunotoxin for targeting human CCR4(+) cells in vivo	Wang, ZH and Wei, M and Zhang, HP and Chen, HY and Germana, S and Huang, CA and Madsen, JC and Sachs, DH and Wan	10.1016/j.molonc.2015.04.004
Large-Scale Human Dendritic Cell Differentiation Revealing Notch-Dependent Lineage Bifurcation and Heterogeneity	Balan, S and Arnold-Schrauf, C and Abbas, A and Couespel, N and Savoret, J and Imperatore, F and Villani, AC and Manh, TP	10.1016/j.celrep.2018.07.033
Massively parallel single-cell chromatin landscapes of human immune cell development and intratumoral T cell exhaustion	Satpathy, AT and Granja, JM and Yost, KE and Qi, YY and Meschi, F and McDermott, GP and Olsen, BN and Mumbach, MR an	10.1038/s41587-019-0206-z
Isolation Protocol of Mouse Monocyte-derived Dendritic Cells and Their Subsequent In Vitro Activation with Tumor Immune Complexes	Santana-Magal, N and Rasoulouniriana, D and Saperia, C and Gutwillig, A and Rider, P and Engleman, EG and Carmi, Y	10.3791/57188
Neutralization of hepatitis B virus by a novel DNA-encoded monoclonal antibody	Zankharia, US and Kudchodkar, S and Khoshnejad, M and Perales-Puchalt, A and Choi, H and Ho, M and Zaidi, F and Ugen, K	10.1080/21645515.2020.1763686
Development of Allogeneic Stem Cell-Based Platform for Delivery and Potentiation of Oncolytic Virotherapy	Nguyen, DH and Herrmann, T and Hartl, B and Draganov, D and Minev, I and Neuharth, F and Gomez, A and Alamillo, A and S	10.3390/cancers14246136
Targeting prohibitins at the cell surface prevents Th17-mediated autoimmunity	Buehler, U and Schulenburg, K and Yurugi, H and Solman, M and Abankwa, D and Ulges, A and Tenzer, S and Bopp, T and Thi	10.15252/embj.201899429
Systemic Agonistic Anti-CD40 Treatment of Tumor-Bearing Mice Modulates Hepatic Myeloid-Suppressive Cells and Causes Immune-Mediated Liver Damage	Medina-Echeverz, J and Ma, C and Duffy, AG and Eggert, T and Hawk, N and Kleiner, DE and Korangy, F and Greten, TF	10.1158/2326-6066.CIR-14-0182
A New Pipeline to Predict and Confirm Tumor Neoantigens Predict Better Response to Immune Checkpoint BlockadeS	Lazdun, Y and Si, H and Creasy, T and Ranade, K and Higgs, BW and Streicher, K and Durham, NM	10.1158/1541-7786.MCR-19-1118
Bystander T-Cells Support Clonal T-Cell Activation by Controlling the Release of Dendritic Cell-Derived Immune-Stimulatory Extracellular Vesicles	Lindenbergh, MFS and Koerhuis, DGJ and Borg, EGF and van't Veld, EM and Driedonks, TAP and Wubbolts, R and Stoorvogel,	10.3389/fimmu.2019.00448
Future perspectives of therapeutic monoclonal antibodies	Tsumoto, K and Isozaki, Y and Yagami, H and Tomita, M	10.2217/imt-2018-0130
Predicting CD4 T-cell epitopes based on antigen cleavage, MHCII presentation, and TCR recognition	Schneidman-Duhovny, D and Khuri, N and Dong, GQ and Winter, MB and Shifrut, E and Friedman, N and Craik, CS and Pratt	10.1371/journal.pone.0206654
Low Energy Electron Irradiation Is a Potent Alternative to Gamma Irradiation for the Inactivation of (CAR-)NK-92 Cells in ATMP Manufacturing	Walcher, L and Kistenmacher, AK and Sommer, C and Bohlen, S and Ziemann, C and Dehmel, S and Braun, A and Tretbar, U	10.3389/fimmu.2021.684052
Autologous tumor cell vaccination combined with systemic CpG-B and IFN- promotes immune activation and induces clinical responses in patients with metastatic renal cell carcinoma: a phase II trial	Koster, BD and Santegoets, SJAM and Harting, J and Baars, A and van Ham, SM and Scheper, RJ and Hooijberg, E and de Gru	10.1007/s00262-019-02320-0
Effect of atorvastatin on humoral immune response to 23-valent pneumococcal polysaccharide vaccination in healthy volunteers: The StatVax randomized clinical trial	Wildes, TJ and Grippin, A and Fasanya, H and Dyson, KA and Brantly, M	10.1016/j.vaccine.2019.01.023
Prevention of allergy by virus-like nanoparticles (VNP) delivering shielded versions of major allergens in a humanized murine allergy model	Kratzer, B and Kohler, C and Hofer, S and Smole, U and Trapin, D and Iturri, J and Pum, D and Kienzl, P and Elbe-Burger, A an	10.1111/all.13573
Pharmacological inhibition of p110 delta subunit of PI3K confers protection against experimental leishmaniasis	Khadem, F and Jia, P and Mou, ZR and Barazandeh, AF and Liu, D and Keynan, Y and Uzonna, JE	10.1093/jac/dkw448
Development of off-the-shelf hematopoietic stem cell-engineered invariant natural killer T cells for COVID-19 therapeutic intervention	Li, YR and Dunn, ZS and Garcia, G and Carmona, C and Zhou, Y and Lee, D and Yu, JJ and Huang, J and Kim, JT and Arumugas	10.1186/s13287-022-02787-2
Memory T Cell Proliferation before Hepatitis C Virus Therapy Predicts Antiviral Immune Responses and Treatment Success	Mendez-Lagares, G and Lu, D and Chen, C and Terrault, N and Segal, MR and Khalili, M and Monto, A and Shen, H and Mano	10.4049/jimmunol.1701364
IL-6/IFN-gamma double knockdown CAR-T cells reduce the release of multiple cytokines from PBMCs in vitro	Zhang, HH and Lv, XF and Kong, QF and Tan, Y	10.1080/21645515.2021.2016005
Pathogenic mechanisms and therapeutic promise of phytochemicals and nanocarriers based drug delivery against radiotherapy-induced neurotoxic manifestations	Iqubal, A and Iqubal, MK and Sharma, S and Wasim, M and Alfaleh, MA and Md, S and Baboota, S and Ali, J and Haque, SE	10.1080/10717544.2022.2064562
Prevalence of human papillomavirus in Jeddah, Saudi Arabia	Mousa, M and Al-amri, SS and Degnah, AA and Tolah, AM and Abduljabbar, HH and Oraif, AM and Abduljabbar, HS and Mirza	10.5144/0256-4947.2019.403
Phenotype, specificity and avidity of antitumour CD8(+) T cells in melanoma	Oliveira, G and Stromhaug, K and Klaeger, S and Kula, T and Frederick, DT and Le, PM and Forman, J and Huang, T and Li, SQ	10.1038/s41586-021-03704-y
Consensus guidelines for the treatment of invasive mould infections in haematological malignancy and haemopoietic stem cell transplantation, 2014	Blyth, CC and Gilroy, NM and Guy, SD and Chambers, ST and Cheong, EY and Gottlieb, T and McGuinness, SL and Thursky, K	10.1111/imj.12598
Postvaccination graft dysfunction/aplastic anemia relapse with massive clonal expansion of autologous CD8(+) lymphocytes	Ritz, C and Meng, WZ and Stanley, NL and Baroja, ML and Xu, C and Yan, P and Huang, AC and Hausler, R and Nicholas, P ar	10.1182/bloodadvances.2019000853
The Breadth of Expandable Memory CD8(+) T Cells Inversely Correlates with Residual Viral Loads in HIV Elite Controllers	Ndhlovu, ZM and Stampouloglou, E and Cesa, K and Mavrothalassitis, O and Alvino, DM and Li, JZ and Wilton, S and Karel, D	10.1128/JVI.01527-15

Small Molecule Inhibitors of MERTK and FLT3 Induce Cell Cycle Arrest in Human CD8(+) T Cells	Powell, RM and Peeters, MJW and Rahbech, A and Aehnlich, P and Seremet, T and Straten, PT	10.3390/vaccines9111294
The CD8(+) Memory Stem T Cell (T-SCM) Subset Is Associated with Improved Prognosis in Chronic HIV-1 Infection	Ribeiro, SP and Milush, JM and Cunha-Neto, E and Kallas, EG and Kalil, J and Somsouk, M and Hunt, PW and Deeks, SG and	10.1128/JVI.01948-14
MHC class I loaded ligands from breast cancer cell lines: A potential HLA-I-typed antigen collection	Rozanov, DV and Rozanov, ND and Chiotti, KE and Reddy, A and Wilmarth, PA and David, LL and Cha, SW and Woo, S and Pe	10.1016/j.jprot.2018.01.004
Both Methylation and Copy Number Variation Participated in the Varied Expression of PRAME in Multiple Myeloma	Yang, L and Dao, FT and Chang, Y and Wang, YZ and Li, LD and Chen, WM and Long, LY and Liu, YR and Lu, J and Liu, KY and	10.2147/OTT.S240979
Virus-Like Particle-Drug Conjugates Induce Protective, Long-lasting Adaptive Antitumor Immunity in the Absence of Specifically Targeted Tumor Antigens	Kines, RC and Thompson, CD and Spring, S and Li, ZY and de los Pinos, E and Monks, S and Schiller, JT	10.1158/2326-6066.CIR-19-0974
Phytochemical-Based Nano-Pharmacotherapeutics for Management of Burn Wound Healing	Qadir, A and Jahan, S and Aqil, M and Warsi, MH and Alhakamy, NA and Alfaleh, MA and Khan, N and Ali, A	10.3390/gels7040209
Lymphopenia in COVID-19: gamma delta T Cells-Based Therapeutic Opportunities	Lo Presti, E and Dieli, F and Meraviglia, S	10.3390/vaccines9060562
Ablation of CD8(+) T cell recognition of an immunodominant epitope in SARS-CoV-2 Omicron variants BA.1, BA.2 and BA.3	Swaminathan, S and Lineburg, KE and Panikkar, A and Raju, J and Murdolo, LD and Szeto, C and Crooks, P and Le Texier, L a	10.1038/s41467-022-34180-1
Analysis of Major Histocompatibility Complex-Bound HIV Peptides Identified from Various Cell Types Reveals Common Nested Peptides and Novel T Cell Responses	Rucevic, M and Kourjian, G and Boucau, J and Blatnik, R and Bertran, WG and Berberich, MJ and Walker, BD and Riemer, AB	10.1128/JVI.00599-16
Preinfusion polyfunctional anti-CD19 chimeric antigen receptor T cells are associated with clinical outcomes in NHL	Rossi, J and Paczkowski, P and Shen, YW and Morse, K and Flynn, B and Kaiser, A and Ng, C and Gallatin, K and Cain, T and F	10.1182/blood-2018-01-828343
Vaccinia virus E3 prevents sensing of Z-RNA to block ZBP1-dependent necroptosis	Koehler, H and Cotsmire, S and Zhang, T and Balachandran, S and Upton, JW and Langland, J and Kalman, D and Jacobs, BL	10.1016/j.chom.2021.05.009
Rexinoids Modulate Effector T Cell Expression of Mucosal Homing Markers CCR9 and alpha 4 beta 7 Integrin and Direct Their Migration In Vitro	Manhas, KR and Marshall, PA and Wagner, CE and Jurutka, PW and Mancenido, MV and Debray, HZ and Blattman, JN	10.3389/fimmu.2022.746484
In vitro induction of T regulatory cells by a methylated CpG DNA sequence in humans: Potential therapeutic applications in allergic and autoimmune diseases	Lawless, OJ and Bellanti, JA and Brown, ML and Sandberg, K and Umans, JG and Zhou, L and Sandberg, K and Umans, JG and	10.2500/aap.2018.39.4113
Structural Determination of the Broadly Reactive Anti-IGHV1-69 Anti-idiotypic Antibody G6 and Its Idiotope	Avnir, Y and Prachanronarong, KL and Zhang, Z and Hou, S and Peterson, EC and Sui, JH and Zayed, H and Kurella, VB and M	10.1016/j.celrep.2017.11.056
Addition of CpG ODN and Poly (I:C) to a standard maturation cocktail generates monocyte-derived dendritic cells and induces a potent Th1 polarization with migratory capacity	Zhu, M and Xu, W and Su, H and Huang, Q and Wang, BL	10.1080/21645515.2015.1046659
Green Nanoemulsion Stabilized by In Situ Self-Assembled Natural Oil/Native Cyclodextrin Complexes: An Eco-Friendly Approach for Enhancing Anticancer Activity of Costunolide against Lung Cancer Cells	Alhakamy, NA and Badr-Eldin, SM and Ahmed, OAA and Aldawsari, HM and Okbazghi, SZ and Alfaleh, MA and Abdulaal, WH	10.3390/pharmaceutics14020227
Spatiotemporal evolution of venous narrowing in acute MS lesions	Eisele, P and Szabo, K and Ebert, A and Brueck, W and Platten, M and Gass, A	10.1212/NXI.0000000000000440
HiChIRP reveals RNA-associated chromosome conformation	Mumbach, MR and Granja, JM and Flynn, RA and Roake, CM and Satpathy, AT and Rubin, AJ and Qi, YY and Jiang, ZZ and Sha	10.1038/s41592-019-0407-x
Quick and Easy Assembly of a One-Step qRT-PCR Kit for COVID-19 Diagnostics Using In-House Enzymes	Takahashi, M and Tehseen, M and Salunke, R and Takahashi, E and Mfarrej, S and Sobhy, MA and Alhamlan, FS and Hala, S a	10.1021/acsomega.0c05635
ERBB2-CAR-Engineered Cytokine-Induced Killer Cells Exhibit Both CAR-Mediated and Innate Immunity Against High-Risk Rhabdomyosarcoma	Merker, M and Wagner, J and Kreyenberg, H and Heim, C and Moser, LM and Wels, WS and Bonig, H and Ivics, Z and Ullrich,	10.3389/fimmu.2020.581468
Consensus guidelines for improving patients' understanding of invasive fungal disease and related risk prevention in the haematology/oncology setting, 2021	Fernando, SS and Paige, EK and Dendle, C and Weinkove, R and Kong, DCM and Omond, P and Routledge, DJ and Szer, J an	10.1111/imj.15593
Large expert-curated database for benchmarking document similarity detection in biomedical literature search	Brown, P and Tan, AC and El-Esawi, MA and Liehr, T and Blanck, O and Gladue, DP and Almeida, GMF and Cernava, T and So	10.1093/database/baz085
Cysteine-binding adjuvant enhances survival and promotes immune function in a murine model of acute myeloid leukemia	Slezak, Anna J. and Chang, Kevin and Beckman, Taryn N. and Refvik, Kirsten C. and Alpar, Aaron T. and Lauterbach, Abigail L	https://doi.org/10.1182/bloodadvances.2023012529
Immunotherapy in leukaemia.	Mu X and Chen C and Dong L and Kang Z and Sun Z and Chen X and Zheng J and Zhang Y	10.3724/abbs.2023101
Combination of AFP vaccine and immune checkpoint inhibitors slows hepatocellular carcinoma progression in preclinical models.	Lu X and Deng S and Xu J and Green BL and Zhang H and Cui G and Zhou Y and Zhang Y and Xu H and Zhang F and Mao R and	10.1172/JCI163291
Dabrafenib plus trametinib in BRAFV600E-mutated rare cancers: the phase 2 ROAR trial.	Subbiah V and Kreitman RJ and Wainberg ZA and Gazzah A and Lassen U and Stein A and Wen PY and Dietrich S and de Jong	10.1038/s41591-023-02321-8
T Cell Based Immunotherapy for Cancer: Approaches and Strategies.	Want MY and Bashir Z and Najar RA	10.3390/vaccines11040835
Role of tumor microenvironment in cancer progression and therapeutic strategy.	Wang Q and Shao X and Zhang Y and Zhu M and Wang FXC and Mu J and Li J and Yao H and Chen K	10.1002/cam4.5698
A perspective of immunotherapy for acute myeloid leukemia: Current advances and challenges.	Chen Y and Wang J and Zhang F and Liu P	10.3389/fphar.2023.1151032
Cancer Resistance to Immunotherapy: Comprehensive Insights with Future Perspectives.	Said SS and Ibrahim WN	10.3390/pharmaceutics15041143
Self-replicating vehicles based on negative strand RNA viruses.	Lundstrom K	10.1038/s41417-022-00436-7
How Can We Prevent Mother-to-Child Transmission of HTLV-1?	Itabashi K and Miyazawa T and Uchimaru K	10.3390/ijms24086961
Modulating the immune system as a therapeutic target for myelodysplastic syndromes and acute myeloid leukemia.	Putnam C and Kondeti L and Kesler M and Varney M	10.1139/bcb-2022-0374
Checkpoint Immunotherapy in Pediatric Oncology: Will We Say Checkmate Soon?	Ciurej A and Lewis E and Gupte A and Al-Antary E	10.3390/vaccines11121843
Role of Next Generation Immune Checkpoint Inhibitor (ICI) Therapy in Philadelphia Negative Classic Myeloproliferative Neoplasm (MPN): Review of the Literature.	Yadav R and Hakobyan N and Wang JC	10.3390/ijms241512502

Advanced strategies in improving the immunotherapeutic effect of CAR-T cell therapy.	Wang M and Jia L and Dai X and Zhang X	10.1002/1878-0261.13621
Immunotherapies of acute myeloid leukemia: Rationale, clinical evidence and perspective.	Wu Y and Li Y and Gao Y and Zhang P and Jing Q and Zhang Y and Jin W and Wang Y and Du J and Wu G	10.1016/j.biopha.2024.116132
Dendritic Cells as a Therapeutic Strategy in Acute Myeloid Leukemia: Vaccines.	Palomares F and Pina A and Dakhaoui H and Leiva-Castro C and Munera-Rodriguez AM and Cejudo-Guillen M and Granados	10.3390/vaccines12020165
Combination of cancer vaccine with CD122-biased IL-2/anti-IL-2 Ab complex shapes the stem-like effector NK and CD8(+) T cells against tumor.	Shimizu K and Ueda S and Kawamura M and Aoshima H and Satoh M and Nakabayashi J and Fujii SI	10.1136/jitc-2022-006409
Immunoreactivity to WT1 peptide vaccine is associated with prognosis in elderly patients with acute myeloid leukemia: follow-up study of randomized phase II trial of OCV-501, an HLA class II-binding WT1 polypeptide.	Naoe T and Saito A and Hosono N and Kasahara S and Muto H and Hatano K and Ogura M and Masunari T and Tanaka M and	10.1007/s00262-023-03432-4
Biological Effects of Small Sized Graphene Oxide Nanosheets on Human Leukocytes.	Aventaggiato M and Valentini F and Caissutti D and Relucenti M and Tafani M and Misasi R and Zicari A and Di Martino S and	10.3390/biomedicines12020256
Protocol for a multicentre randomised controlled trial examining the effects of temporarily pausing Bruton tyrosine kinase inhibitor therapy to coincide with SARS-CoV-2 vaccination and its impact on immune responses in patients with chronic lymphocytic leukaemia.	Barber VS and Peckham N and Duley L and Francis A and Abhishek A and Moss P and Cook JA and Parry HM	10.1136/bmjopen-2023-077946
Randomized controlled trial of molnupiravir SARS-CoV-2 viral and antibody response in at-risk adult outpatients.	Standing JF and Buggiotti L and Guerra-Assuncao JA and Woodall M and Ellis S and Agyeman AA and Miller C and Okechukw	10.1038/s41467-024-45641-0
Bioengineered small extracellular vesicles deliver multiple SARS-CoV-2 antigenic fragments and drive a broad immunological response.	Jackson HK and Long HM and Yam-Puc JC and Palmulli R and Haigh TA and Gerber PP and Lee JS and Matheson NJ and Youn	10.1002/jev2.12412
Photothermal Prussian blue nanoparticles generate potent multi-targeted tumor-specific T cells as an adoptive cell therapy	Sweeney, E.E. and Sekhri, P. and Muniraj, N. and Chen, J. and Feng, S. and Terao, J. and Chin, S.J. and Schmidt, D.E. and Bol	10.1002/btm2.10639
Epidemiology and risk factors for the development of infectious complications in newly diagnosed multiple myeloma: a multicenter prospective cohort study in Uruguay	Riva, E. and Garrido, D. and Villano, F. and Bove, V.	10.1016/j.htct.2023.09.2362
The lncRNA TRG-AS1 promotes the growth of colorectal cancer cells through the regulation of P2RY10/GNA13	Shi, L. and Luo, B. and Deng, L. and Zhang, Q. and Li, Y. and Sun, D. and Zhang, H. and Zhuang, L.	10.1080/00365521.2024.2318363
Oxidized mRNA Lipid Nanoparticles for In Situ Chimeric Antigen Receptor Monocyte Engineering	Mukalel, A.J. and Hamilton, A.G. and Billingsley, M.M. and Li, J. and Thatte, A.S. and Han, X. and Safford, H.C. and Padilla, M	10.1002/adfm.202312038
Evaluation of the safety and efficiency of cytotoxic T cell therapy sensitized by tumor antigens original from T-ALL-iPSC in vivo	Li, W. and Zhou, M. and Wang, L. and Huang, L. and Chen, X. and Sun, X. and Liu, T.	10.1002/cai2.95
Development of New CD38 Targeted Peptides for Cancer Imaging	Zheleznyak, A. and Tang, R. and Duncan, K. and Manion, B. and Liang, K. and Xu, B. and Vanover, A. and Ghai, A. and Prior, J.	10.1007/s11307-024-01901-5
Shortwave-infrared-light-emitting probes for the in vivo tracking of cancer vaccines and the elicited immune responses	Ren, F. and Wang, F. and Baghdasaryan, A. and Li, Y. and Liu, H. and Hsu, R.S. and Wang, C. and Li, J. and Zhong, Y. and Sala	10.1038/s41551-023-01083-5
Dynamics of The Γδtcr Repertoires During The Dedifferentiation Process and Pilot Implications for Immunotherapy of Thyroid Cancer	Hao, Q. and Li, R. and Li, H. and Rui, S. and You, L. and Zhang, L. and Zhao, Y. and Li, P. and Li, Y. and Kong, X. and Chen, H.	10.1002/advs.202306364
Allogeneic “Zombie Cell” as Off-The-Shelf Vaccine for Postsurgical Cancer Immunotherapy	Li, B. and Zhang, P. and Li, J. and Zhou, R. and Zhou, M. and Liu, C. and Liu, X. and Chen, L. and Li, L.	10.1002/advs.202307030
Leveraging Aptamer-Based DNA Nanotechnology for Bioanalysis and Cancer Therapeutics	Huang, Z. and Wang, D. and Zhang, Q. and Zhang, Y. and Peng, R. and Tan, W.	10.1021/accountsmr.3c00249
High-dimensional multi-pass flow cytometry via spectrally encoded cellular barcoding	Kwok, S.J.J. and Forward, S. and Fahlberg, M.D. and Assita, E.R. and Cosgriff, S. and Lee, S.H. and Abbott, G.R. and Zhu, H. a	10.1038/s41551-023-01144-9
Covid Antibody Titers in Cancer Patients Following Vaccination with ChAdOx1 nCOV-19 Vaccine	Chavan, A. and Shriyan, B. and Chavan, P. and Shirsat, A. and Gavhane, U. and Pillai, B. and Bhat, V. and Dhamne, C. and G	10.1055/s-0043-1771273
Targeting PRMT9-mediated arginine methylation suppresses cancer stem cell maintenance and elicits cGAS-mediated anticancer immunity	Dong, H. and He, X. and Zhang, L. and Chen, W. and Lin, Y.-C. and Liu, S.-B. and Wang, H. and Nguyen, L.X.T. and Li, M. and	10.1038/s43018-024-00736-x
Design of a mucin-selective protease for targeted degradation of cancer-associated mucins	Pedram, K. and Shon, D.J. and Tender, G.S. and Mantuano, N.R. and Northey, J.J. and Metcalf, K.J. and Wisnovsky, S.P. and F	10.1038/s41587-023-01840-6
EPI-X4, a CXCR4 antagonist inhibits tumor growth in pancreatic cancer and lymphoma models	Sagini, M.N. and Zepp, M. and Eyol, E. and Ali, D.M. and Gromova, S. and Dahlmann, M. and Behrens, D. and Groeschel, C. a	10.1016/j.peptides.2023.171111
Engineering Antigen-Specific Tolerance to an Artificial Protein Hydrogel	Rapp, P.B. and Baccile, J.A. and Galimidi, R.P. and Vielmetter, J.	10.1021/acsbiomaterials.3c01430
Prolonged viral pneumonia and high mortality in COVID-19 patients on anti-CD20 monoclonal antibody therapy	Feuth, E. and Nieminen, V. and Palomäki, A. and Ranti, J. and Sucksdorff, M. and Finnilä, T. and Oksi, J. and Vuorinen, T. and	10.1007/s10096-024-04776-0
PLGA microparticle formulations for tunable delivery of a nano-engineered filamentous bacteriophage-based vaccine: in vitro and in silico-supported approach	Jamaledin, R. and Sartorius, R. and Di Natale, C. and Onesto, V. and Manco, R. and Mollo, V. and Vecchione, R. and De Bera	10.1007/s40097-022-00519-9
Upregulation of IL4-induced gene 1 enzyme by B2 cells during melanoma progression impairs their antitumor properties	Bekkat, F. and Seradj, M. and Lengagne, R. and Fiore, F. and Kato, M. and Lucas, B. and Castellano, F. and Molinier-Frenkel,	10.1002/eji.202350615
Engineering a Programmed Death-Ligand 1-Targeting Monobody Via Directed Evolution for SynNotch-Gated Cell Therapy	Zhu, L. and Man, C.-W. and Harrison, R.E. and Wu, Z. and Limsakul, P. and Peng, Q. and Hashimoto, M. and Mamaril, A.P. ar	10.1021/acsnano.4c01597
Transient inhibition of neutrophil functions enhances the antitumor effect of intravenously delivered oncolytic vaccinia virus	Zhou, D. and Xu, W. and Ding, X. and Guo, H. and Wang, J. and Zhao, G. and Zhang, C. and Zhang, Z. and Wang, Z. and Wang	10.1111/cas.16105
Entropy-Driven Strand Displacements Around DNA Tetrahedron for Sensitive Detection and Intracellular Imaging of mRNA	Zhu, J. and Qu, X. and Zhuang, Y. and Miao, P.	10.1002/sstr.202300420
Evaluating the Validation Process: Embracing Complexity and Transparency in Health Economic Modelling	Corro Ramos, I. and Feenstra, T. and Ghabri, S. and Al, M.	10.1007/s40273-024-01364-0
Prediction of tumor-reactive T cell receptors from scRNA-seq data for personalized T cell therapy	Tan, C.L. and Lindner, K. and Boschert, T. and Meng, Z. and Rodriguez Ehrenfried, A. and De Roia, A. and Haltenhof, G. and F	10.1038/s41587-024-02161-y

Enhancing CAR Macrophage Efferocytosis Via Surface Engineered Lipid Nanoparticles Targeting LXR Signaling	Chuang, S.T. and Stein, J.B. and Nevins, S. and Kilic Bektas, C. and Choi, H.K. and Ko, W.-K. and Jang, H. and Ha, J. and Lee,	10.1002/adma.202308377
Remuneration of donors for cell and gene therapies: an update on the principles and perspective of the World Marrow Donor Association	Hamad, L. and Ahmed, S.M. and van Eerden, E. and van Walraven, S.M. and Machin, L.	10.1038/s41409-024-02246-x
Siglec15/TGF-β bispecific antibody mediates synergistic anti-tumor response against 4T1 triple negative breast cancer in mice	Shen, L. and Schaefer, A.M. and Tiruthani, K. and Wolf, W. and Lai, S.K.	10.1002/btm2.10651
Multimodal stimulation screens reveal unique and shared genes limiting T cell fitness	Lin, C.-P. and Levy, P.L. and Alflen, A. and Apriamashvili, G. and Ligtenberg, M.A. and Vredevoogd, D.W. and Bleijerveld, O.B	10.1016/j.ccell.2024.02.016
TGF-β signaling pathway-related genes in predicting the prognosis of colon cancer and guiding immunotherapy	Chen, J. and Ji, C. and Liu, S. and Wang, J. and Wang, C. and Pan, J. and Qiao, J. and Liang, Y. and Cai, M. and Ma, J.	10.1016/j.cpt.2023.12.002
Glutarate regulates T cell metabolism and anti-tumour immunity	Minogue, E. and Cunha, P.P. and Wadsworth, B.J. and Grice, G.L. and Sah-Teli, S.K. and Hughes, R. and Bargiela, D. and Qua	10.1038/s42255-023-00855-2
RBD- specific Th1 responses are associated with vaccine-induced protection against SARS-CoV-2 infection in patients with hematological malignancies	Bigenwald, C and Haddad, Y and Thelemaque, C and Carrier, A and Birebent, R and Ly, P and Flament, C and Lahmar, I and	10.1080/2162402X.2022.2163785
Current status and future prospects of chimeric antigen receptor-T cell therapy in lymphoma research: A bibliometric analysis	Ou, LJ and Su, C and Liang, L and Duan, QT and Li, YF and Zang, H and He, YZ and Zeng, RL and Li, YJ and Zhou, H and Xiao, L	10.1080/21645515.2023.2267865
Bioengineered small extracellular vesicles deliver multiple SARS-CoV-2 antigenic fragments and drive a broad immunological response	Jackson, HK and Long, HM and Yam-Puc, JC and Palmulli, R and Haigh, TA and Gerber, PP and Lee, JS and Matheson, NJ and	10.1002/jev2.12412

Second step selection list

Computational or in vitro approaches	CAR-T or other cell adoptive transfer studies	Immunotherapy without a clear protocol for therapeutic vaccination	Title	Authors	doi
x			TAX and HBZ: hFc Ɣ 1 proteins as targets for passive immunotherapy	Akbarin MM, Rafatpanah H, Soleimanpour S, Amini AA, Arian A, Mosavat A, Rezaee SA.	10.22038/IJBMS.2022.64787.14266
x			Chronic lymphocytic leukemia monitoring with a Lamprey idiotope-specific antibody	Nakahara H, Herrin BR, Alder MN, Catera R, Yan XJ, Chiorazzi N, Cooper MD.	10.1158/2326-6066.CIR-13-0052
x			Prevalence of the BCR/ABL fusion gene and T cell stimulation capacity of dendritic cells in chronic myelogenous leukemia	Gaafar A, Al-Omar HM, Manogaran PS, Almohareb F, Alhussein K.	
	x		Antitumor activity of CAR-T cells targeting the intracellular oncoprotein WT1 can be enhanced by vaccination		https://doi.org/10.1182/blood-2017-08-802926
	x		EBAG9 silencing exerts an immune checkpoint function without aggravating adverse effects		https://doi.org/10.1016/j.ymthe.2022.07.009
	x		CAR-tropic extracellular vesicles carry tumor-associated antigens and modulate CAR T cell functionality	Ukrainskaya V.M., Musatova O.E., Volkov D.V., Osipova D.S., Pershin D.S., Moysenovich A.M., Evtushenko E.G., Kulakovskaya E.A., Maksimov E.G., Zhang H., Rubtsov Y.P., Maschan M.A., Stepanov A.V., Gabibov A.G.	10.1038/s41598-023-27604-5
x			Trypanosoma cruzi Antigenic Proteins Shared with Acute Lymphoblastic Leukemia and Neuroblastoma	Eligio García L., Crisóstomo Vázquez M.D.P., Maravelez Acosta V.A., Soria Guerrero M., Cortés Campos A., Jiménez Cardoso E.	10.3390/ph15111421
	x		Development of a T cell-redirecting bispecific antibody targeting B-cell maturation antigen for the suppression of multiple myeloma cell growth	Huo J., Huang Y., Zheng Z., Tay X.N., Mahfut F.B., Zhang W., Lam K.-P., Yang Y., Xu S.	10.1093/abt/tbac012
	x		Human T cells engineered with a leukemia lipid-specific TCR enables donor-unrestricted recognition of CD1c-expressing leukemia	Consonni M., Garavaglia C., Grilli A., de Lalla C., Mancino A., Mori L., De Libero G., Montagna D., Casucci M., Serafini M., Bonini C., Häussinger D., Ciceri F., Bernardi M., Mastaglio S., Bicciato S., Dellabona P., Casorati G.	10.1038/s41467-021-25223-0
	x		Antibodies against vaccine-preventable infections after CAR-T cell therapy for B cell malignancies	Walti C.S., Krantz E.M., Maalouf J., Boonyaratanakornkit J., Keane-Candib J., Joncas-Schronce L., Stevens-Ayers T., Dasgupta S., Taylor J.J., Hirayama A.V., Bar M., Gardner R.A., Cowan A.J., Green D.J., Boeckh M.J., Maloney D.G., Turtle C.J., Hill J.A.	10.1172/jci.insight.146743
		x	TNB-486 induces potent tumor cell cytotoxicity coupled with low cytokine release in preclinical models of B-NHL	Malik-Chaudhry H.K., Prabhakar K., Ugamraj H.S., Boudreau A.A., Buelow B., Dang K., Davison L.M., Harris K.E., Jorgensen B., Ogana H., Pham D., Schellenberger U., Van Schooten W., Buelow R., Iyer S., Trinklein N.D., Rangaswamy U.S.	10.1080/19420862.2021.1890411
	x		Transgenic CD8αβ co-receptor rescues endogenous TCR function in TCR-transgenic virus-specific T cells	Bajwa G., Lanz I., Cardenas M., Brenner M.K., Arber C.	10.1136/jitc-2020-001487
		x	Antagonistic anti-LILRB1 monoclonal antibody regulates antitumor functions of natural killer cells	Chen H., Chen Y., Deng M., John S., Gui X., Kansagra A., Chen W., Kim J., Lewis C., Wu G., Xie J., Zhang L., Huang R., Liu X., Arase H., Huang Y., Yu H., Luo W., Xia N., Zhang N., An Z., Zhang C.C.	10.1136/jitc-2019-000515
		x	Combination immunotherapy with anti-PD-L1 antibody and depletion of regulatory T cells during acute viral infections results in improved virus control but lethal immunopathology	David P., Drabczyk-Pluta M., Pastille E., Knuschke T., Werner T., Honke N., Megger D.A., Akhmetzyanova I., Shaabani N., Eyking-Singer A., Cario E., Kershaw O., Gruber A.D., Tenbusch M., Dietze K.K., Trilling M., Liu J., Schadendorf D., Streeck H., Lang K.S., Xie Y., Zimmer L., Sitek B., Paschen A., Westendorf A.M., Dittmer U., Zelinskyy G.	10.1371/journal.ppat.1008340
	x		Activated Allogeneic Donor-derived Marrow-infiltrating Lymphocytes Display Measurable In Vitro Antitumor Activity	Biavati L., Noonan K., Luznik L., Borrello I.	10.1097/CJI.0000000000000256
	x		Murine pre–B-cell ALL induces T-cell dysfunction not fully reversed by introduction of a chimeric antigen receptor	Qin H., Ishii K., Nguyen S., Su P.P., Burk C.R., Kim B.-H., Duncan B.B., Tarun S., Shah N.N., Kohler M.E., Fry T.J.	10.1182/blood-2017-12-815548
	x		Safety and persistence of WT1-specific T-cell receptor gene2transduced lymphocytes in patients with AML and MDS	Tawara I., Kageyama S., Miyahara Y., Fujiwara H., Nishida T., Akatsuka Y., Ikeda H., Tanimoto K., Terakura S., Murata M., Inaguma Y., Masuya M., Inoue N., Kidokoro T., Okamoto S., Tomura D., Chono H., Nukaya I., Mineno J., Naoe T., Emi N., Yasukawa M., Katayama N., Shiku H.	10.1182/blood-2017-06-791202
		x	STING activation reverses lymphoma-mediated resistance to antibody immunotherapy	Dahal L.N., Dou L., Hussain K., Liu R., Earley A., Cox K.L., Murinello S., Tracy I., Forconi F., Steele A.J., Duriez P.J., Gomez-Nicola D., Teeling J.L., Glennie M.J., Cragg M.S., Beers S.A.	10.1158/0008-5472.CAN-16-2784
x			CD20-specific immunoligands engaging NKG2D enhance γδ T cell-mediated lysis of lymphoma cells	Peipp M., Wesch D., Oberg H.-H., Lutz S., Muskulus A., Van De Winkel J.G.J., Parren P.W.H.I., Burger R., Humpe A., Kabelitz D., Gramatzki M., Kellner C.	10.1111/sji.12581
	x		CD19 isoforms enabling resistance to CART-19 immunotherapy are expressed in B-ALL patients at initial diagnosis	Fischer J., Paret C., El Malki K., Alt F., Wingerter A., Neu M.A., Kron B., Russo A., Lehmann N., Roth L., Fehr E.-M., Attig S., Hohberger A., Kindler T., Faber J.	10.1097/CJI.0000000000000169
x			T cell-recruiting triplebody 19-3-19 mediates serial lysis of malignant B-lymphoid cells by a single T cell	Roskopf C.C., Schiller B. C.B., Braciak A. T.A., Kobold S., Schubert A. I.A., Fey H. G.H., Hopfner K.-P., Oduncu S. F.S.	10.18632/oncotarget.2238
x			B-CLL cells acquire APC- and CTL-like phenotypic characteristics after stimulation with CpG ODN and IL-21	Hagn M., Blackwell S.E., Beyer T., Ebel V., Fabricius D., Lindner S., Stilgenbauer S., Simmet T., Tam C., Neeson P., Trapani J.A., Schrezenmeier H., Weiner G.J., Jahrsdörfer B.	10.1093/intimm/dxu001
		x	Cancer-associated CD43 glycoforms as target of immunotherapy	Tuccillo F.M., Palmieri C., Fiume G., De Laurentiis A., Schiavone M., Falcone C., Iaccino E., Galandrini R., Capuano C., Santoni A., D'Armiento F.P., Arra C., Barbieri A., Dal Piaz F., Venzon D., Bonelli P., Buonaguro F.M., Scala I., Mallardo M., Quinto I., Scala G.	10.1158/1535-7163.MCT-13-0651
	x		Minor Antigen Distribution Predicts Site-Specific Graft-versus-Tumor Activity of Adoptively Transferred, Minor Antigen-Specific CD8 T Cells	Shand J.C., Qin H., Nasholm N., Capitini C.M., Fry T.J.	10.1016/j.bbmt.2013.10.009
x			A blood dendritic cell vaccine for acute myeloid leukemia expands anti-tumor T cell responses at remission	Hsu, JL and Bryant, CE and Papadimitrious, MS and Kong, B and Gasiorowski, RE and Orellana, D and McGuire, HM and Groth, BFD and Joshua, DE and Ho, PJ and Larsen, S and Iland, HJ and Gibson, J and Clark, GJ and Fromm, PD and Hart, DNJ	10.1080/2162402X.2017.1419114
	x		A novel costimulatory molecule gene-modified leukemia cell-derived exosome-targeted CD4(+) T cell vaccine efficiently enhances anti-leukemia immunity	Li, JQ and Huang, F and Jiang, Y and Zhao, J and Wan, JB and Hao, SG	10.3389/fimmu.2022.1043484
x			In vitro Generation of Cytotoxic T Cells With Potential for Adoptive Tumor Immunotherapy of Multiple Myeloma	Khalaf, WS and Garg, M and Mohamed, YS and Stover, CM and Browning, MJ	10.3389/fimmu.2019.01792
		x	Immunoreactivity to WT1 peptide vaccine is associated with prognosis in elderly patients with acute myeloid leukemia: follow-up study of randomized phase II trial of OCV-501, an HLA class II-binding WT1 polypeptide	Naoe, T and Saito, A and Hosono, N and Kasahara, S and Muto, H and Hatano, K and Ogura, M and Masunari, T and Tanaka, M and Usuki, K and Ishikawa, Y and Ando, K and Kondo, Y and Takagi, Y and Takada, S and Ishikawa, M and Choi, I and Sano, A and Nagai, H	10.1007/s00262-023-03432-4
	x		Development of the (T-ALL)iPSC-based therapeutic cancer vaccines for T-cell acute lymphoblastic leukemia	Li, Z and Chen, XM and Liu, LN and Zhou, ML and Zhou, GQ and Liu, T	10.1007/s12032-022-01809-6
x			Effect of thymosin alpha(1) on the phenotypic and functional maturation of dendritic cells from children with acute lymphoblastic leukemia	Li, XR and Liu, XD and Zhao, YX and Zhong, R and Song, AQ and Sun, LR	10.3892/mmr.2015.4153
x			Clinical Grade Production of Wilms' Tumor-1 Loaded Cord Blood-Derived Dendritic Cells to Prevent Relapse in Pediatric AML After Cord Blood Transplantation	Plantinga, M and Lo Presti, V and de Haar, CG and Dunnebach, E and Madrigal, A and Lindemans, CA and Boelens, JJ and Nierkens, S	10.3389/fimmu.2020.559152
x			Generation of a cord blood-derived Wilms Tumor 1 dendritic cell vaccine for AML patients treated with allogeneic cord blood transplantation	de Haar, C and Plantinga, M and Blokland, NJG and van Til, NP and Flinsenberg, TWH and Van Tendeloo, VF and Smits, EL and Boon, L and Spel, L and Boes, M and Boelens, JJ and Nierkens, S	10.1080/2162402X.2015.1023973
x			The HLA ligandome landscape of chronic myeloid leukemia delineates novel T-cell epitopes for immunotherapy	Bilich, T and Nelde, A and Bichmann, L and Roerden, M and Salih, HR and Kowalewski, DJ and Schuster, H and Tsou, CC and Marcu, A and Neidert, MC and Lubke, M and Rieth, J and Schemionek, M and Brummendorf, TH and Vucinic, V and Niederwieser, D and Bauer, J and Marklin, M and Peper, JK and Klein, R and Kohlbacher, O and Kanz, L and Rammensee, HG and Stevanovic, S and Walz, JS	10.1182/blood-2018-07-866830
x			Short-term cultured autologous peripheral blood mononuclear cells as a potential immunogen to activate Tax-specific CTL response in adult T-cell leukemia patients	Ishizawa, M and Ganbaatar, U and Hasegawa, A and Takatsuka, N and Kondo, N and Yoneda, T and Katagiri, K and Masuda, T and Utsunomiya, A and Kannagi, M	10.1111/cas.14800
		x	Tumoricidal efficacy coincides with CD11c up-regulation in antigen-specific CD8(+) T cells during vaccine immunotherapy	Takeda, Y and Azuma, M and Matsumoto, M and Seya, T	10.1186/s13046-016-0416-x
x			A novel vaccine for mantle cell lymphoma based on targeting cyclin D1 to dendritic cells via CD40	Chen, JT and Zurawski, G and Zurawski, S and Wang, ZQ and Akagawa, K and Oh, S and Hideki, U and Fay, J and Banchereau, J and Song, WR and Palucka, AK	10.1186/s13045-015-0131-7
x			A High-avidity WT1-reactive T-Cell Receptor Mediates Recognition of Peptide and Processed Antigen but not Naturally Occurring WT1-positive Tumor Cells	Jaigirdar, A and Rosenberg, SA and Parkhurst, M	10.1097/CJI.0000000000000116
x			Cytokine Release Patterns in Mixed Lymphocyte Culture (MLC) of T-Cells with Dendritic Cells (DC) Generated from AML Blasts Contribute to Predict anti-Leukaemic T-Cell Reactions and Patients' Response to Immunotherapy	Fischbacher, D and Merle, M and Liepert, A and Grabrucker, C and Kroell, T and Kremser, A and Dreyssig, J and Freudenreich, M and Schuster, F and Borkhardt, A and Kraemer, D and Koehne, CH and Kolb, HJ and Schmid, C and Schmetzer, HM	10.1080/15419061.2016.1223634
	x		mRNA vaccination with charge-altering releasable transporters elicits human T cell responses and cures established tumors in mice	Haabeth, OAW and Blake, TR and McKinlay, CJ and Waymouth, RM and Wender, PA and Levy, R	10.1073/pnas.1810002115
x			HLA class I-restricted MYD88 L265P-derived peptides as specific targets for lymphoma immunotherapy	Nelde, A and Walz, JS and Kowalewski, DJ and Schuster, H and Wolz, OO and Peper, JK and Gloria, YC and Langerak, AW and Muggen, AF and Claus, R and Bonzheim, I and Fend, F and Salih, HR and Kanz, L and Rammensee, HG and Stevanovic, S and Weber, ANR	10.1080/2162402X.2016.1219825
x			Expansion and CD2/CD3/CD28 stimulation enhance Th2 cytokine secretion of human invariant NKT cells with retained anti-tumor cytotoxicity	Andrews, K and Hamers, AAJ and Sun, XD and Neale, G and Verbist, K and Tedrick, P and Nichols, KE and Pereira, S and Geraghty, DE and Pillai, AB	10.1016/j.jcyt.2020.01.011
x			Interleukin-15-Cultered Dendritic Cells Enhance Anti-Tumor Gamme Delta T Cell Functions through IL-15 Secretion	Van Acker, HH and Anguille, S and De Reu, H and Berneman, ZN and Smits, EL and Van Tendeloo, VF	10.3389/fimmu.2018.00658
x			Transfer of Cellular Content from the Allogeneic Cell-Based Cancer Vaccine DCP-001 to Host Dendritic Cells Hinges on Phosphatidylserine and Is Enhanced by CD47 Blockade	Zuo, HX and Van Lierop, MJC and Kaspers, J and Bos, R and Reurs, A and Sarkar, S and Konry, T and Kamermans, A and Kooij, G and de Vries, HE and de Gruijl, TD and Karlsson-Parra, A and Manting, EH and Kruisbeek, AM and Singh, SK	10.3390/cells10113233
	x		siRNA silencing of PD-1 ligands on dendritic cell vaccines boosts the expansion of minor histocompatibility antigen-specific CD8(+) T cells in NOD/SCID/IL2Rg(null) mice	van der Waart, AB and Fredrix, H and van der Voort, R and Schaap, N and Hobo, W and Dolstra, H	10.1007/s00262-015-1668-6
x			Chronic Myelocytic Leukemia (CML) Patient-Derived Dendritic Cells Transfected with Autologous Total RNA Induces CML-Specific Cytotoxicity	Yu, L and Hu, T and Zou, T and Shi, QZ and Chen, GA	10.1007/s12288-016-0643-5
	x		Pre-clinical development of gene modification of haematopoietic stem cells with chimeric antigen receptors for cancer immunotherapy	Larson, SM and Truscott, LC and Chiou, TT and Patel, A and Kao, R and Tu, A and Tyagi, T and Lu, X and Elashoff, D and De Oliveira, SN	10.1080/21645515.2016.1268745
x			An immunogenic WT1-derived peptide that induces T cell response in the context of HLA-A*02:01 and HLA-A*24:02 molecules	Dao, T and Korontsvit, T and Zakhaleva, V and Jarvis, C and Mondello, P and Oh, C and Scheinberg, DA	10.1080/2162402X.2016.1252895
x			Novel IL-15 dendritic cells have a potent immunomodulatory effect in immunotherapy of multiple myeloma	Chu, TH and Vo, MC and Lakshmi, TJ and Ahn, SY and Kim, M and Song, GY and Yang, DH and Ahn, JS and Kim, HJ and Jung, SH and Lee, JJ	10.1016/j.tranon.2022.101413
x			Mass spectrometry-based identification of a B-cell maturation antigen-derived T-cell epitope for antigen-specific immunotherapy of multiple myeloma	Bilich, T and Nelde, A and Bauer, J and Walz, S and Roerden, M and Salih, HR and Weisel, K and Besemer, B and Marcu, A and Lubke, M and Schuhmacher, J and Neidert, MC and Rammensee, HG and Stevanovic, S and Walz, JS	10.1038/s41408-020-0288-3
	x		Induction of WT1-specific human CD8(+) T cells from human HSCs in HLA class I Tg NOD/SCID/IL2rgKO mice	Najima, Y and Tomizawa-Murasawa, M and Saito, Y and Watanabe, T and Ono, R and Ochi, T and Suzuki, N and Fujiwara, H and Ohara, O and Shultz, LD and Yasukawa, M and Ishikawa, F	10.1182/blood-2014-10-604777
	x		Development of CMV-CD19 bi-specific CAR T cells with post-infusion in vivo boost using an anti-CMV vaccine	Wang, XL and Diamond, DJ and Forman, SJ and Nakamura, R	10.1007/s12185-021-03215-6
	x		A randomized phase 2 trial of idiotype vaccination and adoptive autologous T-cell transfer in patients with multiple myeloma	Qazilbash, MH and Saini, NY and Cha, SC and Wang, Z and Stadtmauer, EA and Baladandayuthapani, V and Lin, H and Tross, B and Honhar, M and Rao, SS and Kim, K and Popescu, M and Szymura, S and Zhang, TT and Anderson, A and Bashir, Q and Shpall, EJ and Orlowski, RZ and Levine, BL and Kerr, N and Garfall, A and Cohen, A and Vogl, DT and Dengel, K and June, CH and Champlin, R and Kwak, LW	10.1182/blood.2020008493
x			Optimization of Liposomes for Antigen Targeting to Splenic CD169(+) Macrophages	Twilhaar, MKN and Czentner, L and Grabowska, J and Affandi, AJ and Lau, CYJ and Olesek, K and Kalay, H and van Nostrum, CF and van Kooyk, Y and Storm, G and den Haan, JMM	10.3390/pharmaceutics12121138
x			DC generation from peripheral blood mononuclear cells in patients with chronic myeloid leukemia: Influence of interferons on DC yield and functional properties	Florcken, A and Kopp, J and Kolsch, U and Meisel, C and Dorken, B and Pezzutto, A and Westermann, J	10.1080/21645515.2015.1132965
		x	Colloidal Assemblies Composed of Polymeric Micellar/Emulsified Systems Integrate Cancer Therapy Combining a Tumor-Associated Antigen Vaccine and Chemotherapeutic Regimens	Huang, CY and Lin, SY and Hsu, TA and Hsieh, HP and Huang, MH	10.3390/nano11071844
	x		K562-Derived Whole-Cell Vaccine Enhances Antitumor Responses of CAR-Redirected Virus-Specific Cytotoxic T Lymphocytes In Vivo	Caruana, I and Weber, G and Ballard, BC and Wood, MS and Savoldo, B and Dotti, G	10.1158/1078-0432.CCR-14-2998
x			Selective targeting of multiple myeloma by B cell maturation antigen (BCMA)-specific central memory CD8(+) cytotoxic T lymphocytes: immunotherapeutic application in vaccination and adoptive immunotherapy	Bae, J and Samur, M and Richardson, P and Munshi, NC and Anderson, KC	10.1038/s41375-019-0414-z
		x	Antibody Opsonization of a TLR9 Agonist-Containing Virus-like Particle Enhances In Situ Immunization	Lemke-Miltner, CD and Blackwell, SE and Yin, C and Krug, AE and Morris, AJ and Krieg, AM and Weiner, GJ	10.4049/jimmunol.1900742
		x	Intratumoral delivery of antigen with complement C3-bound liposomes reduces tumor growth in mice	Francian, A and Namen, S and Stanley, M and Mann, K and Martinson, H and Kullberg, M	10.1016/j.nano.2018.10.009
x			Different In Vitro-Generated MUTZ-3-Derived Dendritic Cell Types Secrete Dexosomes with Distinct Phenotypes and Antigen Presentation Potencies	Sakamoto, T and Koya, T and Togi, M and Yoshida, K and Kato, T and Ishigaki, Y and Shimodaira, S	10.3390/ijms23158362
x			Transpresentation of interleukin-15 by IL-15/IL-15Ra mRNA-engineered human dendritic cells boosts antitumoral natural killer cell activity	Van den Bergh, J and Willemen, Y and Lion, E and Van Acker, H and De Reu, H and Anguille, S and Goossens, H and Berneman, Z and Van Tendeloo, V and Smits, E	10.18632/oncotarget.6536
	x		Identification of CD8(+) T-cell epitope from multiple myeloma-specific antigen AKAP4	Ma, N and Liu, HH and Zhang, Y and Liu, W and Liang, ZY and Wang, Q and Sun, YH and Wang, LH and Li, Y and Ren, HY and Dong, YJ	10.3389/fimmu.2022.927804
	x		A transplant "immunome" screening platform defines a targetable epitope fingerprint of multiple myeloma	Schieferdecker, A and Oberle, A and Thiele, B and Hofmann, F and Gothel, M and Miethe, S and Hust, M and Braig, F and Voigt, M and von Pein, UM and Koch-Nolte, F and Haag, F and Alawi, M and Indenbirken, D and Grundhoff, A and Bokemeyer, C and Bacher, U and Kroger, N and Binder, M	10.1182/blood-2015-10-676536
		x	Live attenuated VZV vaccination induces antitumor immunity in ATLL patients	Jo, T and Kubota-Koketsu, R and Kaneko, Y and Sakai, T and Noguchi, K and Irie, S and Matsuo, M and Taguchi, J and Abe, K and Shigematsu, K	10.1007/s00262-022-03301-6
x			In situ loading of skin dendritic cells with apoptotic bleb-derived antigens for the induction of tumor-directed immunity	Ruben, JM and Bontkes, HJ and Westers, TM and Hooijberg, E and Ossenkoppele, GJ and van de Loosdrecht, AA and de Gruijl, TD	10.4161/21624011.2014.946360
x			Lentiviral Gene Therapy Combined with Low-Dose Busulfan in Infants with SCID-X1	Mamcarz, E and Zhou, S and Lockey, T and Abdelsamed, H and Cross, SJ and Kang, G and Ma, ZJ and Condori, J and Dowdy, J and Triplett, B and Li, C and Maron, G and Becerra, JCA and Church, JA and Dokmeci, E and Love, JT and Ain, ACD and van der Watt, H and Tang, X and Janssen, W and Ryu, BY and De Ravin, SS and Weiss, MJ and Youngblood, B and Long-Boyle, JR and Gottschalk, S and Meagher, MM and Malech, HL and Puck, JM and Cowan, MJ and Sorrentino, BP	10.1056/NEJMoa1815408
x			PGE(2)-Induced IDO1 Inhibits the Capacity of Fully Mature DCs to Elicit an In Vitro Antileukemic Immune Response	Trabanelli, S and Lecciso, M and Salvestrini, V and Cavo, M and Ocadlikova, D and Lemoli, R and Curti, A	10.1155/2015/253191
x			In vitro polyclonal activation of conventional T cells with a CD28 superagonist protects mice from acute graft versus host disease	Beyersdorf, N and Werner, S and Wolf, N and Hunig, T and Kerkau, T	10.1002/eji.201445317
			Targeting LAG3/GAL-3 to overcome immunosuppression and enhance anti-tumor immune responses in multiple myeloma	Bae, J and Accardi, F and Hideshima, T and Tai, YT and Prabhala, R and Shambley, A and Wen, K and Rowell, S and Richardson, PG and Munshi, NC and Anderson, KC	10.1038/s41375-021-01301-6
